# Chromosome-level reference genome and alternative splicing atlas of moso bamboo (*Phyllostachys edulis*)

**DOI:** 10.1093/gigascience/giy115

**Published:** 2018-09-08

**Authors:** Hansheng Zhao, Zhimin Gao, Le Wang, Jiongliang Wang, Songbo Wang, Benhua Fei, Chunhai Chen, Chengcheng Shi, Xiaochuan Liu, Hailin Zhang, Yongfeng Lou, LianFu Chen, Huayu Sun, Xianqiang Zhou, Sining Wang, Chi Zhang, Hao Xu, Lichao Li, Yihong Yang, Yanli Wei, Wei Yang, Qiang Gao, Huanming Yang, Shancen Zhao, Zehui Jiang

**Affiliations:** 1 State Forestry Administration Key Open Laboratory on the Science and Technology of Bamboo and Rattan, Institute of Gene Science for Bamboo and Rattan Resources, International Center for Bamboo and Rattan, Futongdong Rd, WangJing, Chaoyang District Beijing 100102, China; 2 BGI Genomics, BGI-Shenzhen, Building No. 7, BGI Park, No. 21 Hongan 3rd Street, Yantian District, Shenzhen 518083, China; 3 Department of Plant Sciences, University of California, Davis, One Shield Avenue, Davis, CA 95617, USA; 4 BGI Institute of Applied Agriculture, BGI-Shenzhen, No. 7 PengFei Rd, Dapeng District, Shenzhen 518120, China; 5 BGI-Qingdao, No. 2877, Tuanjie Rd, Sino-German Ecopark, Qingdao, Shandong Province, 266555, China

**Keywords:** moso bamboo, genome, annotation, alternative splicing, transcriptome, evolution

## Abstract

**Background:**

Bamboo is one of the most important nontimber forestry products worldwide. However, a chromosome-level reference genome is lacking, and an evolutionary view of alternative splicing (AS) in bamboo remains unclear despite emerging omics data and improved technologies.

**Results:**

Here, we provide a chromosome-level *de novo* genome assembly of moso bamboo (*Phyllostachys edulis*) using additional abundance sequencing data and a Hi-C scaffolding strategy. The significantly improved genome is a scaffold N50 of 79.90 Mb, approximately 243 times longer than the previous version. A total of 51,074 high-quality protein-coding loci with intact structures were identified using single-molecule real-time sequencing and manual verification. Moreover, we provide a comprehensive AS profile based on the identification of 266,711 unique AS events in 25,225 AS genes by large-scale transcriptomic sequencing of 26 representative bamboo tissues using both the Illumina and Pacific Biosciences sequencing platforms. Through comparisons with orthologous genes in related plant species, we observed that the AS genes are concentrated among more conserved genes that tend to accumulate higher transcript levels and share less tissue specificity. Furthermore, gene family expansion, abundant AS, and positive selection were identified in crucial genes involved in the lignin biosynthetic pathway of moso bamboo.

**Conclusions:**

These fundamental studies provide useful information for future in-depth analyses of comparative genome and AS features. Additionally, our results highlight a global perspective of AS during evolution and diversification in bamboo.

## Background

Bamboo (Bambusoideae) is a fast-growing plant with substantial potential for generating income, restoring degraded landscapes, and combating climate change in numerous Asian and African countries. Approximately 2.5 billion people economically depend on bamboo, reaching an annual international trade of more than $2.5 billion US dollars [1]. Bamboo is a perennial grass in temperate and tropical forests worldwide, with a cellulose and hemicellulose content comparable to that of woody trees [2]. Moso bamboo (*Phyllostachys edulis*) accounts for ∼73.76% of the bamboo-growing regions of China (4.43 million ha), constitutes the most abundant natural resource of nonwood products, and plays significant roles in the economy, ecology, culture, aesthetics, and technology [3].

Only a limited number of genome-wide studies have been performed in bamboo. We first reported a draft genome of moso bamboo in 2013 and released 2.05 Gb of the draft genome with 328 Kb of scaffold N50 and 31,987 predicted genes [4]. Due to advances in sequencing technology and analytical methods, a chromosome-level reference genome with improved precision and contiguity could facilitate functional and evolutionary analyses of bamboo.

Alternative splicing (AS) is a major mechanism underlying the increased complexity and diversity of proteins made from a limited number of genes in eukaryotes [5]. More than 95% of human multiexon genes have been predicted to express multiple splice isoforms [6, 7], and the occurrence of AS events in plants is reported to be ∼61%, ∼52%, ∼42%, ∼40%, ∼40%, and ∼33% in *Arabidopsis thaliana* [8, 9], *Glycine max* [10], *Brachypodium distachyon* [11], *Gossypium raiimondi* [12], *Zea mays* [13], and *Oryza sativa* [14], respectively. The different splicing products of a single gene represent major sources of functional plasticity and supposedly play important roles in plant growth, development, defense responses, signal transduction, and flowering time [15–19]. Species-specific AS is partly responsible for generating a wide variety of functional diversity with limited repertoires of protein-coding genes [20–22]. However, the mechanism by which AS modulates plant evolution is unclear. Moreover, the AS characteristics of genes with differing degrees of conservation remains elusive.

The incomplete and scattered scaffolds of the moso bamboo genome and the low-coverage transcriptomes of a handful of tissues make it difficult to fully dissect AS profiles. Therefore, a high-quality assembled genome and extensive RNA sequencing are critical for comprehensive AS identification. Thus, we substantially improved the moso bamboo genome assembly and gene annotation and performed a comprehensive genome-wide analysis to uncover AS profiles in bamboo using transcriptome data from 26 mixed samples collected from six major bamboo-producing areas in China. These transcriptome data were generated using the Illumina and Pacific Biosciences (PacBio) platforms. Numerous AS genes and events were detected, and various types of AS events were identified. We performed a genome-wide investigation to determine the relationship between amino acid conservation and AS and to examine the evolution of the AS status of genes that are involved in lignin biosynthesis. In conclusion, our analysis not only provides a global profile of AS in bamboo for further experimental studies investigating the functions of genes and regulatory networks but also reveals the roles of AS from an evolutionary perspective.

## Data Description

For the assembly of the moso bamboo genome, ∼603.3 Gb of genome data were generated using different sequencing strategies. Whole-genome sequence (WGS) assembly was performed using ∼154 Gb of newly acquired and ∼220 Gb of previously acquired clean data [4]. Hi-C assembly was performed using ∼157 Gb of raw data from a Hi-C library, and ∼17.58 Gb of valid reads were obtained after quality control (Supplementary Table S1). Additionally, for transcriptomic analysis, ∼379 Gb and ∼5 Gb of raw data were produced from the Illumina and PacBio platforms, respectively (Supplementary Tables S2–S7). Thus, we identified 266,711 unique AS events in 25,225 AS genes in moso bamboo according to the chromosome-level genome reference and the high-throughput transcriptome data.

## Analyses

### Chromosome-level genome assembly and gene annotation in moso bamboo

**Figure 1:**
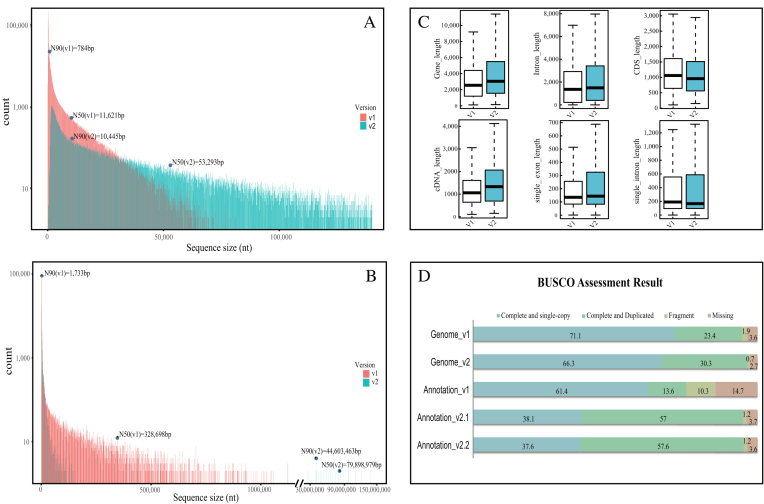
Comparative results based on two versions of the moso bamboo genome. **(A)** The distribution of contigs between two versions of the moso bamboo genome. Contig N50 and N90 are marked. **(B)** The distribution of scaffolds between two versions of the moso bamboo genome. Scaffold N50 and N90 are masked. **(C)** Box plots comparing the two versions of the moso bamboo genome, including gene length, intron length, corresponding coding sequence length, cDNA length, single exon length, and single intron length. **(D)** The Benchmarking Universal Single-Copy Orthologs (BUSCO) assessment result was provided, including the five assessment results (two genomes and three annotations). The two genomes contained the previous WGS version and the latest chromosome-level version. Annotation v1 was based on version 1 of the moso bamboo genome; annotation v2.1 was based on version 2; and annotation v2.2 was the manually verified version of Annotation v2.1.

To enhance the quality of the moso bamboo genome, 61 libraries were used and subjected to sequencing according to the instructions of the sequencer manufacturer (Supplementary Table S1). In total, we obtained ∼603.3 Gb of genome data with read lengths ranging from 76 bp to 250 bp. Subsequently, we applied different assembly strategies to obtain a better genome assembly (see the Additional File for details). First, the WGS assembly reached 1.91 Gb with a contig and scaffold N50 length of 55 Kb and 894 Kb, respectively (Supplementary Table S8). Compared with our previous version [4], the assembly statistics and quality of the new WGS assembly were clearly improved (Supplementary Tables S9 and S10). For example, the scaffold N50 and contig N50 lengths were increased by 172% and 358%, respectively, and the ambiguous base rate was decreased by 43%. Next, the Hi-C assembly was generated with a total length of 1.91 Gb and contig and scaffold N50 lengths of 53.29 Kb and 79.90 Mb (Fig. 1A and B). Approximately 93.17% of scaffolds from the WGS assembly were anchored onto 24 chromosomes (Supplementary Table S10) [23], and the scaffold N50 was increased by ∼89-fold (Table 1). According to the contact map (Supplementary Fig. S1) and the assembly results, the boundaries between the 24 chromosomes were clearly observed. We then aligned the moso bamboo chromosomes to the rice genome and found a mean coverage of ∼59.77% (Supplementary Fig. S2 and Table S11). Additionally, we evaluated the chromosome-level assembly using bamboo-derived Bacterial Artificial Chromosome (BAC) sequences, full-length cDNAs (FL-cDNAs) [24], and some known genes (Supplementary Fig. S3 and Tables S12–S14). The chromosome-level assembly displayed more extensive genome coverage, and the accuracy was higher than that of the first assembly.

**Table 1:** Statistics for the assembly of the moso bamboo genome using different sequencing data

Statistics	WGS assembly		Hi-C assembly	
	Scaffold	Contig	Scaffold	Contig
Total number	19,285	76,900	19,684	84,758
Genome size (bp)	1,908,074,089	1,795,528,836	1,907,603,590	1,795,510,437
Gap number (bp)	112,545,253	0	112,093,153	0
Average length (bp)	98,940.84	23,348.88	96,911.38	21,183.96
N50 length (bp)	894,858	54,955	79,898,979	53,293
N90 length (bp)	115,487	11,757	44,603,463	10,445
Maximum length (bp)	5,406,526	738,589	137,299,170	738,589
Minimum length (bp)	926	157	318	1
GC content (%)	44.2	44.2	44.2	44.2

The chromosome-level assembly generated here can facilitate gene prediction in subsequent analyses after annotation of repetitive sequences (Supplementary Table S15). Based on numerous transcriptomic data (Supplementary Table S16), full-length cDNAs [24], and homologous proteins, we predicted 51,074 high-quality protein-coding loci with intact structures in moso bamboo (Supplementary Table S17). Average intron and exon length were 668 bp and 284 bp, respectively (Fig. 1C and Supplementary Table S18). A combination of single-molecule real-time sequencing and manual verifications was carried out to confirm or correct certain irregular predictions. Approximately 17% of the gene models were improved by untranslated region addition and internal structural adjustment (Supplementary Table S19). According to the completeness assessment of the annotation using Benchmarking Universal Single-Copy Orthologs [25], moso bamboo (95.2%) was more complete than *Z. mays* (92.2%) but close to *O. sativa* (95.6%) (Fig. 1D and Supplementary Table S20). Compared with the previous annotation, 97.23% of the gene models in our analysis were identified in public databases, which facilitated the accurate detection of AS events (Supplementary Table S21). Detailed information regarding gene model prediction and genome evolution are presented in Supplementary Tables S22–S24 and Figs. S3–S9. Additionally, the latest genome assembly and gene annotation were released via the GigaScience *Giga*DB repository [26]. The entire dataset comprises the newly released bamboo genome sequence, gene sets, repeat elements, tRNAs, miRNAs, and gene clusters, providing a reliable resource for many analyses, including genomic, genetic, and molecular biology experiments.

### Vast transcriptomic data generated using the Illumina and PacBio platforms

To facilitate the genome-wide investigation of AS profiles in moso bamboo and to comprehensively identify the factors influencing AS at the posttranslational level, we performed high-throughput RNA sequencing (RNA-seq) using the Illumina HiSeq-4000 platform. In total, 26 individual representative RNA samples were sequenced (150 bp of paired-ends; Supplementary Table S2 and Figs. S10 and S11). After preprocessing, we obtained an average of 90 million high-quality reads (∼13.6 Gb) per sample, accounting for 92.78% of the raw reads. Approximately 80.57% of the high-quality reads were mapped to the reference genome at a unique position and designated as unique reads (Supplementary Tables S3 and S4). According to the alignment distribution, most sequences were mapped in exonic regions. The exon-mapping rate was on average 81.94%. The remaining reads were mapped in intronic regions (8.46%) and intergenic regions (9.6%) (Supplementary Table S5 and Figs. S12 and S13). The exonic coverage was found to be ∼2521× per sample (Supplementary Fig. S14). Therefore, these large-scale, in-depth, high-quality transcriptomic data, together with a high-quality reference genome, will likely contribute to accurate AS identification in moso bamboo.

To accurately identify full-length splice isoforms, we sequenced the bamboo transcriptome using the PacBio platform. FL-cDNA sequencing of alternatively spliced isoforms (Iso-Seq) used RNA from a mixture of 26 samples. According to the length distributions of the transcripts in all samples (Supplementary Table S6), we constructed three single-molecular real-time Bell (SMRTBell) libraries (1–2 kb, 2–3 kb, and >3 kb) for the mixed sample and sequenced nine cells, generating ∼5 Gb of raw data and 214,372 reads-of-insert (ROIs), including 133,599 full-length ROIs (containing a 5′ primer, 3′ primer, and a poly(A) tail); the remaining ROIs were non-full-length ROIs (Supplementary Table S7 and Fig. S15). Accuracy evaluation based on aligning the ROIs against the new genome showed that the per-nucleotide error was approximately 2.05% and consisted of mismatches (0.32%), insertions (0.98%), and deletions (0.75%).

### Numerous genes undergo AS in moso bamboo

Based on the improved reference genome and large-scale transcriptome data, we performed a genome-wide analysis to identify AS in moso bamboo using a previously described pipeline [10]. In total, 266,711 unique AS events were identified in 25,225 AS genes, accounting for *ca*. 49.39% of all annotated genes. Except for the 12,653 AS genes identified in the gene annotation, the remaining (12,572 genes) were considered novel AS genes (Supplementary Fig. S16).

**Figure 2:**
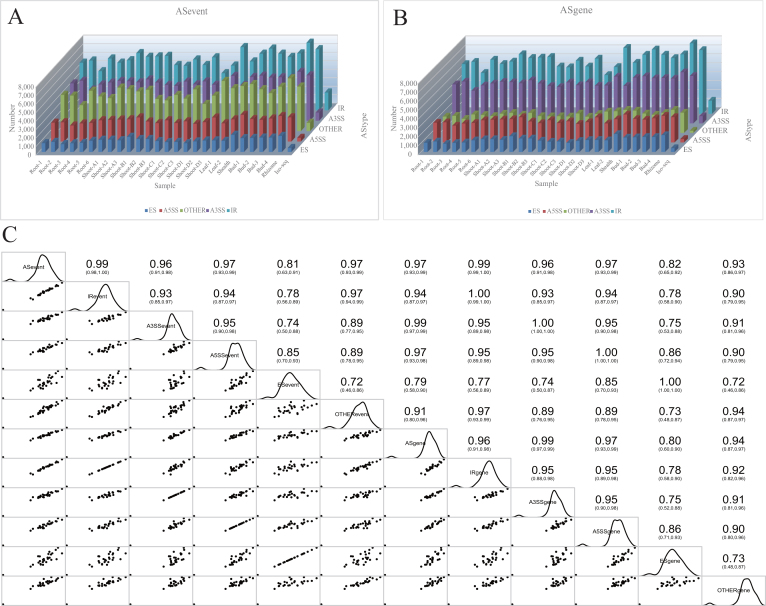
The distribution of AS genes and events and their correlation. **(A)** The distribution of AS genes in bamboo, including the four main types and Iso-Seq results. **(B)** The distribution of AS events in bamboo, including the four main types and Iso-Seq results. **(C)** The correlation between AS genes and events. IR, A3SS, A5SS, and ES represent intron retention, alternative 3′ splice site donor, alternative 5′ splice site acceptor, and exon skipping, respectively.

The Iso-Seq data were also utilized to detect AS in an analysis parallel to the Illumina RNA-seq analysis. In total, 4,246 AS events and 2,218 AS genes were identified (Fig. 2A, B). According to the PacBio-Illumina overlap analysis, which was performed to assess the validity of the AS prediction, 81.21% of the AS events and 97.34% of the AS genes identified in the Iso-Seq analysis completely overlapped with those in the RNA-seq analysis. In subsequent analyses, we defined the four main AS types as intron retention (IR), alternative 3′ splice site donor (A3SS), alternative 5′ splice site acceptor (A5SS), and exon skipping (ES), and we also defined other AS types that were distinct from the above four main AS types. On average, 80.37% of the AS events and 95.59% of the AS genes overlapped among the four main AS types (Supplementary Fig. S17). The high degree of overlap between the PacBio and Illumina AS genes is a strong indicator of the validity of computationally predicted AS.

The AS event number was strongly and positively correlated with the AS gene number and those among the four main AS types (R^2^ > 0.91, Mann-Whitney *U* test with *P* value < 0.05) (Fig. 2C). The four main AS types were detected in AS events in moso bamboo according to canonical splicing patterns (GT-AG, GC-AG, and AT-AC splice sites). As shown in Fig. 2B, IR (38.22%) represented the most abundant type of AS event, followed by A3SS (20.20%) and A5SS (10.48%). ES (2.92%) was the least prevalent type among the four main AS types.

Regarding the functional implications of AS genes, enrichment analysis showed that 885 genes, which were AS in all samples, were significantly enriched in RNA metabolic processing, mRNA processing, RNA processing, and RNA splicing (Supplementary Table S25). Since AS shows strong tissue and developmental specificity, we identified 181,105 tissue-specific AS events (67.57%), which account for two-thirds of the AS events (termed "among-tissue"). The remaining one-third of AS events were then detected based on comparisons of transcript isoforms within individual tissues (termed "within-tissue") (Supplementary Fig. S18).

Transposable element (TE) analysis showed that 26,366 genes have TE insertion, accounting for 51.62% of all genes, and the total length of TE insertion in genes was ∼46 Mb. According to the different position of the TE-inserted intron, TE-introns were mainly concentrated in the front and rear of a gene (Supplementary Fig. S19). Additionally, the usage and distribution of splice sites revealed that GT-AG splice sites were the most abundant, corresponding to 97.31% of all AS events, followed by GC-AG (2.33%) and GT-AT (0.32%) splice sites (Supplementary Fig. S20). In addition to canonical splice sites (GT-AG, GC-AG, and AT-AC), the remaining 2,406 splice sites were identified as noncanonical splice sites, which contained 2,373 GT-AT splice sites and 33 splice sites of other types.

### Evolutionary analysis of AS in moso bamboo

**Figure 3:**
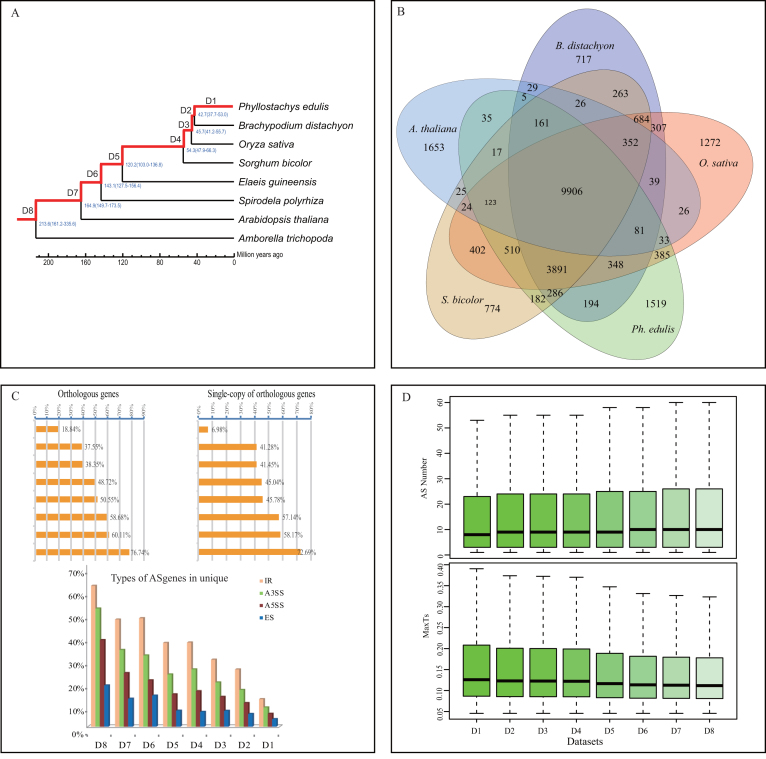
Evolutionary analysis of plant species across bamboo. **(A)** The phylogenetic relationship of *Amborella trichopoda, Elaeis guineensis, Arabidopsis thaliana, Brachypodium distachyon, Oryza sativa, Spirodela polyrhiza, Sorghum bicolor*, and *Ph. edulis*. Phylogenetic tree of the selected eight plant species with branches leading to bamboo represented by the red line. The notation indicates the eight unique orthologous gene datasets (D8-D1) identified in our study. **(B)** A Venn diagram of orthologous genes in the related eight species was exhibited. **(C)** AS percentage and AS type for the two types of eight orthologous genes datasets. **(D)** Increasing AS abundance and the decreasing tissues specificity is displayed in D8-D1.

Based on the genome-wide identification of orthologous genes in the selected eight plant species (*Amborella trichopoda, A. thaliana, Elaeis guineensis, B. distachyon, O. sativa, Spirodela polyrhiza, S. bicolor*, and *Ph. edulis*) and the constructed phylogeny (Fig. 3A and B), we identified eight unique orthologous gene datasets (D8-D1) based on the origination times of genes in each dataset. For instance, unique orthologous gene dataset 7 (D7) contained only orthologous genes that originated between 164.9 million years ago (Mya) and 213.6 Mya (Fig. 3A). In addition, we also extracted single-copy genes from the above datasets, termed D8s-D1s. We considered the bamboo-specific genes (4,023 orthologous genes; termed D1) to be poorly conserved, whereas the genes present in all selected plant species (18,997 orthologous genes; termed D8) are highly conserved. The degree of conservation decreased monotonically from D8 to D1. AS was detected in all the datasets, but the proportion of AS genes in each dataset gradually decreased from D8 to D1 (Mann-Whitney *U* test with *P* value < 0.05). This trend was also observed in the single-copy datasets (D8s-D1s). Therefore, more conserved genes clearly contained more AS genes in bamboo.

**Figure 4:**
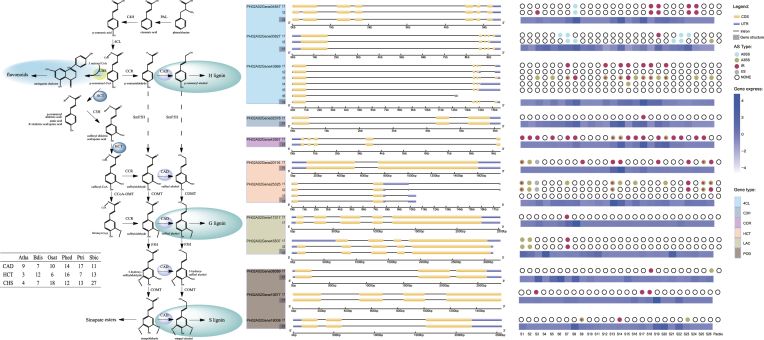
The gene family expansion and AS abundance of bamboo in the lignin biosynthetic pathway. **(A)** A total of 13 gene families in the lignin biosynthetic pathway were identified using six genomes, *i.e*., *A. thaliana, B. distachyon, O. sativa, Ph. edulis, P. trichocarpa*, and *S. bicolor*. The copy number and genes under positive selection were added. **(B)** The structure, distribution, and types of AS and related gene expression levels for the six gene families (4CL, C3H, CCR, HCT, LAC, and POD). The lignin biosynthetic enzymes are PAL, phenylalanine ammonia-lyase; TAL, tyrosine ammonia-lyase; C4H, cinnamate 4-hydroxylase; C3H, 4-hydroxycinnamate 3-hydroxylase; COMT, caffeic acid 3-O-methyltransferase; F5H, ferulate 5-hydroxylase; 4CL, 4-coumarate:CoA ligase; CCoA-3H, coumaroyl-coenzyme A 3-hydroxylase; CCoA-OMT, caffeoyl-coenzyme A O-methyltransferase; CCR, cinnamoyl-CoA reductase; CAD, cinnamyl alcohol; and HCT, dehydrogenase hydroxycinnamoyl transferase.

We investigated the distribution pattern of the four focal AS types in each dataset and found identical trends (Fig. 3C), but the proportion of AS types differed (IR > A3SS > A5SS > ES, Chi-square test with *P* value > 0.86). The proportion of IR in D8 was 57.76%, which was ∼3.4-fold higher than that in D1 (16.95%). The ratio of the other AS types increased as the level of conservation decreased. In the two types of datasets, the number of AS events gradually decreased from D8 to D1 and from D8s to D1s (Fig. 3C). The most abundant AS events appeared in D8, and the least abundant AS events were detected in D1. Additionally, we compared the AS events among the genes expressed in samples with different tissue specificities (maxTs) (for details, see Methods section), where maxTs = 1 and maxTs = 0 represent constitutive expression and tissue-specific expression, respectively. We found that maxTs was negatively correlated with the origination time of the genes in D8-D1 (R^2^ > 0.86 and *P* value < 0.01), representing an enhancement in tissue specificity from the highly conserved gene dataset to the poorly conserved dataset (Fig. 3D). Altogether, the conserved genes tended to have more AS genes, more AS events, and less tissue specificity.

To obtain an overview of the AS perspective and its relationships with gene features, as well as to evaluate the factors that influence AS, we also examined the correlations between AS distribution and gene features in the datasets (Supplementary Fig. S21). All genes in the different datasets (D8-D1) were positively correlated (R^2^ > 0.90 and *P* value < 0.05) with gene length, corresponding coding sequence size, intron size, and exon number and negatively correlated (R^2^ > 0.81 and *P* value < 0.05) with exon cassette length and intron cassette length. Moreover, the distribution of the TE genes in the eight datasets was examined, and a substantially negative correlation was observed (R^2^ > 0.77 and *P* value < 0.05), indicating that the more conserved genes had more TE insertions.

### Expansion of gene families involved in lignin biosynthesis and implications for gene functional diversity

We systematically identified 13 gene families involved in the lignin biosynthetic pathway using genome sequences from *A. thaliana, B. distachyon, O. sativa, Ph. edulis, P. trichocarpa*, and *S. bicolor*. The expansion of most families was detected in bamboo (Supplementary Table S26). Each gene had multiple copies in the bamboo genome, and the total size of the gene families in the lignin biosynthetic pathway was the largest in bamboo, with an average of ∼19 copies per family. The highest and lowest copy numbers were detected in the peroxidase gene family (77 genes) and *p*-coumarate 3-hydroxylase gene family (3 genes), respectively. Additionally, the divergence of genes involved in the lignin biosynthetic pathway (Supplementary Fig. S22) occurred at 5∼16 Mya, which corresponds to a whole-genome duplication (WGD) event 7∼12 Mya in the moso bamboo genome [4].

Moreover, we performed an AS analysis of the genes in the lignin biosynthetic pathway (Fig. 4). In total, 10 of the 13 families had AS genes, accounting for more than half of the total, except for the ferulate 5-hydroxylase gene family, which had a low proportion, and the chalcone synthases (CHS) and caffeic acid *o*-methyltransferase gene families, in which AS genes were not detected. A high percentage (>75%) of AS events was observed in the 4-coumarate:CoA ligase, hydroxycinnamoyl transferase (HCT), and cinnamyl alcohol dehydrogenase (CAD) gene families. In addition, we tested for positive selection in the gene families involved in the lignin biosynthetic pathway using a branch-site model. Several genes in two gene families, e.g., *HCT* and *CAD*, exhibited positive selection. The information provided by the phylogenetic relationship using the best model and log likelihood ratio is provided in Supplementary Table S27.

## Discussion

### The first major update of the moso bamboo genome

High-throughput genome sequencing and improved assembly strategies are broadly applied in current plant genomic studies with the development of new technologies and more useful data. In 2013, our initial analysis of the *Ph. edulis* genome provided a genome-wide perspective on genome and gene structure, the history of WGD events, and functional genes in critical functional categories [4]. In the present study, we enhanced both the precision and contiguity of the *Ph. edulis* genome and updated its annotation, accurately positioning the bamboo genome from an evolutionary perspective by performing comparative studies involving different species. Additionally, various biological characteristics of bamboo were studied in great detail using knowledge obtained from the latest version. Therefore, the chromosome-level reference genome and refined annotation will pave the way for future genomic studies of bamboo and other related plant species.

### AS is common and AS exhibits variation in different tissues of moso bamboo

We provided global AS profiles in bamboo based on a large amount of high-throughput data from RNA-seq and Iso-Seq. These data enabled the accurate detection of transcripts with low expression levels and the acquisition of complete gene structure, particularly in the AS analysis. A series of AS analyses improved our understanding of AS in bamboo during post-transcriptional regulation, including the identification of AS genes and AS events, the distribution of AS types, the use of splice sites, and the length distribution of alternative exons. AS is considered a major mechanism responsible for creating diversity from a limited repertoire of genes. For example, by combining one exon of four alternatively spliced regions that contain 12, 48, 33, and 2 alternative exons each, it is possible to generate, at most, 38,016 protein isoforms (12 × 48 × 33 × 2) from the *Dscam* gene in *Drosophila* [27]. In bamboo, we identified 266,711 unique AS events and 25,225 genes in all samples; on average, 15,971 AS events and 9,080 AS genes were detected in each sample. Thus, AS might be tissue specific, and the actual AS percentages in bamboo might be underestimated. More AS events, supported by transcripts with low expression levels, can be detected as the sequencing depth increases [28].

According to our observations, the rhizome tissue had more AS events than the root tissue in moso bamboo, which may be because the two tissues play different roles during bamboo development. Photoassimilates are unavailable during the rapid growth of the moso bamboo shoots since no leaves are growing [29], and thus, the large amounts of nutrients and energy in the shoot would have to come from the attached matured bamboo through underground rhizomes. Therefore, as a rhizomatous plant, the rhizome in moso bamboo plays a critical role in nutrient and energy transport, which might explain the higher number of AS events detected in the rhizome.

### The characteristics of moso bamboo might be related to the proportion of AS types

Differences in the frequencies or proportions of AS types may reflect differences in pre-mRNA splicing, and this analysis is common in most genome-wide identifications of AS. Analyzing the distribution of AS types revealed that IR was predominant, and the importance of IR can be inferred based on its prevalence throughout evolution in plants. Nevertheless, higher percentages of IR (38.22%) and other AS types (total 28.18%) were observed in bamboo. These higher percentages may be due to the unique features of bamboo and/or the depth of the sequencing, which can be tested in future comparative analyses. Additionally, the distribution of the four main AS types is consistent with that in *Arabidopsis* [5, 9, 28], soybean [10], and maize [13]. However, the allocation in animals and yeast differs from that in plants. The most abundant AS event is ES, followed by A3SS and A5SS, while IR is the least common [30]. The discrepancies in the occurrence of AS models between plants and animals suggest differences between plants and animals in terms of genomic structure and the mechanism of splice site recognition [31]. In addition, the identification of splice sites in an individual gene may provide an essential resource for fully understanding AS and isoform construction [32, 33]. With respect to their distribution, the main AS types (e.g., GT-AG, GC-AG, and AT-AC) were consistent with those observed previously in animals and other plants [19].

### Highly conserved genes with more AS events might play critical roles in evolution and function

We examined the relationship between AS and evolution via comparative genome analysis. To date, the relationship between gene conservation and AS remains unknown. To explore this issue, we performed a genome-wide analysis to examine AS in two types of eight orthologous gene datasets (D8-D1 and D8s-D1s) with different degrees of conservation. AS genes were more likely to be enriched in the highly conserved gene datasets, and these AS genes had more AS events. This finding was robust because we found identical trends in both types of datasets. Previous reports have demonstrated that duplication is a major source of functional diversity and the generation of new genes [34], and conserved genes tend to have higher connectivity in gene-gene interaction networks, indicating their functional importance, while new genes are initially added into gene-gene interaction networks with low connectivity and then gradually increase their connectivity and acquire pleiotropic roles [22, 35]. In our study, highly conserved genes tended to have more AS events than poorly conserved genes, which was consistent with the trend that conserved genes are apt to have higher connectivity in gene-gene interaction networks. Thus, we proposed that AS may be associated with increases in gene connectivity during evolution. Additionally, compared with the poorly conserved gene datasets, the highly conserved AS gene datasets had a low tissue-specific expression profile, indicating that these genes might be critical in fundamental functions, such as having higher connectivity in gene-gene networks. Therefore, we suggested that functionally important genes are generated by more frequent AS events. As an essential biological process, AS plays a crucial role in acquiring more functions, which might explain why highly conserved AS genes possess more AS events. We hypothesize that this phenomenon likely applies not only to bamboo but also to other plants or even animals.

### AS and the expansion of gene families in the lignin biosynthetic pathway in moso bamboo might be related to WGD

Lignin represents a class of complex aromatic heteropolymers of monolignols that encrusts and interacts with the cellulose/hemicellulose matrix of the secondary cell wall [36]. Lignin accounts for up to ∼25% of the total dry weight in bamboo [2]. We performed a thorough examination of the lignin biosynthetic pathway by combining AS and evolutionary analyses. The expansion of gene families in the lignin biosynthetic pathway was detected in bamboo. Combined with the results of the divergence times of lignin biosynthesis genes and our previous study [4], we estimated the occurrence of a putative WGD event at 7∼12 Mya in the moso bamboo genome, suggesting a potentially tetraploidization event some time during bamboo evolution [4]. The ancient tetraploid then evolved into the current diploid moso bamboo. Additionally, WGD can provide more gene copies, facilitating the evolution of genes with new functions [37]. Therefore, the expansion of lignin biosynthetic genes in moso bamboo may be due to the occurrence of a WGD event. Additionally, two gene families (e.g., *HCT* and *CAD*) underwent more AS events and positive selection. HCT generates lignin from *p*-coumaroyl CoA [38], which is also used by CHS to generate flavonoids. HCT and CHS compete with each other to bind *p*-coumaroyl CoA. In bamboo, the *HCT* family has more members and AS events than the *CHS* family, likely indicating that the *HCT* family might be in a dominant position to compete for *p*-coumaroyl CoA binding compared with the *CHS* family. CAD catalyzes many different substrates to generate different types of lignin. The aromatic lignin polymers commonly found in bamboo are composed of three monolignols, namely, *p*-hydroxyphenyl (H), vanillin (G), and syringaldehyde (S). Previous studies have shown the abundance of G and S lignin and a small amount of H lignin in bamboo [2]. The expansion of the *CAD* family in bamboo and the corresponding positive selection may explain the different substrate preferences that generate different proportions of monolignols in bamboo. The abundance of AS events, gene expansion, and positive selection were all consistent with the remarkable adaptability of bamboo in producing lignin.

## Conclusions

To deeply explore the AS profile from an evolutionary perspective in bamboo, we improved the reference genome and refined the annotation of moso bamboo. Based on the chromosome-level genome sequence and the abundant transcriptomic data from multiple tissues from six main bamboo-producing areas in China, we provided a comprehensive analysis of AS in moso bamboo, identifying 266,711 unique AS events in 25,225 AS genes using both Illumina and PacBio sequencing platforms. Moreover, the integrated analysis of the AS results in bamboo, as well as the comparative analysis among eight representative plant species, showed that more conserved genes tended to accumulate higher transcript levels and exhibit less tissue specificity. Finally, by studying lignin biosynthesis from an AS and evolutionary standpoint, we observed several characteristics of crucial genes related to lignin biosynthesis in moso bamboo, including gene family expansion, abundant AS, and positive selection. In summary, these results will likely provide important resources for studies investigating bamboo's unique woodiness in the Grass family (Poaceae) and for exploring AS in bamboo from an evolutionary perspective.

## Methods

### Plant material collection

To obtain a comprehensive AS profile, moso bamboo (*Phyllostachys edulis*) samples used in these experiments were collected from six major bamboo-producing areas in China during the Spring of 2015, including Yixing, Jiangsu Province (N:31°15′08.41″, E:119°43′42.55″, 212 m); Tianmu Mountain, Zhejiang Province (N:30°19′13.42″, E:119°26′55.21″, 480 m); Xianning, Hubei Province (N:29°81′10.02″, E:114°31′21.12″ 150 m); Taojiang, Hunan Province (N:28°28′39.74″, E:112°11′18.62″, 320 m); Guilin, Guangxi Province (N:28°28′39.74″, E:112°11′18.62″, 216 m); and Chishui, Guizhou Province (N:28°28′15.27″, E:105°59′41.43″, 120 m). Twenty-six tissues were collected, including the rhizome, root, shoot, leaf, sheath, and bud, during different developmental stages. Each sample was a mixed sample collected from the above-listed major bamboo-producing areas. Detailed information regarding the biological samples is provided in Supplementary Table S19.

### Genome sequencing, assembly, and annotation

We assembled the moso bamboo genome using the WGS and Hi-C strategies and annotated the new genome sequence as described in a previous study [39] and in the Additional Files. Detailed descriptions for this section are also provided in Protocols.io [40].

### Hi-C library preparation, sequencing, and assembly

The Hi-C library was prepared as previously described [39] and the detailed descriptions are presented in the Additional Files.

### RNA isolation and Illumina RNA-seq library construction

We used standard methods for the RNA isolation, purity and concentration determination, reverse transcription, and cDNA library construction, as described in a previous study [41]. All cDNA libraries were constructed and normalized as described in the Additional Files.

### RNA-seq using the Illumina platform

After quality control, the pooled libraries were optically examined using an Illumina cluster station and were then sequenced on the Illumina HiSeq-4000 platform (150 bp of paired ends) according to the manufacturer's protocols. Finally, the quality of the reads was evaluated, and the low-quality reads were filtered using FastQC (version 0.11.3)(FastQC, RRID:SCR_014583) [42] with the default parameters. The statistics of the key metrics applied to the RNA-seq data were calculated using RNA-SeQC (version1.1.8) [43] with the default parameters.

### RNA-seq data analysis

A detailed description for this section is provided in Protocols.io [44]. Briefly, adaptor sequences and low-quality sequences were trimmed using Trimmomatic (version 0.33) (Trimmomatic, RRID:SCR_011848) [45] during the preprocessing of the RNA-seq data. Then, the cleaned data were mapped to the improved genome using HISAT2 (version 2.0.2) (HiSat2, RRID:SCR_015530) [46] with the following modifications from the default parameters: maximum intron length (4000), specify strand-specific information (RF), and minimum score (L, -0.1, -0.1). Report alignments tailored to the transcript assemblers were allowed. The empirical transcripts in each sample were obtained using Cufflinks (version 2.2.1) (Cufflinks, RRID:SCR_014597) [47] after the reads were aligned. The default parameters were used, except for the following parameters: the minimum isoform fraction (0.05), the small anchor fraction of the spliced reads (0.05), the minimum intron length (20), the maximum intron length (4000), the library type (fr-firststrand), the corrected frag bias, and the corrected multiread. AStalavista (version 4.0) (AStalavista, RRID:SCR_001815) [48, 49] was used with the default parameters to identify the AS genes and events after the different assembled transcript isoforms were mapped to the corresponding gene model using Cuffcompare, which is a component of the Cufflink program. The four main AS types, i.e., IR, A3SS, A5SS, and ES, were analyzed and compared. In addition, an enrichment analysis of the different genes was conducted using Ontologizer (version 2.0) [50] with annotations from the Gene Ontology database (GO, RRID:SCR_002811) [51]. We also calculated the tissue specificity (Ts) values for each sample and each gene based on the expression level (the total number of fragments per kilobase of sequence per million reads mapped). A detailed description is provided in a previous report [52]. Briefly, Ts is defined as the fractional expression of a gene in one sample tissue relative to the sum of its expression in all samples. Thus, the maximum Ts value (maxTs) of a gene serves as an indicator of the tissue specificity. Higher tissue specificity values represent more tissue-specific expression [53].

### Construction and sequencing of the Iso-Seq library

The construction of the Iso-Seq library and sequencing were performed based on the manufacturer's (PacBio) protocol as previously described [54]. According to the length distribution of the transcripts predicted by bioinformatics (Supplementary Table S22), three SMRTBell libraries (1-2 kb of three cells, 2-3 kb of two cells, and >3 kb of four cells) were size-selected, and nine SMRT cells were sequenced on the PacBio platform.

### Iso-Seq data analysis

The sequencing data produced using PacBio RS II were processed to obtain consensus full-length isoforms. The isoforms from the multiple libraries were merged, and redundancy was removed to obtain the final consensus isoforms after processing the reads of the insert, classifying, and clustering. The assembled transcripts were mapped to the reference genome using PASA (version 2.0.2) (PASA, RRID:SCR_014656) [55] with the default parameters. Then, similar to the short-read data, the output file of the gtf was analyzed using AStalavista with the default parameters to identify the AS.

### Evolutionary analysis

We identified gene families, constructed a phylogenetic tree, and predicted divergence times according to a previously study [4]. The detailed information is provided in the Additional Files and Protocols.io [56].

### Genome-wide identification of genes involved in the lignin biosynthetic pathway

The five genome sequences of *A. thaliana* (TAIR10), *B. distachyon* (v3.1), *O. sativa* (v7.0), *Populus trichocarpa* (JGI2.0.31), and *S. bicolor* (v3.1) were downloaded from the ENSEMBL database (Ensembl, RRID:SCR_002344) [57]. According to wide literature-based investigations, 140 genes from the lignin biosynthetic pathway were collected based on experimental validation in previous studies (Supplementary Table S28). Then, these known genes were used as query sequences for further gene identification. We identified lignin biosynthetic genes using a Basic Local Alignment Search Tool (BLAST) search (National Center for Biotechnology Information [NCBI] BLAST, RRID:SCR_004870) and domain analysis as described previously [41, 58]. Briefly, we performed standard protein BLAST searches (version 2.2.26) against the six genome sequences including moso bamboo using the coding sequences of known genes with the following cutoff values: E-value <1e-10, identity >40%, and coverage rate >95% of the query sequence. The filtered sequences were subsequently analyzed by hmmsearch (version 3.1b2) using the Pfam-A.hmm database (released 31 Mar. 2017), and unclear sequences with incomplete domains were discarded after manual correction. Phylogenetic analyses were subsequently carried out [4]. We also calculated the synonymous substitution rate analysis for 13 gene families involved in the lignin biosynthesis using the yn00, which is a package in PAML to estimate the synonymous and nonsynonymous substitution rates. Then, the Ks rate was translated to divergence times using the formula T = Ks/2r (r = 6.5 × 10^−9^).

### Positive selection analysis

We performed a positive selection analysis based on the coding sequences of the lignin biosynthetic pathway genes. In each family, protein sequences were first aligned with PROBCONS (version 1.12)(ProbCons, RRID:SCR_011813) [59] using the default parameters, except for the option of iterative refinement, for which we used 1,000 iterations. Then, we back-translated the protein alignment to its corresponding coding sequences. After obtaining the conserved blocks from the sequence alignment using Gblocks (version 0.91b) [60], jModelTest (version 2.1.6) [61] was used to find the best model according to the Bayesian information criterion. Subsequently, PhyML (version 3.0)(PhyML, RRID:SCR_014629) [62] was used to reconstruct the phylogenetic tree under the best model, with bootstrapping of 1,000 replicates. Finally, certain branches selected from the phylogenetic tree were examined in a positive-selection analysis using PAML (version 4.8) [63] with a branch-site model. Additional details are provided in protocols.io [64].

## Availability of supporting data

The short-read sequencing data from this whole-genome shotgun project were deposited at European Molecular Biology Laboratory under the accession number ERP001340. The RNA-seq raw sequence data and Iso-Seq raw sequence data for a mixture sample were deposited in the NCBI Short Read Archive database under the accession numbers SRX2408703-28 and SRR7032261-69, respectively. The chromosome-level genome and the latest annotation were provided in *Giga*DB [26]. Additionally, protocols for the methods were uploaded to Protocols.io [40, 44, 56, 58, 64].

## Additional files

Additional File-Revised2-30Aug-zhs.docx

Additional Table 25.xls

Additional Table 28.xls

## Abbreviations

A3SS: alternative 3′ splice site donor; A5SS: alternative 5′ splice site acceptor; AS: alternative splicing; BLAST: Basic Local Alignment Search Tool; CAD: cinnamyl alcohol dehydrogenase; CHS: chalcone synthases; ES: exon skipping; FL-cDNAs: full-length cDNAs; HCT: hydroxycinnamoyl transferase; IR: intron retention; Iso-Seq: FL-cDNA sequencing of alternatively spliced isoforms; maxTs: maximum tissue specificity; Mya: Million years ago; NCBI: National Center for Biotechnology Information; PacBio: Pacific Biosciences; RNA-Seq: RNA sequencing; ROI: reads-of-inserts; SMRT: single-molecule real-time; TE: transposable element; Ts: tissue specificity; WGD: whole genome duplication; WGS: whole genome sequencing.

## Competing interests

The authors declare that they have no competing interests.

## Funding

This work received financial support from the Special Fund for Forest Scientific Research in the Public Welfare from State Forestry Administration of China (201504106) and the Sub-Project of National Science and Technology Support Plan of the Twelfth Five-Year in China (2015BAD04B03 and 2015BAD04B01).

## Author contribution

Experimental design: H.Z., Z.G., L.W., C.C., B.F., S.W., Z.C., H.Y., and Z.J. Experimental conduction: H.Z., J.W., H.Z., L.C., Z.X., C.Z., and Y.W. Data analysis: W.Y., H.S., L.L., S.W., Y.Y., Y.L., Q.G., C.C., X.C., and H.X. Providing of reagents, materials, and analysis tools: H.Z. and Z.G. Article writing: H.Z., Z.C., Z.G., and B.F. All of the authors read and approved the final manuscript.

## Supplementary Material

GIGA-D-18-00076_Original_submission.pdf

GIGA-D-18-00076_Revision_1.pdf

GIGA-D-18-00076_Revision_2.pdf

Response_to_Reviewer_Comments_Report_(Original_Submission).pdf

Response_to_Reviewer_Comments_Report_Revision_1.pdf

Reviewer_1_Report_(Original_Submission) -- Cláudio Benício Cardoso-Silva, Ph.D 3/27/2018 Reviewed

Reviewer_1_Report_Revision_1 -- Cláudio Benício Cardoso-Silva, Ph.D 4/26/2018 Reviewed

Reviewer_2_Report_(Original_Submission) -- Fay-Wei Li 4/4/2018 Reviewed

Reviewer_2_Report_Revision_1 -- Fay-Wei Li 5/1/2018 Reviewed

Reviewer_2_Report_Revision_2 -- Fay-Wei Li 8/10/2018 Reviewed

Reviewer_2_Revision_1_attachment.pdf

Reviewer_3_Report_Revision_1 -- Araxi Urrutia 6/22/2018 Reviewed

Supplemental Files
