# Supplementary material for: Chromosome-level reference genome and alternative splicing atlas of moso bamboo (Phyllostachys edulis)
Source: Gigascience. 2018 Sep 8;7(10):giy115. doi: 10.1093/gigascience/giy115 (PMC6204424; doi:10.1093/gigascience/giy115)
Supplement: GIGA-D-18-00076_Original_submission.pdf [file giy115_giga-d-18-00076_original_submission.pdf]

GigaScience

Chromosome-level reference genome and alternative splicing atlas of moso bamboo (Phyllostachys edulis)

--Manuscript Draft--

Manuscript Number:	GIGA-D-18-00076
Full Title:	Chromosome-level reference genome and alternative splicing atlas of moso bamboo (Phyllostachys edulis)
Article Type:	Research
Funding Information:	Special Fund for Forest Scientific Research in the Public Welfare from State Forestry Administration of China (201504106) — Prof. Hansheng Zhao
Abstract:	Background<br>Bamboo is one of the most important non-timber forest products worldwide. However, a chromosome-level reference genome is lacking, and the evolutionary landscape of alternative splicing (AS) in bamboo remains unclear despite emerging data and improved technologies.<br><br>Results<br>Here, we provide a chromosome-level de novo genome assembly of the moso bamboo (Phyllostachys edulis) using additional abundance data and different assembly strategies. The significantly improved genome is a Scaffold N50 of 79.90 Mb, approximately 243 times longer than the previous version, and 51,074 high-quality protein-coding loci with intact structures were identified using single-molecule real-time sequencing and manual verification. Moreover, we provide a comprehensive AS profile based on the identification of 266,771 uniform AS events in 25,225 AS genes by large-scale transcriptomic sequencing of 26 representative bamboo tissues using both the Illumina and PacBio sequencing platforms. Via comparison with orthologous genes in related plants, we observed dramatic evolutionary characteristics, suggesting that the AS genes are concentrated in more conserved genes that tend to accumulate higher expressed transcripts and share less specificity. Furthermore, gene family expansion, abundant AS and positive selection were identified in crucial genes involved in lignin biosynthesis, indicating that moso bamboo's specificity in being a woody plant in the grass family.<br><br>Conclusions<br>These fundamental studies provide useful information for future studies performing in-depth analyses of comparative genome and AS features. Additionally, our results highlight a global perspective of AS during evolution and diversification in bamboo.
Corresponding Author:	Hansheng Zhao<br>International Center for Bamboo and Rattan<br>Beijing, Beijing CHINA
Corresponding Author Secondary Information:	
Corresponding Author's Institution:	International Center for Bamboo and Rattan
Corresponding Author's Secondary Institution:	
First Author:	Hansheng Zhao
First Author Secondary Information:	
Order of Authors:	Hansheng Zhao<br>Chunhai Chen<br>Benhua Fei<br>Songbo Wang

Powered by Editorial Manager® and ProduXion Manager® from Aries Systems Corporation

	Chengcheng Shi
	XiaoChuan Liu
	Hailin Zhang
	Yongfeng Lou
	Lianfu Chen
	Huayu Sun
	Xianqiang Zhou
	Sining Wang
	Chi Zhang
	Hao Xu
	Lichao Li
	Yihong Yang
	Yanli Wei
	Wei Yang
	Qiang Gao
	Huanming Yang
	Zhimin Gao
	Shancen Zhao
	Zehui Jiang
**Order of Authors Secondary Information:**	
**Opposed Reviewers:**	
**Additional Information:**	
**Question**	**Response**
Are you submitting this manuscript to a special series or article collection?	No
**Experimental design and statistics**<br><br>Full details of the experimental design and statistical methods used should be given in the Methods section, as detailed in our Minimum Standards Reporting Checklist. Information essential to interpreting the data presented should be made available in the figure legends.<br><br>Have you included all the information requested in your manuscript?	Yes
**Resources**<br><br>A description of all resources used, including antibodies, cell lines, animals and software tools, with enough information to allow them to be uniquely	Yes

Powered by Editorial Manager® and ProduXion Manager® from Aries Systems Corporation

identified, should be included in the Methods section. Authors are strongly encouraged to cite Research Resource Identifiers (RRIDs) for antibodies, model organisms and tools, where possible.<br><br>Have you included the information requested as detailed in our Minimum Standards Reporting Checklist?	
**Availability of data and materials**<br><br>All datasets and code on which the conclusions of the paper rely must be either included in your submission or deposited in publicly available repositories (where available and ethically appropriate), referencing such data using a unique identifier in the references and in the "Availability of Data and Materials" section of your manuscript.<br><br>Have you have met the above requirement as detailed in our Minimum Standards Reporting Checklist?	Yes

Manuscript Click here to download Manuscript Manuscript-Moso-Feb.docx

Chromosome-level reference genome and alternative splicing atlas of moso bamboo (*Phyllostachys edulis*)

Hansheng Zhao[1#], Chunhai Chen[2#], Benhua Fei[1#], Songbo Wang[2#], Chengcheng Shi[3], Xiaochuan Liu[3], Hailin Zhang[2], Yongfeng Lou[1], LianFu Chen[1], Huayu Sun[1], Xianqiang Zhou[2], Sining Wang[1], Chi Zhang[2], Hao Xu[1], Lichao Li[1], Yihong Yang[1], Yanli Wei[2], Wei Yang[2], Qiang Gao[2], Huanming Yang[2], Zhimin Gao[1+], Shancen Zhao[2+] and Zehui Jiang[1+]

[1] State Forestry Administration Key Open Laboratory on the Science and Technology of Bamboo and Rattan, Institute of Gene Science for Bamboo and Rattan Resources, International Center for Bamboo and Rattan, Futongdong Rd, WangJing, Chaoyang District Beijing 100102, China;

[2] BGI Genomics, BGI-Shenzhen, Building NO.7, BGI Park, No. 21 Hongan 3rd Street, Yantian District, Shenzhen 518083, China;

[3] BGI-Qingdao, No. 2877, Tuanjie Road, Sino-German Ecopark, Qingdao, Shandong Province, 266555, China;

[+] Co-corresponding author: gaozhimin@icbr.ac.cn, zhaoshancen@genomics.cn, and jiangzehui@icbr.ac.cn

[#] These authors contributed equally to this work.

Abstract

Background

Bamboo is one of the most important non-timber forest products worldwide. However, a chromosome-level reference genome is lacking, and the evolutionary landscape of alternative splicing (AS) in bamboo remains unclear despite emerging data and improved technologies.

Results

Here, we provide a chromosome-level *de novo* genome assembly of the moso bamboo (*Phyllostachys edulis*) using additional abundance data and different assembly strategies. The significantly improved genome is a scaffold N50 of 79.90 Mb, approximately 243 times longer than the previous version, and 51,074 high-quality protein-coding loci with intact structures were identified using single-molecule real-time sequencing and manual verification. Moreover, we provide a comprehensive AS profile based on the identification of 266,771 uniform AS events in 25,225 AS genes by large-scale transcriptomic sequencing of 26 representative bamboo tissues using both the Illumina and PacBio sequencing platforms. Via comparison with orthologous genes in related plants, we observed dramatic evolutionary characteristics, suggesting that the AS genes are concentrated in more conserved genes that tend to accumulate higher expressed transcripts and share less specificity. Furthermore, gene family expansion, abundant AS and positive selection were identified in crucial genes involved in lignin biosynthesis, indicating that moso bamboo's specificity in being a woody plant in the grass family.

Conclusions

These fundamental studies provide useful information for future studies performing in-depth analyses of comparative genome and AS features. Additionally, our results highlight a global perspective of AS during evolution and diversification in bamboo.

Keywords: Moso Bamboo, Genome, Annotation, Alternative Splicing, Transcriptome, Evolution

Background

Bamboo (Bambusoideae) is a fast-growing plant with substantial potential for generating income, restoring degraded landscapes and combating climate change in numerous Asian and African countries. Approximately 2.5 billion people economically depend on bamboo, reaching an annual international trade of over 2.5 billion US dollars[1]. Bamboo is a perennial grass in temperate and tropical forests worldwide. Its cellulose and hemicelluloses content is comparable to that of woody trees[2]. Moso bamboo (*Phyllostachys edulis*) accounts for ~ 73.76% of the bamboo growing region in China (4.43 million ha), constitutes the most abundant natural resource of non-wood products and plays significant roles in economics, ecology, culture, aesthetics and technology[3].

Only a limited number of genome-wide studies have investigated in bamboo. We first reported a draft genome of moso bamboo in 2013 and released 2.05 Gb of the draft genome with 328 Kb of Scaffold N50 and 31,987 predicted genes[4]. Due to the development of sequencing technology and analytical methods, a chromosome-level reference genome with improved precision and contiguity could facilitate functional and evolutionary analyses of bamboo.

Alternative splicing (AS) is a major mechanism underlying the increased complexity and diversity of proteins made from a limited number of genes in eukaryotes[5]. More than 95% of human multi-exon genes have been predicted to express multiple splice isoforms[6,7], and the occurrence of AS events in plants is reported to be ~61%, ~52%, ~42%, ~40%, ~40% and 33% in *Arabidopsis thaliana*[8,9], *Glycine max*[10], *Brachypodium distachyon*[11], *Gossypium raiimondi*[12], *Zea mays*[13] and *Oryza sativa*[14], respectively. The different splicing products of a single gene represent major sources of functional plasticity and supposedly play important roles in plant growth, development, defense responses, signal transduction and flowering time[15-19]. Species-specific AS is responsible for our colorful dynamic world full of a wide variety of biodiversity with limited repertoires of protein coding genes[20-22]. However, the mechanism by which AS affects some changes in the regulation of the gradual evolutionary process in plants based on genome-wide and deeply transcriptomic analyses is unclear. Moreover, the AS characteristics of genes with a diverse conservative status remains elusive.

In this study, we substantially improved the moso bamboo genome assembly and gene annotation. Based on the improved genome reference, we performed a comprehensive genome-wide analysis to uncover the

AS landscapes in bamboo using transcriptome data from 26 mixed samples collected from six main bamboo producing areas in China. These transcriptome data were generated using the Illumina and PacBio platforms. Numerous AS genes and events were detected, and various types of AS events were identified. We performed a genome-wide investigation to determine the relationship between conservation and AS and between evolution and the AS status of genes that are involved in the biosynthesis of lignin. In conclusion, our analysis not only provides a global profile of AS in bamboo for further experimental studies investigating the functions of genes and regulatory networks but also reveals the roles of AS in the evolutionary landscape.

Data description

For the assembly of the moso bamboo genome, approximately 603.3 Gb genome data with different strategies were generated. The WGS assembly was performed using ~154 Gb of newly acquired and ~220 Gb of previously acquired clean data[4]. The Hi-C assembly was using ~157 Gb raw data from Hi-C library and 17.58 Gb valid reads were obtained after quality control (Additional Table S1). Additionally, for the transcriptomic analysis, approximately 379 Gb and 562 Mb of raw data were produced from the Illumina and PacBio platforms, respectively. Thus, we identified 266,711 uniform AS events in 25,225 AS genes in moso bamboo according to the chromosome-level genome reference and the high-throughput transcriptome data.

Analyses

Chromosome-level genome assembly and gene annotation in moso bamboo

In order to enhance the quality of the moso bamboo genome, a total of 61 libraries were used and subjected to sequencing according to the instructions of the sequencer manufacturer (Additional Table S1). In total, we obtained ~ 603.3 Gb genome data with read length ranging from 76 bp to 250 bp. Subsequently, we performed the genome assembly using different strategies to obtain a better genome assembly. First, the WGS assembly reached 1.91 Gb with a contig and scaffold N50 length of 55 Kb and 894 Kb, respectively (Additional Table S2). Compared with those of our previous version[4], the statistics and quality of the new WGS assembly were obviously improved (Additional Tables S2-3). For example, the length of scaffold N50 and contig N50 were increased by 172% and 358%, respectively, and the 'N' base rate was decreased by 43%. Then, the Hi-C assembly was generated with total length reached 1.91 Gb as well as contig and scaffold

N50 length with 53.29 Kb and 79.90 Mb based on the Hi-C data and the improved WGS assembly. About 93.17% scaffolds from the WGS assembly were anchored onto 24 chromosomes (Additional Table S4) [23]and the scaffold N50 was increased by ~89-folds (Table 1). According to the contact map (Additional Fig. S1) and the assembly results, the boundaries between 24 chromosomes were observed clearly. Then we aligned the moso chromosomes to the rice genome to find a mean coverage of ~59.77% (Additional Fig. S2 and Additional Table S5). Additionally, we evaluated the chromosome-level assembly using bamboo-derived BAC sequences, full-length cDNAs [24] and some known genes (Additional Fig. S3 and Additional Tables S6-8). The chromosome-level assembly had a more extensive genome coverage, and the accuracy was higher than that of the first assembly.

The chromosome-level assembly generated could facilitate gene prediction in subsequent analyses. Based on numerous transcriptomic data (Additional Table S9), full-length cDNAs[24], and homologous proteins, we predicted 51,074 high-quality protein-coding loci with intact structures in moso bamboo (Additional Table S10). The average introns and exons were 668 bp and 284 bp in length, respectively (Fig. 1c and Additional Table S10). Combinations of single-molecule real-time sequencing and manual verifications were implemented to refine certain irrational predictions. According to our results, ~17% of the gene models were precisely refined by the UTR addition and internal structural adjustment (Additional Table S11). According to the completeness assessment of the annotation using BUSCO, moso bamboo (95.2%) was higher than *Z. mays* (92.2%) but close to *O. sativa* (95.6%) (Fig. 1d and Additional Table S12). Compared with the previous annotation, 97.23% of the gene models in our analysis were identified in public databases, which facilitated the accurate detection of alternative splicing events (Additional Table S13). Detailed information regarding the gene model prediction and genome evolution are presented in Additional Tables S14-18 and Figs. S3-9. Additionally, the latest genome assembly and gene annotation were released at the GigaDB[25]. The entire dataset comprises genome assemblies, gene sets, a list of repeat elements, tRNAs, miRNAs, gene clusters, and the newly released bamboo genome, providing a reliable resource for many analyses, including genomic, genetic, and molecular biology experiments

Vast transcriptomic data generated using the Illumina and PacBio platforms

To facilitate the genome-wide investigation of the AS landscape in moso bamboo and comprehensively identify the factors that influence AS at the post-regulation level, we performed high-throughput RNA

sequencing (RNA-Seq) using the Illumina HiSeq-4000 platform. In total, 26 individual representative RNA samples were sequenced using 150 base paired ends (Additional Table S19 and Fig. S10-11). After preprocessing, we obtained an average of 90 million high-quality reads (~13.6 Gb) per sample, accounting for 92.78% of the raw reads. Approximately 80.57% of the high-quality reads were mapped to the reference genome at a unique position and designated unique reads (Additional Tables S20-21). According to the alignment distribution, most sequences were mapped in exonic regions. The exonic rate, which was defined as the fraction of reads mapping within exons, was on average 81.94%. The remaining reads were mapped in intronic regions (8.46%) and intergenic regions (9.6%) (Additional Table S22 and Fig. S12-13). An in-depth exonic coverage (~2,521× per sample) was detected (Additional Fig. S14). Therefore, the large-scale, in-depth high-quality transcriptomic data, together with a high-quality reference genome, contributed to an accurate AS identification in moso bamboo.

To accurately identify the full-length splice isoforms, we sequenced the bamboo transcriptome using the PacBio platform. RNA from a mixture of 26 samples was used based on full-length cDNA sequencing of alternatively spliced isoforms (Iso-Seq). According to the length distribution of the transcripts in all samples (Additional Table S24), we constructed 3 SMRTbell libraries (1-2 kb, 2-3 kb, and >3 kb) for the mixed sample and sequenced 9 cells, generating ~562 Mb of raw data and 214,372 reads-of-insert (ROIs), including 133,599 full-length ROIs (containing a 5' primer, 3' primer and a poly(A) tail); the remaining ROIs were non-full-length ROIs (Additional Table S24 and Fig. S15). The accuracy evaluation based on aligning the ROIs against the new genome showed that the per-nucleotide error was approximately 2.05% and consisted of mismatches (0.32%), insertion (0.98%) and deletions (0.75%).

Numerous genes underwent AS in moso bamboo

Based on the improved reference genome and large-scale transcriptome data, we performed a genome-wide analysis to identify AS in moso bamboo using the previous pipeline[10]. In total, 266,711 uniform AS events were identified in 25,225 AS genes, accounting for *ca.* 49.39% of all annotated genes. Except for the 12,653 AS genes identified in the gene annotation, the remaining (12,572) genes were considered novel AS genes (Additional Fig. S16).

The Iso-Seq data were also utilized to detect AS in an analysis parallel to the RNA-Seq analysis. In total, 4,246 AS events and 2,218 AS genes were identified (Fig. 2a, b). According to the PacBio-Illumina

overlapping analysis, which was performed to assess the validity of the AS prediction, 81.21% of the AS events and 97.34% of the AS genes identified in the Iso-Seq analysis completely overlapped with those in the RNA-Seq analysis. Among the four main AS types, on average, 80.37% of the AS events and 95.59% of the AS genes also overlapped (Additional Fig. S17). Thus, a higher accuracy is a strong indicator of the validity of the computationally predicted AS.

The AS gene number was strongly and positively correlated with the AS event number (correlation coefficient = 0.97, p-value <0.05) (Fig. 2c). The four main AS types were detected in the AS events in moso bamboo according to the canonical splicing patterns (GT-AG, GC-AG, and AT-AC splice sites). As shown in Fig. 2b, intron retention (IR, 38.22%) represented the most abundant type of AS event, followed by alternative 3′ splice site acceptor (A3SS, 20.20%) and alternative 5′ splice site acceptor (A5SS, 10.48%). Exon skipping (ES, 2.92%) was the least prevalent type among the four main AS types.

As the functional implication of AS genes, the enrichment analysis result showed 885 genes, which alternatively spliced in all samples, significantly enriched in RNA metabolic processing, mRNA processing, RNA processing and RNA splicing in the processes (Additional Table S26). Since AS possess strong specificity to different tissues or developmental stages, we identified 181,105 tissue-specific AS events (67.57%), which account for one-third of the AS events (termed as among-tissue). Then, the remaining two-third of the AS events were detected based on comparisons of the transcript isoforms within individual tissues (termed as within-tissue) (Additional Fig. S18).

The transposable element (TE) analysis showed 26,366 genes were detected to TE-insertion, accounted for 51.62% of all genes, and the total length of TE-insertion in genes was ~46 Mb. According to the different position of the TE-inserted intron, TE-introns mainly concentrated in the front and rear of a gene (Additional Fig. S19). Additionally, the usage and distribution of splice sites demonstrated GT-AG splice sites were the most abundant, corresponding to 97.31% of entire AS events, followed by GC-AG (2.33%) and GT-AT (0.32%) splice sites (Additional Fig. S20). Except for canonical splice site (GT-AG, GC-AG, and AT-AC), the remaining 2,406 splice sites were identified as non-canonical splice sites, contained 2,373 GT-AT splice sites and 33 splice sites of other types.

Evolutionary analysis of AS in moso bamboo

We obtained 8 datasets of orthologous genes representing different levels of conservation (Fig. 3a)

designated dataset8 (most conserved genes) to dataset1 (bamboo-specific genes) based on a phylogenetic relationship of 8 selected species (i.e., *Amborella trichopoda*, *A. thaliana*, *Elaeis guineensis*, *B. distachyon*, *O. sativa*, *Spirodela polyrhiza*, *S. bicolor* and *Ph. edulis*). AS was detected in all datasets, but the proportion of AS genes in each dataset gradually decreased from D8 to D1 ($p<0.05$). This trend was also observed in the two other datasets, i.e., all original datasets removing the overlapping genes and all original datasets removing duplicated genes. Therefore, the robust pattern, i.e., more conserved genes having more AS genes, should also exist in bamboo.

We investigated the distribution pattern of the four focal AS types in each dataset and found identical trends (IR>A3SS>A5SS>ES) (Fig. 3b), but the proportion of the AS types significantly differed. The proportion of IR in D8 was 60.80%, which was ~6-folds of that in D1 (11.88%). The ratio of the other AS types increased as the level of conservation decreased. In all datasets, the number of AS events gradually decreased from D8 to D1 (Fig. 3c). The most abundant AS events appeared in D8, and the least abundant AS events were detected in D1. Additionally, compared with the AS events among the genes expressed in samples with different specificities (maxTs) (for details, see Methods), the maxTs obviously increased from D8 to D1, representing an enhancement in the sample specificity from a highly conserved gene dataset to a poorly conserved dataset (Fig. 3c). Altogether, the conserved genes tended to have more AS genes, more AS events and less specificity.

We also examined the correlations among the gene length, CDS length, intron length, exon number, exon cassette length, and intron cassette length in all datasets (Additional Fig. S21). All genes in the different datasets were positively correlated with the gene length, CDS size, intron size, and exon number and negatively correlated with the exon cassette length and intron cassette length. Moreover, the distribution of the TE genes in the 8 datasets was examined. A substantially negative correlation was observed, indicating that the more conserved genes had more TE insertions.

Expansion of the gene family involved in the lignin biosynthesis pathway and implications for gene functional diversity

We systematically identified 13 gene families involved in the lignin biosynthesis pathway using the six genome sequences of *A. thaliana*, *B. distachyon*, *O. sativa*, *Ph. edulis*, *P. trichocarpa*, and *S. bicolor*. The expansion of most families was detected in bamboo (Additional Table S26). Each gene had multiple copies

in the bamboo genome, and the total size of the gene families in the lignin biosynthesis pathway was the largest in bamboo, with an average of ~19 copies per family. The most and least copy numbers were detected in the peroxidase gene family (*POD*, 77 genes) and *p*-coumarate 3-hydroxylase gene family (*C3H*, 3 genes), respectively.

Moreover, we performed an AS analysis of the genes in the lignin biosynthetic pathway. In total, 10 of the 13 families had AS genes accounting for more than half of the total, except for the ferulate 5-hydroxylase (F5H) gene family, which had a low proportion, and the CHS and caffeic acid *o*-methyltransferase (COMT) gene families, in which AS genes were not detected. A high abundance (>75%) of AS events was observed in the 4-coumarate: CoA ligase (4CL), hydroxycinnamoyl transferase (HCT) and cinnamyl alcohol dehydrogenase (CAD) gene families. In addition, we tested for positive selection in the gene families involved in the lignin biosynthetic pathway using a branch-site model. Several genes in two gene families, i.e., *HCT* and *CAD*, exhibited positive selection. The information provided by the phylogenetic relationship using the best model and log likelihood ratio (lnL) was provided in Additional Table S27.

Discussion

Current plant genomic studies are performed in a new era characterized by high-throughput genome sequencing and assembly generation using new technologies and more useful data. In 2013, our initial analysis of the *Ph. edulis* genome provided a genome-wide perspective of the structures of the genome and genes, the histories of the whole-genome duplication events, and the functional genes in critical functional categories[4]. In the present study, we enhanced both the precision and contiguity of the *Ph. edulis* genome and updated its annotation, accurately positioning the bamboo genome in an evolutionary landscape by performing comparative studies involving different species. Additionally, various biological characteristics of bamboo were studied in great detail using knowledge obtained from the latest version. Therefore, the chromosome-level reference genome and refined annotation paved the way for extra-genomic studies of bamboo and other related plants.

We provided the global AS landscape in bamboo based on a large amount of high-throughput data from RNA-Seq and Iso-Seq. These data enabled the accurate detection of transcripts with a low expression level and the acquisition of the complete gene structure, particularly in the AS analysis. A series of AS analyses

expanded our holistic understanding of AS in bamboo during post-transcriptional regulation, including the identification of AS genes and AS events, the distribution of the AS types, the use of a splice site, the length distribution of an alternative exon, etc. AS is considered a major mechanism responsible for multicellular diversity and the enhancement of the number of proteins from a limited repertoire of genes. For example, by combining one exon of four alternatively spliced regions that contain 12, 48, 33, and 2 alternative exons each, it was possible to generate, at most, 38,016 protein isoforms (12×48×33×2) from the *Dscam* gene in *Drosophila*[26]. In addition to the protein-coding genes, AS generates diverse transcripts of non-coding genes, indicating that the functional diversity of genes might be derived from AS. In bamboo, we identified 266,711 AS events and 25,225 genes in all samples, and on average, 15,971 AS events and 9,080 AS genes were detected in each sample. Thus, AS might be tissue specific, and the actual AS percentages in bamboo might be underestimated. More AS events, supported by transcripts with a low expression level, can be detected as the sequencing depth increases[27]. Additionally, the distribution of the AS type is consistent with that in *Arabidopsis*[5,9,27], soybean[10], and maize[13]. Nevertheless, a higher percentage of IR (38.22%) and other AS types (total 28.18%) were observed in bamboo. This higher percentage may be due to the unique features of bamboo and/or the depth of the sequencing, which will be addressed in a comprehensive comparative analysis using additional data in a future study. In addition, TEs constitute crucial gene regulatory elements and influence gene transcription and gene expression[28]. We did not detect a noticeable relationship between the TE genes and AS genes. Although previous reports have indicated that TE inserted within an intron interferes with the normal splicing pattern of pre-mRNA, provoking various forms of alternative splicing[29], our result implied that TE might be a driving force during the formation process of AS in bamboo. Furthermore, the identification of splice sites in an individual gene may provide an essential resource for fully understanding alternative splicing and isoform construction[30,31]. With respect to their distribution, the main AS types (i.e., GT-AG, GC-AG, and AT-AC) were consistent with those previously observed in animals and other plants[19].

More AS events were identified in the sample with vigorous growth, which is consistent with the previous studies[27,32]. However, according to our observations, the rhizome tissue had more AS events than the root tissue in moso bamboo, which may because the two tissues play differential roles during bamboo development. Photoassimilates were unavailable during the rapid growth of the moso bamboo shoots since no leaves were growing[33], and thus, the large amount of nutrients and energy in the shoot

mainly originate from the attached matured bamboos through underground rhizomes. Therefore, as a rhizomatous plant, the rhizome in moso bamboo plays a critical role in the transportation of nutrients and energy, which might explain the higher number of AS events detected in the rhizome. Moreover, to unveil the relationship between the incredible growth speed and AS in the shoots of moso bamboo, we selected shoots with 4 different heights and sampled 3 internodes (i.e., top, middle, and base) from each shoot according to the classification of shoot development. Obvious differences were observed in the AS event numbers in the final three shoot developmental stages, likely contributing to the fast growth during shoot development.

We performed an evolutionary analysis to unveil the relationship between AS and evolution using a comparative genome analysis. To date, the relationship between conservation and AS remains unknown. To tentatively address this issue, we performed a genome-wide analysis to examine AS in eight gene datasets with different degrees of conservation. The AS genes were more likely to be enriched in the highly conserved gene datasets, and these AS genes had more AS events. This finding was robust because we analyzed both the redundancy and copy number aspects. Previous reports have illuminated that duplication is a major source of functional diversity and the generation of new genes in plants[34], and new genes have generally low expression and suffer certain restrictions[35]. Altogether, we proposed that the relationship between conservation and AS may be associated with gene evolution and the generation of new genes. As a necessary substrate for the evolution of AS, new genes might first generate a single-functional gene without an AS event and then gradually form multifunctional and conserved genes with many AS events[22]. Conserved genes tend to be the hubs in gene-gene interaction networks, indicating their functional diversity, and during the gradual evolutionary process, newly generated genes are gradually added to this network and acquire pleiotropic roles[36]. Additionally, the four main AS types were abundant in the highly conserved gene datasets, and many other AS types appeared in the poorly conserved datasets. Thus, the four main AS types were conserved, and other types might represent an intermediate evolutionary stage. The distribution of the AS types depicted that IR occupied the dominant position, indicating that the importance of IR could be inferred from inspecting its prevalence throughout evolution in plants. The allocation in animals and yeast differs from that in plants. The most abundant AS event is ES, followed by AA and AD, while IR is the least common[37]. The discrepancies in the occurrence of the AS models between plants and animals suggest that differences exist between plants and animals in the genomic structure and mechanism of splice site

recognition[38].

Moreover, the conserved genes tended to be less specifically expressed and more likely to be hub genes interacting with many other genes. The mechanisms underlying the generation of a hub genes are unclear. According to our results, the conserved genes had more AS genes and events, which either produce functional alternative protein-coding transcripts with distinct functions in biological processes or modulate the functional spliced transcript level by producing certain non-coding transcripts[22]. However, since a large fraction of non-coding RNAs showed cell type-specific expression and was derived from evolutionary highly conserved promoter regions, non-coding RNAs likely represent a pool of sequences that can be recruited by evolution to regulate gene expression[39]. Therefore, highly conserved genes might be critical for evolution and function. We hypothesize that AS might play an important role in generating gene functional diversity, and this process might undergo rigorous regulation during long-term evolution since the non-conserved genes had less AS events than the conserved genes. Additionally, compared with the poorly conserved gene datasets, the more conserved AS gene dataset had a low tissue-specific expression profile, indicating the important fundamental roles of these genes, such as serving as hubs in gene-gene networks. We propose that functionally important genes are generated by more frequent AS events. As an essential biological process, AS plays a crucial role in acquiring more functions, which might explain why more conserved AS possesses more AS events. We hypothesize that this phenomenon likely applies not only to bamboo but also to other plants or even animals.

Furthermore, we observed the relationship between the AS genes in different conserved datasets and gene structure features. The AS genes in the highly conserved gene datasets possessed a longer gene length and more CDS, introns and exons and a shorter exon and intron cassette length. The architecture of the longer introns and shorter exons detected in the highly conserved gene dataset might be helpful in matching the constraints imposed by splicing recognition in the evolutionary process[21]. The exon-intron architecture in the different conserved genes might indicate that the splice-site choice and transcription by RNA polymerase II is changed during evolution[40,41]. During the evolutionary process, a new gene might be generated by duplication, which then forms less AS under strict constraints. Subsequently, functional AS is gradually generated and then evolves more functions largely through inducing changes in the gene structure, such as increasing the gene lengths, shorting the lengths of the exon cassette, inducing site mutations, etc.

Lignin represents a class of complex aromatic heteropolymers of monolignols that encrusts and interacts

with the cellulose/hemicellulose matrix of the secondary cell wall[42]. Lignin accounts for up to ~25% of the total dry weight in bamboo[2]. We performed a deep examination by combining AS and evolution analyses of the lignin biosynthesis pathway. The expansion of the gene families in the lignin biosynthesis pathway was detected in bamboo, and the two gene families (i.e., *HCT* and *CAD*) underwent more AS events and positive selection. HCT generates lignin by catalyzing *p*-coumaroyl CoA[43]. Then, *p*-coumaroyl CoA is also catalyzed by CHS to generate flavonoids. HCT and CHS compete with each other to bind *p*-coumaroyl CoA. In bamboo, the *HCT* family has more members and AS events than the *CHS* family, which might indicate its functional priority. CAD catalyzes many different substrates to generate different types of lignin. The aromatic lignin polymers commonly found in bamboo are composed of three monolignols, namely, *p*-hydroxyphenyl (H), vanillin (G), and syringaldehyde (S). Previous studies have shown the abundance of G and S lignin and a small amount of H lignin in bamboo[2]. The *CAD* family expansion in bamboo and positive selection may explain the different preferences of substrates to generate different proportions of monolignols in bamboo. The abundance of AS events, gene expansion, and positive selection were all consistent with the phenomenon that bamboo is remarkably adaptive to produce lignin.

Conclusions

To deeply explore the AS profile in the evolutionary landscape in bamboo, we improved reference genome and refined the annotation of moso bamboo. Based on the chromosome-level genome sequence and the abundant transcriptomic data from multiple tissues from six main bamboo producing areas in China, we provide a comprehensive AS perspective of moso bamboo by identifying 266,711 uniform AS events in 25,225 AS genes using both the Illumina and PacBio sequencing technology platforms. Moreover, the integrated analysis of the AS results in bamboo and comparative analysis among eight representative plant species exhibited that the more conserved genes tended to accumulate higher transcript levels and exhibit less specificity. Finally, by studying the lignin biosynthesis based on AS and evolution, we observed several characteristics of crucial genes related to lignin biosynthesis in bamboo, including gene family expansion, abundant AS and positive selection. In summary, these results will likely provide important resources for studies investigating bamboo's specificity as a woody plant in the grass family and exploring AS in the bamboo evolutionary landscape.

Method

Plant material collection

To obtain a comprehensive AS profile, the moso bamboo (*Phyllostachys edulis*) used in these experiments was collected from six main bamboo producing areas in China during the Spring of 2105, including (1) YiXing, JiangSu Province (N:31°15′08.41″, E:119°43′42.55″, 212 M), (2) TianMu Mountain, ZheJiang Province (N:30°19′13.42″, E:119°26′55.21″, 480 M), (3) XianNing, HuBei Province (N:29°81′10.02″, E:114°31′21.12″ 150 M), (4) TaoJiang, HuHan Province (N:28°28′39.74″, E:112°11′18.62″, 320 M), (5) GuiLin, GuangXi Province (N:28°28′39.74″, E:112°11′18.62″, 216 M) and (6) ChiShui, GuiZhou Province (N:28°28′15.27″, E:105°59′41.43″, 120 M). Twenty-six tissues were collected, including the rhizome, root, shoot, leaf, sheath, and bud, during different developmental stages. Each mixed sample was collected from the above six areas. Detailed information regarding the biological samples is provided in Additional Table S19.

Genome sequencing, assembly and annotation

We assembled the moso bamboo genome using WGS and Hi-C strategies and annotated the new genome sequence as described in a previous study[44] and Additional Files.

Hi-C library preparation, sequencing and assembling

The construction of Hi-C library was prepared as previously described[44] and the detailed descriptions were presented in Additional Files.

RNA isolation and Illumina RNA-Seq library construction

We used standard methods of RNA isolation, purity, concentration, reverse transcription, and cDNA library construction, as described in the previous study[45]. All cDNA libraries were constructed and normalized as described in the Additional File.

RNA-Seq using the Illumina platform

After passing quality control, the pooled libraries were optically examined using an Illumina Cluster Station and were then 150 base paired end sequenced on the Illumina HiSeq-4000 platform according to the

manufacturer's protocols. Finally, the quality of the reads was evaluated, and the low-quality reads were filtered using FastQC (version 0.11.3, http://www.bioinformatics.babraham.ac.uk/projects/fastqc/) with the default parameters. The statistics of the key metrics applied to the RNA-Seq data were calculated using RNA-SeQC (version1.1.8)[46] with the default parameter.

RNA-Seq data analysis

The adaptor sequences and low-quality sequences were trimmed using Trimmomatic (version 0.33)[47] during the preprocessing of the RNA-Seq data. Then, the cleaned data were mapped to the improved genome using HISAT (version 2.0.2)[48] with the following modifications from the default parameters: maximum intron length (4,000); specify strand-specific information (RF); and minimum score (L, -0.1, -0.1); report alignments tailored to transcript assemblers were allowed. The empirical transcripts in each sample were obtained using Cufflinks (version 2.2.1) [49] after the reads were aligned. The default parameters were used, except for the following parameters: the minimum isoform fraction (0.05); the small anchor fraction of the spliced reads (0.05); the minimum intron length (20); the maximum intron length (4,000); the library type (fr-firststrand); the corrected frag bias; and the corrected multi-read. ASTALAVISTA (version 4.0) [50,51] was used with the default parameters to identify the AS genes and events after the different assembled transcript isoforms were mapped to the corresponding gene model using Cuffcompare, which is a component of the Cufflink program. The main four types, *i.e.,* IR, A3SS, A5SS, and ES, were analyzed in the AS types. In addition, an enrichment analysis of the different genes was conducted using Ontologizer (version 2.0) [52] with the annotations from the Gene Ontology (GO) database (www.geneontology.org). We also calculated the sample specificity (Ts) values in each sample and each gene based on the expression level (FPKM values, the total number of fragments per kilobase of sequence per million reads mapped). A detailed description is provided in the previous report[53]. Briefly, Ts is defined as the fractional expression of a gene in one sample tissue relative to the sum of its expression in all samples. Thus, the maximum Ts value (maxTs) of a gene serves as an indicator of the sample specificity. Higher specificity values represent more tissue-specific expression[54].

Construction and sequencing of the Iso-Seq library

The construction of Iso-Seq library and sequencing were performed based on the PacBio manufacturer's protocol as previously described[55]. According to the length distribution of the transcripts predicted by

bioinformatics (Additional Table S22), three SMRTBell libraries (1-2 kb of 3 cells, 2-3 kb of 2 cells, and >3 kb of 4 cells) were size-selected and a total of 9 SMRT cells were sequenced on the PacBio platform.

Iso-Seq data analysis

The qualified sequencing data produced using PacBio RS II were processed to obtain consensus full-length isoforms. The isoforms from the multiple libraries were merged, and redundancy was removed to obtain the final consensus isoforms after processing the reads of the insert, classifying, and clustering. The assembled transcripts were mapped to the reference genome using PASA (version 2.0.2, http://pasapipeline.github.io/) with the default parameters. Then, similar to the short-read data, the output file of the gtf was analyzed using ASTALAVISTA with the default parameters to identify the AS.

Evolutionary analysis

We identified gene families, constructed a phylogenetic tree, predicted divergence times as a previously study[4] and the detailed information was provided in Additional Files.

Genome-wide identification of genes involved in the lignin biosynthetic pathway

The five genome sequences of *A. thaliana* (TAIR10), *B. distachyon* (v3.1), *O. sativa* (v7.0), *Populus trichocarpa* (JGI2.0.31), and *S. bicolor* (v3.1) were downloaded from the ENSEMBL database[56]. According to vast literature-based investigations, 140 genes from the lignin biosynthetic pathway was experimentally validated from previous studies (Additional Table S28), and then, these known genes were collected and used as the query sequences for further identification. We identified lignin biosynthetic genes using a BLAST search and domain analysis as described in the previous article[57]. Briefly, we performed BLASTN searches against the six genome sequences including moso bamboo using the coding sequence of the known genes with the following cut-off values: E-value $<1e^{-10}$; identity >95%; and coverage rate >40% query sequence. The filtered sequences were subsequently analyzed by hmmsearch using the Pfam-A.hmm database with an E-value $<1e^{-10}$. Consequently, unclear sequences with incomplete domains were discarded by manual correction. A phylogenetic tree was constructed using the same protocol described above.

Positive selection analysis

We performed a positive selection analysis based on the coding sequences of the lignin biosynthetic pathway. In each family, protein sequences were first aligned by PROBCONS (version 1.12)[58] using the default

parameters, except for the option of iterative refinement, for which we used 1,000 iterations. Then, we backed the alignment to its corresponding coding sequences. After obtaining the conserved blocks from the sequence alignment using Gblocks (version 0.91b)[59], jModelTest (verision 2.1.6)[60] was used to find the best model according to the Bayesian Information Criterion. Subsequently, PhyML (release 20141106)[61] was used to reconstruct the phylogenetic tree under the best model, with bootstrapping of 1,000 replicates. Finally, certain branches selected from the phylogenetic tree were examined in a positive selection analysis using PAML (version 4.8)[62] and a branch-site model.

Publicly available dataset

Short-read sequencing data from this whole-genome shotgun project can be deposited at European Molecular Biology Laboratory (EMBL) under the accession ERP001340. RNA-Seq raw sequence data for the 26 samples from this article were deposited in NCBI Short Read Archive database under the accession numbers: SRX2408703-28. Additionally, the chromosome-level genome and the latest annotation were provided in GigaDB.

Declarations

Author's Contribution

Experimental design: H.Z., Z.G., C.C., B.F. S.W., Z.C., H.Y. and Z.J. Experimental preformation: H.Z., H.T.; H.Z., L.C., Z.X., C.Z. and Y.W. Data analysis: W.Y., H.S., L.L., S.W., Y.Y., Y.L., Q.G., C.C., X.C and H.X. The providing of reagents, materials and analysis tools: H.Z. and Z.G. Article writing: H.Z., Z.C., Z.G. and B.F. All of the authors read and approved the final manuscript.

Competing interests

The authors declare that they have no competing interests.

Acknowledgements

This work received financial support from the Special Fund for Forest Scientific Research in the Public

Welfare from State Forestry Administration of China (No. 201504106), and the Sub-Project of National Science and Technology Support Plan of the Twelfth Five-Year in China (No. 2015BAD04B03 and No. 2015BAD04B01).

References

1. Zhao H, Zhao S, International Network for Bamboo and Rattan, Fei B, Liu H, Yang H, et al. Announcing the Genome Atlas of Bamboo and Rattan (GABR) project: promoting research in evolution and in economically and ecologically beneficial plants. GigaScience. 2017;6:1–7.

2. Bai Y-Y, Xiao L-P, Shi Z-J, Sun R-C. Structural variation of bamboo lignin before and after ethanol organosolv pretreatment. Int J Mol Sci. Multidisciplinary Digital Publishing Institute; 2013;14:21394–413.

3. Jiang Z. Bamboo and Rattan in the World. Beijing: China Forestry Publishing House.

4. Peng Z, Lu Y, Li L, Zhao Q, Feng Q, Gao Z, et al. The draft genome of the fast-growing non-timber forest species moso bamboo (Phyllostachys heterocycla). Nature Genetics. 2013;45:456–61.

5. Filichkin SA, Priest HD, Givan SA, Shen R, Bryant DW, Fox SE, et al. Genome-wide mapping of alternative splicing in Arabidopsis thaliana. Genome research. Cold Spring Harbor Lab; 2010;20:45–58.

6. Pan Q, Shai O, Lee LJ, Frey BJ, Blencowe BJ. Deep surveying of alternative splicing complexity in the human transcriptome by high-throughput sequencing. Nature Genetics. Nature Publishing Group; 2008;40:1413–5.

7. Wang ET, Sandberg R, Luo S, Khrebtukova I, Zhang L, Mayr C, et al. Alternative isoform regulation in human tissue transcriptomes. Nature. 2008;456:470–6.

8. Zhang PG, Huang SZ, Pin A-L, Adams KL. Extensive divergence in alternative splicing patterns after gene and genome duplication during the evolutionary history of Arabidopsis. Molecular Biology and Evolution. 2010;27:1686–97.

9. Marquez Y, Brown JWS, Simpson C, Barta A, Kalyna M. Transcriptome survey reveals increased complexity of the alternative splicing landscape in Arabidopsis. Genome research. Cold Spring Harbor Lab; 2012;22:1184–95.

10. Shen Y, Zhou Z, Wang Z, Li W, Fang C, Wu M, et al. Global dissection of alternative splicing in paleopolyploid soybean. Plant Cell. 2014;26:996–1008.

11. Mandadi KK, Scholthof K-BG. Genome-wide analysis of alternative splicing landscapes modulated during plant-virus interactions in Brachypodium distachyon. Plant Cell. 2015;27:71–85.

12. Li Q, Xiao G, Zhu Y-X. Single-nucleotide resolution mapping of the Gossypium raimondii transcriptome reveals a new mechanism for alternative splicing of introns. Mol Plant. 2014;7:829–40.

13. Thatcher SR, Zhou W, Leonard A, Wang B-B, Beatty M, Zastrow-Hayes G, et al. Genome-wide analysis of alternative splicing in Zea mays: landscape and genetic regulation. Plant Cell. 2014;26:3472–87.

14. Zhang G, Guo G, Hu X, Zhang Y, Li Q, Li R, et al. Deep RNA sequencing at single base-pair resolution reveals high complexity of the rice transcriptome. Genome research. 2010;20:646–54.

15. Rühl C, Stauffer E, Kahles A, Wagner G, Drechsel G, Rätsch G, et al. Polypyrimidine tract binding

protein homologs from Arabidopsis are key regulators of alternative splicing with implications in fundamental developmental processes. Plant Cell. 2012;24:4360–75.

16. Staiger D, Brown JWS. Alternative splicing at the intersection of biological timing, development, and stress responses. Plant Cell. 2013;25:3640–56.

17. Li W, Lin W-D, Ray P, Lan P, Schmidt W. Genome-wide detection of condition-sensitive alternative splicing in Arabidopsis roots. PLANT PHYSIOLOGY. American Society of Plant Biologists; 2013;162:1750–63.

18. Cui P, Zhang S, Ding F, Ali S, Xiong L. Dynamic regulation of genome-wide pre-mRNA splicing and stress tolerance by the Sm-like protein LSm5 in Arabidopsis. Genome Biology. BioMed Central; 2014;15:R1.

19. Reddy ASN. Alternative splicing of pre-messenger RNAs in plants in the genomic era. Annu Rev Plant Biol. 2007;58:267–94.

20. Barbosa-Morais NL, Irimia M, Pan Q, Xiong HY, Gueroussov S, Lee LJ, et al. The Evolutionary Landscape of Alternative Splicing in Vertebrate Species. Science. 2012;338:1587–93.

21. Keren H, Lev-Maor G, Ast G. Alternative splicing and evolution: diversification, exon definition and function. Nature Reviews Genetics. 2010;11:345–55.

22. Roy SW, Irimia M. Splicing in the eukaryotic ancestor: form, function and dysfunction. Trends Ecol. Evol. (Amst.). 2009;24:447–55.

23. Chen RY, Li XL, Song WQ, Liang GL, Zhang PX, Lin RS, et al. Chromosome atlas of major economic plants genome in China. Tomus 4. Chromosome atlas of various bamboo species[M]. Beijing: Science Press xxx, 646p.-illus.. ISBN 7030108353 Ch, En Chromosome numbers. Geog= 0 Systematics: ANGIOSPERMAE (GRAMINEAE)(KR, 200303867), 2003.

24. Peng Z, Lu T, Li L, Liu X, Gao Z, Hu T, et al. Genome-wide characterization of the biggest grass, bamboo, based on 10,608 putative full-length cDNA sequences. BMC plant biology. 2010;10:116.

25. Sneddon TP, Li P, Edmunds SC. GigaDB: announcing the GigaScience database. GigaScience. 2012;1:11.

26. Celotto AM, Graveley BR. Alternative splicing of the Drosophila Dscam pre-mRNA is both temporally and spatially regulated. Genetics. Genetics Society of America; 2001;159:599–608.

27. Wang B-B, Brendel V. Genomewide comparative analysis of alternative splicing in plants. Proceedings of the National Academy of Sciences. 2006;103:7175–80.

28. Slotkin RK, Martienssen R. Transposable elements and the epigenetic regulation of the genome. Nature Reviews Genetics. Nature Publishing Group; 2007;8:272–85.

29. Feschotte C. Transposable elements and the evolution of regulatory networks. Nature Reviews Genetics. Nature Publishing Group; 2008;9:397–405.

30. Li Y, Li-Byarlay H, Burns P, Borodovsky M, Robinson GE, Ma J. TrueSight: a new algorithm for splice junction detection using RNA-seq. Nucleic Acids Res. 2013;41:e51–1.

31. Nilsen TW, Graveley BR. Expansion of the eukaryotic proteome by alternative splicing. Nature. Nature Publishing Group; 2010;463:457–63.

32. Barbazuk WB, Fu Y, McGinnis KM. Genome-wide analyses of alternative splicing in plants: opportunities and challenges. Genome research. 2008;18:1381–92.

33. Song X, Peng C, Zhou G, Gu H, Li Q, Zhang C. Dynamic allocation and transfer of non-structural carbohydrates, a possible mechanism for the explosive growth of Moso bamboo (Phyllostachys heterocycla). Scientific Reports. 2016;6.

34. Flagel LE, Wendel JF. Gene duplication and evolutionary novelty in plants. New Phytol. Blackwell Publishing Ltd; 2009;183:557–64.

35. Lan X, Pritchard JK. Coregulation of tandem duplicate genes slows evolution of subfunctionalization in mammals. Science. 2016;352:1009–13.

36. Zhang W, Landback P, Gschwend AR, Shen B, Long M. New genes drive the evolution of gene interaction networks in the human and mouse genomes. Genome Biology. BioMed Central; 2015;16:202.

37. Kim E, Magen A, Ast G. Different levels of alternative splicing among eukaryotes. Nucleic Acids Res. 2007;35:125–31.

38. Nakai K, Sakamoto H. Construction of a novel database containing aberrant splicing mutations of mammalian genes. Gene. 1994;141:171–7.

39. Amaral PP, Dinger ME, Mercer TR, Mattick JS. The eukaryotic genome as an RNA machine. Science. 2008;319:1787–9.

40. Chen M, Manley JL. Mechanisms of alternative splicing regulation: insights from molecular and genomics approaches. Nat. Rev. Mol. Cell Biol. Nature Publishing Group; 2009;10:741–54.

41. Licatalosi DD, Darnell RB. RNA processing and its regulation: global insights into biological networks. Nature Reviews Genetics. Nature Publishing Group; 2010;11:75–87.

42. Martone PT, Estevez JM, Lu F, Ruel K, Denny MW, Somerville C, et al. Discovery of lignin in seaweed reveals convergent evolution of cell-wall architecture. Curr. Biol. 2009;19:169–75.

43. Li X, Bonawitz ND, Weng J-K, Chapple C. The growth reduction associated with repressed lignin biosynthesis in Arabidopsis thaliana is independent of flavonoids. Plant Cell. 2010;22:1620–32.

44. Dudchenko O, Batra SS, Omer AD, Nyquist SK, Hoeger M, Durand NC, et al. De novo assembly of the Aedes aegypti genome using Hi-C yields chromosome-length scaffolds. Science. 2017;356:92–5.

45. Zhao H, Sun H, Li L, Lou Y, Li R, Qi L, et al. Transcriptome-based investigation of cirrus development and identifying microsatellite markers in rattan (Daemonorops jenkinsiana). Scientific Reports. 2017;7:46107.

46. DeLuca DS, Levin JZ, Sivachenko A, Fennell T, Nazaire M-D, Williams C, et al. RNA-SeQC: RNA-seq metrics for quality control and process optimization. Bioinformatics. 2012;28:1530–2.

47. Bolger AM, Lohse M, Usadel B. Trimmomatic: a flexible trimmer for Illumina sequence data. Bioinformatics. 2014;30:2114–20.

48. Kim D, Langmead B, Salzberg SL. HISAT: a fast spliced aligner with low memory requirements. Nature Methods. Nature Publishing Group; 2015;12:357–60.

49. Trapnell C, Williams BA, Pertea G, Mortazavi A, Kwan G, van Baren MJ, et al. Transcript assembly and quantification by RNA-Seq reveals unannotated transcripts and isoform switching during cell differentiation. Nature Biotechnology. Nature Publishing Group; 2010;28:511–5.

50. Foissac S, Sammeth M. Analysis of alternative splicing events in custom gene datasets by AStalavista. Methods Mol. Biol. New York, NY: Springer New York; 2015;1269:379–92.

51. Foissac S, Sammeth M. ASTALAVISTA: dynamic and flexible analysis of alternative splicing events in custom gene datasets. Nucleic Acids Res. 2007;35:W297–9.

52. Bauer S, Grossmann S, Vingron M, Robinson PN. Ontologizer 2.0--a multifunctional tool for GO term enrichment analysis and data exploration. Bioinformatics. 2008;24:1650–1.

53. Marques AC, Tan J, Lee S, Kong L, Heger A, Ponting CP. Evidence for conserved post-transcriptional roles of unitary pseudogenes and for frequent bifunctionality of mRNAs. Genome Biology. BioMed Central; 2012;13:R102.

54. Winter EE, Goodstadt L, Ponting CP. Elevated rates of protein secretion, evolution, and disease among tissue-specific genes. Genome research. 2004;14:54–61.

55. Wang B, Tseng E, Regulski M, Clark TA, Hon T, Jiao Y, et al. Unveiling the complexity of the maize transcriptome by single-molecule long-read sequencing. Nature Communications. Nature Publishing Group; 2016;7:11708.

56. Kersey PJ, Allen JE, Allot A, Barba M, Boddu S, Bolt BJ, et al. Ensembl Genomes 2018: an integrated omics infrastructure for non-vertebrate species. Nucleic Acids Res. 2018;46:D802–8.

57. Fischer S, Brunk BP, Chen F, Gao X, Harb OS, Iodice JB, et al. Using OrthoMCL to assign proteins to OrthoMCL-DB groups or to cluster proteomes into new ortholog groups. Curr Protoc Bioinformatics. Hoboken, NJ, USA: John Wiley & Sons, Inc; 2011;Chapter 6:Unit6.12.1–19.

58. Roshan U. Multiple sequence alignment using Probcons and Probalign. Methods Mol. Biol. Totowa, NJ: Humana Press; 2014;1079:147–53.

59. Talavera G, Castresana J. Improvement of phylogenies after removing divergent and ambiguously aligned blocks from protein sequence alignments. Kjer K, Page R, Sullivan J, editors. Systematic Biology. 2007;56:564–77.

60. Darriba D, Taboada GL, Doallo R, Posada D. jModelTest 2: more models, new heuristics and parallel computing. Nature Methods. Nature Publishing Group; 2012;9:772–2.

61. Guindon S, Dufayard J-F, Lefort V, Anisimova M, Hordijk W, Gascuel O. New algorithms and methods to estimate maximum-likelihood phylogenies: assessing the performance of PhyML 3.0. Systematic Biology. 2010;59:307–21.

62. Yang Z. PAML 4: phylogenetic analysis by maximum likelihood. Molecular Biology and Evolution. 2007;24:1586–91.

Figure legends:

Figure 1. The comparative results based on two versions of the moso bamboo genome.

(A) The distribution of the contigs between two versions of the moso bamboo genome. The parameters, N50 and N90 of contigs, were masked. (B) The distribution of the scaffolds between two versions of the moso bamboo genome. The parameters, N50 and N90 of scaffolds, were masked. (C) The box plots were showed based on two versions of the moso bamboo genome, including gene length, intron length, CDS length, cDNA length, single exon length, and single intron length. (D) The BUSCO assessment result was provided, including the five assessment results (two genomes and three annotations). Two genomes contained the previous WGS version and the latest chromosome-level version. Annotation v1 was based on the version 1 of the moso bamboo genome. Annotation v2.1 and Annotation v2.2 were based on the version 2 and Annotation v2.2 was manually verified by Annotation v2.1.

Figure 2. The distribution of AS genes and events and their correlation

(A) The distribution of AS genes in bamboo, including four main types and Iso-Seq result. (B) The distribution of AS events in bamboo, including four main types and Iso-Seq result. (C) the correlation between AS genes and events was provided.

Figure 3. The evolutionary analysis in plants across bamboo

(A) The phylogenetic tree with divergence times was constructed, including *Amborella trichopoda*, *Elaeis guineensis*, *Arabidopsis thaliana*, *Brachypodium distachyon*, *Oryza sativa*, *Spirodela polyrhiza*, *Sorghum bicolor* and *Ph. edulis*. (B) a Venn of orthologous genes in related eight species was exhibited. (C) AS percentage and AS type were provided in D1 to D8 datasets, including redundant/non-redundant and multi-copy/single-copy. (D) increasing AS abundance and the decreasing specificity were displayed from D1 to D8.

Figure 4. The gene family expansion and AS abundance of bamboo in lignin biosynthetic pathway

A) A total of 13 families in lignin biosynthesis pathway were identified using six genomes of *A. thaliana*, *B. distachyon*, *O. sativa*, *Ph. edulis*, *P. trichocarpa*, and *S. bicolor*. Copy number and genes under positive selection were added. B) The structure, distribution and types of AS and related gene expression level were

exhibited in six gene families (4CL, C3H, CCR, HCT, LAC, and POD). The lignin biosynthetic enzymes are: PAL phenylalanine ammonia-lyase; TAL tyrosine ammonia-lyase; C4H cinnamate 4-hydroxylase; C3H 4-hydroxycinnamate 3-hydroxylase; COMT caffeic acid 3-O-methyltransferase; F5H ferulate 5-hydroxylase; 4CL 4-coumarate: CoA ligase; CCoA-3H coumaroyl-coenzyme A 3-hydroxylase; CCoA-OMT caffeoyl-coenzyme A O-methyltransferase; CCR cinnamoyl-CoA reductase; CAD cinnamyl alcohol, and HCT dehydrogenase hydroxycinnamoyl transferase.

Table 1. Statistics for the assembly of the moso genome using different sequence data

Statistics	WGS assembly		Hi-C assembly	
	Scaffold	Contig	Scaffold	Contig
Total number	19,285	76,900	19,684	84,758
Genome size (bp)	1,908,074,089	1,795,528,836	1,907,603,590	1,795,510,437
Gap number (bp)	112,545,253	0	112,093,153	0
Average length (bp)	98,940.84	23,348.88	96,911.38	21,183.96
N50 length (bp)	894,858	54,955	79,898,979	53,293
N90 length (bp)	115,487	11,757	44,603,463	10,445
Maximum length (bp)	5,406,526	738,589	137,299,170	738,589
Minimum length (bp)	926	157	318	1
GC content (%)	44.2	44.2	44.2	44.2

Figure 1 Click here to download Figure Figure1_ch_hic4.pdf

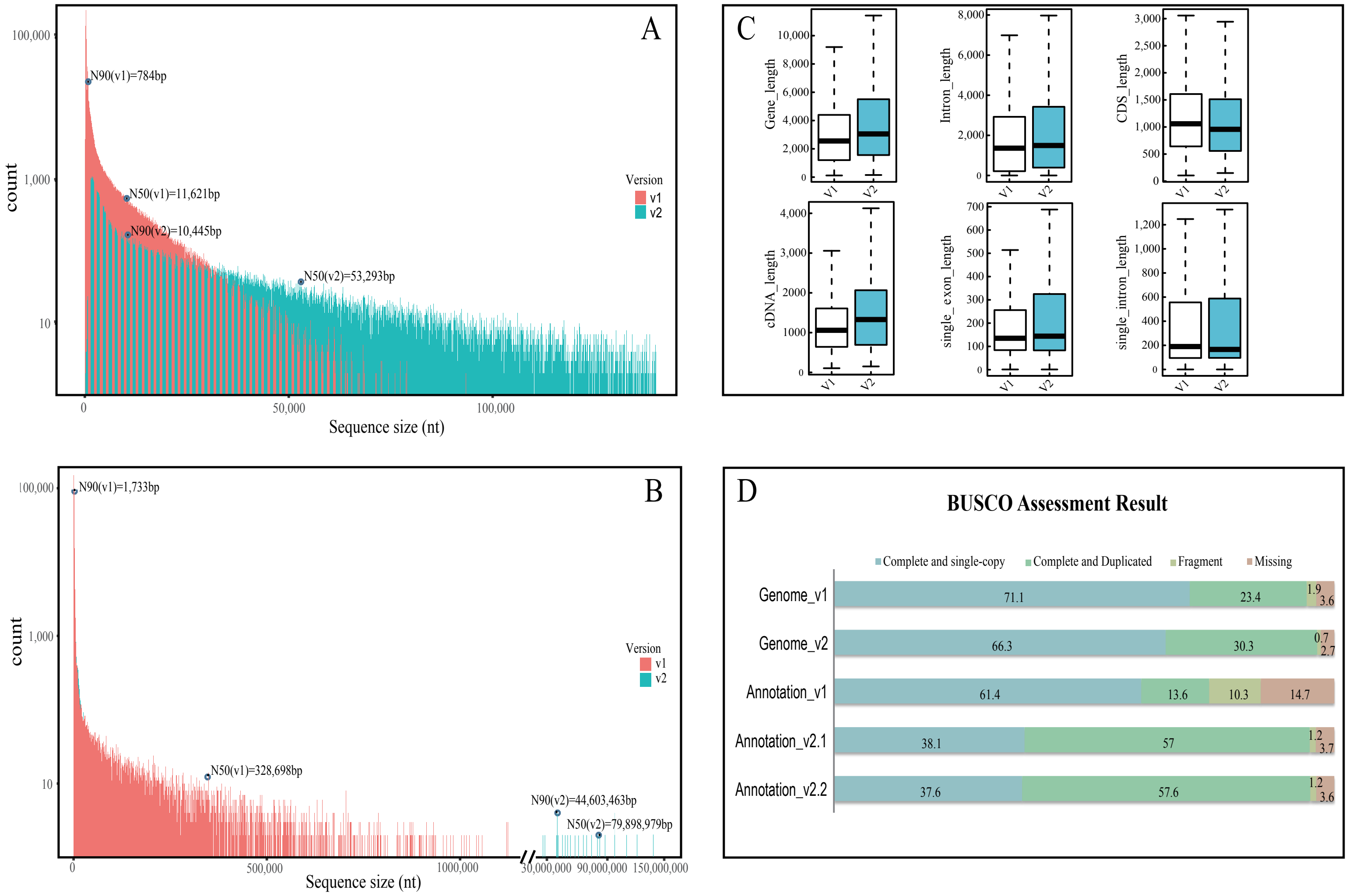

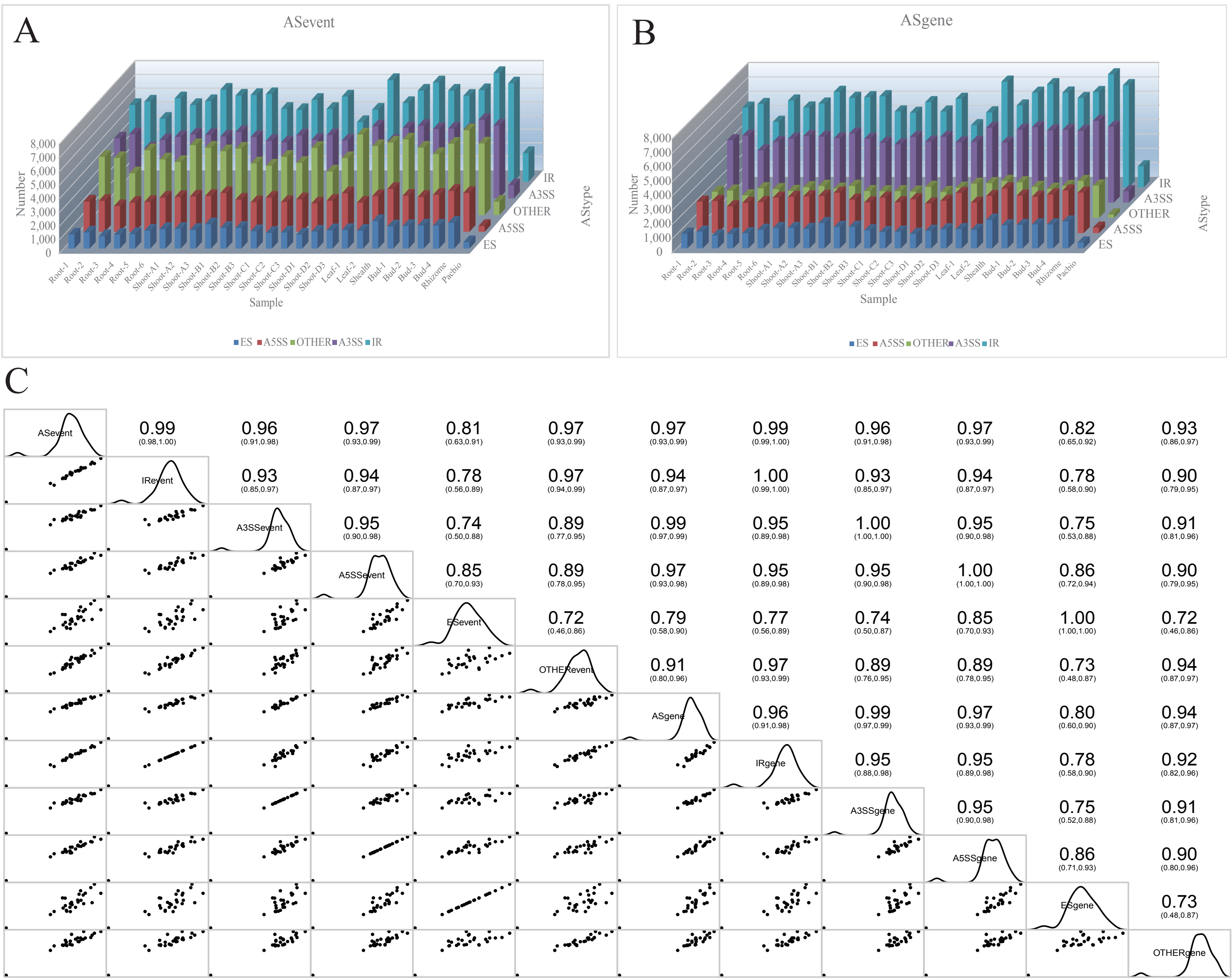
A
ASevent
Number
8,000
7,000
6,000
5,000
4,000
3,000
2,000
1,000
0
Root-1 Root-2 Root-3 Root-4 Root-5 Root-6 Shoot-A1 Shoot-A2 Shoot-A3 Shoot-B1 Shoot-B2 Shoot-B3 Shoot-C1 Shoot-C2 Shoot-C3 Shoot-D1 Shoot-D2 Shoot-D3 Leaf-1 Leaf-2 Sheath Bud-1 Bud-2 Bud-3 Bud-4 Rhizome Pachio
Sample
IR
A3SS
OTHER
A5SS
ES
AStype
ES A5SS OTHER A3SS IR
B
ASgene
Number
8,000
7,000
6,000
5,000
4,000
3,000
2,000
1,000
0
Root-1 Root-2 Root-3 Root-4 Root-5 Root-6 Shoot-A1 Shoot-A2 Shoot-A3 Shoot-B1 Shoot-B2 Shoot-B3 Shoot-C1 Shoot-C2 Shoot-C3 Shoot-D1 Shoot-D2 Shoot-D3 Leaf-1 Leaf-2 Sheath Bud-1 Bud-2 Bud-3 Bud-4 Rhizome Pachio
Sample
IR
A3SS
OTHER
A5SS
ES
AStype
ES A5SS OTHER A3SS IR
C
ASevent 0.99 (0.98,1.00) 0.96 (0.91,0.98) 0.97 (0.93,0.99) 0.81 (0.63,0.91) 0.97 (0.93,0.99) 0.97 (0.93,0.99) 0.99 (0.99,1.00) 0.96 (0.91,0.98) 0.97 (0.93,0.99) 0.82 (0.65,0.92) 0.93 (0.86,0.97)
IRevent 0.93 (0.85,0.97) 0.94 (0.87,0.97) 0.78 (0.56,0.89) 0.97 (0.94,0.99) 0.94 (0.87,0.97) 1.00 (0.99,1.00) 0.93 (0.85,0.97) 0.94 (0.87,0.97) 0.78 (0.58,0.90) 0.90 (0.79,0.95)
A3SSevent 0.95 (0.90,0.98) 0.74 (0.50,0.88) 0.89 (0.77,0.95) 0.99 (0.97,0.99) 0.95 (0.89,0.98) 1.00 (1.00,1.00) 0.95 (0.90,0.98) 0.75 (0.53,0.88) 0.91 (0.81,0.96)
A5SSevent 0.85 (0.70,0.93) 0.89 (0.78,0.95) 0.97 (0.93,0.98) 0.95 (0.89,0.98) 0.95 (0.90,0.98) 1.00 (1.00,1.00) 0.86 (0.72,0.94) 0.90 (0.79,0.95)
ESevent 0.72 (0.46,0.86) 0.79 (0.58,0.90) 0.77 (0.56,0.89) 0.74 (0.50,0.87) 0.85 (0.70,0.93) 1.00 (1.00,1.00) 0.72 (0.46,0.86)
OTHERevent 0.91 (0.80,0.96) 0.97 (0.93,0.99) 0.89 (0.76,0.95) 0.89 (0.78,0.95) 0.73 (0.48,0.87) 0.94 (0.87,0.97)
ASgene 0.96 (0.91,0.98) 0.99 (0.97,0.99) 0.97 (0.93,0.99) 0.80 (0.60,0.90) 0.94 (0.87,0.97)
IRgene 0.95 (0.88,0.98) 0.95 (0.89,0.98) 0.78 (0.58,0.90) 0.92 (0.82,0.96)
A3SSgene 0.95 (0.90,0.98) 0.75 (0.52,0.88) 0.91 (0.81,0.96)
A5SSgene 0.86 (0.71,0.93) 0.90 (0.80,0.96)
ESgene 0.73 (0.48,0.87)
OTHERgene

Figure 3

Click here to download Figure Figure3.pdf

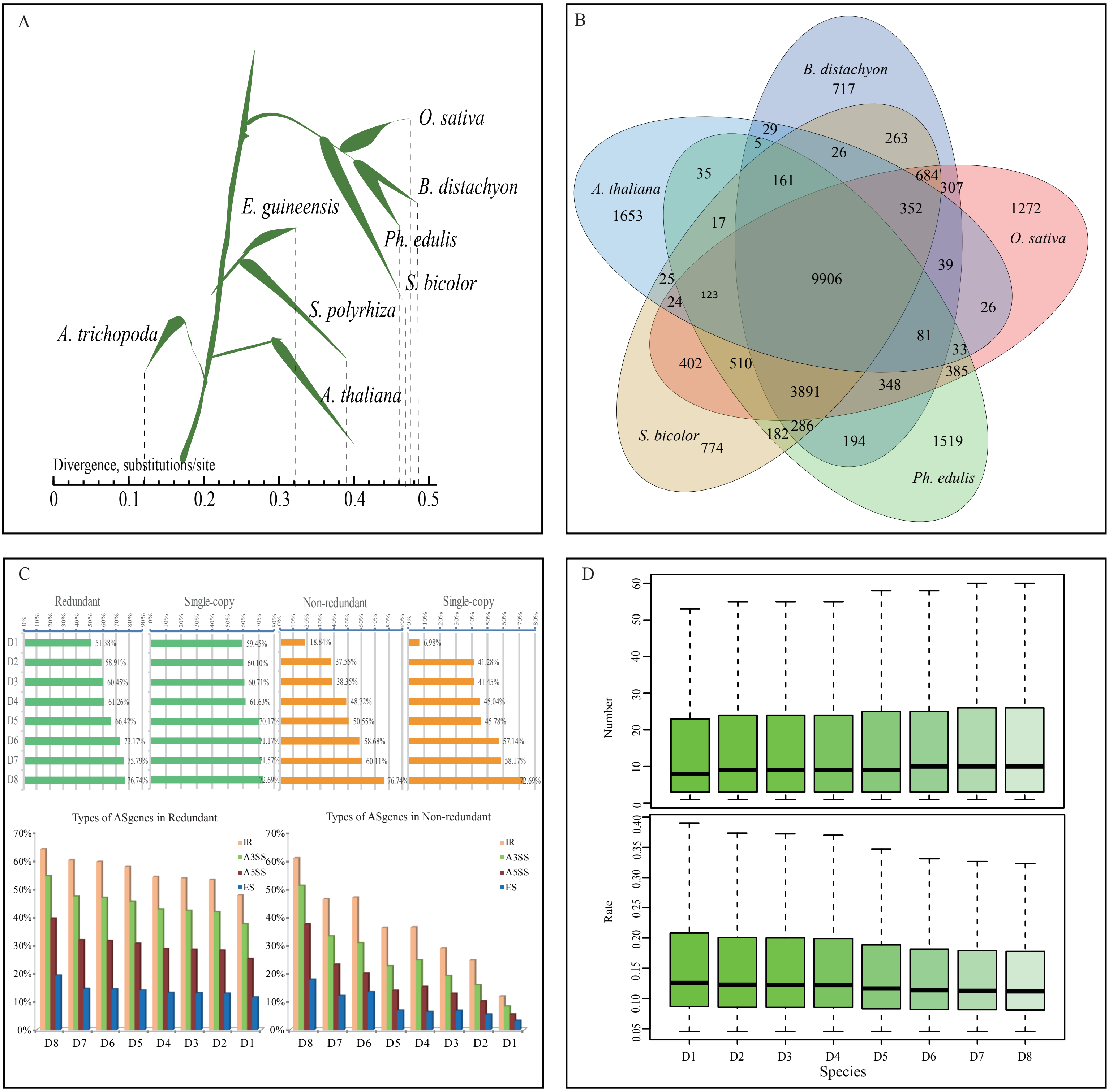

Figure 4

Click here to download Figure Figure4.pdf

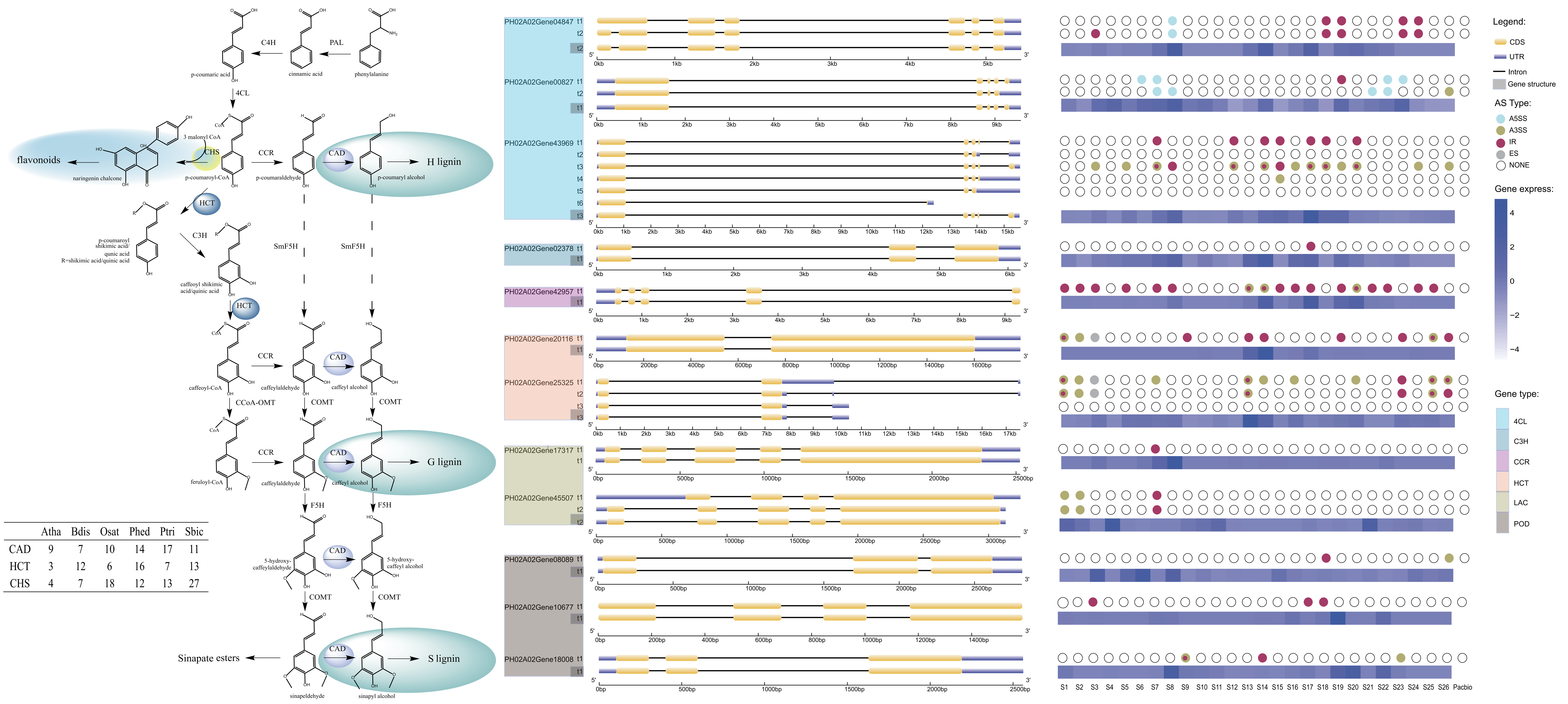

	Atha	Bdis	Osat	Phed	Ptri	Sbic
CAD	9	7	10	14	17	11
HCT	3	12	6	16	7	13
CHS	4	7	18	12	13	27

Additional File

Click here to access/download
Supplementary Material
Additional File2018.docx

Additional Table S25

Click here to access/download
Supplementary Material
Additional Table 25.xls

Additional Table S28

Click here to access/download
Supplementary Material
Additional Table 28.xls
