# Supplementary material for: Chromosome-level reference genome and alternative splicing atlas of moso bamboo (Phyllostachys edulis)
Source: Gigascience. 2018 Sep 8;7(10):giy115. doi: 10.1093/gigascience/giy115 (PMC6204424; doi:10.1093/gigascience/giy115)
Supplement: GIGA-D-18-00076_Revision_1.pdf [file giy115_giga-d-18-00076_revision_1.pdf]

GigaScience

Chromosome-level reference genome and alternative splicing atlas of moso bamboo (Phyllostachys edulis)

--Manuscript Draft--

**Manuscript Number:**	GIGA-D-18-00076R1
**Full Title:**	Chromosome-level reference genome and alternative splicing atlas of moso bamboo (Phyllostachys edulis)
**Article Type:**	Research
**Funding Information:**	Special Fund for Forest Scientific Research in the Public Welfare from State Forestry Administration of China (201504106) — Prof. Hansheng Zhao
**Abstract:**	Background<br>Bamboo is one of the most important non-timber forest products worldwide. However, a chromosome-level reference genome is lacking, and the evolutionary landscape of alternative splicing (AS) in bamboo remains unclear despite emerging data and improved technologies.<br>Results<br>Here, we provide a chromosome-level de novo genome assembly of the moso bamboo (Phyllostachys edulis) using additional abundance sequencing data and hybrid-combined de novo assembly strategies. The significantly improved genome is a scaffold N50 of 79.90 Mb, approximately 243 times longer than the previous version, and 51,074 high-quality protein-coding loci with intact structures were identified using single-molecule real-time sequencing and manual verification. Moreover, we provide a comprehensive AS profile based on the identification of 266,711 uniform AS events in 25,225 AS genes by large-scale transcriptomic sequencing of 26 representative bamboo tissues using both the Illumina and PacBio sequencing platforms. Via comparison with orthologous genes in related plants, we observed that the AS genes are concentrated in more conserved genes that tend to accumulate higher expressed transcripts and share less specificity. Furthermore, gene family expansion, abundant AS and positive selection were identified in crucial genes involved in lignin biosynthesis, indicating that moso bamboo is a woody plant in the grass family.<br>Conclusions<br>These fundamental studies provide useful information for future studies performing in-depth analyses of comparative genome and AS features. Additionally, our results highlight a global perspective of AS during evolution and diversification in bamboo.
**Corresponding Author:**	Hansheng Zhao<br>International Center for Bamboo and Rattan<br>Beijing, Beijing CHINA
**Corresponding Author Secondary Information:**	
**Corresponding Author's Institution:**	International Center for Bamboo and Rattan
**Corresponding Author's Secondary Institution:**	
**First Author:**	Hansheng Zhao
**First Author Secondary Information:**	
**Order of Authors:**	Hansheng Zhao
	Chunhai Chen
	Benhua Fei
	Songbo Wang
	Chengcheng Shi
	Jiongliang Wang

Powered by Editorial Manager® and ProduXion Manager® from Aries Systems Corporation

	XiaoChuan Liu
	Hailin Zhang
	Yongfeng Lou
	Lianfu Chen
	Huayu Sun
	Xianqiang Zhou
	Sining Wang
	Chi Zhang
	Hao Xu
	Lichao Li
	Yihong Yang
	Yanli Wei
	Wei Yang
	Qiang Gao
	Huanming Yang
	Zhimin Gao
	Shancen Zhao
	Zehui Jiang
**Order of Authors Secondary Information:**	
**Response to Reviewers:**	Scott Edmunds<br>Executive Editor<br>GigaScience<br><br>19 Apr 2018<br><br>Dear Dr. Scott,<br><br>Re: Manuscript reference No. GIGA-D-18-00076<br>Please find attached a revised version of our manuscript "Chromosome-level reference genome and alternative splicing atlas of moso bamboo (Phyllostachys edulis)", which we would like to resubmit for publication as a research article in GigaScience.<br><br>Your comments and those of the reviewers were highly insightful and enabled us to greatly improve the quality of our manuscript. In the following pages are our point-by-point responses to each of the comments and suggestions of the reviewers.<br><br>Revisions in the text are shown using red highlight. In accordance with the two reviewers' suggestions, we carefully revised our manuscript, including modified incorrected descriptions, putting the methods into Protocols.io (https://www.protocols.io/researchers/hansheng-zhao), and adding the analyses and extensive description of alternative splicing and evolution. We hope that these revisions in the manuscript and our accompanying responses will be sufficient to make our manuscript suitable for publication in GigaScience.<br><br>We shall look forward to hearing from you at your earliest convenience.<br><br><br>Yours sincerely,<br><br>Prof. Hansheng Zhao<br>Address: No. 8, Fu Tong Dong Da Jie, Chaoyang District, Beijing 100102, P.R. China<br>Tel: +86-010-8478 9804

Powered by Editorial Manager® and ProduXion Manager® from Aries Systems Corporation

Fax: +86-010-8478 9802
E-mail: zhaohansheng@icbr.ac.cn

Responses to the comments of Reviewer #1

The authors provide a high-quality genome assembly and gene annotation of moso bamboo in order to improve the first version published in 2013. Transcriptomic analysis was performed using several tissues to identify alternative splicing events and polymorphism within gene transcription by providing a repertoire of alternative transcription that could support tissue specialization. Additionally, an evolutionary insight, especially regarding the genes involved in lignin biosynthesis.

The genomic resource described in this manuscript will facilitate future studies on the evolution and functional genomic of moso bamboo and other grasses by providing a valuable information to the researchers interested in this area.

I recommend the manuscript for publication, following some minor revision, which I have listed below by manuscript page (p.) and line (L) numbers.

1. p. 3 - L20: Instead of "…have investigated in bamboo…" should be '…have been investigated in bamboo...'.

Response: Thank you very much for pointing out this error. We have revised the sentence, as follows:
"Only a limited number of genome-wide studies have been investigated in bamboo"

2. p. 4 - L27: Please add suppl. table reference for transcriptomic data.

Response: Thank you for this excellent suggestion. We have added additional table reference, as follows:
"Additionally, for the transcriptomic analysis, approximately 379 Gb and 5 Gb of raw data were produced from the Illumina and PacBio platforms, respectively (Additional Tables S2-7)"

3. p. 4 - L30: It was found a conflicting value "…We identified 266,711 uniform AS…". In the abstract section, the number of transcripts mentioned is 266,771. Please insert the correct value.

Response: Thank you very much for pointing out this error. The number, 266,711, is right number. We are sorry for the typo error and we have revised the sentence in the Abstract section, as follows:
"Moreover, we provide a comprehensive AS profile based on the identification of 266,711 uniform AS events in 25,225 AS genes by large-scale transcriptomic sequencing of 26 representative bamboo tissues using both the Illumina and PacBio sequencing platforms."

4. p. 4 L49: In the sentence "…we performed the genome assembly using different strategies to obtain a better genome assembly.", I suggest indicating the additional reference for detailed steps of the genome assembly.

Response: Thank you for this excellent suggestion. We have added related descriptions, as follows:
"Subsequently, we performed the genome assembly using different strategies to obtain a better genome assembly (see the Additional File for details)"

5. p. 5 L8: Instead of "…rice genome to find a mean coverage…" should be '…rice genome and we obtained a mean coverage…'.

Response: Thank you for this excellent suggestion. We have revised the sentence, as follows:
“Then we aligned the moso chromosomes to the rice genome and we obtained a mean coverage of ~59.77%”

6. p. 5 L13: About bamboo BAC sequences, I suggest mentioning that these sequences are derived from other bamboo specie (Ph. heterocycla) in this section or in the additional table S6.

Response: We appreciate this observation. In fact, the old Latin name, Ph. heterocycla, is a synonym of Ph. edulis and the both Latin names indicate the same bamboo (moso bamboo). Therefore, the BAC sequences mentioned in our manuscript are also derived from moso bamboo.

7. p. 5 L24: Provide correct reference - "…we predicted 51,074 high-quality protein-coding loci… (Additional Table S10)". Instead of Table S10, it should be Table S11.

Response: Thank you very much for pointing out this error. We have re-organized and re-numbered the Additional Table, as follows:
“we predicted 51,074 high-quality protein-coding loci with intact structures in moso bamboo (Additional Table S17)”

8. p. 5 L30: Provide correct reference - "… ~17% of the gene models were precisely refined (Additional Table S11)". Instead of Table S11, it should be Table S12.

Response: Thank you very much for pointing out this error. We have revised the table and re-organized and re-numbered the Additional Table, as follows:
“According to our results, ~17% of the gene models were precisely refined by the UTR addition and internal structural adjustment (Additional Table S19).”

9. p. 5 L36: Provide correct reference - Regarding the annotation using BUSCO, the reference should be Additional Table S13 instead of S12 in "(Fig 1d and Additional Table S12)".

Response: Thank you very much for pointing out this error. We have added the reference of BUSCO, and re-organized and re-numbered the Additional Table, as follows:
“According to the completeness assessment of the annotation using BUSCO [1], moso bamboo (95.2%) was more complete than Z. mays (92.2%) but close to O. sativa (95.6%) (Fig. 1d and Additional Table S20)”

10. p. 7 L27: The additional table reference for the enrichment analysis should be S25 instead of S26.

Response: Thank you very much for pointing out this error. We have re-organized and re-numbered the Additional Table, as follows:
“As the functional implication of AS genes, the enrichment analysis result showed 885 genes, which alternatively spliced in all samples, significantly enriched in RNA metabolic processing, mRNA processing, RNA processing and RNA splicing in the processes (Additional Table S25).”

11. p. 7 L32: "…which account for one-third of the AS events (termed as among-tissue)." According to additional Fig S18, the AS events classified as among-tissue correspond to two-third of the AS events.

Response: Thank you very much for pointing out this error. We are sorry for the typo error and we have revised the sentence,

Powered by Editorial Manager® and ProduXion Manager® from Aries Systems Corporation

as follows:
“Since AS possess strong specificity to different tissues or developmental stages, we identified 181,105 tissue-specific AS events (67.57%), which account for two-thirds of the AS events (termed as among-tissue).”

12. p. 8 L43-45: The sentence "the distribution of the TE genes in the 8 datasets was examined. A substantially negative correlation" could be '…was examined and a substantially negative…'

Response: Thank you for this excellent suggestion. We have revised this sentence, as follows:
“Moreover, the distribution of the TE genes in the 8 datasets was examined and a substantially negative correlation was observed, indicating that the more conserved genes had more TE insertions.”

13. p. 10 L28: "…a higher percentage of IR (38.22%) and other AS types (total 28.18%) were observed in bamboo." In order to avoid misunderstanding, I suggest clarifying that 'other AS types' represent a set of AS events except the main AS types already mentioned in the manuscript.

Response: Thank you for this excellent suggestion. We have added the related descriptions in the Analysis part, as follows:
“In subsequent analyses, we defined the four main AS types represented intron retention (IR), alternative 3’ splice site donor (A3SS), alternative 5’ splice site acceptor (A5SS), and exon skipping (ES), and we also defined the other AS types represented some AS types except the above four main AS types.”

14. p. 16 L50: Please, provide release number of the pfam-A.hmm database.

Response: Thank you for this excellent suggestion. We have added the related information, as follows:
“The filtered sequences were subsequently analyzed by hmmsearch using the Pfam-A.hmm database (released 2017/03/31).”

Figures and Tables
15. Figure 2: The figure legend should explain the meaning of the acronyms IR, A3SS, A5SS, and ES.

Response: We appreciate this observation. We have added the related description in the figure legend of Figure 2, as follows:
“IR, A3SS, A5SS, and ES represents intron retention, alternative 3’ splice site donor, alternative 5’ splice site acceptor, and exon skipping, respectively.”

16. Figure S3: In the figure legend "…The while boxes…" should be '…The white boxes…'

Response: Thank you very much for pointing out this error. We are sorry for the typo error and we have revised the figure legend of Figure S3, as follows:
“The white boxes in the BAC represent ambiguous bases (Ns) and the yellow line represent well aligned sequences between the BAC and the sequences.”

17. Fig. S17: Is the x-axis data label named 'AS' correct?

Response: Thank you very much for pointing out this error. We are sorry for the typo error. The second pillar in the X-axis should be ‘IR’ instead of ‘AS’. Therefore, we have revised the figure.

Powered by Editorial Manager® and ProduXion Manager® from Aries Systems Corporation

18. Table S1: Please provide the correct number of libraries in the 'Total' description.

Response: Thank you very much for pointing out this error. The total number should be 61 and we have revised the table

19. Table S3: Asterisk with description in the legend is not shown in the table.

Response: Thank you very much for pointing out this error. We have added asterisks in the Table S3

20. Table S28: I recommend excluding the words 'totally' and 'were' in the table legend.

Response: Thank you for this excellent suggestion. We have revised the table legend of Table S28, as follows:
"Additional Table S28. One hundred and forty genes of lignin biosynthesis pathway experimentally validated collected from public studies"

21. Some figures and tables citation are missing in the manuscript, such as Fig. 1a, 1b, and 3d; and additional table S23.

Response: Thank you for this excellent suggestion. We have added the figures and tables citation in the manuscript and reorganized tables citation in the Additional Files, as follows:
"Then, the Hi-C assembly was generated with total length reached 1.91 Gb as well as contig and scaffold N50 length with 53.29 Kb and 79.90 Mb based on the Hi-C data and the improved WGS assembly (Fig. 1a and 1b)."
"Additionally, compared with the AS events among the genes expressed in samples with different specificities (maxTs) (for details, see Methods), the maxTs obviously increased from D8 to D1, representing an enhancement in the sample specificity from a highly conserved gene dataset to a poorly conserved dataset (Fig. 3d)."
"Additionally, for the transcriptomic analysis, approximately 379 Gb and 5 Gb of raw data were produced from the Illumina and PacBio platforms, respectively (Additional Tables S2-7)"

Dataset
22. The PacBio reads (IsoSeq) must also be submitted to GiGADB or SRA and their accession number provided in the manuscript.

Response: We appreciate this observation. We have provided the SRA accession number (SRR7032261-69) for Iso-Seq data in the manuscript, as follows:
"RNA-Seq raw sequence data for the 26 samples and Iso-Seq raw sequence data for a mixture sample were deposited in NCBI Short Read Archive database under the accession numbers: SRX2408703-28 and SRR7032261-69, respectively."

Responses to the comments of Reviewer #2
Reviewer #2: Zhao et al reported a much improved genome assembly of moso bamboo, and characterized its alternative splicing (AS) atlas. While I find the assembly result very impressive, I have several questions on the methodology.
1. According to the method text in the "Additional File", the RNA reads were mapped onto the genome by "BLAT" and refined by HISAT. This is a rather unconventional way

Powered by Editorial Manager® and ProduXion Manager® from Aries Systems Corporation

to map RNA-seq data to a reference genome. Why not just use HISAT2? BLAT was designed to align transcripts, not individual RNA-seq reads. Also, I'm not aware of the adjustment function in HISAT. Please make sure the read mapping was done correctly because this is fundamental to the AS analysis.

Response: Thank you very much for pointing out this error. We are sorry for the unclear and confusion description of the RNA-Seq analyses in our Additional File. Indeed, as your mentioned, correctly mapping is fundamental for AS analyses, we double-checked our shell scripts and found the aligning RNA-Seq reads only used HISAT2 (release 2.0.4) rather than HISAT and BLAT. We have revised the sentence in the Additional File, as follows:
"Similarly, RNA-Seq data, a kind of high-throughput expressed data, were mapped to the genome to identify exon-intron splicing junctions and refine the alignment of RNA-Seq reads to the genome, using HISAT2 (version 2.0.4)[2]."

2. One important conclusion the authors made is that the conserved genes tend to have more AS events. It is however unclear to me how the authors measured the degrees of conservation. The authors did a gene family classification, and "obtained 8 datasets of orthologous genes representing different levels of conservation (Fig. 3a) designated dataset8 (more conserved genes) to dataset1 (bamboo-specific genes) based on a phylogenetic relationship of 8 selected species." But there was no further explanation. What does the "most conserved genes" in dataset8 entail? Based on presence or absence? And what is the difference between, say dataset8 and dataset7? How gene families were clustered was also unexplained (at least I couldn't find it). These are critical details that are missing.

Response: Thank you for this excellent suggestion. Based on the genome-wide identification of orthologous genes in the selected 8 plants (Amborella trichopoda, A. thaliana, Elaeis guineensis, B. distachyon, O. sativa, Spirodela polyrhiza, S. bicolor and Ph. edulis) and the species divergence time in a phylogeny tree (Fig. 3a), we identified eight orthologous gene datasets. For instance, dataset8 (D8) represents common orthologous genes in the selected 8 plants, which were located in an early divergence time in the phylogeny tree. D7 represents common orthologous genes in the selected 7 plants except A. trichopoda (the specie with the earliest divergence time) and D7 doesn't contain orthologues genes in D8, and so on. Thus, D1 represents bamboo-specific orthologous genes, which were located in later divergence time. According to a previous study [3], we obtained the divergence times of genes based on the presence and absence of orthologs in the phylogeny. In our subsequent study, thus, we considered the bamboo-specific genes (D1) as a poorly conserved gene dataset and the common genes in all selected plants (D8) as a highly conserved gene dataset, and the degree of conservation decreased monotonically from D8 to D1. Lastly, we have revised the related descriptions and Fig. 3a, as follows:
"Evolutionary analysis of AS in moso bamboo
Based on the genome-wide identification of orthologous genes in the selected 8 plants (Amborella trichopoda, A. thaliana, Elaeis guineensis, B. distachyon, O. sativa, Spirodela polyrhiza, S. bicolor and Ph. edulis) and the species divergence time in a phylogeny tree (Fig. 3a), we identified eight orthologous gene datasets. For instance, dataset8 (D8) represented common orthologous genes in the selected 8 plants, which were located in an early divergence time in our constructed phylogeny. D7 represented common orthologous genes in the selected 7 plants except A. trichopoda (the specie with the earliest divergence time) and D7 doesn't contain orthologues genes in D8. And so on. Thus, D1 represented bamboo-specific orthologous genes, which were located in later divergence time. According to a previous study[3], we obtained the divergence times of genes based on the presence and absence of orthologs in the phylogeny. In our subsequent study, thus, we considered the bamboo-specific genes (D1) as a poorly conserved gene dataset and the common genes in all selected plants (D8) as a highly conserved gene dataset, and the degree of conservation decreased monotonically from D8 to D1."

3. A species phylogeny was reconstructed from 8 genomes, but no information is provided about how this was done. What are the "single-copy orthologous genes"? What methods and programs you used for phylogenetic reconstruction?

Powered by Editorial Manager® and ProduXion Manager® from Aries Systems Corporation

Response: Thank you for this excellent suggestion. We have revised the related methods in the Additional File, as follows:
"S3.1 Orthologous Gene and Phylogenetic
The identification of orthologous gene clusters was considered as a fundamental aspect of genome evolution. Single-copy gene families and multi-gene families were identified by orthMCL (version 2.0.9) [4] among Ph. edulis and other 7 plant species, including Amborella trichopoda (version 1.0) from Amborella Genome Database (amborella.huck.psu.edu), Elaeis guineensis (GCF_000442705.1) from NCBI database, Arabidopsis thaliana (TAIR10), Brachypodium distachyon (version 3.1), Oryza sativa (version 7.0), Spirodela polyrhiza (version 2) and Sorghum bicolor (version 3.1) from the ENSEMBL database. The statistic of the gene family clustering in the 8 species was showed in Additional Table S24. The comparison of gene family clustering was provided in Additional Fig. S7. Afterwards, all single-copy genes were used to construct the phylogenetic tree by PhyML (version 3.0) [5] specifying a HKY85 substitution model with a gamma distribution across sites (Additional Fig. S8)."

4. Species divergence time was estimated, but again, the authors provided no methodological detail. How was the molecular clock estimated? Did you test the validity of assuming a molecular clock (e.g. relative rate test)? Further, the time calibrations listed in the Additional File need citations; they also to me look like secondary calibrations rather than "fossil time".

Response: Thank you for this excellent suggestion. We are sorry for the unclear and confusion description in the analysis of the species divergence time. Indeed, we estimated the species divergence time using calibration time rather than fossil time and we have revised the related Method in the Additional File, as follows:
"S3.3 Estimation of Divergence Time
In order to estimate the divergence time between Ph. edulis and the other 7 sequenced plant genomes, a Bayesian relaxed molecular clock approach was used to estimate the divergence time using MCMCTREE in PAML (version 4)[6]. Calibration times were gained from a previous study [7] (O. sativa vs. B. distachyon: 40-54 Mya; O. sativa vs. S. bicolor: 45-60 Mya; A. trichopoda vs. S. bicolor: 119.7-199.3 Mya)."

5.Though I appreciate the artistic value of Fig. 3A, it is scientifically incorrect (or at least very confusing). The x-axis is apparently in unit of substitution/site, which is a branch length measurement. It does not make sense to have a terminal tip linked (by vertical dashed line) to a branch length value. There was also no "divergence times" information in this figure and the legend should be revised.

Response: Thank you for this excellent suggestion. we have re-made the Fig.3a.

6. The expansion of lignin biosynthesis genes could be due to whole genome duplication (WGD), but WGD was not discussed. Are the two decoupled?

Response: Thank you for this excellent suggestion. According to the additional analysis of the divergence time of lignin biosynthesis genes, we have added an explanation about the expansion of lignin biosynthesis genes in the aspect of WGD in the Analysis and Discussion, respectively, as follows:
In the section of Analysis
"Additionally, we calculated the synonymous substitution rate analysis for 13 gene families evolved in the lignin biosynthesis using the yn00, which was a package in PAML to estimate synonymous and nonsynonymous substitution rates. Then, the Ks rate was translated to the divergence time by the formula T=Ks/2r (r=6.5×10-9). As shown in Additional Fig. S22, the result indicated that the divergence time of the lignin biosynthesis genes occurred at the 5~16 million year ago (Mya), which correspond to the whole genome duplication (WGD) time 7~12 Mya in the moso bamboo genome [8]."
In the section of Discussion
"Combined with the results of the divergence time of the lignin biosynthesis genes and our previous study [8], we estimated the occurrence of a putative WGD event at 7~12

Powered by Editorial Manager® and ProduXion Manager® from Aries Systems Corporation

Mya in the moso bamboo genome, suggesting that there might have been a tetraploidization event during bamboo history [8]. Then, the ancient tetraploid moso bamboo evolved into a current diploid moso bamboo. Additionally, WGD could provide more gene copies, which facilitated evolving the genes with new functions [9]. Therefore, the expansion of the lignin biosynthesis genes in moso bamboo could be due to the occurrence of WGD event."

Some other comments are listed below. The manuscript has no page number, and the line numbers does not match the actual lines and restart in each page, which make the review difficult. Anyway, I tried my best to point out where in the text I was referring to.
Abstract
7. Line 18 - what is "additional abundance data"? You meant sequencing data?

Response: We appreciate this observation. Indeed, the data means the sequencing data and we have revised the sentence in the Abstract, as follows:
"Here, we provide a chromosome-level de novo genome assembly of the moso bamboo (Phyllostachys edulis) using additional abundance sequencing data and hybrid-combined de novo assembly strategies."

8. Line 31 - "dramatic evolutionary characteristics" is too dramatic and unclear. Please be specific or take out this sentence.

Response: Thank you for this excellent suggestion. We have removed the sentence in the Abstract, as follows:
"Via comparison with orthologous genes in related plants, we observed that the AS genes are concentrated in more conserved genes that tend to accumulate higher expressed transcripts and share less specificity.

9. Line 39 - what does "bamboo's specificity in being a woody plant" mean? Please clarify "specificity".

Response: Thank you for this excellent suggestion. Our result indicated moso bamboo has the features of woody bamboo in the grass family based on the analysis of the lignin biosynthesis pathway. To properly express the meaning, we have revised the sentence in the Abstract, as follows:
"Furthermore, gene family expansion, abundant AS and positive selection were identified in crucial genes involved in lignin biosynthesis, indicating that moso bamboo is a woody plant in the grass family.

Background
10. Line 20 - change "investigated" to "been carried out".

Response: Thank you very much for pointing out this error. We have revised the sentence, as follows:
"Only a limited number of genome-wide studies have been investigated in bamboo."

11. Line 46 - change "is responsible" to "is partly responsible".

Response: Thank you for this excellent suggestion. We have revised the sentence, as follows:
"Species-specific AS is partly responsible for a wide variety of biodiversity with limited repertoires of protein coding genes"

12. Line 46 - take out "our colorful dynamic world full of".

Response: Thank you for this excellent suggestion. We have removed the part, as follows:
"Species-specific AS is partly responsible for a wide variety of biodiversity with limited

repertoires of protein coding genes.”

13. Line 6 "between conservation and AS" - What conservation? Sequence conservation? Gene functional conservation? Amino acid conservation? Protein structural conservation?

Response: Thank you for this excellent suggestion. We have revised the sentence, as follows:
“We performed a genome-wide investigation to determine the relationship between amino acid conservation and AS and examine the evolution of AS status of genes that are involved in the lignin biosynthesis.”

14. Line 6 - change "between evolution and the AS status of genes …" to "examine the evolution of AS status of genes …"

Response: Thank you for this excellent suggestion. We have revised the sentence, as follows:
“We performed a genome-wide investigation to determine the relationship between amino acid conservation and AS and examine the evolution of AS status of genes that are involved in the lignin biosynthesis.”

Data description
15. Line 21 - change "different strategies" to "different sequencing strategies".

Response: Thank you for this excellent suggestion. We have revised the sentence, as follows:
“For the assembly of the moso bamboo genome, approximately 603.3 Gb genome data with different sequencing strategies were generated.”

Analyses
16. Line 53 - "assembly" statistics.

Response: Thank you for this excellent suggestion. We have revised the sentence, as follows:
“Compared with those of our previous version[8], the assembly statistics and quality of the new WGS assembly were obviously improved (Additional Tables S9-10).”

17. Line 34 - change "was higher than" to "more complete than"

Response: Thank you for this excellent suggestion. We have revised the sentence, as follows:
“According to the completeness assessment of the annotation using BUSCO, moso bamboo (95.2%) was more complete than Z. mays (92.2%) but close to O. sativa (95.6%).”

18. Line 58 - what is "post-regulation level"? you meant post-translational level?

Response: Thank you for this excellent suggestion. We have revised the sentence, as follows:
“To facilitate the genome-wide investigation of the AS landscape in moso bamboo and comprehensively identify the factors that influence AS at the post-translational level, we performed high-throughput RNA sequencing (RNA-Seq) using the Illumina HiSeq-4000 platform.”

19. Line 25 - "RNA from a mixture of … " this sentence is unclear.

Response: Thank you for this excellent suggestion. We have revised the sentence, as

Powered by Editorial Manager® and ProduXion Manager® from Aries Systems Corporation

follows:
“The full-length cDNA sequencing of alternatively spliced isoforms (Iso-Seq) used RNA from a mixture of 26 samples.”

20. Line 48 - "…uniform AS events…" You meant "unique AS events"?

Response: We appreciate this observation. The number of the total AS events identified in our study were counted after removing repeated AS events in all 26 samples. Therefore, the word “unique” properly expressed the meaning and we have revised the sentence, as follows:
“In total, 266,711 unique AS events were identified in 25,225 AS genes, accounting for ca. 49.39% of all annotated genes.”

21. Line 4 - what are the four AS types? You need to introduce them first.

Response: Thank you for this excellent suggestion. We have added the introduction in Page 7, as follows:
“In subsequent analyses, we defined the four main AS types represented intron retention (IR), alternative 3’ splice site donor (A3SS), alternative 5’ splice site acceptor (A5SS), and exon skipping (ES) [10], and we also defined the other AS types represented some AS types except the above four main AS types.”

22. Line 6 - "A higher accuracy is a strong indicator of …" A higher accuracy of what?

Response: Thank you for this excellent suggestion. We have revised the sentence, as follows:
“Thus, a higher proportion of the PacBio-Illumina overlapping AS genes is a strong indicator of the validity of the computationally predicted AS”

23. Line 38 - change "were detected to TE-insertion" to "have TE insertion"

Response: Thank you for this excellent suggestion. We have revised the sentence, as follows:
“The transposable element (TE) analysis showed 26,366 genes have TE insertion, accounted for 51.62% of all genes, and the total length of TE-insertion in genes was ~46 Mb.”

24. Line 1 - Define D1-D8

Response: Thank you for this excellent suggestion. Based on the genome-wide identification of orthologous genes in the selected 8 plants (Amborella trichopoda, A. thaliana, Elaeis guineensis, B. distachyon, O. sativa, Spirodela polyrhiza, S. bicolor and Ph. edulis) and the species divergence time in a phylogeny tree (Fig. 3a), we identified eight orthologous gene datasets. For instance, dataset8 (D8) represents common orthologous genes in the selected 8 plants, which were located in an early divergence time in the phylogeny tree. D7 represents common orthologous genes in the selected 7 plants except A. trichopoda (the specie with the earliest divergence time) and D7 doesn’t contain orthologues genes in D8, and so on. Thus, D1 represents bamboo-specific orthologous genes, which were located in later divergence time. According to a previous study [3], we obtained the divergence times of genes based on the presence and absence of orthologs in the phylogeny. In our subsequent study, thus, we considered the bamboo-specific genes (D1) as a poorly conserved gene dataset and the common genes in all selected plants (D8) as a highly conserved gene dataset, and the degree of conservation decreased monotonically from D8 to D1.

25. Line 6 - which statistic test you used to derive this p value?

Response: Thank you for this excellent suggestion. We used Mann-Whitney U test for

P value and we have revised the sentence, as follows:
“AS was detected in all datasets, but the proportion of AS genes in each dataset gradually decreased from D8 to D1 (Mann-Whitney U test with $p<0.05$).”

26. Line 8 - what do the "original dataset", "overlapping genes", and "duplicated genes" mean here?

Response: Thank you for this excellent suggestion. We have revised the part, as follows:
“This trend was also observed in the two other datasets, i.e., removing common genes in more than two gene datasets in eight original datasets and using single-copy genes in eight original datasets. The eight-original dataset was derived from the genome-wide identification of orthologous genes in the selected 8 plants.”

27. Line 15 - change "abundance" to "percentage"

Response: Thank you for this excellent suggestion. We have revised the sentence, as follows:
“A high percentage (>75%) of AS events was observed in the 4-coumarate: CoA ligase (4CL), hydroxycinnamoyl transferase (HCT) and cinnamyl alcohol dehydrogenase (CAD) gene families.”

Discussion
28. Line 33 - please rephrase this sentence.

Response: Thank you for this excellent suggestion. We have revised the sentence, as follows:
“High-throughput genome sequencing and assembly strategy were broadly applied in current plant genomic studies with the development of new technologies and more useful data.”

29. Line 13 - "in addition to the protein-coding genes AS generates diverse transcripts of non-coding genes" Citation is needed here

Response: Thank you for this excellent suggestion. We have removed the sentence.

29. Line 35 - I do not follow the logic here. You found "no noticeable relationship between TE genes and AS genes", but you suggested that "TE might be a driving force during the formation process of AS in bamboo"?

Response: Thank you for this excellent suggestion. A previous study [11] shown TEs constitute crucial gene regulatory elements and influence gene transcription and gene expression. However, the noticeable relationship between TE genes and AS genes was unavailable in our study. Therefore, combined with our result and the previous study, we had implied that TE might be not a main reason of generating alternative splicing and might be a driving force during the formation process of AS in bamboo, although the mechanism of AS formation is still unknown.

30. Line 28 - what do you mean by "redundancy" here?

Response: Thank you for this excellent suggestion. The redundancy means some genes appeared in more than gene datasets and we have revised the sentence, as follows:
“This finding was robust based on we analyzed using the orthologous genes only in one dataset and using single-copy genes in selected species, respectively.”

31. Line 34 - take out "As a necessary substrate for the evolution of AS".

	Response: Thank you for this excellent suggestion. We have removed the part, as follows:<br>“New genes might first generate a single-functional gene without an AS event and then gradually form multifunctional and conserved genes with many AS events [12]”<br><br>32. Line 47 - what are the "many other AS types"?<br><br>Response: We appreciate this observation. Many AS types represented some AS types except the main four AS types and we have added the introduction in Page 7, as follows:<br>“In subsequent analyses, we defined the four main AS types represented intron retention (IR), alternative 3’ splice site donor (A3SS), alternative 5’ splice site acceptor (A5SS), and exon skipping (ES) [10], and we also defined the other AS types represented some AS types except the above four main AS types.”<br><br>33. Line 49 - there is no way you could infer the "intermediate evolutionary stage". Plus I couldn't figure out what are the other AS types.<br><br>Response: We appreciate this observation. We have added the introduction to the other AS types in Page 7 (see the above answer for details) and revised the sentence, as follows:<br>“Thus, the four main AS types were conserved, and other types might represent an intermediate stage”<br><br>34. Line 2 - line 36 please revise this paragraph. I could not follow the logic nor find the main point.<br><br>Response: Thank you for this excellent suggestion. We have greatly revised the paragraph, as follows:<br>“According to our results, the highly conserved gene datasets had more AS genes and events, which either produce functional alternative protein-coding transcripts with distinct functions in biological processes or modulate the functional spliced transcript level by producing certain non-coding transcripts [12]. We hypothesize that the highly conserved genes with more AS events might be critical for evolution and function in generating gene functional diversity and the generation process of the highly conserved genes might undergo rigorous regulation during long-term evolution since the poorly conserved genes had less AS events than the highly conserved genes. Additionally, compared with the poorly conserved gene datasets, the highly conserved AS gene datasets had a low tissue-specific expression profile, indicating these genes might be core genes in fundamental functions, such as serving as hubs in gene-gene networks. Therefore, we proposed that functionally important genes are generated by more frequent AS events. As an essential biological process, AS plays a crucial role in acquiring more functions, which might explain why the highly conserved AS possesses more AS events. We also hypothesize that this phenomenon likely applies not only to bamboo but also to other plants or even animals.”<br><br>35. Line 10 - why having more AS events would have "functional priority"?<br>Response: We appreciate this observation. We have removed the confused description and revised the related description, as follows:<br>“In bamboo, the HCT family has more members and AS events than the CHS family, which indicate that the HCT family might be in a dominant position in the competition to bind p-coumaroyl CoA.”<br><br>36. Line 41 - "uniform" you meant "unique"?<br><br>Response: Thank you for this excellent suggestion. We have revised the sentence, as follows:<br>“Based on the chromosome-level genome sequence and the abundant transcriptomic data from multiple tissues from six main bamboo producing areas in China, we provide

	a comprehensive AS perspective of moso bamboo by identifying 266,711 unique AS events in 25,225 AS genes using both the Illumina and PacBio sequencing technology platforms."<br><br>References:<br>1. Simão FA, Waterhouse RM, Ioannidis P, Kriventseva EV, Zdobnov EM. BUSCO: assessing genome assembly and annotation completeness with single-copy orthologs. Bioinformatics. 2015;31:3210–2.<br>2. Kim D, Langmead B, Salzberg SL. HISAT: a fast spliced aligner with low memory requirements. Nature Methods. 2015;12:357–60.<br>3. Zhang YE, Vibranovski MD, Landback P, Marais GAB, Long M. Chromosomal redistribution of male-biased genes in mammalian evolution with two bursts of gene gain on the X chromosome. Barton NH, editor. PLoS Biology. 2010;8:e1000494.<br>4. Chen F, Mackey AJ, Stoeckert CJ, Roos DS. OrthoMCL-DB: querying a comprehensive multi-species collection of ortholog groups. Nucleic Acids Research. 2006;34:D363–8.<br>5. Guindon S, Dufayard J-F, Lefort V, Anisimova M, Hordijk W, Gascuel O. New algorithms and methods to estimate maximum-likelihood phylogenies: assessing the performance of PhyML 3.0. Systematic Biology. 2010;59:307–21.<br>6. Yang Z. PAML 4: phylogenetic analysis by maximum likelihood. Molecular Biology and Evolution. 2007;24:1586–91.<br>7. International Brachypodium Initiative. Genome sequencing and analysis of the model grass Brachypodium distachyon. Nature. 2010;463:763–8.<br>8. Peng Z, Lu Y, Li L, Zhao Q, Feng Q, Gao Z, et al. The draft genome of the fast-growing non-timber forest species moso bamboo (Phyllostachys heterocycla). Nature Genetics. 2013;45:456–61.<br>9. Taylor JS, Raes J. Duplication and divergence: the evolution of new genes and old ideas. Annual Review of Genetics. 2004;38:615–43.<br>10. Barbosa-Morais NL, Irimia M, Pan Q, Xiong HY, Gueroussov S, Lee LJ, et al. The Evolutionary Landscape of Alternative Splicing in Vertebrate Species. Science. 2012;338:1587–93.<br>11. Slotkin RK, Martienssen R. Transposable elements and the epigenetic regulation of the genome. Nature Reviews Genetics. 2007;8:272–85.<br>12. Roy SW, Irimia M. Splicing in the eukaryotic ancestor: form, function and dysfunction. Trends in Ecology and Evolution. 2009;24:447–55.
**Additional Information:**	
**Question**	**Response**
Are you submitting this manuscript to a special series or article collection?	No
**Experimental design and statistics**<br><br>Full details of the experimental design and statistical methods used should be given in the Methods section, as detailed in our Minimum Standards Reporting Checklist. Information essential to interpreting the data presented should be made available in the figure legends.<br><br>Have you included all the information requested in your manuscript?	Yes
**Resources**	Yes

Powered by Editorial Manager® and ProduXion Manager® from Aries Systems Corporation

A description of all resources used, including antibodies, cell lines, animals and software tools, with enough information to allow them to be uniquely identified, should be included in the Methods section. Authors are strongly encouraged to cite Research Resource Identifiers (RRIDs) for antibodies, model organisms and tools, where possible.<br><br>Have you included the information requested as detailed in our Minimum Standards Reporting Checklist?	
**Availability of data and materials**<br><br>All datasets and code on which the conclusions of the paper rely must be either included in your submission or deposited in publicly available repositories (where available and ethically appropriate), referencing such data using a unique identifier in the references and in the "Availability of Data and Materials" section of your manuscript.<br><br>Have you have met the above requirement as detailed in our Minimum Standards Reporting Checklist?	Yes

Revision Click here to download Manuscript Manuscript-Moso-Revised1-419.docx

Chromosome-level reference genome and alternative splicing atlas of moso bamboo (*Phyllostachys edulis*)

Hansheng Zhao[1#], Chunhai Chen[2#], Benhua Fei[1#], Songbo Wang[2#], Chengcheng Shi[3], Jiongliang Wang[1], Xiaochuan Liu[3], Hailin Zhang[2], Yongfeng Lou[1], LianFu Chen[1], Huayu Sun[1], Xianqiang Zhou[2], Sining Wang[1], Chi Zhang[2], Hao Xu[1], Lichao Li[1], Yihong Yang[1], Yanli Wei[2], Wei Yang[2], Qiang Gao[2], Huanming Yang[2], Zhimin Gao[1+], Shancen Zhao[2+] and Zehui Jiang[1+]

[1] State Forestry Administration Key Open Laboratory on the Science and Technology of Bamboo and Rattan, Institute of Gene Science for Bamboo and Rattan Resources, International Center for Bamboo and Rattan, Futongdong Rd, WangJing, Chaoyang District Beijing 100102, China;

[2] BGI Genomics, BGI-Shenzhen, Building NO.7, BGI Park, No. 21 Hongan 3rd Street, Yantian District, Shenzhen 518083, China;

[3] BGI-Qingdao, No. 2877, Tuanjie Road, Sino-German Ecopark, Qingdao, Shandong Province, 266555, China;

[+] Co-corresponding author: gaozhimin@icbr.ac.cn, zhaoshancen@genomics.cn, and jiangzehui@icbr.ac.cn

[#] These authors contributed equally to this work.

Abstract

Background

Bamboo is one of the most important non-timber forest products worldwide. However, a chromosome-level reference genome is lacking, and the evolutionary landscape of alternative splicing (AS) in bamboo remains unclear despite emerging data and improved technologies.

Results

Here, we provide a chromosome-level *de novo* genome assembly of the moso bamboo (*Phyllostachys edulis*) using additional abundance sequencing data and hybrid-combined *de novo* assembly strategies. The significantly improved genome is a scaffold N50 of 79.90 Mb, approximately 243 times longer than the previous version, and 51,074 high-quality protein-coding loci with intact structures were identified using single-molecule real-time sequencing and manual verification. Moreover, we provide a comprehensive AS profile based on the identification of 266,711 uniform AS events in 25,225 AS genes by large-scale transcriptomic sequencing of 26 representative bamboo tissues using both the Illumina and PacBio sequencing platforms. Via comparison with orthologous genes in related plants, we observed that the AS genes are concentrated in more conserved genes that tend to accumulate higher expressed transcripts and share less specificity. Furthermore, gene family expansion, abundant AS and positive selection were identified in crucial genes involved in lignin biosynthesis, indicating that moso bamboo is a woody plant in the grass family.

Conclusions

These fundamental studies provide useful information for future studies performing in-depth analyses of comparative genome and AS features. Additionally, our results highlight a global perspective of AS during evolution and diversification in bamboo.

Keywords: Moso Bamboo, Genome, Annotation, Alternative Splicing, Transcriptome, Evolution

Background

Bamboo (Bambusoideae) is a fast-growing plant with substantial potential for generating income, restoring degraded landscapes and combating climate change in numerous Asian and African countries. Approximately 2.5 billion people economically depend on bamboo, reaching an annual international trade of over 2.5 billion US dollars [1]. Bamboo is a perennial grass in temperate and tropical forests worldwide. Its cellulose and hemicelluloses content is comparable to that of woody trees [2]. Moso bamboo (*Phyllostachys edulis*) accounts for ~73.76% of the bamboo growing region in China (4.43 million ha), constitutes the most abundant natural resource of non-wood products and plays significant roles in economics, ecology, culture, aesthetics and technology [3].

Only a limited number of genome-wide studies have been investigated in bamboo. We first reported a draft genome of moso bamboo in 2013 and released 2.05 Gb of the draft genome with 328 Kb of Scaffold N50 and 31,987 predicted genes [4]. Due to the development of sequencing technology and analytical methods, a chromosome-level reference genome with improved precision and contiguity could facilitate functional and evolutionary analyses of bamboo.

Alternative splicing (AS) is a major mechanism underlying the increased complexity and diversity of proteins made from a limited number of genes in eukaryotes [5]. More than 95% of human multi-exon genes have been predicted to express multiple splice isoforms [6,7], and the occurrence of AS events in plants is reported to be ~61%, ~52%, ~42%, ~40%, ~40% and 33% in *Arabidopsis thaliana* [8,9], *Glycine max* [10], *Brachypodium distachyon* [11], *Gossypium raiimondi* [12], *Zea mays* [13] and *Oryza sativa* [14], respectively. The different splicing products of a single gene represent major sources of functional plasticity and supposedly play important roles in plant growth, development, defense responses, signal transduction and flowering time [15-19]. Species-specific AS is partly responsible for a wide variety of biodiversity with limited repertoires of protein coding genes [20-22]. However, the mechanism by which AS affects some changes in the regulation of the gradual evolutionary process in plants based on genome-wide and deeply transcriptomic analyses is unclear. Moreover, the AS characteristics of genes with a diverse conservative status remains elusive.

In this study, we substantially improved the moso bamboo genome assembly and gene annotation. Based on the improved genome reference, we performed a comprehensive genome-wide analysis to uncover the

AS landscapes in bamboo using transcriptome data from 26 mixed samples collected from six main bamboo producing areas in China. These transcriptome data were generated using the Illumina and PacBio platforms. Numerous AS genes and events were detected, and various types of AS events were identified. We performed a genome-wide investigation to determine the relationship between amino acid conservation and AS and examine the evolution of AS status of genes that are involved in the lignin biosynthesis. In conclusion, our analysis not only provides a global profile of AS in bamboo for further experimental studies investigating the functions of genes and regulatory networks but also reveals the roles of AS in the evolutionary landscape.

Data description

For the assembly of the moso bamboo genome, approximately 603.3 Gb genome data with different sequencing strategies were generated. The WGS assembly was performed using ~154 Gb of newly acquired and ~220 Gb of previously acquired clean data [4]. The Hi-C assembly was using ~157 Gb raw data from Hi-C library and 17.58 Gb valid reads were obtained after quality control (Additional Table S1). Additionally, for the transcriptomic analysis, approximately 379 Gb and 5 Gb of raw data were produced from the Illumina and PacBio platforms, respectively (Additional Tables S2-7). Thus, we identified 266,711 unique AS events in 25,225 AS genes in moso bamboo according to the chromosome-level genome reference and the high-throughput transcriptome data.

Analyses

Chromosome-level genome assembly and gene annotation in moso bamboo

In order to enhance the quality of the moso bamboo genome, a total of 61 libraries were used and subjected to sequencing according to the instructions of the sequencer manufacturer (Additional Table S1). In total, we obtained ~ 603.3 Gb genome data with read length ranging from 76 bp to 250 bp. Subsequently, we performed the genome assembly using different strategies to obtain a better genome assembly (see the Additional File for details). First, the WGS assembly reached 1.91 Gb with a contig and scaffold N50 length of 55 Kb and 894 Kb, respectively (Additional Table S8). Compared with those of our previous version [4], the assembly statistics and quality of the new WGS assembly were obviously improved (Additional Tables S9-10). For example, the length of scaffold N50 and contig N50 were increased by 172% and 358%, respectively, and the 'N' base rate was decreased by 43%. Then, the Hi-C assembly was generated with total

length reached 1.91 Gb as well as contig and scaffold N50 length with 53.29 Kb and 79.90 Mb based on the Hi-C data and the improved WGS assembly (Fig. 1a and 1b). About 93.17% scaffolds from the WGS assembly were anchored onto 24 chromosomes (Additional Table S10) [23] and the scaffold N50 was increased by ~89-folds (Table 1). According to the contact map (Additional Fig. S1) and the assembly results, the boundaries between 24 chromosomes were observed clearly. Then we aligned the moso chromosomes to the rice genome and we obtained find a mean coverage of ~59.77% (Additional Fig. S2 and Additional Table S11). Additionally, we evaluated the chromosome-level assembly using bamboo-derived BAC sequences, full-length cDNAs [24] and some known genes (Additional Fig. S3 and Additional Tables S12-14). The chromosome-level assembly had a more extensive genome coverage, and the accuracy was higher than that of the first assembly.

The chromosome-level assembly generated could facilitate gene prediction in subsequent analyses after annotating repetitive sequences (Additional Table S15). Based on numerous transcriptomic data (Additional Table S16), full-length cDNAs [24], and homologous proteins, we predicted 51,074 high-quality protein-coding loci with intact structures in moso bamboo (Additional Table S17). The average introns and exons were 668 bp and 284 bp in length, respectively (Fig. 1c and Additional Table S18). Combinations of single-molecule real-time sequencing and manual verifications were implemented to refine certain irrational predictions. According to our results, ~17% of the gene models were precisely refined by the UTR addition and internal structural adjustment (Additional Table S19). According to the completeness assessment of the annotation using BUSCO [25], moso bamboo (95.2%) was more complete than *Z. mays* (92.2%) but close to *O. sativa* (95.6%) (Fig. 1d and Additional Table S20). Compared with the previous annotation, 97.23% of the gene models in our analysis were identified in public databases, which facilitated the accurate detection of alternative splicing events (Additional Table S21). Detailed information regarding the gene model prediction and genome evolution are presented in Additional Tables S22-24 and Figs. S3-9. Additionally, the latest genome assembly and gene annotation were released at the *Giga*DB [26]. The entire dataset comprises genome assemblies, gene sets, a list of repeat elements, tRNAs, miRNAs, gene clusters, and the newly released bamboo genome, providing a reliable resource for many analyses, including genomic, genetic, and molecular biology experiments

Vast transcriptomic data generated using the Illumina and PacBio platforms

To facilitate the genome-wide investigation of the AS landscape in moso bamboo and comprehensively identify the factors that influence AS at the post-translational level, we performed high-throughput RNA sequencing (RNA-Seq) using the Illumina HiSeq-4000 platform. In total, 26 individual representative RNA samples were sequenced using 150 base paired ends (Additional Table S2 and Figs. S10-11). After preprocessing, we obtained an average of 90 million high-quality reads (~13.6 Gb) per sample, accounting for 92.78% of the raw reads. Approximately 80.57% of the high-quality reads were mapped to the reference genome at a unique position and designated unique reads (Additional Tables S3-4). According to the alignment distribution, most sequences were mapped in exonic regions. The exonic rate, which was defined as the fraction of reads mapping within exons, was on average 81.94%. The remaining reads were mapped in intronic regions (8.46%) and intergenic regions (9.6%) (Additional Table S5 and Figs. S12-13). An in-depth exonic coverage (~2,521× per sample) was detected (Additional Fig. S14). Therefore, the large-scale, in-depth high-quality transcriptomic data, together with a high-quality reference genome, contributed to an accurate AS identification in moso bamboo.

To accurately identify the full-length splice isoforms, we sequenced the bamboo transcriptome using the PacBio platform. The full-length cDNA sequencing of alternatively spliced isoforms (Iso-Seq) used RNA from a mixture of 26 samples. According to the length distribution of the transcripts in all samples (Additional Table S6), we constructed 3 SMRTbell libraries (1-2 kb, 2-3 kb, and >3 kb) for the mixed sample and sequenced 9 cells, generating ~562 Mb of raw data and 214,372 reads-of-insert (ROIs), including 133,599 full-length ROIs (containing a 5' primer, 3' primer and a poly(A) tail); the remaining ROIs were non-full-length ROIs (Additional Table S7 and Fig. S15). The accuracy evaluation based on aligning the ROIs against the new genome showed that the per-nucleotide error was approximately 2.05% and consisted of mismatches (0.32%), insertion (0.98%) and deletions (0.75%).

Numerous genes underwent AS in moso bamboo

Based on the improved reference genome and large-scale transcriptome data, we performed a genome-wide analysis to identify AS in moso bamboo using the previous pipeline [10]. In total, 266,711 unique AS events were identified in 25,225 AS genes, accounting for *ca.* 49.39% of all annotated genes. Except for the 12,653 AS genes identified in the gene annotation, the remaining (12,572) genes were considered novel AS genes (Additional Fig. S16).

The Iso-Seq data were also utilized to detect AS in an analysis parallel to the RNA-Seq analysis. In total, 4,246 AS events and 2,218 AS genes were identified (Fig. 2a, b). According to the PacBio-Illumina overlapping analysis, which was performed to assess the validity of the AS prediction, 81.21% of the AS events and 97.34% of the AS genes identified in the Iso-Seq analysis completely overlapped with those in the RNA-Seq analysis. Among the four main AS types, on average, 80.37% of the AS events and 95.59% of the AS genes also overlapped (Additional Fig. S17). Thus, a higher proportion of the PacBio-Illumina overlapping AS genes is a strong indicator of the validity of the computationally predicted AS.

The AS gene number was strongly and positively correlated with the AS event number (correlation coefficient = 0.97, Mann-Whitney U test with p-value <0.05) (Fig. 2c). In subsequent analyses, we defined the four main AS types represented intron retention (IR), alternative 3' splice site donor (A3SS), alternative 5' splice site acceptor (A5SS), and exon skipping (ES), and we also defined the other AS types represented some AS types except the above four main AS types. The four main AS types were detected in the AS events in moso bamboo according to the canonical splicing patterns (GT-AG, GC-AG, and AT-AC splice sites). As shown in Fig. 2b, intron retention (IR, 38.22%) represented the most abundant type of AS event, followed by alternative 3′ splice site acceptor (A3SS, 20.20%) and alternative 5′ splice site acceptor (A5SS, 10.48%). Exon skipping (ES, 2.92%) was the least prevalent type among the four main AS types.

As the functional implication of AS genes, the enrichment analysis result showed 885 genes, which alternatively spliced in all samples, significantly enriched in RNA metabolic processing, mRNA processing, RNA processing and RNA splicing in the processes (Additional Table S25). Since AS possess strong specificity to different tissues or developmental stages, we identified 181,105 tissue-specific AS events (67.57%), which account for two-thirds of the AS events (termed as among-tissue). Then, the remaining two-third of the AS events were detected based on comparisons of the transcript isoforms within individual tissues (termed as within-tissue) (Additional Fig. S18).

The transposable element (TE) analysis showed 26,366 genes have TE insertion, accounted for 51.62% of all genes, and the total length of TE-insertion in genes was ~46 Mb. According to the different position of the TE-inserted intron, TE-introns mainly concentrated in the front and rear of a gene (Additional Fig. S19). Additionally, the usage and distribution of splice sites demonstrated GT-AG splice sites were the most abundant, corresponding to 97.31% of entire AS events, followed by GC-AG (2.33%) and GT-AT (0.32%) splice sites (Additional Fig. S20). Except for canonical splice site (GT-AG, GC-AG, and AT-AC), the

remaining 2,406 splice sites were identified as non-canonical splice sites, contained 2,373 GT-AT splice sites and 33 splice sites of other types.

Evolutionary analysis of AS in moso bamboo

Based on the genome-wide identification of orthologous genes in the selected 8 plants (*Amborella trichopoda*, *A. thaliana*, *Elaeis guineensis*, *B. distachyon*, *O. sativa*, *Spirodela polyrhiza*, *S. bicolor* and *Ph. edulis*) and the species divergence time in a phylogeny tree (Fig. 3a), we identified eight orthologous gene datasets. For instance, dataset8 (D8) represented common orthologous genes in the selected 8 plants, which were located in an early divergence time in our constructed phylogeny. D7 represented common orthologous genes in the selected 7 plants except from *A. trichopoda* (the specie with the earliest divergence time) and D7 doesn't contain orthologues genes in D8. And so on. Thus, D1 represented bamboo-specific orthologous genes, which were located in later divergence time. According to a previous study [27], we obtained the divergence times of genes based on the presence and absence of orthologs in the phylogeny. In our subsequent study, thus, we considered the bamboo-specific genes (D1) as a poorly conserved gene dataset and the common genes in all selected plants (D8) as a highly conserved gene dataset, and the degree of conservation decreased monotonically from D8 to D1. AS was detected in all datasets, but the proportion of AS genes in each dataset gradually decreased from D8 to D1 (Mann-Whitney U test with $p<0.05$). This trend was also observed in the two other datasets, i.e., removing common genes in more than two gene datasets in eight original datasets and using single-copy genes in eight original datasets. The eight original datasets were derived from the genome-wide identification of orthologous genes in the selected 8 plants. Therefore, the robust pattern, i.e., more conserved genes having more AS genes, should also exist in bamboo.

We investigated the distribution pattern of the four focal AS types in each dataset and found identical trends (IR>A3SS>A5SS>ES) (Fig. 3b), but the proportion of the AS types significantly differed. The proportion of IR in D8 was 60.80%, which was ~6-folds of that in D1 (11.88%). The ratio of the other AS types increased as the level of conservation decreased. In all datasets, the number of AS events gradually decreased from D8 to D1 (Fig. 3c). The most abundant AS events appeared in D8, and the least abundant AS events were detected in D1. Additionally, compared with the AS events among the genes expressed in samples with different specificities (maxTs) (for details, see Methods), the maxTs obviously increased from D8 to D1, representing an enhancement in the sample specificity from a highly conserved gene dataset to a

poorly conserved dataset (Fig. 3d). Altogether, the conserved genes tended to have more AS genes, more AS events and less specificity.

We also examined the correlations among the gene length, CDS length, intron length, exon number, exon cassette length, and intron cassette length in all datasets (Additional Fig. S21). All genes in the different datasets were positively correlated with the gene length, CDS size, intron size, and exon number and negatively correlated with the exon cassette length and intron cassette length. Moreover, the distribution of the TE genes in the 8 datasets was examined and a substantially negative correlation was observed, indicating that the more conserved genes had more TE insertions.

Expansion of the gene family involved in the lignin biosynthesis pathway and implications for gene functional diversity

We systematically identified 13 gene families involved in the lignin biosynthesis pathway using the six genome sequences of *A. thaliana*, *B. distachyon*, *O. sativa*, *Ph. edulis*, *P. trichocarpa*, and *S. bicolor*. The expansion of most families was detected in bamboo (Additional Table S26). Each gene had multiple copies in the bamboo genome, and the total size of the gene families in the lignin biosynthesis pathway was the largest in bamboo, with an average of ~19 copies per family. The most and least copy numbers were detected in the peroxidase gene family (*POD*, 77 genes) and *p*-coumarate 3-hydroxylase gene family (*C3H*, 3 genes), respectively. Additionally, we calculated the synonymous substitution rate analysis for 13 gene families evolved in the lignin biosynthesis using the yn00, which was a package in PAML to estimate synonymous and nonsynonymous substitution rates. Then, the Ks rate was translated to the divergence time by the formula $T=Ks/2r$ ($r=6.5\times10^{-9}$). As shown in Additional Fig. S22, the result indicated that the divergence time of the lignin biosynthesis genes occurred at the 5~16 million year ago (Mya), which correspond to the whole genome duplication (WGD) time 7~12 Mya in the moso bamboo genome [4].

Moreover, we performed an AS analysis of the genes in the lignin biosynthetic pathway. In total, 10 of the 13 families had AS genes accounting for more than half of the total, except for the ferulate 5-hydroxylase (F5H) gene family, which had a low proportion, and the CHS and caffeic acid *o*-methyltransferase (COMT) gene families, in which AS genes were not detected. A high percentage (>75%) of AS events was observed in the 4-coumarate: CoA ligase (4CL), hydroxycinnamoyl transferase (HCT) and cinnamyl alcohol dehydrogenase (CAD) gene families. In addition, we tested for positive selection in the gene families

involved in the lignin biosynthetic pathway using a branch-site model. Several genes in two gene families, i.e., *HCT* and *CAD*, exhibited positive selection. The information provided by the phylogenetic relationship using the best model and log likelihood ratio (lnL) was provided in Additional Table S27.

Discussion

High-throughput genome sequencing and assembly strategy were broadly applied in current plant genomic studies with the development of new technologies and more useful data. In 2013, our initial analysis of the *Ph. edulis* genome provided a genome-wide perspective of the structures of the genome and genes, the histories of the whole-genome duplication events, and the functional genes in critical functional categories[4]. In the present study, we enhanced both the precision and contiguity of the *Ph. edulis* genome and updated its annotation, accurately positioning the bamboo genome in an evolutionary landscape by performing comparative studies involving different species. Additionally, various biological characteristics of bamboo were studied in great detail using knowledge obtained from the latest version. Therefore, the chromosome-level reference genome and refined annotation paved the way for extra-genomic studies of bamboo and other related plants.

We provided the global AS landscape in bamboo based on a large amount of high-throughput data from RNA-Seq and Iso-Seq. These data enabled the accurate detection of transcripts with a low expression level and the acquisition of the complete gene structure, particularly in the AS analysis. A series of AS analyses expanded our holistic understanding of AS in bamboo during post-transcriptional regulation, including the identification of AS genes and AS events, the distribution of the AS types, the use of a splice site, the length distribution of an alternative exon, etc. AS is considered a major mechanism responsible for multicellular diversity and the enhancement of the number of proteins from a limited repertoire of genes. For example, by combining one exon of four alternatively spliced regions that contain 12, 48, 33, and 2 alternative exons each, it was possible to generate, at most, 38,016 protein isoforms (12×48×33×2) from the *Dscam* gene in *Drosophila* [28]. In bamboo, we identified 266,711 AS events and 25,225 genes in all samples, and on average, 15,971 AS events and 9,080 AS genes were detected in each sample. Thus, AS might be tissue specific, and the actual AS percentages in bamboo might be underestimated. More AS events, supported by transcripts with a low expression level, can be detected as the sequencing depth

increases [29]. Additionally, the distribution of the AS type is consistent with that in *Arabidopsis* [5,9,29], soybean [10], and maize [13]. Nevertheless, a higher percentage of IR (38.22%) and other AS types (total 28.18%) were observed in bamboo. This higher percentage may be due to the unique features of bamboo and/or the depth of the sequencing, which will be addressed in a comprehensive comparative analysis using additional data in a future study. In addition, TEs constitute crucial gene regulatory elements and influence gene transcription and gene expression [30]. We did not detect a noticeable relationship between the TE genes and AS genes. Although previous reports have indicated that TE inserted within an intron interferes with the normal splicing pattern of pre-mRNA, provoking various forms of alternative splicing [31], our result implied that TE might be a driving force during the formation process of AS in bamboo. Furthermore, the identification of splice sites in an individual gene may provide an essential resource for fully understanding alternative splicing and isoform construction [32,33]. With respect to their distribution, the main AS types (i.e., GT-AG, GC-AG, and AT-AC) were consistent with those previously observed in animals and other plants [19].

More AS events were identified in the sample with vigorous growth, which is consistent with the previous studies [29,34]. However, according to our observations, the rhizome tissue had more AS events than the root tissue in moso bamboo, which may because the two tissues play differential roles during bamboo development. Photoassimilates were unavailable during the rapid growth of the moso bamboo shoots since no leaves were growing [35], and thus, the large amount of nutrients and energy in the shoot mainly originate from the attached matured bamboos through underground rhizomes. Therefore, as a rhizomatous plant, the rhizome in moso bamboo plays a critical role in the transportation of nutrients and energy, which might explain the higher number of AS events detected in the rhizome. Moreover, to unveil the relationship between the incredible growth speed and AS in the shoots of moso bamboo, we selected shoots with 4 different heights and sampled 3 internodes (i.e., top, middle, and base) from each shoot according to the classification of shoot development. Obvious differences were observed in the AS event numbers in the final three shoot developmental stages, likely contributing to the fast growth during shoot development.

We performed an evolutionary analysis to unveil the relationship between AS and evolution using a comparative genome analysis. To date, the relationship between conservation and AS remains unknown. To tentatively address this issue, we performed a genome-wide analysis to examine AS in eight gene datasets

with different degrees of conservation. The AS genes were more likely to be enriched in the highly conserved gene datasets, and these AS genes had more AS events. This finding was robust because we analyzed using the orthologous genes only in one dataset and using single-copy genes in selected species, respectively. Previous reports have illuminated that duplication is a major source of functional diversity and the generation of new genes in plants [36], and new genes have generally low expression and suffer certain restrictions [37]. Altogether, we proposed that the relationship between conservation and AS may be associated with gene evolution and the generation of new genes. New genes might first generate a single-functional gene without an AS event and then gradually form multifunctional and conserved genes with many AS events [22]. Conserved genes tend to be the hubs in gene-gene interaction networks, indicating their functional diversity, and during the gradual evolutionary process, newly generated genes are gradually added to this network and acquire pleiotropic roles [38]. Additionally, the four main AS types were abundant in the highly conserved gene datasets, and many other AS types appeared in the poorly conserved datasets. Thus, the four main AS types were conserved, and other types might represent an intermediate stage. The distribution of the AS types depicted that IR occupied the dominant position, indicating that the importance of IR could be inferred from inspecting its prevalence throughout evolution in plants. The allocation in animals and yeast differs from that in plants. The most abundant AS event is ES, followed by AA and AD, while IR is the least common [39]. The discrepancies in the occurrence of the AS models between plants and animals suggest that differences exist between plants and animals in the genomic structure and mechanism of splice site recognition [40].

According to our results, the highly conserved gene datasets had more AS genes and events, which either produce functional alternative protein-coding transcripts with distinct functions in biological processes or modulate the functional spliced transcript level by producing certain non-coding transcripts [22]. We hypothesize that the highly conserved genes with more AS events might be critical for evolution and function in generating gene functional diversity and the generation process of the highly conserved genes might undergo rigorous regulation during long-term evolution since the poorly conserved genes had less AS events than the highly conserved genes. Additionally, compared with the poorly conserved gene datasets, the highly conserved AS gene datasets had a low tissue-specific expression profile, indicating these genes might be core genes in fundamental functions, such as serving as hubs in gene-gene networks. Therefore, we proposed that functionally important genes are generated by more frequent AS events. As an essential biological

process, AS plays a crucial role in acquiring more functions, which might explain why the highly conserved AS possesses more AS events. We hypothesize that this phenomenon likely applies not only to bamboo but also to other plants or even animals.

Furthermore, we observed the relationship between the AS genes in different conserved datasets and gene structure features. The AS genes in the highly conserved gene datasets possessed a longer gene length and more CDS, introns and exons and a shorter exon and intron cassette length. The architecture of the longer introns and shorter exons detected in the highly conserved gene dataset might be helpful in matching the constraints imposed by splicing recognition in the evolutionary process [21]. The exon-intron architecture in the different conserved genes might indicate that the splice-site choice and transcription by RNA polymerase II is changed during evolution [41,42]. During the evolutionary process, a new gene might be generated by duplication, which then forms less AS under strict constraints. Subsequently, functional AS is gradually generated and then evolves more functions largely through inducing changes in the gene structure, such as increasing the gene lengths, shorting the lengths of the exon cassette, inducing site mutations, etc.

Lignin represents a class of complex aromatic heteropolymers of monolignols that encrusts and interacts with the cellulose/hemicellulose matrix of the secondary cell wall [43]. Lignin accounts for up to ~25% of the total dry weight in bamboo [2]. We performed a deep examination by combining AS and evolution analyses of the lignin biosynthesis pathway. The expansion of the gene families in the lignin biosynthesis pathway was detected in bamboo. Combined with the results of the divergence time of the lignin biosynthesis genes and our previous study [4], we estimated the occurrence of a putative WGD event at 7~12 Mya in the moso bamboo genome, suggesting that there might have been a tetraploidization event during bamboo history [4]. Then, the ancient tetraploid moso bamboo evolved into a current diploid moso bamboo. Additionally, WGD could provide more gene copies, which facilitated evolving the genes with new functions [44]. Therefore, the expansion of the lignin biosynthesis genes in moso bamboo could be due to the occurrence of WGD event. Additionally, the two gene families (i.e., *HCT* and *CAD*) underwent more AS events and positive selection. HCT generates lignin by catalyzing *p*-coumaroyl CoA [45]. Then, *p*-coumaroyl CoA is also catalyzed by CHS to generate flavonoids. HCT and CHS compete with each other to bind *p*-coumaroyl CoA. In bamboo, the *HCT* family has more members and AS events than the *CHS* family, which indicate that the *HCT* family might be in a dominant position in the competition to bind *p*-coumaroyl CoA. CAD catalyzes many different substrates to generate different types of lignin. The aromatic lignin polymers

commonly found in bamboo are composed of three monolignols, namely, *p*-hydroxyphenyl (H), vanillin (G), and syringaldehyde (S). Previous studies have shown the abundance of G and S lignin and a small amount of H lignin in bamboo [2]. The *CAD* family expansion in bamboo and positive selection may explain the different preferences of substrates to generate different proportions of monolignols in bamboo. The abundance of AS events, gene expansion, and positive selection were all consistent with the phenomenon that bamboo is remarkably adaptive to produce lignin.

Conclusions

To deeply explore the AS profile in the evolutionary landscape in bamboo, we improved reference genome and refined the annotation of moso bamboo. Based on the chromosome-level genome sequence and the abundant transcriptomic data from multiple tissues from six main bamboo producing areas in China, we provide a comprehensive AS perspective of moso bamboo by identifying 266,711 unique AS events in 25,225 AS genes using both the Illumina and PacBio sequencing technology platforms. Moreover, the integrated analysis of the AS results in bamboo and comparative analysis among eight representative plant species exhibited that the more conserved genes tended to accumulate higher transcript levels and exhibit less specificity. Finally, by studying the lignin biosynthesis based on AS and evolution, we observed several characteristics of crucial genes related to lignin biosynthesis in bamboo, including gene family expansion, abundant AS and positive selection. In summary, these results will likely provide important resources for studies investigating bamboo’s specificity as a woody plant in the grass family and exploring AS in the bamboo evolutionary landscape.

Method

Plant material collection

To obtain a comprehensive AS profile, the moso bamboo (*Phyllostachys edulis*) used in these experiments was collected from six main bamboo producing areas in China during the Spring of 2105, including (1) YiXing, JiangSu Province (N:31°15′08.41″, E:119°43′42.55″, 212 M), (2) TianMu Mountain, ZheJiang Province (N:30°19′13.42″, E:119°26′55.21″, 480 M), (3) XianNing, HuBei Province (N:29°81′10.02″, E:114°31′21.12″ 150 M), (4) TaoJiang, HuHan Province (N:28°28′39.74″, E:112°11′18.62″, 320 M), (5)

GuiLin, GuangXi Province (N:28°28′39.74″, E:112°11′18.62″, 216 M) and (6) ChiShui, GuiZhou Province (N:28°28′15.27″, E:105°59′41.43″, 120 M). Twenty-six tissues were collected, including the rhizome, root, shoot, leaf, sheath, and bud, during different developmental stages. Each mixed sample was collected from the above six areas. Detailed information regarding the biological samples is provided in Additional Table S19.

Genome sequencing, assembly and annotation

We assembled the moso bamboo genome using WGS and Hi-C strategies and annotated the new genome sequence as described in a previous study [46] and Additional Files. The detailed descriptions of this section were provided in Protocol.io [47].

Hi-C library preparation, sequencing and assembling

The construction of Hi-C library was prepared as previously described [46] and the detailed descriptions were presented in Additional Files.

RNA isolation and Illumina RNA-Seq library construction

We used standard methods of RNA isolation, purity, concentration, reverse transcription, and cDNA library construction, as described in the previous study [48]. All cDNA libraries were constructed and normalized as described in the Additional File.

RNA-Seq using the Illumina platform

After passing quality control, the pooled libraries were optically examined using an Illumina Cluster Station and were then 150 base paired end sequenced on the Illumina HiSeq-4000 platform according to the manufacturer's protocols. Finally, the quality of the reads was evaluated, and the low-quality reads were filtered using FastQC (version 0.11.3, http://www.bioinformatics.babraham.ac.uk/projects/fastqc/) with the default parameters. The statistics of the key metrics applied to the RNA-Seq data were calculated using RNA-SeQC (version1.1.8) [49] with the default parameter.

RNA-Seq data analysis

The detailed description of this section was provided in Protocol.io. Briefly, the adaptor sequences and low-quality sequences were trimmed using Trimmomatic (version 0.33)[50] during the preprocessing of the

RNA-Seq data. Then, the cleaned data were mapped to the improved genome using HISAT2 (version 2.0.2) [51] with the following modifications from the default parameters: maximum intron length (4,000); specify strand-specific information (RF); and minimum score (L, -0.1, -0.1); report alignments tailored to transcript assemblers were allowed. The empirical transcripts in each sample were obtained using Cufflinks (version 2.2.1) [52] after the reads were aligned. The default parameters were used, except for the following parameters: the minimum isoform fraction (0.05); the small anchor fraction of the spliced reads (0.05); the minimum intron length (20); the maximum intron length (4,000); the library type (fr-firststrand); the corrected frag bias; and the corrected multi-read. ASTALAVISTA (version 4.0) [53,54] was used with the default parameters to identify the AS genes and events after the different assembled transcript isoforms were mapped to the corresponding gene model using Cuffcompare, which is a component of the Cufflink program. The main four types, *i.e.,* IR, A3SS, A5SS, and ES, were analyzed in the AS types. In addition, an enrichment analysis of the different genes was conducted using Ontologizer (version 2.0) [55] with the annotations from the Gene Ontology (GO) database (www.geneontology.org). We also calculated the sample specificity (Ts) values in each sample and each gene based on the expression level (FPKM values, the total number of fragments per kilobase of sequence per million reads mapped). A detailed description is provided in the previous report [56]. Briefly, Ts is defined as the fractional expression of a gene in one sample tissue relative to the sum of its expression in all samples. Thus, the maximum Ts value (maxTs) of a gene serves as an indicator of the sample specificity. Higher specificity values represent more tissue-specific expression [57].

Construction and sequencing of the Iso-Seq library

The construction of Iso-Seq library and sequencing were performed based on the PacBio manufacturer's protocol as previously described [58]. According to the length distribution of the transcripts predicted by bioinformatics (Additional Table S22), three SMRTBell libraries (1-2 kb of 3 cells, 2-3 kb of 2 cells, and >3 kb of 4 cells) were size-selected and a total of 9 SMRT cells were sequenced on the PacBio platform.

Iso-Seq data analysis

The qualified sequencing data produced using PacBio RS II were processed to obtain consensus full-length isoforms. The isoforms from the multiple libraries were merged, and redundancy was removed to obtain the final consensus isoforms after processing the reads of the insert, classifying, and clustering. The assembled transcripts were mapped to the reference genome using PASA (version 2.0.2, http://pasapipeline.github.io/)

with the default parameters. Then, similar to the short-read data, the output file of the gtf was analyzed using ASTALAVISTA with the default parameters to identify the AS.

Evolutionary analysis

We identified gene families, constructed a phylogenetic tree, predicted divergence times as a previously study [4] and the detailed information was provided in Additional Files and Protocol.io.

Genome-wide identification of genes involved in the lignin biosynthetic pathway

The five genome sequences of *A. thaliana* (TAIR10), *B. distachyon* (v3.1), *O. sativa* (v7.0), *Populus trichocarpa* (JGI2.0.31), and *S. bicolor* (v3.1) were downloaded from the ENSEMBL database [59]. According to vast literature-based investigations, 140 genes from the lignin biosynthetic pathway was experimentally validated from previous studies (Additional Table S28), and then, these known genes were collected and used as the query sequences for further identification. We identified lignin biosynthetic genes using a BLAST search and domain analysis as described in the previous article[60]. Briefly, we performed standard nucleotide BLAST searches (version 2.2.26) against the six genome sequences including moso bamboo using the coding sequence of the known genes with the following cut-off values: E-value $<1e^{-10}$; identity >95%; and coverage rate >40% query sequence. The filtered sequences were subsequently analyzed by hmmsearch (version 3.1b2) using the Pfam-A.hmm database (released 2017/03/31). Consequently, unclear sequences with incomplete domains were discarded by manual correction. A phylogenetic tree was constructed using the same protocol described above.

Positive selection analysis

We performed a positive selection analysis based on the coding sequences of the lignin biosynthetic pathway. In each family, protein sequences were first aligned by PROBCONS (version 1.12) [61] using the default parameters, except for the option of iterative refinement, for which we used 1,000 iterations. Then, we backed the alignment to its corresponding coding sequences. After obtaining the conserved blocks from the sequence alignment using Gblocks (version 0.91b) [62], jModelTest (version 2.1.6) [63] was used to find the best model according to the Bayesian Information Criterion. Subsequently, PhyML (version 3.0) [64] was used to reconstruct the phylogenetic tree under the best model, with bootstrapping of 1,000 replicates. Finally, certain branches selected from the phylogenetic tree were examined in a positive selection analysis

using PAML (version 4.8) [65] and a branch-site model.

Availability of data and materials

Short-read sequencing data from this whole-genome shotgun project can be deposited at European Molecular Biology Laboratory (EMBL) under the accession ERP001340. RNA-Seq raw sequence data and Iso-Seq raw sequence data for a mixture sample were deposited in NCBI Short Read Archive database under the accession numbers: SRX2408703-28 and SRR7032261-69, respectively. The chromosome-level genome and the latest annotation were provided in *Giga*DB. Additionally, protocols to the methods are uploaded to Protocols.io (https://www.protocols.io/researchers/hansheng-zhao).

Declarations

Author's Contribution

Experimental design: H.Z., Z.G., C.C., B.F. S.W., Z.C., H.Y. and Z.J. Experimental preformation: H.Z., J.W.; H.Z., L.C., Z.X., C.Z. and Y.W. Data analysis: W.Y., H.S., L.L., S.W., Y.Y., Y.L., Q.G., C.C., X.C. and H.X. The providing of reagents, materials and analysis tools: H.Z. and Z.G. Article writing: H.Z., Z.C., Z.G. and B.F. All of the authors read and approved the final manuscript.

Competing interests

The authors declare that they have no competing interests.

Acknowledgements

This work received financial support from the Special Fund for Forest Scientific Research in the Public Welfare from State Forestry Administration of China (No. 201504106), and the Sub-Project of National Science and Technology Support Plan of the Twelfth Five-Year in China (No. 2015BAD04B03 and No. 2015BAD04B01).

References

1. Zhao H, Zhao S, International Network for Bamboo and Rattan, Fei B, Liu H, Yang H, et al. Announcing the Genome Atlas of Bamboo and Rattan (GABR) project: promoting research in evolution and in economically and ecologically beneficial plants. GigaScience. 2017;6:1–7.

2. Bai Y-Y, Xiao L-P, Shi Z-J, Sun R-C. Structural variation of bamboo lignin before and after ethanol organosolv pretreatment. International Journal of Molecular Sciences. 2013;14:21394–413.

3. Jiang Z. Bamboo and Rattan in the World. Beijing: China Forestry Publishing House.

4. Peng Z, Lu Y, Li L, Zhao Q, Feng Q, Gao Z, et al. The draft genome of the fast-growing non-timber forest species moso bamboo (*Phyllostachys heterocycla*). Nature Genetics. 2013;45:456–61.

5. Filichkin SA, Priest HD, Givan SA, Shen R, Bryant DW, Fox SE, et al. Genome-wide mapping of alternative splicing in *Arabidopsis thaliana*. Genome research. 2010;20:45–58.

6. Pan Q, Shai O, Lee LJ, Frey BJ, Blencowe BJ. Deep surveying of alternative splicing complexity in the human transcriptome by high-throughput sequencing. Nature Genetics. 2008;40:1413–5.

7. Wang ET, Sandberg R, Luo S, Khrebtukova I, Zhang L, Mayr C, et al. Alternative isoform regulation in human tissue transcriptomes. Nature. 2008;456:470–6.

8. Zhang PG, Huang SZ, Pin A-L, Adams KL. Extensive divergence in alternative splicing patterns after gene and genome duplication during the evolutionary history of Arabidopsis. Molecular Biology and Evolution. 2010;27:1686–97.

9. Marquez Y, Brown JWS, Simpson C, Barta A, Kalyna M. Transcriptome survey reveals increased complexity of the alternative splicing landscape in Arabidopsis. Genome research. Cold Spring Harbor Lab; 2012;22:1184–95.

10. Shen Y, Zhou Z, Wang Z, Li W, Fang C, Wu M, et al. Global dissection of alternative splicing in paleopolyploid soybean. Plant Cell. 2014;26:996–1008.

11. Mandadi KK, Scholthof K-BG. Genome-wide analysis of alternative splicing landscapes modulated during plant-virus interactions in *Brachypodium distachyon*. Plant Cell. 2015;27:71–85.

12. Li Q, Xiao G, Zhu Y-X. Single-nucleotide resolution mapping of the *Gossypium raimondii* transcriptome reveals a new mechanism for alternative splicing of introns. Molecular Plant. 2014;7:829–40.

13. Thatcher SR, Zhou W, Leonard A, Wang B-B, Beatty M, Zastrow-Hayes G, et al. Genome-wide analysis of alternative splicing in *Zea mays*: landscape and genetic regulation. Plant Cell. 2014;26:3472–87.

14. Zhang G, Guo G, Hu X, Zhang Y, Li Q, Li R, et al. Deep RNA sequencing at single base-pair resolution reveals high complexity of the rice transcriptome. Genome research. 2010;20:646–54.

15. Rühl C, Stauffer E, Kahles A, Wagner G, Drechsel G, Rätsch G, et al. Polypyrimidine tract binding protein homologs from Arabidopsis are key regulators of alternative splicing with implications in fundamental developmental processes. Plant Cell. 2012;24:4360–75.

16. Staiger D, Brown JWS. Alternative splicing at the intersection of biological timing, development, and stress responses. Plant Cell. 2013;25:3640–56.

17. Li W, Lin W-D, Ray P, Lan P, Schmidt W. Genome-wide detection of condition-sensitive alternative splicing in Arabidopsis roots. Plant Physiology. 2013;162:1750–63.

18. Cui P, Zhang S, Ding F, Ali S, Xiong L. Dynamic regulation of genome-wide pre-mRNA splicing and

stress tolerance by the Sm-like protein LSm5 in Arabidopsis. Genome Biology. 2014;15:R1.

19. Reddy ASN. Alternative splicing of pre-messenger RNAs in plants in the genomic era. Annual Review Plant Biology. 2007;58:267–94.

20. Barbosa-Morais NL, Irimia M, Pan Q, Xiong HY, Gueroussov S, Lee LJ, et al. The Evolutionary Landscape of Alternative Splicing in Vertebrate Species. Science. 2012;338:1587–93.

21. Keren H, Lev-Maor G, Ast G. Alternative splicing and evolution: diversification, exon definition and function. Nature Reviews Genetics. 2010;11:345–55.

22. Roy SW, Irimia M. Splicing in the eukaryotic ancestor: form, function and dysfunction. Trends in Ecology Evolution. 2009;24:447–55.

23. Chen RY, Li XL, Song WQ, Liang GL, Zhang PX, Lin RS, et al. Chromosome atlas of major economic plants genome in China. Tomus 4. Chromosome atlas of various bamboo species. Beijing: Science Press xxx, 646p.-illus.. ISBN 7030108353 Ch, En Chromosome numbers. Geog= 0 Systematics: ANGIOSPERMAE (GRAMINEAE)(KR, 200303867), 2003.

24. Peng Z, Lu T, Li L, Liu X, Gao Z, Hu T, et al. Genome-wide characterization of the biggest grass, bamboo, based on 10,608 putative full-length cDNA sequences. BMC plant biology. 2010;10:116.

25. Simão FA, Waterhouse RM, Ioannidis P, Kriventseva EV, Zdobnov EM. BUSCO: assessing genome assembly and annotation completeness with single-copy orthologs. Bioinformatics. 2015;31:3210–2.

26. Sneddon TP, Li P, Edmunds SC. GigaDB: announcing the GigaScience database. GigaScience. 2012;1:11.

27. Zhang YE, Vibranovski MD, Landback P, Marais GAB, Long M. Chromosomal redistribution of male-biased genes in mammalian evolution with two bursts of gene gain on the X chromosome. Barton NH, editor. PLoS Biol. 2010;8:e1000494.

28. Celotto AM, Graveley BR. Alternative splicing of the *Drosophila Dscam* pre-mRNA is both temporally and spatially regulated. Genetics. 2001;159:599–608.

29. Wang B-B, Brendel V. Genomewide comparative analysis of alternative splicing in plants. Proceedings of the National Academy of Sciences. 2006;103:7175–80.

30. Slotkin RK, Martienssen R. Transposable elements and the epigenetic regulation of the genome. Nature Reviews Genetics. 2007;8:272–85.

31. Feschotte C. Transposable elements and the evolution of regulatory networks. Nature Reviews Genetics. 2008;9:397–405.

32. Li Y, Li-Byarlay H, Burns P, Borodovsky M, Robinson GE, Ma J. TrueSight: a new algorithm for splice junction detection using RNA-seq. Nucleic Acids Research. 2013;41:e51–1.

33. Nilsen TW, Graveley BR. Expansion of the eukaryotic proteome by alternative splicing. Nature. 2010;463:457–63.

34. Barbazuk WB, Fu Y, McGinnis KM. Genome-wide analyses of alternative splicing in plants: opportunities and challenges. Genome research. 2008;18:1381–92.

35. Song X, Peng C, Zhou G, Gu H, Li Q, Zhang C. Dynamic allocation and transfer of non-structural carbohydrates, a possible mechanism for the explosive growth of Moso bamboo (*Phyllostachys heterocycla*). Scientific Reports. 2016;6.

36. Flagel LE, Wendel JF. Gene duplication and evolutionary novelty in plants. New Phytologist. 2009;183:557–64.

37. Lan X, Pritchard JK. Coregulation of tandem duplicate genes slows evolution of subfunctionalization in mammals. Science. 2016;352:1009–13.

38. Zhang W, Landback P, Gschwend AR, Shen B, Long M. New genes drive the evolution of gene interaction networks in the human and mouse genomes. Genome Biology. 2015;16:202.

39. Kim E, Magen A, Ast G. Different levels of alternative splicing among eukaryotes. Nucleic Acids Research. 2007;35:125–31.

40. Nakai K, Sakamoto H. Construction of a novel database containing aberrant splicing mutations of mammalian genes. Gene. 1994;141:171–7.

41. Chen M, Manley JL. Mechanisms of alternative splicing regulation: insights from molecular and genomics approaches. Nature Review Molecular Cell Biology. 2009;10:741–54.

42. Licatalosi DD, Darnell RB. RNA processing and its regulation: global insights into biological networks. Nature Reviews Genetics. Nature Publishing Group; 2010;11:75–87.

43. Martone PT, Estevez JM, Lu F, Ruel K, Denny MW, Somerville C, et al. Discovery of lignin in seaweed reveals convergent evolution of cell-wall architecture. Current Biology. 2009;19:169–75.

44. Taylor JS, Raes J. Duplication and divergence: the evolution of new genes and old ideas. Annual Review of Genetics. 2004;38:615–43.

45. Li X, Bonawitz ND, Weng J-K, Chapple C. The growth reduction associated with repressed lignin biosynthesis in Arabidopsis thaliana is independent of flavonoids. Plant Cell. 2010;22:1620–32.

46. Dudchenko O, Batra SS, Omer AD, Nyquist SK, Hoeger M, Durand NC, et al. *De novo* assembly of the Aedes aegypti genome using Hi-C yields chromosome-length scaffolds. Science. 2017;356:92–5.

47. Teytelman L, Stoliartchouk A, Kindler L, Hurwitz BL. Protocols.io: Virtual Communities for Protocol Development and Discussion. PLoS Biology. 2016;14:e1002538.

48. Zhao H, Sun H, Li L, Lou Y, Li R, Qi L, et al. Transcriptome-based investigation of cirrus development and identifying microsatellite markers in rattan (*Daemonorops jenkinsiana*). Scientific Reports. 2017;7:46107.

49. DeLuca DS, Levin JZ, Sivachenko A, Fennell T, Nazaire M-D, Williams C, et al. RNA-SeQC: RNA-seq metrics for quality control and process optimization. Bioinformatics. 2012;28:1530–2.

50. Bolger AM, Lohse M, Usadel B. Trimmomatic: a flexible trimmer for Illumina sequence data. Bioinformatics. 2014;30:2114–20.

51. Kim D, Langmead B, Salzberg SL. HISAT: a fast spliced aligner with low memory requirements. Nature Methods. 2015;12:357–60.

52. Trapnell C, Williams BA, Pertea G, Mortazavi A, Kwan G, van Baren MJ, et al. Transcript assembly and quantification by RNA-Seq reveals unannotated transcripts and isoform switching during cell differentiation. Nature Biotechnology. 2010;28:511–5.

53. Foissac S, Sammeth M. Analysis of alternative splicing events in custom gene datasets by AStalavista. Methods in Molecular Biology. 2015;1269:379–92.

54. Foissac S, Sammeth M. ASTALAVISTA: dynamic and flexible analysis of alternative splicing events

in custom gene datasets. Nucleic Acids Research. 2007;35:W297–9.

55. Bauer S, Grossmann S, Vingron M, Robinson PN. Ontologizer 2.0--a multifunctional tool for GO term enrichment analysis and data exploration. Bioinformatics. 2008;24:1650–1.

56. Marques AC, Tan J, Lee S, Kong L, Heger A, Ponting CP. Evidence for conserved post-transcriptional roles of unitary pseudogenes and for frequent bifunctionality of mRNAs. Genome Biology. 2012;13:R102.

57. Winter EE, Goodstadt L, Ponting CP. Elevated rates of protein secretion, evolution, and disease among tissue-specific genes. Genome research. 2004;14:54–61.

58. Wang B, Tseng E, Regulski M, Clark TA, Hon T, Jiao Y, et al. Unveiling the complexity of the maize transcriptome by single-molecule long-read sequencing. Nature Communications. 2016;7:11708.

59. Kersey PJ, Allen JE, Allot A, Barba M, Boddu S, Bolt BJ, et al. Ensembl Genomes 2018: an integrated omics infrastructure for non-vertebrate species. Nucleic Acids Research. 2018;46:D802–8.

60. Fischer S, Brunk BP, Chen F, Gao X, Harb OS, Iodice JB, et al. Using OrthoMCL to assign proteins to OrthoMCL-DB groups or to cluster proteomes into new ortholog groups. Current Protocol of Bioinformatics. Hoboken, NJ, USA: John Wiley & Sons, Inc; 2011;Chapter 6:Unit6.12.1–19.

61. Roshan U. Multiple sequence alignment using Probcons and Probalign. Methods in Molecular Biology. 2014;1079:147–53.

62. Talavera G, Castresana J. Improvement of phylogenies after removing divergent and ambiguously aligned blocks from protein sequence alignments. Systematic Biology. 2007;56:564–77.

63. Darriba D, Taboada GL, Doallo R, Posada D. jModelTest 2: more models, new heuristics and parallel computing. Nature Methods. 2012;9:772–2.

64. Guindon S, Dufayard J-F, Lefort V, Anisimova M, Hordijk W, Gascuel O. New algorithms and methods to estimate maximum-likelihood phylogenies: assessing the performance of PhyML 3.0. Systematic Biology. 2010;59:307–21.

65. Yang Z. PAML 4: phylogenetic analysis by maximum likelihood. Molecular Biology and Evolution. 2007;24:1586–91.

Figure legends:

Figure 1. The comparative results based on two versions of the moso bamboo genome.

(A) The distribution of the contigs between two versions of the moso bamboo genome. The parameters, N50 and N90 of contigs, were masked. (B) The distribution of the scaffolds between two versions of the moso bamboo genome. The parameters, N50 and N90 of scaffolds, were masked. (C) The box plots were showed based on two versions of the moso bamboo genome, including gene length, intron length, CDS length, cDNA length, single exon length, and single intron length. (D) The BUSCO assessment result was provided, including the five assessment results (two genomes and three annotations). Two genomes contained the previous WGS version and the latest chromosome-level version. Annotation v1 was based on the version 1 of the moso bamboo genome. Annotation v2.1 and Annotation v2.2 were based on the version 2 and Annotation v2.2 was manually verified by Annotation v2.1.

Figure 2. The distribution of AS genes and events and their correlation

(A) The distribution of AS genes in bamboo, including four main types and Iso-Seq result. (B) The distribution of AS events in bamboo, including four main types and Iso-Seq result. (C) the correlation between AS genes and events was provided. IR, A3SS, A5SS, and ES represents intron retention, alternative 3' splice site donor, alternative 5' splice site acceptor, and exon skipping, respectively.

Figure 3. The evolutionary analysis in plants across bamboo

(A) The phylogenetic tree with divergence times was constructed, including *Amborella trichopoda*, *Elaeis guineensis*, *Arabidopsis thaliana*, *Brachypodium distachyon*, *Oryza sativa*, *Spirodela polyrhiza*, *Sorghum bicolor* and *Ph. edulis*. Phylogenetic tree of the selected 8 plants with branches leading to bamboo as red line. The notation indicates the eight orthologous gene datasets (D8-D1) identified in our study. (B) a Venn of orthologous genes in related eight species was exhibited. (C) AS percentage and AS type were provided in D1 to D8 datasets, including redundant/non-redundant and multi-copy/single-copy. (D) increasing AS abundance and the decreasing specificity were displayed from D1 to D8.

Figure 4. The gene family expansion and AS abundance of bamboo in lignin biosynthetic pathway

A) A total of 13 families in lignin biosynthesis pathway were identified using six genomes of *A. thaliana*, *B. distachyon*, *O. sativa*, *Ph. edulis*, *P. trichocarpa*, and *S. bicolor*. Copy number and genes under positive

selection were added. B) The structure, distribution and types of AS and related gene expression level were exhibited in six gene families (4CL, C3H, CCR, HCT, LAC, and POD). The lignin biosynthetic enzymes are: PAL phenylalanine ammonia-lyase; TAL tyrosine ammonia-lyase; C4H cinnamate 4-hydroxylase; C3H 4-hydroxycinnamate 3-hydroxylase; COMT caffeic acid 3-O-methyltransferase; F5H ferulate 5-hydroxylase; 4CL 4-coumarate: CoA ligase; CCoA-3H coumaroyl-coenzyme A 3-hydroxylase; CCoA-OMT caffeoyl-coenzyme A O-methyltransferase; CCR cinnamoyl-CoA reductase; CAD cinnamyl alcohol, and HCT dehydrogenase hydroxycinnamoyl transferase.

Table 1. Statistics for the assembly of the moso bamboo genome using different sequence data

Statistics	WGS assembly		Hi-C assembly	
	Scaffold	Contig	Scaffold	Contig
Total number	19,285	76,900	19,684	84,758
Genome size (bp)	1,908,074,089	1,795,528,836	1,907,603,590	1,795,510,437
Gap number (bp)	112,545,253	0	112,093,153	0
Average length (bp)	98,940.84	23,348.88	96,911.38	21,183.96
N50 length (bp)	894,858	54,955	79,898,979	53,293
N90 length (bp)	115,487	11,757	44,603,463	10,445
Maximum length (bp)	5,406,526	738,589	137,299,170	738,589
Minimum length (bp)	926	157	318	1
GC content (%)	44.2	44.2	44.2	44.2

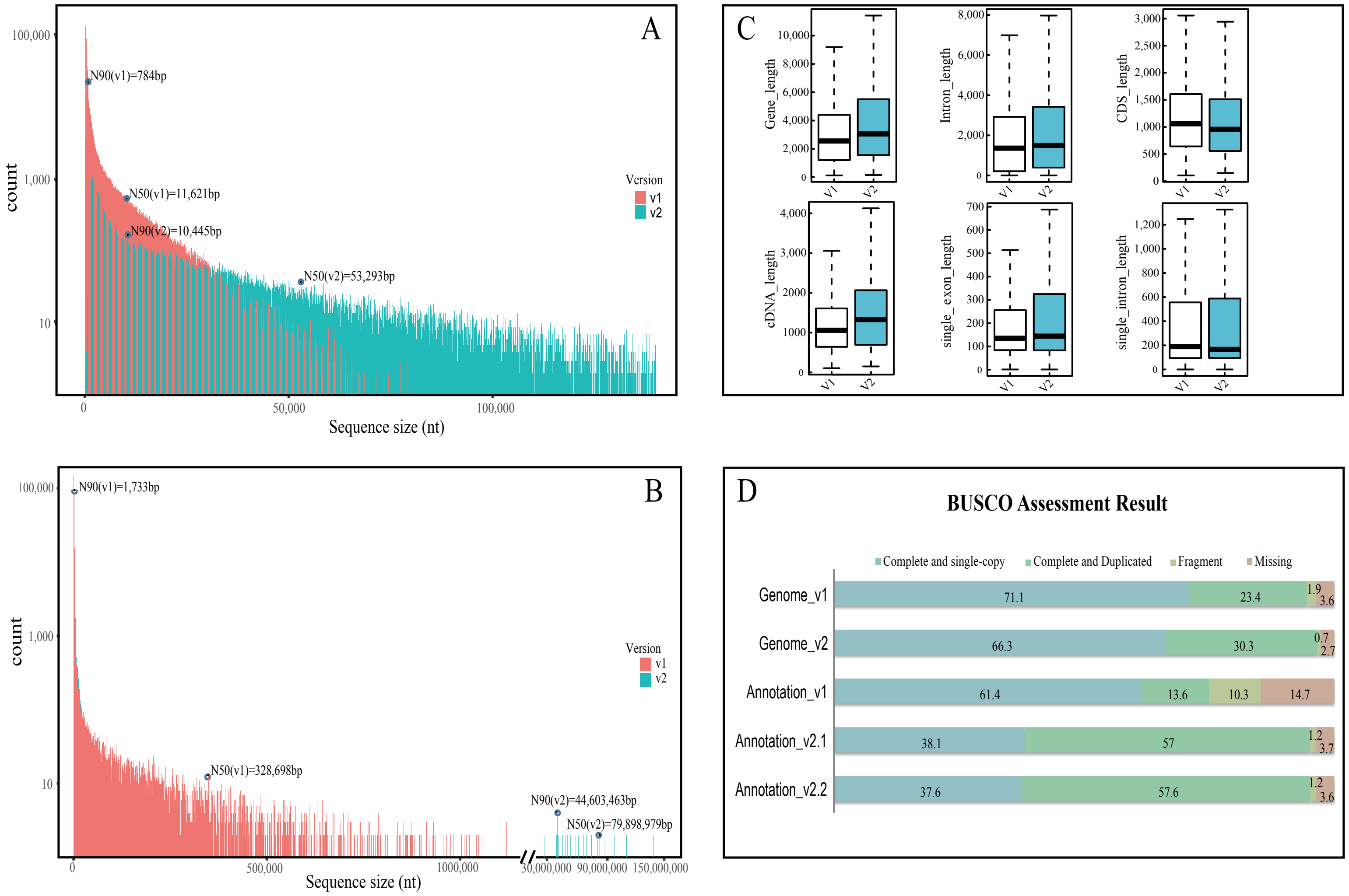
A
count
100,000
1,000
10
N90(v1)=784bp
N50(v1)=11,621bp
N90(v2)=10,445bp
N50(v2)=53,293bp
Version
v1
v2
0
50,000
100,000
Sequence size (nt)
B
100,000
1,000
10
N90(v1)=1,733bp
N50(v1)=328,698bp
N90(v2)=44,603,463bp
N50(v2)=79,898,979bp
500,000
1000,000
30,000,000
90,000,000
150,000,000
C
Gene_length
Intron_length
CDS_length
cDNA_length
single_exon_length
single_intron_length
V1
V2
D
BUSCO Assessment Result
Complete and single-copy
Complete and Duplicated
Fragment
Missing
Genome_v1
71.1
23.4
1.9
3.6
Genome_v2
66.3
30.3
0.7
2.7
Annotation_v1
61.4
13.6
10.3
14.7
Annotation_v2.1
38.1
57
1.2
3.7
Annotation_v2.2
37.6
57.6
1.2
3.6

Figure 2 Click here to download Figure Figure2.pdf

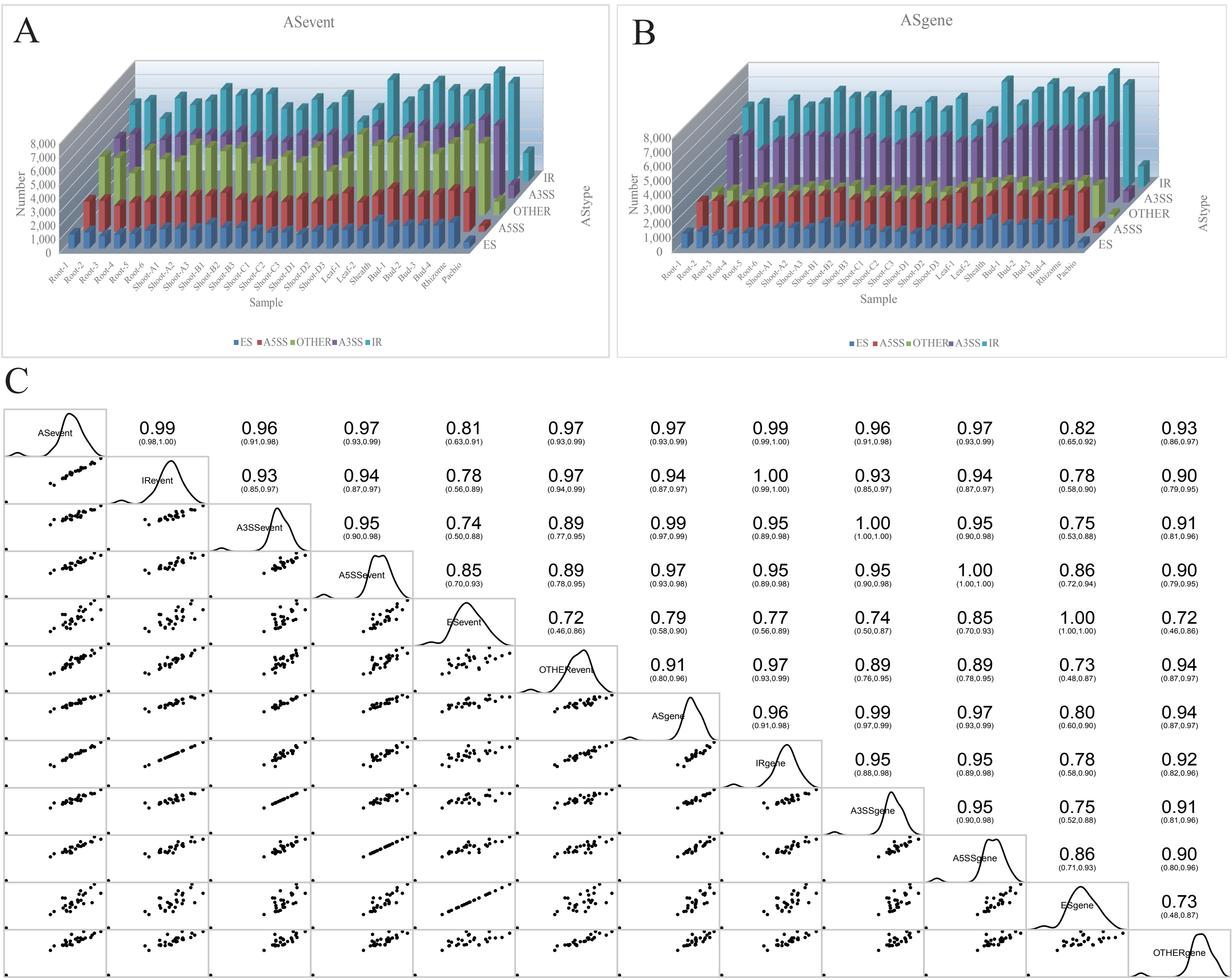

Figure 3-revised

Click here to download Figure Figure3-revised.pdf

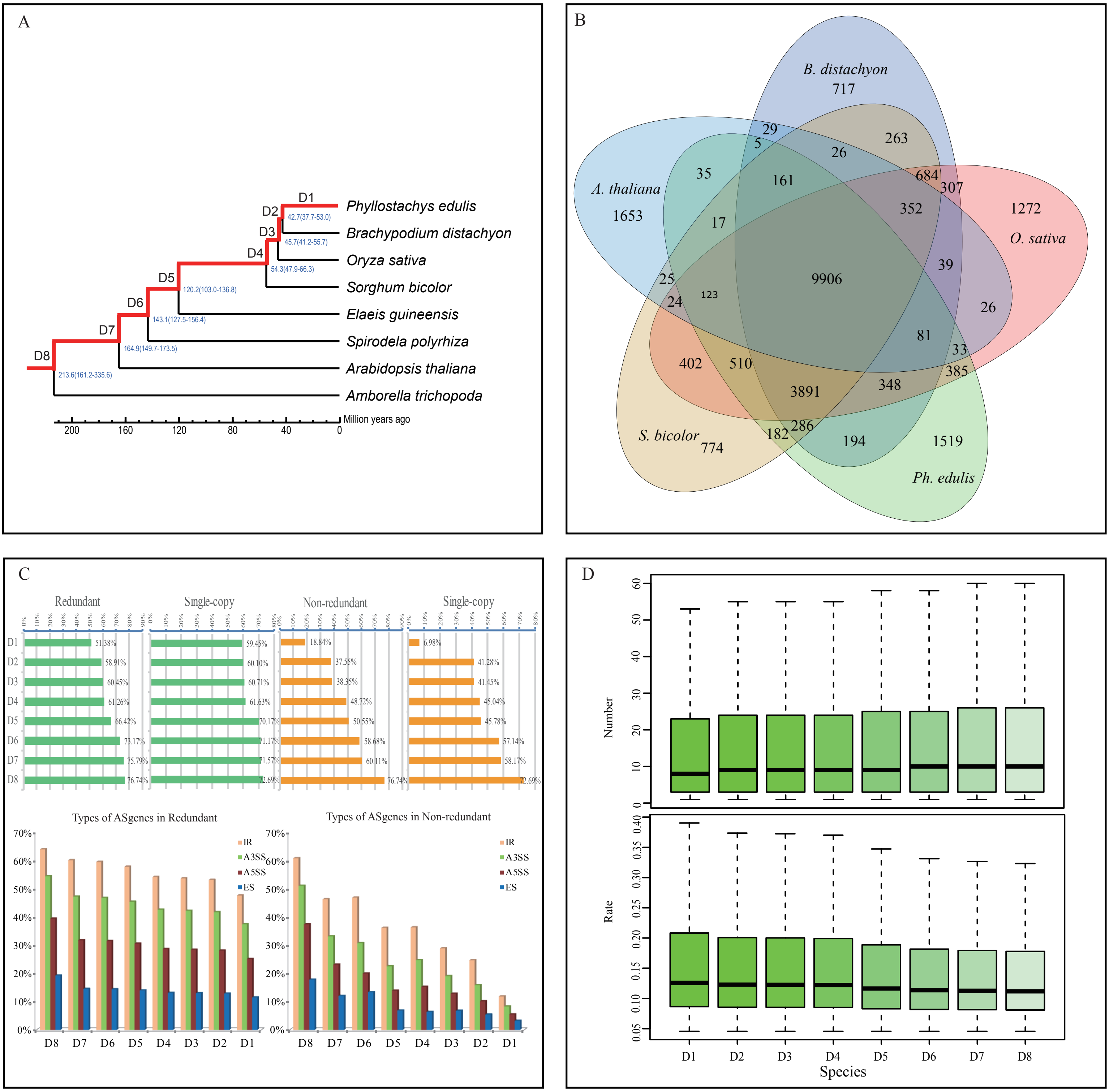

Click here to download Figure Figure4.pdf

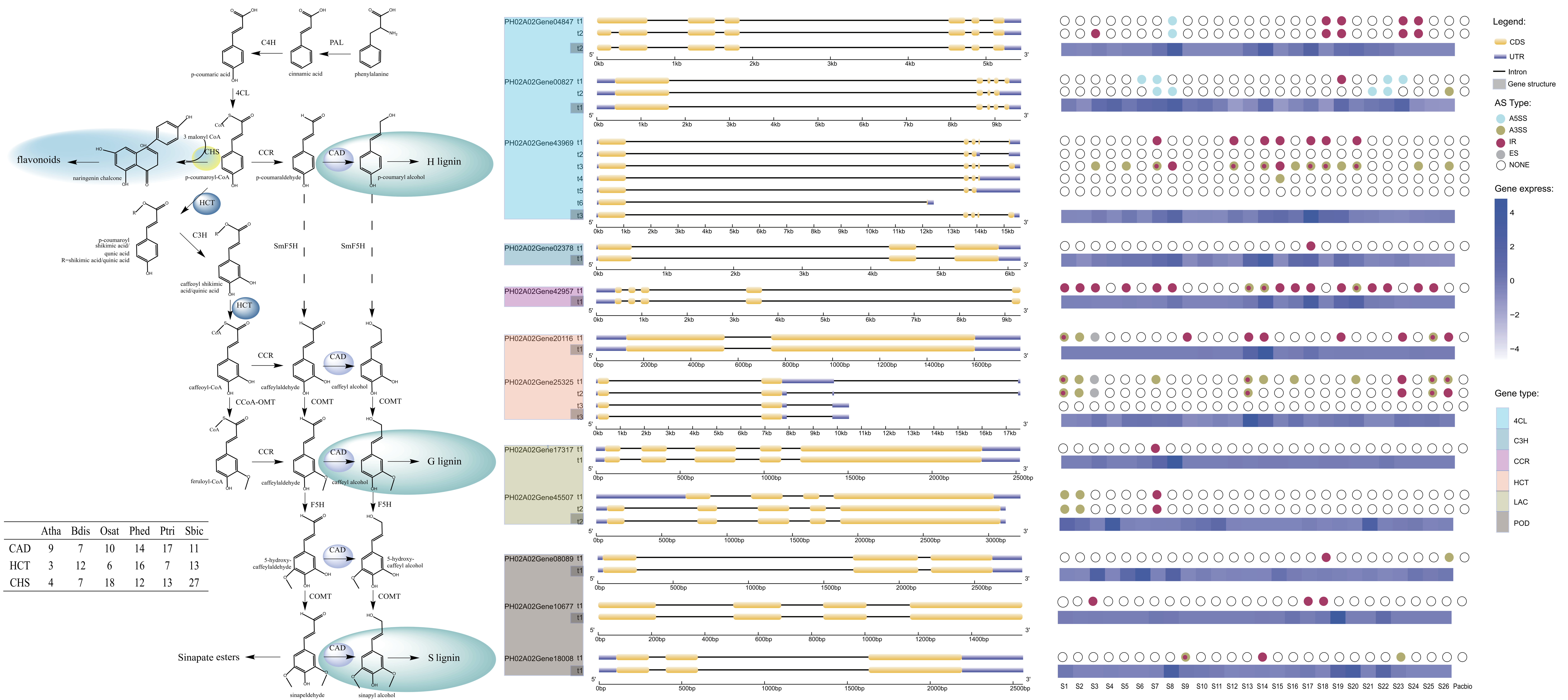

	Atha	Bdis	Osat	Phed	Ptri	Sbic
CAD	9	7	10	14	17	11
HCT	3	12	6	16	7	13
CHS	4	7	18	12	13	27

Additional File

Click here to access/download
Supplementary Material
Additional File-Revised1-419.docx

Additional Table S25

Click here to access/download
Supplementary Material
Additional Table 25.xls

Additional Table S28

Click here to access/download
Supplementary Material
Additional Table 28.xls
