# Supplementary material for: Chromosome-level reference genome and alternative splicing atlas of moso bamboo (Phyllostachys edulis)
Source: Gigascience. 2018 Sep 8;7(10):giy115. doi: 10.1093/gigascience/giy115 (PMC6204424; doi:10.1093/gigascience/giy115)
Supplement: GIGA-D-18-00076_Revision_2.pdf [file giy115_giga-d-18-00076_revision_2.pdf]

GigaScience

Chromosome-level reference genome and alternative splicing atlas of moso bamboo (Phyllostachys edulis)

--Manuscript Draft--

**Manuscript Number:**	GIGA-D-18-00076R2
**Full Title:**	Chromosome-level reference genome and alternative splicing atlas of moso bamboo (Phyllostachys edulis)
**Article Type:**	Research
**Funding Information:**	Special Fund for Forest Scientific Research in the Public Welfare from State Forestry Administration of China (201504106) — Prof. Hansheng Zhao
**Abstract:**	Background<br>Bamboo is one of the most important non-timber forest products worldwide. However, a chromosome-level reference genome is lacking, and the evolutionary aspect of alternative splicing (AS) in bamboo remains unclear despite emerging omics data and improved technologies.<br><br>Results<br>Here, we provide a chromosome-level de novo genome assembly of moso bamboo (Phyllostachys edulis) using additional abundance sequencing data and a HiC scaffolding strategy. The significantly improved genome is a scaffold N50 of 79.90 Mb, approximately 243 times longer than the previous version. A total of 51,074 high-quality protein-coding loci with intact structures were identified using single-molecule real-time sequencing and manual verification. Moreover, we provide a comprehensive AS profile based on the identification of 266,711 unique AS events in 25,225 AS genes by large-scale transcriptomic sequencing of 26 representative bamboo tissues using both the Illumina and PacBio sequencing platforms. Via comparison with orthologous genes in related plant species, we observed that the AS genes are concentrated in more conserved genes that tend to accumulate higher expressed transcripts and share less tissue specificity. Furthermore, gene family expansion, abundant AS and positive selection were identified in crucial genes involved in the lignin biosynthesis pathway of moso bamboo.<br><br>Conclusions<br>These fundamental studies provide useful information for future studies performing in-depth analyses of comparative genome and AS features. Additionally, our results highlight a global perspective of AS during evolution and diversification in bamboo.
**Corresponding Author:**	Hansheng Zhao<br>International Center for Bamboo and Rattan<br>Beijing, Beijing CHINA
**Corresponding Author Secondary Information:**	
**Corresponding Author's Institution:**	International Center for Bamboo and Rattan
**Corresponding Author's Secondary Institution:**	
**First Author:**	Hansheng Zhao
**First Author Secondary Information:**	
**Order of Authors:**	Hansheng Zhao
	Chunhai Chen
	Benhua Fei
	Songbo Wang
	Chengcheng Shi
	Jiongliang Wang

Powered by Editorial Manager® and ProduXion Manager® from Aries Systems Corporation

	XiaoChuan Liu
	Hailin Zhang
	Yongfeng Lou
	Lianfu Chen
	Huayu Sun
	Xianqiang Zhou
	Sining Wang
	Chi Zhang
	Hao Xu
	Lichao Li
	Yihong Yang
	Yanli Wei
	Wei Yang
	Qiang Gao
	Huanming Yang
	Zhimin Gao
	Shancen Zhao
	Zehui Jiang
**Order of Authors Secondary Information:**	
**Response to Reviewers:**	Scott Edmunds<br>Executive Editor<br>GigaScience<br><br>4 Jul 2018<br><br>Dear Dr. Scott,<br><br>Re: Manuscript reference No. GIGA-D-18-00076R1<br>Please find attached a latest revised version (Revision 2) of our manuscript "Chromosome-level reference genome and alternative splicing atlas of moso bamboo (Phyllostachys edulis)", which we would like to resubmit for publication as a research article in GigaScience.<br><br>The comments of the reviewers were highly insightful and enabled us to greatly improve the quality of our manuscript. In the following pages are our point-by-point responses to each of the comments and suggestions of the reviewer.<br><br>Revision 2 in the text is shown using red highlight based on Revision 1. We hope that these revisions in the manuscript and our accompanying responses will be sufficient to make our manuscript suitable for publication in GigaScience.<br><br>We shall look forward to hearing from you at your earliest convenience.<br><br>Yours sincerely,<br><br>Prof. Hansheng Zhao<br>Address: No. 8, Fu Tong Dong Da Jie, Chaoyang District, Beijing 100102, P.R. China<br>Tel: +86-010-8478 9804<br>Fax: +86-010-8478 9802<br>E-mail: zhaohansheng@icbr.ac.cn

Powered by Editorial Manager® and ProduXion Manager® from Aries Systems Corporation

Responses to comments of Reviewer #2

The authors have clarified some of the issues I raised, but I still have a few comments/suggestions/edits. Please also see the marked word document attached. Note the page and line numbers below are based on the attached word file.

1.Page 2, line 12. What does "uniform" mean here. You meant "unique"?

Response: Thank you for this excellent suggestion. According to your suggestion, we have revised the sentence, as follows:
“we provide a comprehensive AS profile based on the identification of 266,711 unique AS events in 25,225 AS genes by large-scale transcriptomic sequencing of 26 representative bamboo tissues using both the Illumina and PacBio sequencing platforms.”

2. Page 2, line 16. Please be specific about "specificity" here. You meant "tissue specificity"?

Response: Thank you for this excellent suggestion. Indeed, the description of “tissue specificity” was more proper and we have revised the sentence, as follows:
“Via comparison with orthologous genes in related plant species, we observed that the AS genes are concentrated in more conserved genes that tend to accumulate higher expressed transcripts and share less tissue specificity.”

3. Page 2, line 17. This sentence does not make sense. AS and positive selection on lignin biosynthesis do not indicate moso bamboo is a woody plant.

Response: Thank you for this excellent suggestion. We have removed the confused description, as follows:
“Furthermore, gene family expansion, abundant AS and positive selection were identified in crucial genes involved in the lignin biosynthesis pathway of moso bamboo.”

4. Page 4, line 6. What is "evolutionary landscape"?

Response: Thank you for this excellent suggestion. In the latest submission, we have used the description of “evolutionary aspect”, instead of “evolutionary landscape”, as follows:
“In conclusion, our analysis not only provides a global profile of AS in bamboo for further experimental studies investigating the functions of genes and regulatory networks but also reveals the roles of AS from the evolutionary aspect.”

5. Page 7, line 2. "the four main AS types" appears too suddenly here. You'd need to explain what these four types are first.

Response: Thank you for this excellent suggestion. We have transferred the related explanation to the first mentioned place of the four main AS types, as follows:
“In subsequent analyses, we defined the four main AS types as: intron retention (IR), alternative 3’ splice site donor (A3SS), alternative 5’ splice site acceptor (A5SS), and exon skipping (ES), and we also defined the other AS types represented some AS types except for the above four main AS types. Then, we found that on average, 80.37% of the AS events and 95.59% of the AS genes overlapped among the four main AS types (Additional Fig. S17).”

6. Page 7, line 5-6. This correlation was found among the four AS types? If so, you'd need to describe the four AS types first, and then say you found correlation of event

Powered by Editorial Manager® and ProduXion Manager® from Aries Systems Corporation

number and gene number among the four AS types.

Response: Thank you for this excellent suggestion. According to your suggestion, we have transferred the related explanation to the first mentioned place of the four main AS types and provided the correlation among the four main AS types, as follows:
“In subsequent analyses, we defined the four main AS types as: intron retention (IR), alternative 3’ splice site donor (A3SS), alternative 5’ splice site acceptor (A5SS), and exon skipping (ES), and we also defined the other AS types represented some AS types except for the above four main AS types.”
“The AS event number was strongly and positively correlated with the AS gene number and those among the four main AS types (R2>0.91, Mann-Whitney U test with p value <0.05) (Fig. 2c).”

7. Page 7, line 18. You have two "two-thirds" here, which does not make sense mathematically.

Response: Thank you very much for pointing out this error. We have revised the sentence, as follows:
“Since AS possess strong specificity to different tissues or developmental stages, we identified 181,105 tissue-specific AS events (67.57%), which account for two-thirds of the AS events (termed as among-tissue). Then, the remaining one-third of the AS events were detected based on comparisons of the transcript isoforms within individual tissues (termed as within-tissue) (Additional Fig. S18).”

8. Page 8, line 4. Why do species divergence estimates have anything to do with ortholog classification you described in the following?

Response: Thank you for this excellent suggestion. We had performed a genome-wide classification of orthologous genes in the 8 groups. These groups were identified based on the species tree (Fig. 3a). The species divergence time was used to exhibit the origination time of genes in different datasets. The Fig. 3a facilitated to vividly exhibit the relationship. Additionally, we have revised the related description and added the description of origination time, as follows:
“Based on the genome-wide identification of orthologous genes in the selected 8 plant species (Amborella trichopoda, A. thaliana, Elaeis guineensis, B. distachyon, O. sativa, Spirodela polyrhiza, S. bicolor and Ph. edulis) and the constructed phylogeny (Fig. 3a), we identified eight unique orthologous gene datasets (D8-D1) based on the origination times of genes in each dataset. For instance, unique orthologous gene dataset 7 (D7) only contained orthologous genes which originated between 164.9 million year ago (Mya) and 213.6 Mya. In addition, we also extracted single-copy genes respectively from above datasets and termed as D8s-D1s.”

9. Page 8, line 6. "which were located in an early divergence time in our constructed phylogeny" this statement does not make sense. You cannot "locate" in a "time" in a "phylogeny".

Response: Thank you for this excellent suggestion. We have revised the description, as follows:
“For instance, unique orthologous gene dataset 7 (D7) only contained orthologous genes which originated between 164.9 million year ago (Mya) and 213.6 Mya.”

10. Page 8, line 8. "According to a previous study [27], we obtained the divergence times of genes based on the presence and absence of orthologs in the phylogeny" please clarify this sentence. Presence/absence of orthologs cannot tell you the divergence of genes.

Response: Thank you for this excellent suggestion. According to your suggestion, we have removed the description.

Powered by Editorial Manager® and ProduXion Manager® from Aries Systems Corporation

11. Page 8, line 12. Please clarify "removing common genes in more than two gene datasets in eight original datasets and using single-copy genes in eight original datasets". I could not follow. What does "genes in more than two gene datasets" mean?

Response: Thank you for this excellent suggestion. We have revised the description, as follows:
“This trend was also observed in the single-copy datasets (D8s-D1s).”

12. Page 8, line 16. Did you do statistical test on all these four datasets?

Response: Thank you for this excellent suggestion. We have conducted a Chi square test on each corresponding orthologous group between D8-D1 and D8s-D1s (for example: D7 vs D7s), with p-value ranging from 0.86 to 0.98. Therefore, we concluded that the identical trends were detected in the two datasets. Additionally, we have revised the description, as follows:
“We investigated the distribution pattern of the four focal AS types in each dataset and found the identical trends (Fig. 3b), but the proportion of the AS types differed (IR>A3SS>A5SS>ES, Chi square test with p-value >0.86).”

13. Page 8, line 21-25. Again, what is the statistical result?

Response: Thank you for this excellent suggestion. We have conducted Pearson correlation between the median of maxTs and origination time in each group on D8-D1 (R2=0.863 and p value <0.01). Additionally, we have revised the description, as follows:
“Additionally, we compared with the AS events among the genes expressed in samples with different tissue specificities (maxTs) (for details, see Methods). The maxTs=1 and maxTs=0 represented constitutive expression and tissue specific expression, respectively. We found that the maxTs was negatively correlated with the origination time of the genes in D8-D1 (R2 > 0.86 and p value <0.01), representing an enhancement in the tissue specificity from a highly conserved gene dataset to a poorly conserved dataset (Fig. 3d).”

14. Page 8, line 25. "specificity" you meant "tissue specificity"?

Response: Thank you for this excellent suggestion. We have revised the sentence, as follows:
“Additionally, compared with the AS events among the genes expressed in samples with different specificities (maxTs) (for details, see Methods), the maxTs obviously increased from D8 to D1, representing an enhancement in the tissue specificity from a highly conserved gene dataset to a poorly conserved dataset (Fig. 3d).

15. Page9, line 11-16. A lot of this can go into the method.

Response: Thank you for this excellent suggestion. We have revised the part and transferred the related description to the Method, as follows:

Analysis:
“Additionally, the divergence time of the gene involved in the lignin biosynthesis pathway (Additional Fig. S22) occurred at the 5~16 Mya, which correspond to the whole genome duplication (WGD) time 7~12 Mya in the moso bamboo genome.”

Method:
“We calculated the synonymous substitution rate analysis for 13 gene families evolved in the lignin biosynthesis using the yn00, which was a package in PAML to estimate synonymous and nonsynonymous substitution rates. Then, the Ks rate was translated to the divergence time by the formula T=Ks/2r (r=6.5×10-9).”

Powered by Editorial Manager® and ProduXion Manager® from Aries Systems Corporation

16. Page 9 Discussion. Discussion in the current form is poorly organized. There are redundant points appearing in multiple paragraphs. Adding subheadings would help.

Response: Thank you for this excellent suggestion. According to your suggestion and the author instruction of GigaScience, we have removed redundant description and added the subheading. Please see the new revision for details due to many modifications.

17. Page 9, line 28. "High-throughput" is not appropriate to describe "assembly strategy". Also this sentence is written as like you developed new technologies, but I don't think so?

Response: Thank you for this excellent suggestion. We have revised the sentence, as follows:
“High-throughput genome sequencing and improved assembly strategy were broadly applied in current plant genomic studies with the development of new technologies and more useful data.”

18. Page 10, line 1-2. I still do not understand why TE could be "a driving force during the formation process of AS in bamboo". Where is the evidence?

Response: Thank you for this excellent suggestion. We have removed the description.

19. Page 11, "More AS events were identified in the sample with vigorous growth, which is consistent with the previous studies" - this was not mentioned in the Results.

Response: Thank you for this excellent suggestion. We have removed the description in discussion.

20. Page 11, "Obvious differences were observed in the AS event numbers in the final three shoot developmental stages, likely contributing to the fast growth during shoot development" - this was not mentioned in the Results. Also please provide statistical results to support "obvious".

Response: Thank you for this excellent suggestion. We have removed the description in discussion.

21. Page 11, line 23-24. I couldn't follow this sentence: "This finding was robust because we analyzed using the orthologous genes only in one dataset and using single-copy genes in selected species, respectively."

Response: Thank you for this excellent suggestion. We have revised the description, as follows:
“This finding was robust because we found the identical trends in the two types of eight gene datasets (D8-D1 and D8s-D1s).”

22. Page 11 line 27. I found the discussion on "new genes" is not well thought out. For example, the "hub genes" or "conserved genes" should have less functional diversity, not higher as the authors asserted here. I suggest drop this section.

Response: Thank you for this excellent suggestion. According to the references focusing on new genes, we have tried to revise the description, as follows:
“Previous reports have demonstrated that duplication is a major source of functional diversity and the generation of new genes [35], and conserved genes tend to have higher connectivity in gene-gene interaction networks, indicating their functional importance, while new genes were firstly added into gene-gene interaction networks with low connectivity and then gradually increased their connectivity and acquire pleiotropic roles [22,36]. In our study, highly conserved genes tended to have more AS

Powered by Editorial Manager® and ProduXion Manager® from Aries Systems Corporation

events than poorly ones, which was consistent with the trend that conserved genes were apt to have higher connectivity in gene-gene interaction networks. Thus, we proposed that the AS may be associated with the increase of gene connectivity during evolution."

23. Page 12, line 3-5. "Additionally, the four main AS types were abundant in the highly conserved gene datasets, and many other AS types appeared in the poorly conserved datasets. Thus, the four main AS types were conserved, and other types might represent an intermediate stage." I do not follow the logic here. Why AS of other types in the poorly conserved datasets would suggest they are "intermediate". I suggest remove this section.

Response: Thank you for this excellent suggestion. According to your suggestion, we have removed the section in discussion.

24. Page 12, line 14-18. "We hypothesize that the highly conserved genes with more AS events might be critical for evolution and function in generating gene functional diversity and the generation process of the highly conserved genes might undergo rigorous regulation during long-term evolution since the poorly conserved genes had less AS events than the highly conserved genes." This sentence is too long and complicated. Please rephrase.

Response: Thank you for this excellent suggestion. We have rewritten the section and removed the redundant point in discussion, as follows:
"Previous reports have demonstrated that duplication is a major source of functional diversity and the generation of new genes [35], and conserved genes tend to have higher connectivity in gene-gene interaction networks, indicating their functional importance, while new genes were firstly added into gene-gene interaction networks with low connectivity and then gradually increased their connectivity and acquire pleiotropic roles [22,36]. In our study, highly conserved genes tended to have more AS events than poorly ones, which was consistent with the trend that conserved genes were apt to have higher connectivity in gene-gene interaction networks. Thus, we proposed that the AS may be associated with the increases of gene connectivity during evolution."

25. Page 13, line 2-3. "During the evolutionary process, a new gene might be generated by duplication, which then forms less AS under strict constraints." Again this argument is flawed. A newly duplicated gene should have a "relaxed" functional constraint because a redundant copy is created.

Response: Thank you for this excellent suggestion. Indeed, the generation of a new genes was likely caused by either relaxation of functional constraint or positive Darwinian selection [1,2] and we have reorganized the section and removed the redundant point, as follows:
"Previous reports have demonstrated that duplication is a major source of functional diversity and the generation of new genes [35], and conserved genes tend to have higher connectivity in gene-gene interaction networks, indicating their functional importance, while new genes were firstly added into gene-gene interaction networks with low connectivity and then gradually increased their connectivity and acquire pleiotropic roles [22,36]. In our study, highly conserved genes tended to have more AS events than poorly ones, which was consistent with the trend that conserved genes were apt to have higher connectivity in gene-gene interaction networks. Thus, we proposed that the AS may be associated with the increases of gene connectivity during evolution."

26. Page 13, line 19-20. What is the rationale that more AS would indicate "a dominant position in the competition to bind p-coumaroyl CoA"?

Response: Thank you for this excellent suggestion. HCT generates lignin by catalyzing p-coumaroyl CoA, which is also catalyzed by CHS to generate flavonoids. Thus, HCT

and CHS compete with each other to bind p-coumaroyl CoA. In bamboo, the HCT family has more members and AS events than the CHS family as well as positive selection was detected in HCT family, which likely indicate that HCT family, compared to the CHS family, might be in a dominant position in the competition to bind p-coumaroyl CoA. Additionally, we have revised the description, as follows:
"In bamboo, the HCT family has more members and AS events than the CHS family, which likely indicate that the HCT family, compared to the CHS family, might be in a dominant position in the competition to bind p-coumaroyl CoA."

27. Page 14, line 12. What does "bamboo evolutionary landscape" mean?

Response: Thank you for this excellent suggestion. We have used the description of "evolutionary aspect", instead of "evolutionary landscape". Additionally, we have revised the description, as follows:
"In summary, these results will likely provide important resources for studies investigating bamboo's unique woodiness in the Grass family (Poaceae) and exploring AS from the bamboo evolutionary aspect."

28. Page 14, line 19. You meant "HuNan", not "HuHan" right?

Response: Thank you very much for pointing out this error. We have revised the sentence, as follows:
"(4) TaoJiang, HuNan Province (N:28°28′39.74″, E:112°11′18.62″, 320 M),"

29. Page 17, line 8. "identity > 95%"? Could you double check this threshold? Nucleotide identity > 95% is extremely stringent, and I cannot imagine you could get anything out.

Response: Thank you for this excellent suggestion. Indeed, the threshold was mistakes by double-checking our script and we have revised the description, as follows:
"Briefly, we performed standard protein BLAST searches (version 2.2.26) against the six genome sequences including moso bamboo using the coding sequence of the known genes with the following cut-off values: E-value <1e-10; identity > 40%; and coverage rate > 95% of query sequence."

30. Figure 2. Change "PacBio" to "Iso-Seq".

Response: Thank you for this excellent suggestion. According to your suggestion, we have revised the Figure 2.

31. Figure 3C. It is unclear to me what this panel is showing. The figure legend also did not help much.

Response: Thank you for this excellent suggestion. According to your suggestion, we have revised the Figure 3C.

32. Figure 3D. Explain the y-axis: what does "number" and "rate" refer to? Also the x-axis "Species" should be replaced by something like "Datasets" right?

Response: Thank you for this excellent suggestion. Number and Rate refer to AS number and maxTs. According to your suggestion, we have revised the Figure 3D.

Responses to the comments of Reviewer #3

Powered by Editorial Manager® and ProduXion Manager® from Aries Systems Corporation

Reviewer #3: The manuscript presents a comprehensive assembly of the bamboo genome which provides assembled chromosomes; an improvement from the current more fragmented assembly for the species. It also identifies alternative splicing events from transcriptome data corresponding to 26 different tissues.
I believe that the data presented will be of great use to the scientific community, in particular those working on genomics and those interested in transcriptomics and alternative splicing.

Main comments:
1. FOCUS AND JUSTIFICATION. However, I think that study could be better justified and given a focus. While the relevance of having a more complete genome for an important plant is well justified, it is not clear how this relates to alternative splicing. There is also no justification as to why examining alternative splicing is important. For example, in the abstract it is stated that the paper assembles the genome and identifies alternative splicing events but does not explain WHY has alternative splicing was an important aspect to explore in a paper presenting a more complete assembly of the bamboo genome. Thus, one has the impression that this paper contains two separate stories running side by side.
Perhaps one solution is to explain that gene duplication and alternative splicing are important drivers of functional evolution in genomes. That incomplete and fragmented scaffolds of genomes makes it difficult to assess patterns of gene duplication and that low coverage transcriptomes of only a handful of tissues does not allow to fully understand the extent of alternative splicing. Thus, having a fully assembled genome as well as an extensive RNA sequencing for recovering RNA isoforms is required. It is important to explain in detail WHY is alternative splicing important.

Response: Thank you for this excellent suggestion. According to your suggestion, we have added the information in the Background, as follows:

"The incomplete and scattered scaffolds of moso bamboo genome and the low coverage transcriptomes of a handful of tissues make it difficult to fully dissert the AS profiles. Therefore, a high-quality assembled genome and an extensive RNA sequencing are critical for the comprehensive AS identification."

2. JUSTIFICATION AND ORDER OF SPECIFIC ANALYSES. It is unclear to me why the description of the transposable element content should be in the section discussing alternative splicing events rather than on the section describing the genome sequence obtained.

Response: Thank you for this excellent suggestion. Due to the major modification, the description of the transposable element was removed in the Discussion.

3. DISCUSSION. Justification and relevance of many of the tests done is more evident in the discussion some of this information would be better placed in the introduction or as brief sentences in the results so that the analyses make sense as they are presented.

Response: Thank you for this excellent suggestion. According to your suggestion, we have majorly revised the Discussion. Please see the details in the latest revision.

More specific points
4. On the evolution of AS section specify how many orthologs were found when comparing between species and what percentage of the total number of genes in the bamboo this represents. Even if these numbers are shown in the figure/tables it is always helpful to get an idea of the patterns from the text alone.

Response: Thank you for this excellent suggestion. According to your suggestion, we have added the orthologs number in the main text, as follows:
"We considered the bamboo-specific genes (4,023 orthologous genes; termed as D1) are poorly conserved, whereas the genes present in all selected plant species (18,997 orthologous genes; termed as D8) are highly conserved."

5. On the evolution of AS section it is not clear what analysis was done to compare the ortholog genes in other species. If I understand correctly, the analyses tries to assess whether having an older ortholog is associated with higher or lower rates of alternative splicing in the bamboo genome? This needs to be better worded. It is also important to explain WHY would this pattern be interesting important to understand evolution of alternative splicing. There is also a reference to a "robust pattern", does this refer to past literature in other species? If so then this needs to be better explained.
If actual conservation or overall patterns of AS in other plants, rather than presence absence of ortholog genes, were compared to the bamboo then this needs to be better explained as at the moment it is very unclear.

Response: Thank you for this excellent suggestion. Your understanding of this part is correct. Indeed, we tried to assess whether having an older ortholog is associated with higher or lower rates of alternative splicing in the bamboo genome? We then found that more conserved genes had more AS genes in bamboo. We are sorry for confusions. According to your suggestion, we have greatly revised this part, as follows:
"Based on the genome-wide identification of orthologous genes in the selected 8 plant species (Amborella trichopoda, A. thaliana, Elaeis guineensis, B. distachyon, O. sativa, Spirodela polyrhiza, S. bicolor and Ph. edulis) and the constructed phylogeny (Fig. 3a), we identified eight unique orthologous gene datasets (D8-D1) based on the origination times of genes in each dataset. For instance, unique orthologous gene dataset 7 (D7) only contained orthologous genes which originated between 164.9 million year ago (Mya) and 213.6 Mya. In addition, we also extracted single-copy genes respectively from above datasets and termed as D8s-D1s. We considered the bamboo-specific genes (4,023 orthologous genes; termed as D1) are poorly conserved, whereas the genes present in all selected plant species (18,997 orthologous genes; termed as D8) are highly conserved. The degree of conservation decreased monotonically from D8 to D1. AS was detected in all the datasets, but the proportion of AS genes in each dataset gradually decreased from D8 to D1 (Mann-Whitney U test with p value <0.05). This trend was also observed in the single-copy datasets (D8s-D1s). Therefore, the result was robust that more conserved genes had more AS genes in bamboo."

6. WHY the authors examine proportions of AS events by type is not entirely clear. There is an extensive literature on the patterns of prevalence of AS types as well as the differences in their potential contribution to functional adaptation.

Response: Thank you for this excellent suggestion. The difference in the frequencies or proportions of the AS types may reflect differences in their pre-mRNA splicing and this analysis is common in most genome-wide identification of AS. Thus, we examined the proportion of AS types and provided the details of bamboo for comparative analysis. Additionally, we have revised the description, as follows:
"The difference in the frequencies or proportions of the AS types may reflect differences in their pre-mRNA splicing and this analysis is common in most genome-wide identification of AS. The distribution of the AS types depicted that IR occupied the dominant position, indicating that the importance of IR could be inferred from inspecting its prevalence throughout evolution in plants. Nevertheless, a higher percentage of IR (38.22%) and other AS types (total 28.18%) were observed in bamboo."

7. It is unclear what the correlations for CDS length and intron number, etc. involved. Is this to correlate these parameters among the bamboo with its ortholog in the other plants? It is also not explained WHY was this done.

Response: Thank you for this excellent suggestion. To obtain an overview of the landscape of AS and its relationships with gene features, and to evaluate the factors that influence AS, we perform correlations between AS distribution and some gene features (e.g. the gene length, CDS length, intron length, exon number, exon cassette length, and intron cassette length) in the datasets. Additionally, we have revised the description, as follows:
"To obtain an overview of the landscape of AS and its relationships with gene features

Powered by Editorial Manager® and ProduXion Manager® from Aries Systems Corporation

and to evaluate the factors that influence AS, we also examined the correlations between AS distribution and gene features in the datasets (Additional Fig. S21)."

8. In the last section of the results, the title implies that the EVOLUTION of gene families was assessed. However the text below does not give any details of how was this done or whether there has actually been an expansion and if so, with respect to WHAT species… It is also not explained WHY only 13 gene families were assessed.

Response: Thank you for this excellent suggestion. To better understand the identification of gene involved in the lignin biosynthetic pathway, we have revised the related method in the following. Additionally, according to the identification described previously [3], 13 gene families belong to the lignin biosynthesis pathway.

In the method
"Genome-wide identification of genes involved in the lignin biosynthetic pathway

The five genome sequences of A. thaliana (TAIR10), B. distachyon (v3.1), O. sativa (v7.0), Populus trichocarpa (JGI2.0.31), and S. bicolor (v3.1) were downloaded from the ENSEMBL database [4]. According to our literature-based investigations, 140 genes from the lignin biosynthetic pathway was experimentally validated from previous studies (Additional Table S28), and then, these known genes were collected and used as the query sequences for further identification. We identified lignin biosynthetic genes using a BLAST search and domain analysis as described in a previous article[5]. Briefly, we performed standard protein BLAST searches (version 2.2.26) against the six genome sequences including moso bamboo using the coding sequence of the known genes with the following cut-off values: E-value <1e-10; identity >40%; and coverage rate >95% query sequence. The filtered sequences were subsequently analyzed by hmmsearch (version 3.1b2) using the Pfam-A.hmm database (released 2017/03/31). Consequently, unclear sequences with incomplete domains were discarded after manual correction. Phylogenetic analyses were carried out following. We also calculated the synonymous substitution rate analysis for 13 gene families evolved in the lignin biosynthesis using the yn00, which was a package in PAML to estimate synonymous and nonsynonymous substitution rates. Then, the Ks rate was translated to the divergence time by the formula $T=Ks/2r$ ($r=6.5\times10^{-9}$)."

9. The discussion states that the paper presents evidence consistent with the role of TE in driving AS. The results only present a description of the rates of AS and the presence of TEs. If one drives the other then some analysis to link the two should be presented.

Response: Thank you for this excellent suggestion. According to your and other reviewer's suggestion, we have removed the description in Discussion.

10. I could not find in the results section a reference to the sample with more vigorous growth to have higher AS as it is stated in the introduction. Could this be made more prominent so that when reading the discussion this result can be easily found.

Response: Thank you for this excellent suggestion. Due to greatly modification of Discussion, we have removed the description.

11. I think a better explanation of what is a poorly conserved dataset and a highly conserved dataset means.

Response: Thank you for this excellent suggestion. According to your suggestion, we have greatly revised the part of "Evolutionary analysis of AS in moso bamboo" and provided an explanation about the poorly/highly conserved dataset, as follows:
"We considered the bamboo-specific genes (4,023 orthologous genes; termed as D1) are poorly conserved, whereas the genes present in all selected plant species (18,997 orthologous genes; termed as D8) are highly conserved. The degree of conservation decreased monotonically from D8 to D1."

Powered by Editorial Manager® and ProduXion Manager® from Aries Systems Corporation

	References:<br>1. Chen S, Zhang YE, Long M. New genes in Drosophila quickly become essential. Science. 2010;330:1682–5.<br>2. Long M, Betrán E, Thornton K, Wang W. The origin of new genes: glimpses from the young and old. Nature Reviews Genetics. Nature Publishing Group; 2003;4:865–75.<br>3. Vanholme R, Demedts B, Morreel K, Ralph J, Boerjan W. Lignin biosynthesis and structure. PLANT PHYSIOLOGY. American Society of Plant Biologists; 2010;153:895–905.<br>4. Kersey PJ, Allen JE, Allot A, Barba M, Boddu S, Bolt BJ, et al. Ensembl Genomes 2018: an integrated omics infrastructure for non-vertebrate species. Nucleic Acids Res. 2018;46:D802–8.<br>5. Fischer S, Brunk BP, Chen F, Gao X, Harb OS, Iodice JB, et al. Using OrthoMCL to assign proteins to OrthoMCL-DB groups or to cluster proteomes into new ortholog groups. Curr Protoc Bioinformatics. Hoboken, NJ, USA: John Wiley & Sons, Inc; 2011;Chapter 6:Unit6.12.1–19.<br>6. Zhang YE, Vibranovski MD, Landback P, Marais GAB, Long M. Chromosomal redistribution of male-biased genes in mammalian evolution with two bursts of gene gain on the X chromosome. Barton NH, editor. PLoS Biol. Public Library of Science; 2010;8:e1000494.
**Additional Information:**	
**Question**	**Response**
Are you submitting this manuscript to a special series or article collection?	No
**Experimental design and statistics**<br><br>Full details of the experimental design and statistical methods used should be given in the Methods section, as detailed in our Minimum Standards Reporting Checklist. Information essential to interpreting the data presented should be made available in the figure legends.<br><br>Have you included all the information requested in your manuscript?	Yes
**Resources**<br><br>A description of all resources used, including antibodies, cell lines, animals and software tools, with enough information to allow them to be uniquely identified, should be included in the Methods section. Authors are strongly encouraged to cite Research Resource Identifiers (RRIDs) for antibodies, model organisms and tools, where possible.	Yes

Powered by Editorial Manager® and ProduXion Manager® from Aries Systems Corporation

Have you included the information requested as detailed in our Minimum Standards Reporting Checklist?	
**Availability of data and materials**<br><br>All datasets and code on which the conclusions of the paper rely must be either included in your submission or deposited in publicly available repositories (where available and ethically appropriate), referencing such data using a unique identifier in the references and in the "Availability of Data and Materials" section of your manuscript.<br><br>Have you have met the above requirement as detailed in our Minimum Standards Reporting Checklist?	Yes

Powered by Editorial Manager® and ProduXion Manager® from Aries Systems Corporation

Manuscript

Click here to access/download;Manuscript;Manuscript_Moso_30Aug-

Click here to view linked References

Chromosome-level reference genome and alternative splicing atlas of moso bamboo (*Phyllostachys edulis*)

Hansheng Zhao[1#], Zhimin Gao[1#], Le Wang[2,3#], Jiongliang Wang[1], Songbo Wang[4], Benhua Fei[1], Chunhai Chen[2], Chengcheng Shi[5], Xiaochuan Liu[5], Hailin Zhang[2], Yongfeng Lou[1], LianFu Chen[1], Huayu Sun[1], Xianqiang Zhou[2], Sining Wang[1], Chi Zhang[2], Hao Xu[1], Lichao Li[1], Yihong Yang[1], Yanli Wei[2], Wei Yang[2], Qiang Gao[2], Huanming Yang[2], Shancen Zhao[4+] and Zehui Jiang[1+]

[1] State Forestry Administration Key Open Laboratory on the Science and Technology of Bamboo and Rattan, Institute of Gene Science for Bamboo and Rattan Resources, International Center for Bamboo and Rattan, Futongdong Rd, WangJing, Chaoyang District Beijing 100102, China;

[2] BGI Genomics, BGI-Shenzhen, Building NO.7, BGI Park, No. 21 Hongan 3rd Street, Yantian District, Shenzhen 518083, China;

[3] Department of Plant Sciences, University of California, Davis, One Shield Avenue, Davis, CA 95617, USA;

[4] BGI Institute of Applied Agriculture, BGI-Shenzhen, No.7 PengFei Rd, Dapeng District, Shenzhen 518120, China;

[5] BGI-Qingdao, No. 2877, Tuanjie Rd, Sino-German Ecopark, Qingdao, Shandong Province, 266555, China.

ORCID IDs: Hansheng Zhao: 0000-0002-5405-0375; Benhua Fei: 0000-0003-0764-2762; Huayu Sun: 0000-0002-6532-7018; Sining Wang: 0000-0002-3532-2777; Huanming Yang: 0000-0002-0858-3410; Zhimin Gao: 0000-0003-4464-7159; Shancen Zhao: 0000-0001-8779-6969; Zehui Jiang: 0000-0002-2696-5500.

[+] Co-corresponding author: zhaoshancen@genomics.cn and jiangzehui@icbr.ac.cn

[#] These authors contributed equally to this work.

Abstract

Background

Bamboo is one of the most important non-timber forest products worldwide. However, a chromosome-level reference genome is lacking, and an evolutionary view of alternative splicing (AS) in bamboo remains unclear despite emerging omics data and improved technologies.

Results

Here, we provide a chromosome-level *de novo* genome assembly of moso bamboo (*Phyllostachys edulis*) using additional abundance sequencing data and a Hi-C scaffolding strategy. The significantly improved genome is a scaffold N50 of 79.90 Megabase (Mb), approximately 243 times longer than the previous version. A total of 51,074 high-quality protein-coding loci with intact structures were identified using single-molecule real-time sequencing and manual verification. Moreover, we provide a comprehensive AS profile based on the identification of 266,711 unique AS events in 25,225 AS genes by large-scale transcriptomic sequencing of 26 representative bamboo tissues using both the Illumina and Pacific Biosciences sequencing platforms. Through comparisons with orthologous genes in related plant species, we observed that the AS genes are concentrated among more conserved genes that tend to accumulate higher expressed transcripts and share less tissue specificity. Furthermore, gene family expansion, abundant AS and positive selection were identified in crucial genes involved in the lignin biosynthetic pathway of moso bamboo.

Conclusions

These fundamental studies provide useful information for future in-depth analyses of comparative genome and AS features. Additionally, our results highlight a global perspective of AS during evolution and diversification in bamboo.

Keywords: Moso Bamboo, Genome, Annotation, Alternative Splicing, Transcriptome, Evolution

Background

Bamboo (Bambusoideae) is a fast-growing plant with substantial potential for generating income, restoring degraded landscapes and combating climate change in numerous Asian and African countries. Approximately 2.5 billion people economically depend on bamboo, reaching an annual international trade of over 2.5 billion US dollars [1]. Bamboo is a perennial grass in temperate and tropical forests worldwide, with a cellulose and hemicellulose content comparable to that of woody trees [2]. Moso bamboo (*Phyllostachys edulis*) accounts for ~73.76% of the bamboo-growing regions of China (4.43 million ha), constitutes the most abundant natural resource of non-wood products and plays significant roles in economics, ecology, culture, aesthetics and technology [3].

Only a limited number of genome-wide studies have been performed in bamboo. We first reported a draft genome of moso bamboo in 2013 and released 2.05 Gigabase (Gb) of the draft genome with 328 Kilobase (Kb) of Scaffold N50 and 31,987 predicted genes [4]. Due to advances in sequencing technology and analytical methods, a chromosome-level reference genome with improved precision and contiguity could facilitate functional and evolutionary analyses of bamboo.

Alternative splicing (AS) is a major mechanism underlying the increased complexity and diversity of proteins made from a limited number of genes in eukaryotes [5]. More than 95% of human multi-exon genes have been predicted to express multiple splice isoforms [6,7], and the occurrence of AS events in plants is reported to be ~61%, ~52%, ~42%, ~40%, ~40% and ~33% in *Arabidopsis thaliana* [8,9], *Glycine max* [10], *Brachypodium distachyon* [11], *Gossypium raiimondi* [12], *Zea mays* [13] and *Oryza sativa* [14], respectively. The different splicing products of a single gene represent major sources of functional plasticity and supposedly play important roles in plant growth, development, defense responses, signal transduction and flowering time [15-19]. Species-specific AS is partly responsible for generating a wide variety of functional diversity with limited repertoires of protein-coding genes [20-22]. However, the mechanism by which AS modulates plant evolution is unclear. Moreover, the AS characteristics of genes with differing degrees of conservation remains elusive.

The incomplete and scattered scaffolds of the moso bamboo genome and the low-coverage transcriptomes of a handful of tissues make it difficult to fully dissect AS profiles. Therefore, a high-quality assembled genome and extensive RNA sequencing are critical for comprehensive AS identification.

Thus, we substantially improved the moso bamboo genome assembly and gene annotation and also performed a comprehensive genome-wide analysis to uncover AS profiles in bamboo using transcriptome data from 26 mixed samples collected from six major bamboo-producing areas in China. These transcriptome data were generated using the Illumina and Pacific Biosciences (PacBio) platforms. Numerous AS genes and events were detected, and various types of AS events were identified. We performed a genome-wide investigation to determine the relationship between amino acid conservation and AS and examine the evolution of the AS status of genes that are involved in lignin biosynthesis. In conclusion, our analysis not only provides a global profile of AS in bamboo for further experimental studies investigating the functions of genes and regulatory networks but also reveals the roles of AS from an evolutionary perspective.

Data description

For the assembly of the moso bamboo genome, approximately ~603.3 Gb of genome data were generated using different sequencing strategies. Whole-genome sequence (WGS) assembly was performed using ~154 Gb of newly acquired and ~220 Gb of previously acquired clean data [4]. Hi-C assembly was performed using ~157 Gb of raw data from a Hi-C library, and ~17.58 Gb of valid reads were obtained after quality control (Additional Table S1). Additionally, for transcriptomic analysis, approximately ~379 Gb and ~5 Gb of raw data were produced from the Illumina and PacBio platforms, respectively (Additional Tables S2-7). Thus, we identified 266,711 unique AS events in 25,225 AS genes in moso bamboo according to the chromosome-level genome reference and the high-throughput transcriptome data.

Analyses

Chromosome-level genome assembly and gene annotation in moso bamboo

To enhance the quality of the moso bamboo genome, a total of 61 libraries were used and subjected to sequencing according to the instructions of the sequencer manufacturer (Additional Table S1). In total, we obtained ~603.3 Gb of genome data with read lengths ranging from 76 bp to 250 bp. Subsequently, we applied different assembly strategies to obtain a better genome assembly (see the Additional File for details). First, the WGS assembly reached 1.91 Gb with a contig and scaffold N50 length of 55 Kb and 894 Kb, respectively (Additional Table S8). Compared with our previous version [4], the assembly statistics

and quality of the new WGS assembly were clearly improved (Additional Tables S9-10). For example, the scaffold N50 and contig N50 lengths were increased by 172% and 358%, respectively, and the ambiguous base rate was decreased by 43%. Next, the Hi-C assembly was generated with a total length of 1.91 Gb and contig and scaffold N50 lengths of 53.29 Kb and 79.90 Mb (Fig. 1a and 1b). Approximately 93.17% of scaffolds from the WGS assembly were anchored onto 24 chromosomes (Additional Table S10) [23], and the scaffold N50 was increased by ~89-fold (Table 1). According to the contact map (Additional Fig. S1) and the assembly results, the boundaries between the 24 chromosomes were clearly observed. We then aligned the moso bamboo chromosomes to the rice genome and found a mean coverage of ~59.77% (Additional Fig. S2 and Additional Table S11). Additionally, we evaluated the chromosome-level assembly using bamboo-derived BAC sequences, full-length cDNAs (FL-cDNAs) [24] and some known genes (Additional Fig. S3 and Additional Tables S12-14). The chromosome-level assembly displayed more-extensive genome coverage, and the accuracy was higher than that of the first assembly.

The chromosome-level assembly generated here can facilitate gene prediction in subsequent analyses after annotation of repetitive sequences (Additional Table S15). Based on numerous transcriptomic data (Additional Table S16), full-length cDNAs [24], and homologous proteins, we predicted 51,074 high-quality protein-coding loci with intact structures in moso bamboo (Additional Table S17). Average intron and exon length were 668 bp and 284 bp, respectively (Fig. 1c and Additional Table S18). A combination of single-molecule real-time sequencing and manual verifications was carried out to confirm or correct certain irregular predictions. Approximately 17% of the gene models were improved by untranslated region (UTR) addition and internal structural adjustment (Additional Table S19). According to the completeness assessment of the annotation using Benchmarking Universal Single-Copy Orthologs (BUSCO) [25], moso bamboo (95.2%) was more complete than *Z. mays* (92.2%) but close to *O. sativa* (95.6%) (Fig. 1d and Additional Table S20). Compared with the previous annotation, 97.23% of the gene models in our analysis were identified in public databases, which facilitated the accurate detection of AS events (Additional Table S21). Detailed information regarding gene model prediction and genome evolution are presented in Additional Tables S22-24 and Figs. S3-9. Additionally, the latest genome assembly and gene annotation were released via the GigaScience *Giga*DB repository [26]. The entire dataset comprises the newly released bamboo genome sequence, gene sets, repeat elements, tRNAs, miRNAs, and gene clusters, providing a reliable resource for many analyses, including genomic, genetic,

and molecular biology experiments

Vast transcriptomic data generated using the Illumina and PacBio platforms

To facilitate the genome-wide investigation of AS profiles in moso bamboo and to comprehensively identify the factors influencing AS at the post-translational level, we performed high-throughput RNA sequencing (RNA-Seq) using the Illumina HiSeq-4000 platform. In total, 26 individual representative RNA samples were sequenced (150 bp of paired-ends; Additional Table S2 and Figs. S10-11). After preprocessing, we obtained an average of 90 million high-quality reads (~13.6 Gb) per sample, accounting for 92.78% of the raw reads. Approximately 80.57% of the high-quality reads were mapped to the reference genome at a unique position and designated as unique reads (Additional Tables S3-4). According to the alignment distribution, most sequences were mapped in exonic regions. The exon-mapping rate was on average 81.94%. The remaining reads were mapped in intronic regions (8.46%) and intergenic regions (9.6%) (Additional Table S5 and Figs. S12-13). The exonic coverage was found to be ~2,521× per sample (Additional Fig. S14). Therefore, these large-scale, in-depth, high-quality transcriptomic data, together with a high-quality reference genome, will likely contribute to accurate AS identification in moso bamboo.

To accurately identify full-length splice isoforms, we sequenced the bamboo transcriptome using the PacBio platform. FL-cDNA sequencing of alternatively spliced isoforms (Iso-Seq) used RNA from a mixture of 26 samples. According to the length distributions of the transcripts in all samples (Additional Table S6), we constructed 3 single-molecular real-time bell (SMRTbell^{TM}) libraries (1-2 kb, 2-3 kb, and >3 kb) for the mixed sample and sequenced 9 cells, generating ~5 Gb of raw data and 214,372 reads-of-insert (ROIs), including 133,599 full-length ROIs (containing a 5' primer, 3' primer and a poly(A) tail); the remaining ROIs were non-full-length ROIs (Additional Table S7 and Fig. S15). Accuracy evaluation based on aligning the ROIs against the new genome showed that the per-nucleotide error was approximately 2.05% and consisted of mismatches (0.32%), insertions (0.98%) and deletions (0.75%).

Numerous genes undergo AS in moso bamboo

Based on the improved reference genome and large-scale transcriptome data, we performed a genome-wide analysis to identify AS in moso bamboo using a previously described pipeline [10]. In total, 266,711 unique AS events were identified in 25,225 AS genes, accounting for *ca.* 49.39% of all annotated genes. Except for the 12,653 AS genes identified in the gene annotation, the rest (12,572 genes) were considered

novel AS genes (Additional Fig. S16).

The Iso-Seq data were also utilized to detect AS in an analysis parallel to the Illumina RNA-Seq analysis. In total, 4,246 AS events and 2,218 AS genes were identified (Fig. 2a, b). According to the PacBio-Illumina overlap analysis, which was performed to assess the validity of the AS prediction, 81.21% of the AS events and 97.34% of the AS genes identified in the Iso-Seq analysis completely overlapped with those in the RNA-Seq analysis. In subsequent analyses, we defined the four main AS types as intron retention (IR), alternative 3' splice site donor (A3SS), alternative 5' splice site acceptor (A5SS), and exon skipping (ES), and we also defined other AS types that were distinct from the above four main AS types. On average, 80.37% of the AS events and 95.59% of the AS genes overlapped among the four main AS types (Additional Fig. S17). The high degree of overlap between the PacBio and Illumina AS genes is a strong indicator of the validity of computationally predicted AS.

The AS event number was strongly and positively correlated with the AS gene number and those among the four main AS types ($R^2 > 0.91$, Mann-Whitney U test with p value < 0.05) (Fig. 2c). The four main AS types were detected in AS events in moso bamboo according to canonical splicing patterns (GT-AG, GC-AG, and AT-AC splice sites). As shown in Fig. 2b, IR (38.22%) represented the most abundant type of AS event, followed by A3SS (20.20%) and A5SS (10.48%). ES (2.92%) was the least prevalent type among the four main AS types.

Regarding the functional implications of AS genes, enrichment analysis showed that 885 genes, which were AS in all samples, were significantly enriched in RNA metabolic processing, mRNA processing, RNA processing and RNA splicing (Additional Table S25). Since AS shows strong tissue and developmental specificity, we identified 181,105 tissue-specific AS events (67.57%), which account for two-thirds of the AS events (termed among-tissue). The remaining one-third of AS events were then detected based on comparisons of transcript isoforms within individual tissues (termed within-tissue) (Additional Fig. S18).

Transposable element (TE) analysis showed that 26,366 genes have TE insertion, accounting for 51.62% of all genes, and the total length of TE insertion in genes was ~46 Mb. According to the different position of the TE-inserted intron, TE-introns were mainly concentrated in the front and rear of a gene (Additional Fig. S19). Additionally, the usage and distribution of splice sites revealed that GT-AG splice sites were the most abundant, corresponding to 97.31% of all AS events, followed by GC-AG (2.33%) and GT-AT

(0.32%) splice sites (Additional Fig. S20). In addition to canonical splice sites (GT-AG, GC-AG, and AT-AC), the remaining 2,406 splice sites were identified as non-canonical splice sites, which contained 2,373 GT-AT splice sites and 33 splice sites of other types.

Evolutionary analysis of AS in moso bamboo

Based on the genome-wide identification of orthologous genes in the selected 8 plant species (*Amborella trichopoda*, *A. thaliana*, *Elaeis guineensis*, *B. distachyon*, *O. sativa*, *Spirodela polyrhiza*, *S. bicolor* and *Ph. edulis*) and the constructed phylogeny (Figs. 3a and 3b), we identified eight unique orthologous gene datasets (D8-D1) based on the origination times of genes in each dataset. For instance, unique orthologous gene dataset 7 (D7) contained only orthologous genes that originated between 164.9 million years ago (Mya) and 213.6 Mya (Fig. 3a). In addition, we also extracted single-copy genes from the above datasets, termed D8s-D1s. We considered the bamboo-specific genes (4,023 orthologous genes; termed D1) to be poorly conserved, whereas the genes present in all selected plant species (18,997 orthologous genes; termed D8) are highly conserved. The degree of conservation decreased monotonically from D8 to D1. AS was detected in all the datasets, but the proportion of AS genes in each dataset gradually decreased from D8 to D1 (Mann-Whitney U test with p value < 0.05). This trend was also observed in the single-copy datasets (D8s-D1s). Therefore, more conserved genes clearly contained more AS genes in bamboo.

We investigated the distribution pattern of the four focal AS types in each dataset and found identical trends (Fig. 3c), but the proportion of AS types differed (IR>A3SS>A5SS>ES, Chi-square test with p value > 0.86). The proportion of IR in D8 was 57.76%, which was ~3.4-fold higher than that in D1 (16.95%). The ratio of the other AS types increased as the level of conservation decreased. In the two types of datasets, the number of AS events gradually decreased from D8 to D1 and from D8s to D1s (Fig. 3c). The most abundant AS events appeared in D8, and the least abundant AS events were detected in D1. Additionally, we compared the AS events among the genes expressed in samples with different tissue specificities (maxTs) (for details, see Methods), where maxTs=1 and maxTs=0 represent constitutive expression and tissue-specific expression, respectively. We found that maxTs was negatively correlated with the origination time of the genes in D8-D1 (R^2 > 0.86 and p value < 0.01), representing an enhancement in tissue specificity from the highly conserved gene dataset to the poorly conserved dataset (Fig. 3d). Altogether, the conserved genes tended to have more AS genes, more AS events and less tissue

specificity.

To obtain an overview of the AS perspective and its relationships with gene features, as well as to evaluate the factors that influence AS, we also examined the correlations between AS distribution and gene features in the datasets (Additional Fig. S21). All genes in the different datasets (D8-D1) were positively correlated ($R^2 > 0.90$ and p value < 0.05) with gene length, CDS size, intron size, and exon number and negatively correlated ($R^2 > 0.81$ and p value < 0.05) with exon cassette length and intron cassette length. Moreover, the distribution of the TE genes in the eight datasets was examined, and a substantially negative correlation was observed ($R^2 > 0.77$ and p value < 0.05), indicating that the more conserved genes had more TE insertions.

Expansion of gene families involved in lignin biosynthesis and implications for gene functional diversity

We systematically identified 13 gene families involved in the lignin biosynthetic pathway using genome sequences from *A. thaliana*, *B. distachyon*, *O. sativa*, *Ph. edulis*, *P. trichocarpa*, and *S. bicolor*. The expansion of most families was detected in bamboo (Additional Table S26). Each gene had multiple copies in the bamboo genome, and the total size of the gene families in the lignin biosynthetic pathway was the largest in bamboo, with an average of ~19 copies per family. The highest and lowest copy numbers were detected in the peroxidase gene family (77 genes) and *p*-coumarate 3-hydroxylase gene family (3 genes), respectively. Additionally, the divergence of genes involved in the lignin biosynthetic pathway (Additional Fig. S22) occurred at 5~16 Mya, which corresponds to a whole-genome duplication (WGD) event 7~12 Mya in the moso bamboo genome [4].

Moreover, we performed an AS analysis of the genes in the lignin biosynthetic pathway. In total, 10 of the 13 families had AS genes, accounting for more than half of the total, except for the ferulate 5-hydroxylase gene family, which had a low proportion, and the chalcone synthases (CHS) and caffeic acid *o*-methyltransferase gene families, in which AS genes were not detected. A high percentage (>75%) of AS events was observed in the 4-coumarate: CoA ligase, hydroxycinnamoyl transferase (HCT) and cinnamyl alcohol dehydrogenase (CAD) gene families. In addition, we tested for positive selection in the gene families involved in the lignin biosynthetic pathway using a branch-site model. Several genes in two gene families, e.g., *HCT* and *CAD*, exhibited positive selection. The information provided by the phylogenetic

relationship using the best model and log likelihood ratio (lnL) is provided in Additional Table S27.

Discussion

The first major update of the moso bamboo genome

High-throughput genome sequencing and improved assembly strategies are broadly applied in current plant genomic studies with the development of new technologies and more useful data. In 2013, our initial analysis of the *Ph. edulis* genome provided a genome-wide perspective on genome and gene structure, the history of WGD events, and functional genes in critical functional categories [4]. In the present study, we enhanced both the precision and contiguity of the *Ph. edulis* genome and updated its annotation, accurately positioning the bamboo genome from an evolutionary perspective by performing comparative studies involving different species. Additionally, various biological characteristics of bamboo were studied in great detail using knowledge obtained from the latest version. Therefore, the chromosome-level reference genome and refined annotation will pave the way for future genomic studies of bamboo and other related plant species.

AS is common and AS exhibits variation in different tissues of moso bamboo

We provided global AS profiles in bamboo based on a large amount of high-throughput data from RNA-Seq and Iso-Seq. These data enabled the accurate detection of transcripts with low expression levels and the acquisition of complete gene structure, particularly in the AS analysis. A series of AS analyses improved our understanding of AS in bamboo during post-transcriptional regulation, including the identification of AS genes and AS events, the distribution of AS types, the use of splice sites, the length distribution of alternative exons, etc. AS is considered a major mechanism responsible for creating diversity from a limited repertoire of genes. For example, by combining one exon of four alternatively spliced regions that contain 12, 48, 33, and 2 alternative exons each, it is possible to generate, at most, 38,016 protein isoforms (12×48×33×2) from the *Dscam* gene in *Drosophila* [27]. In bamboo, we identified 266,711 unique AS events and 25,225 genes in all samples, and on average, 15,971 AS events and 9,080 AS genes were detected in each sample. Thus, AS might be tissue specific, and the actual AS percentages in bamboo might be underestimated. More AS events, supported by transcripts with low expression levels, can be detected as the sequencing depth increases [28].

According to our observations, the rhizome tissue had more AS events than the root tissue in moso bamboo, which may be because the two tissues play different roles during bamboo development. Photoassimilates are unavailable during the rapid growth of the moso bamboo shoots since no leaves are growing [29], and thus, the large amounts of nutrients and energy in the shoot would have to come from the attached matured bamboo through underground rhizomes. Therefore, as a rhizomatous plant, the rhizome in moso bamboo plays a critical role in nutrient and energy transport, which might explain the higher number of AS events detected in the rhizome.

The characteristics of moso bamboo might be related to the proportion of AS types

Differences in the frequencies or proportions of AS types may reflect differences in pre-mRNA splicing, and this analysis is common in most genome-wide identifications of AS. Analyzing the distribution of AS types revealed that IR was predominant, and the importance of IR can be inferred based on its prevalence throughout evolution in plants. Nevertheless, higher percentages of IR (38.22%) and other AS types (total 28.18%) were observed in bamboo. These higher percentages may be due to the unique features of bamboo and/or the depth of the sequencing, which can be tested in future comparative analyses. Additionally, the distribution of the four main AS types is consistent with that in *Arabidopsis* [5,9,28], soybean [10], and maize [13]. However, the allocation in animals and yeast differs from that in plants. The most abundant AS event is ES, followed by AA and AD, while IR is the least common [30]. The discrepancies in the occurrence of AS models between plants and animals suggest differences between plants and animals in terms of genomic structure and the mechanism of splice site recognition [31]. In addition, the identification of splice sites in an individual gene may provide an essential resource for fully understanding AS and isoform construction [32,33]. With respect to their distribution, the main AS types (e.g., GT-AG, GC-AG, and AT-AC) were consistent with those observed previously in animals and other plants [19].

Highly conserved genes with more AS events might play critical roles in evolution and function

We examined the relationship between AS and evolution via comparative genome analysis. To date, the relationship between gene conservation and AS remains unknown. To explore this issue, we performed a genome-wide analysis to examine AS in two types of eight orthologous gene datasets (D8-D1 and D8s-D1s) with different degrees of conservation. AS genes were more likely to be enriched in the highly conserved gene datasets, and these AS genes had more AS events. This finding was robust because we

found identical trends in both types of datasets. Previous reports have demonstrated that duplication is a major source of functional diversity and the generation of new genes [34], and conserved genes tend to have higher connectivity in gene-gene interaction networks, indicating their functional importance, while new genes are initially added into gene-gene interaction networks with low connectivity and then gradually increase their connectivity and acquire pleiotropic roles [22,35]. In our study, highly conserved genes tended to have more AS events than poorly conserved genes, which was consistent with the trend that conserved genes are apt to have higher connectivity in gene-gene interaction networks. Thus, we proposed that AS may be associated with increases in gene connectivity during evolution. Additionally, compared with the poorly conserved gene datasets, the highly conserved AS gene datasets had a low tissue-specific expression profile, indicating that these genes might be critical in fundamental functions, such as having higher connectivity in gene-gene networks. Therefore, we suggested that functionally important genes are generated by more frequent AS events. As an essential biological process, AS plays a crucial role in acquiring more functions, which might explain why highly conserved AS genes possess more AS events. We hypothesize that this phenomenon likely applies not only to bamboo but also to other plants or even animals.

AS and the expansion of gene families in the lignin biosynthetic pathway in moso bamboo might be related to WGD

Lignin represents a class of complex aromatic heteropolymers of monolignols that encrusts and interacts with the cellulose/hemicellulose matrix of the secondary cell wall [36]. Lignin accounts for up to ~25% of the total dry weight in bamboo [2]. We performed a thorough examination of the lignin biosynthetic pathway by combining AS and evolutionary analyses. The expansion of gene families in the lignin biosynthetic pathway was detected in bamboo. Combined with the results of the divergence times of lignin biosynthesis genes and our previous study [4], we estimated the occurrence of a putative WGD event at 7~12 Mya in the moso bamboo genome, suggesting a potentially tetraploidization event some time during bamboo evolution [4]. The ancient tetraploid then evolved into the current diploid moso bamboo. Additionally, WGD can provide more gene copies, facilitating the evolution of genes with new functions [37]. Therefore, the expansion of lignin biosynthetic genes in moso bamboo may be due to the occurrence of a WGD event. Additionally, two gene families (e.g., *HCT* and *CAD*) underwent more AS events and

positive selection. HCT generates lignin from *p*-coumaroyl CoA [38], which is also used by CHS to generate flavonoids. HCT and CHS compete with each other to bind *p*-coumaroyl CoA. In bamboo, the *HCT* family has more members and AS events than the *CHS* family, likely indicating that the *HCT* family might be in a dominant position to compete for *p*-coumaroyl CoA binding compared with the *CHS* family. CAD catalyzes many different substrates to generate different types of lignin. The aromatic lignin polymers commonly found in bamboo are composed of three monolignols, namely, *p*-hydroxyphenyl (H), vanillin (G), and syringaldehyde (S). Previous studies have shown the abundance of G and S lignin and a small amount of H lignin in bamboo [2]. The expansion of the *CAD* family in bamboo and the corresponding positive selection may explain the different substrates preferences that generate different proportions of monolignols in bamboo. The abundance of AS events, gene expansion, and positive selection were all consistent with the remarkable adaptability of bamboo in producing lignin.

Conclusions

To deeply explore the AS profile from an evolutionary perspective in bamboo, we improved the reference genome and refined the annotation of moso bamboo. Based on the chromosome-level genome sequence and the abundant transcriptomic data from multiple tissues from six main bamboo-producing areas in China, we provided a comprehensive analysis of AS in moso bamboo, identifying 266,711 unique AS events in 25,225 AS genes using both Illumina and PacBio sequencing platforms. Moreover, the integrated analysis of the AS results in bamboo, as well as the comparative analysis among eight representative plant species, showed that more conserved genes tended to accumulate higher transcript levels and exhibit less tissue specificity. Finally, by studying lignin biosynthesis from an AS and evolutionary standpoint, we observed several characteristics of crucial genes related to lignin biosynthesis in moso bamboo, including gene family expansion, abundant AS and positive selection. In summary, these results will likely provide important resources for studies investigating bamboo's unique woodiness in the Grass family (Poaceae) and for exploring AS in bamboo from an evolutionary perspective.

Method

Plant material collection

To obtain a comprehensive AS profile, moso bamboo (*Phyllostachys edulis*) samples used in these experiments were collected from six major bamboo-producing areas in China during the Spring of 2105, including (1) YiXing, JiangSu Province (N:31°15′08.41″, E:119°43′42.55″, 212 M), (2) TianMu Mountain, ZheJiang Province (N:30°19′13.42″, E:119°26′55.21″, 480 M), (3) XianNing, HuBei Province (N:29°81′10.02″, E:114°31′21.12″ 150 M), (4) TaoJiang, HuNan Province (N:28°28′39.74″, E:112°11′18.62″, 320 M), (5) GuiLin, GuangXi Province (N:28°28′39.74″, E:112°11′18.62″, 216 M), and (6) ChiShui, GuiZhou Province (N:28°28′15.27″, E:105°59′41.43″, 120 M). Twenty-six tissues were collected, including the rhizome, root, shoot, leaf, sheath, and bud, during different developmental stages. Each sample was a mixed sample collected from the above six major bamboo-producing areas. Detailed information regarding the biological samples is provided in Additional Table S19.

Genome sequencing, assembly and annotation

We assembled the moso bamboo genome using WGS and Hi-C strategies, and annotated the new genome sequence as described in a previous study [39] and Additional Files. Detailed descriptions of this section are also provided in Protocols.io [40].

Hi-C library preparation, sequencing and assembling

The construction of Hi-C library was prepared as previously described [39] and the detailed descriptions were presented in Additional Files.

RNA isolation and Illumina RNA-Seq library construction

We used standard methods of RNA isolation, purity, concentration, reverse transcription, and cDNA library construction, as described in a previous study [41]. All cDNA libraries were constructed and normalized as described in the Additional File.

RNA-Seq using the Illumina platform

After quality control, the pooled libraries were optically examined using an Illumina Cluster Station and were then sequenced on the Illumina HiSeq-4000 platform (150 bp of PE) according to the manufacturer’s protocols. Finally, the quality of the reads was evaluated, and the low-quality reads were filtered using FastQC (version 0.11.3)(FastQC, RRID:SCR_014583) [42] with the default parameters. The statistics of

the key metrics applied to the RNA-Seq data were calculated using RNA-SeQC (version1.1.8) [43] with the default parameter.

RNA-Seq data analysis

Detailed description of this section was provided in Protocols.io [44].Briefly, adaptor sequences and low-quality sequences were trimmed using Trimmomatic (version 0.33) (Trimmomatic, RRID:SCR_011848) [45] during the preprocessing of RNA-Seq data. Then, cleaned data were mapped to the improved genome using HISAT2 (version 2.0.2) (HiSat2, RRID:SCR_015530) [46] with the following modifications from the default parameters: maximum intron length (4,000); specify strand-specific information (RF); and minimum score (L, -0.1, -0.1); report alignments tailored to transcript assemblers were allowed. The empirical transcripts in each sample were obtained using Cufflinks (version 2.2.1) (Cufflinks , RRID:SCR_014597) [47] after the reads were aligned. The default parameters were used, except for the following parameters: the minimum isoform fraction (0.05); the small anchor fraction of the spliced reads (0.05); the minimum intron length (20); the maximum intron length (4,000); the library type (fr-firststrand); the corrected frag bias; and the corrected multi-read. AStalavista (version 4.0) (AStalavista, RRID:SCR_001815) [48,49] was used with the default parameters to identify the AS genes and events after the different assembled transcript isoforms were mapped to the corresponding gene model using Cuffcompare, which is a component of the Cufflink program. The main four AS types, *i.e.,* IR, A3SS, A5SS, and ES, were analyzed and compared. In addition, an enrichment analysis of the different genes was conducted using Ontologizer (version 2.0) [50] with annotations from the Gene Ontology (GO) database (GO, RRID:SCR_002811) [51]. We also calculated the tissue specificity (Ts) values in each sample and each gene based on the expression level (the total number of fragments per kilobase of sequence per million reads mapped). A detailed description is provided in a previous report [52]. Briefly, Ts is defined as the fractional expression of a gene in one sample tissue relative to the sum of its expression in all samples. Thus, the maximum Ts value (maxTs) of a gene serves as an indicator of the tissue specificity. Higher tissue specificity values represent more tissue-specific expression [53].

Construction and sequencing of the Iso-Seq library

The construction of Iso-Seq library and sequencing were performed based on the PacBio manufacturer's protocol as previously described [54]. According to the length distribution of the transcripts predicted by

bioinformatics (Additional Table S22), three SMRTBell libraries (1-2 kb of 3 cells, 2-3 kb of 2 cells, and >3 kb of 4 cells) were size-selected and a total of 9 SMRT cells were sequenced on the PacBio platform.

Iso-Seq data analysis

The sequencing data produced using PacBio RS II were processed to obtain consensus full-length isoforms. The isoforms from the multiple libraries were merged, and redundancy was removed to obtain the final consensus isoforms after processing the reads of the insert, classifying, and clustering. The assembled transcripts were mapped to the reference genome using PASA (version 2.0.2) (PASA, RRID:SCR_014656) [55] with the default parameters. Then, similar to the short-read data, the output file of the gtf was analyzed using AStalavista with the default parameters to identify the AS.

Evolutionary analysis

We identified gene families, constructed a phylogenetic tree, predicted divergence times as a previously study [4] and the detailed information was provided in Additional Files and Protocols.io [56].

Genome-wide identification of genes involved in the lignin biosynthetic pathway

The five genome sequences of *A. thaliana* (TAIR10), *B. distachyon* (v3.1), *O. sativa* (v7.0), *Populus trichocarpa* (JGI2.0.31), and *S. bicolor* (v3.1) were downloaded from the ENSEMBL database (Ensembl , RRID:SCR_002344) [57]. According to wide literature-based investigations, 140 genes from the lignin biosynthetic pathway were collected based on experimental validation in previous studies (Additional Table S28); then, these known genes were used as query sequences for further gene identification. We identified lignin biosynthetic genes using a BLAST search (NCBI BLAST , RRID:SCR_004870) and domain analysis as described previously [41,58]. Briefly, we performed standard protein BLAST searches (version 2.2.26) against the six genome sequences including moso bamboo using the coding sequences of known genes with the following cutoff values: E-value <1e-10; identity >40%; and coverage rate >95% of query sequence. The filtered sequences were subsequently analyzed by hmmsearch (version 3.1b2) using the Pfam-A.hmm database (released 31 Mar. 2017), and unclear sequences with incomplete domains were discarded after manual correction. Phylogenetic analyses were carried out following [4]. We also calculated the synonymous substitution rate analysis for 13 gene families evolved in the lignin biosynthesis

using the yn00, which was a package in PAML to estimate synonymous and nonsynonymous substitution rates. Then, the Ks rate was translated to divergence times by the formula T=Ks/2r ($r=6.5\times10^{-9}$).

Positive selection analysis

We performed a positive selection analysis based on the coding sequences of lignin biosynthetic pathway genes. In each family, protein sequences were first aligned by PROBCONS (version 1.12)(ProbCons, RRID:SCR_011813) [59] using the default parameters, except for the option of iterative refinement, for which we used 1,000 iterations. Then, we back-translate the protein alignment to its corresponding coding sequences. After obtaining the conserved blocks from the sequence alignment using Gblocks (version 0.91b) [60], jModelTest (version 2.1.6) [61] was used to find the best model according to the Bayesian Information Criterion. Subsequently, PhyML (version 3.0)(PhyML, RRID:SCR_014629) [62] was used to reconstruct the phylogenetic tree under the best model, with bootstrapping of 1,000 replicates. Finally, certain branches selected from the phylogenetic tree were examined in a positive selection analysis using PAML (version 4.8) [63] with a branch-site model. See more detailed protocols in protocols.io [64].

Availability of data and materials

Short-read sequencing data from this whole-genome shotgun project can be deposited at European Molecular Biology Laboratory (EMBL) under the accession ERP001340. RNA-Seq raw sequence data and Iso-Seq raw sequence data for a mixture sample were deposited in National Center for Biotechnology Information (NCBI) Short Read Archive (SRA) database under the accession numbers: SRX2408703-28 and SRR7032261-69, respectively. The chromosome-level genome and the latest annotation were provided in *Giga*DB [26]. Additionally, protocols to the methods are uploaded to Protocols.io [40,44,56,58,64].

Abbreviations

A3SS: alternative 3' splice site donor; A5SS: alternative 5' splice site acceptor; AS: alternative splicing; BUSCO: Benchmarking Universal Single-Copy Orthologs; CAD: cinnamyl alcohol dehydrogenase; CHS: chalcone synthases; EMBL: European Molecular Biology Laboratory; ES: exon skipping; FL-cDNAs: full-length cDNAs; Gb: Gigabase; GO: Gene Ontology; HCT: hydroxycinnamoyl transferase; IR: intron retention; Iso-Seq: FL-cDNA sequencing of alternatively spliced isoforms; Kb: kilobase; lnL: log

likelihood ratio; Mb: Megabase; Mya: Million years ago; PacBio: Pacific Biosciences; PE: paired-end; RNA-Seq: RNA sequencing; ROIs: reads-of-inserts; SMRT: Single-Molecule Real-Time; SRA: Short Read Archive; TE: Transposable Element; Ts: tissue specificity; UTR: Untranslated region; WGD: Whole Genome Duplication; WGS: Whole Genome Sequencing.

Declarations

Author's Contribution

Experimental design: H.Z., Z.G., L.W., C.C., B.F. S.W., Z.C., H.Y. and Z.J. Experimental preformation: H.Z., J.W.; H.Z., L.C., Z.X., C.Z. and Y.W. Data analysis: W.Y., H.S., L.L., S.W., Y.Y., Y.L., Q.G., C.C., X.C. and H.X. The providing of reagents, materials and analysis tools: H.Z. and Z.G. Article writing: H.Z., Z.C., Z.G. and B.F. All of the authors read and approved the final manuscript.

Competing interests

The authors declare that they have no competing interests.

Acknowledgements

This work received financial support from the Special Fund for Forest Scientific Research in the Public Welfare from State Forestry Administration of China (No. 201504106), and the Sub-Project of National Science and Technology Support Plan of the Twelfth Five-Year in China (No. 2015BAD04B03 and No. 2015BAD04B01). Additionally, we have appreciated the careful review and constructive suggestions from the editor as well as the three reviewers. These suggestions were highly insightful and enabled us to greatly improve the quality of our manuscript.

References

1. Zhao H, Zhao S, International Network for Bamboo and Rattan, Fei B, Liu H, Yang H, et al. Announcing the Genome Atlas of Bamboo and Rattan (GABR) project: promoting research in evolution and in economically and ecologically beneficial plants. GigaScience. 2017;6:1–7.

2. Bai Y, Xiao L, Shi Z, Sun R. Structural variation of bamboo lignin before and after ethanol organosolv pretreatment. International Journal of Molecular Sciences. 2013;14:21394–413.

3. Jiang Z. Bamboo and Rattan in the World. Beijing: China Forestry Publishing House, 2007.

4. Peng Z, Lu Y, Li L, Zhao Q, Feng Q, Gao Z, et al. The draft genome of the fast-growing non-timber forest species moso bamboo (*Phyllostachys heterocycla*). Nature Genetics. 2013;45:456–61.

5. Filichkin SA, Priest HD, Givan SA, Shen R, Bryant DW, Fox SE, et al. Genome-wide mapping of alternative splicing in *Arabidopsis thaliana*. Genome research. 2010;20:45–58.

6. Pan Q, Shai O, Lee LJ, Frey BJ, Blencowe BJ. Deep surveying of alternative splicing complexity in the human transcriptome by high-throughput sequencing. Nature Genetics. 2008;40:1413–5.

7. Wang ET, Sandberg R, Luo S, Khrebtukova I, Zhang L, Mayr C, et al. Alternative isoform regulation in human tissue transcriptomes. Nature. 2008;456:470–6.

8. Zhang PG, Huang SZ, Pin A-L, Adams KL. Extensive divergence in alternative splicing patterns after gene and genome duplication during the evolutionary history of Arabidopsis. Molecular Biology and Evolution. 2010;27:1686–97.

9. Marquez Y, Brown JWS, Simpson C, Barta A, Kalyna M. Transcriptome survey reveals increased complexity of the alternative splicing landscape in Arabidopsis. Genome research. 2012;22:1184–95.

10. Shen Y, Zhou Z, Wang Z, Li W, Fang C, Wu M, et al. Global dissection of alternative splicing in paleopolyploid soybean. Plant Cell. 2014;26:996–1008.

11. Mandadi KK, Scholthof K-BG. Genome-wide analysis of alternative splicing landscapes modulated during plant-virus interactions in *Brachypodium distachyon*. Plant Cell. 2015;27:71–85.

12. Li Q, Xiao G, Zhu Y-X. Single-nucleotide resolution mapping of the Gossypium raimondii transcriptome reveals a new mechanism for alternative splicing of introns. Molecular Plant. 2014;7:829–40.

13. Thatcher SR, Zhou W, Leonard A, Wang B-B, Beatty M, Zastrow-Hayes G, et al. Genome-wide analysis of alternative splicing in *Zea mays*: landscape and genetic regulation. Plant Cell. 2014;26:3472–87.

14. Zhang G, Guo G, Hu X, Zhang Y, Li Q, Li R, et al. Deep RNA sequencing at single base-pair resolution reveals high complexity of the rice transcriptome. Genome research. 2010;20:646–54.

15. Rühl C, Stauffer E, Kahles A, Wagner G, Drechsel G, Rätsch G, et al. Polypyrimidine tract binding protein homologs from Arabidopsis are key regulators of alternative splicing with implications in fundamental developmental processes. Plant Cell. 2012;24:4360–75.

16. Staiger D, Brown JWS. Alternative splicing at the intersection of biological timing, development, and stress responses. Plant Cell. 2013;25:3640–56.

17. Li W, Lin W, Ray P, Lan P, Schmidt W. Genome-wide detection of condition-sensitive alternative splicing in Arabidopsis roots. Plant Physiology. 2013;162:1750–63.

18. Cui P, Zhang S, Ding F, Ali S, Xiong L. Dynamic regulation of genome-wide pre-mRNA splicing and stress tolerance by the Sm-like protein LSm5 in Arabidopsis. Genome Biology. 2014;15:R1.

19. Reddy ASN. Alternative splicing of pre-messenger RNAs in plants in the genomic era. Annual Review Plant Biology. 2007;58:267–94.

20. Barbosa-Morais NL, Irimia M, Pan Q, Xiong HY, Gueroussov S, Lee LJ, et al. The Evolutionary Landscape of Alternative Splicing in Vertebrate Species. Science. 2012;338:1587–93.

21. Keren H, Lev-Maor G, Ast G. Alternative splicing and evolution: diversification, exon definition and function. Nature Reviews Genetics. 2010;11:345–55.

22. Roy SW, Irimia M. Splicing in the eukaryotic ancestor: form, function and dysfunction. Trends in Ecology Evolution. 2009;24:447–55.

23. Chen RY, Li XL, Song WQ, Liang GL, Zhang PX, Lin RS, et al. Chromosome atlas of major economic plants genome in China. Tomus 4. Chromosome atlas of various bamboo species. Beijing: Science Press xxx, 646p.-illus.. ISBN 7030108353 Ch, En Chromosome numbers. Geog= 0 Systematics: ANGIOSPERMAE (GRAMINEAE)(KR, 200303867), 2003.

24. Peng Z, Lu T, Li L, Liu X, Gao Z, Hu T, et al. Genome-wide characterization of the biggest grass, bamboo, based on 10,608 putative full-length cDNA sequences. BMC Plant Biology. 2010;10:116.

25. Simão FA, Waterhouse RM, Ioannidis P, Kriventseva EV, Zdobnov EM. BUSCO: assessing genome assembly and annotation completeness with single-copy orthologs. Bioinformatics. 2015;31:3210–2.

26. Zhao H; Chen C; Fei B; Wang S; Shi C; Liu X; Zhang H; Lou Y; Chen L; Sun H; Zhou X; Wang S; Zhang C; Xu H; Li L; Yang Y; Wei Y; Yang W; Gao Q; Yang H; Gao Z; Zhao S; Jiang Z (2018): Supporting data for "Chromosome-level reference genome and alternative splicing atlas of moso bamboo (*Phyllostachys edulis*)" GigaScience Database. http://dx.doi.org/10.5524/100498.

27. Celotto AM, Graveley BR. Alternative splicing of the *Drosophila Dscam* pre-mRNA is both temporally and spatially regulated. Genetics. 2001;159:599–608.

28. Wang B-B, Brendel V. Genomewide comparative analysis of alternative splicing in plants. Proceedings of the National Academy of Sciences. 2006;103:7175–80.

29. Song X, Peng C, Zhou G, Gu H, Li Q, Zhang C. Dynamic allocation and transfer of non-structural carbohydrates, a possible mechanism for the explosive growth of Moso bamboo (*Phyllostachys heterocycla*). Scientific Reports. 2016;6.

30. Kim E, Magen A, Ast G. Different levels of alternative splicing among eukaryotes. Nucleic Acids Research. 2007;35:125–31.

31. Nakai K, Sakamoto H. Construction of a novel database containing aberrant splicing mutations of mammalian genes. Gene. 1994;141:171–7.

32. Li Y, Li-Byarlay H, Burns P, Borodovsky M, Robinson GE, Ma J. TrueSight: a new algorithm for splice junction detection using RNA-seq. Nucleic Acids Research. 2013;41:e51–1.

33. Nilsen TW, Graveley BR. Expansion of the eukaryotic proteome by alternative splicing. Nature. 2010;463:457–63.

34. Flagel LE, Wendel JF. Gene duplication and evolutionary novelty in plants. New Phytologist. 2009;183:557–64.

35. Zhang W, Landback P, Gschwend AR, Shen B, Long M. New genes drive the evolution of gene interaction networks in the human and mouse genomes. Genome Biology. 2015;16:202.

36. Martone PT, Estevez JM, Lu F, Ruel K, Denny MW, Somerville C, et al. Discovery of lignin in seaweed reveals convergent evolution of cell-wall architecture. Current Biology. 2009;19:169–75.

37. Taylor JS, Raes J. Duplication and divergence: the evolution of new genes and old ideas. Annual Review Genetics. 2004;38:615–43.

38. Li X, Bonawitz ND, Weng J-K, Chapple C. The growth reduction associated with repressed lignin biosynthesis in *Arabidopsis thaliana* is independent of flavonoids. Plant Cell. 2010;22:1620–32.

39. Dudchenko O, Batra SS, Omer AD, Nyquist SK, Hoeger M, Durand NC, et al. *De novo* assembly of the *Aedes aegypti* genome using Hi-C yields chromosome-length scaffolds. Science. 2017;356:92–5.

40. Zhao H. ALLPATHS-LG Genome Assembly and HI-C mapping strategy. protocols.io. 2018. dx.doi.org/10.17504/protocols.io.phmdj46

41. Zhao H, Wang S, Wang J, Chen C, Hao S, Chen L, et al. The chromosome-level genome assemblies of two rattans (*Calamus simplicifolius* and *Daemonorops jenkinsiana*). Gigascience. 2018 Aug 7. doi: 10.1093/gigascience/giy097.

42. FastQC. http://www.bioinformatics.babraham.ac.uk/projects/fastqc/. Accessed 10 May 2018

43. DeLuca DS, Levin JZ, Sivachenko A, Fennell T, Nazaire M-D, Williams C, et al. RNA-SeQC: RNA-seq metrics for quality control and process optimization. Bioinformatics. 2012;28:1530–2.

44. Zhao H. Alternative Splicing Analysis. protocols.io. 2018. dx.doi.org/10.17504/protocols.io.phndj5e

45. Bolger AM, Lohse M, Usadel B. Trimmomatic: a flexible trimmer for Illumina sequence data. Bioinformatics. 2014;30:2114–20.

46. Kim D, Langmead B, Salzberg SL. HISAT: a fast spliced aligner with low memory requirements. Nature Methods. 2015;12:357–60.

47. Trapnell C, Williams BA, Pertea G, Mortazavi A, Kwan G, van Baren MJ, et al. Transcript assembly and quantification by RNA-Seq reveals unannotated transcripts and isoform switching during cell differentiation. Nature Biotechnology. 2010;28:511–5.

48. Foissac S, Sammeth M. Analysis of alternative splicing events in custom gene datasets by AStalavista. Methods in Molecular Biology. 2015;1269:379–92.

49. Foissac S, Sammeth M. ASTALAVISTA: dynamic and flexible analysis of alternative splicing events in custom gene datasets. Nucleic Acids Research. 2007;35:W297–9.

50. Bauer S, Grossmann S, Vingron M, Robinson PN. Ontologizer 2.0--a multifunctional tool for GO term enrichment analysis and data exploration. Bioinformatics. 2008;24:1650–1.

51. The Gene Ontology Consortium. Expansion of the Gene Ontology knowledgebase and resources. Nucleic Acids Research. 2017;45:D331–8.

52. Marques AC, Tan J, Lee S, Kong L, Heger A, Ponting CP. Evidence for conserved post-transcriptional roles of unitary pseudogenes and for frequent bifunctionality of mRNAs. Genome Biology. 2012;13:R102.

53. Winter EE, Goodstadt L, Ponting CP. Elevated rates of protein secretion, evolution, and disease among tissue-specific genes. Genome research. 2004;14:54–61.

54. Wang B, Tseng E, Regulski M, Clark TA, Hon T, Jiao Y, et al. Unveiling the complexity of the maize transcriptome by single-molecule long-read sequencing. Nature Communications. 2016;7:11708.

55. PASA. http://pasapipeline.github.io/. Accessed 10 May 2018.

56. Zhao H. Orthologous Gene and Phylogenetic analysis. protocols.io. 2018. dx.doi.org/10.17504/protocols.io.phpdj5n.

57. Kersey PJ, Allen JE, Allot A, Barba M, Boddu S, Bolt BJ, et al. Ensembl Genomes 2018: an integrated omics infrastructure for non-vertebrate species. Nucleic Acids Research. 2018;46:D802–8.

58. Zhao H. Genome-wide identification of genes involved in the lignin biosynthetic pathway. protocols.io. 2018. dx.doi.org/10.17504/protocols.io.phjdj4n.

59. Roshan U. Multiple sequence alignment using Probcons and Probalign. Methods in Molecular Biology. 2014;1079:147–53.

60. Talavera G, Castresana J. Improvement of phylogenies after removing divergent and ambiguously aligned blocks from protein sequence alignments. Systematic Biology. 2007;56:564–77.

61. Darriba D, Taboada GL, Doallo R, Posada D. jModelTest 2: more models, new heuristics and parallel computing. Nature Methods. 2012;9:772–2.

62. Guindon S, Dufayard J-F, Lefort V, Anisimova M, Hordijk W, Gascuel O. New algorithms and methods to estimate maximum-likelihood phylogenies: assessing the performance of PhyML 3.0. Systematic Biology. 2010;59:307–21.

63. Yang Z. PAML 4: phylogenetic analysis by maximum likelihood. Molecular Biology and Evolution. 2007;24:1586–91.

64. Zhao H. Positive selection analysis. protocols.io. 2018. dx.doi.org/10.17504/protocols.io.phidj4e.

Figure legends:

Figure 1. Comparative results based on two versions of the moso bamboo genome.

(A) The distribution of contigs between two versions of the moso bamboo genome. Contig N50 and N90 are marked. (B) The distribution of scaffolds between two versions of the moso bamboo genome. Scaffold N50 and N90 are masked. (C) Box plots comparing the two versions of the moso bamboo genome, including gene length, intron length, CDS length, cDNA length, single exon length, and single intron length. (D) The BUSCO assessment result was provided, including the five assessment results (two genomes and three annotations). The two genomes contained the previous WGS version and the latest chromosome-level version. Annotation v1 was based on version 1 of the moso bamboo genome. Annotation v2.1 was based on version 2, and Annotation v2.2 was the manually verified version of Annotation v2.1.

Figure 2. The distribution of AS genes and events and their correlation

(A) The distribution of AS genes in bamboo, including the four main types and Iso-Seq results. (B) The distribution of AS events in bamboo, including the four main types and Iso-Seq results. (C) The correlation between AS genes and events. IR, A3SS, A5SS, and ES represent intron retention, alternative 3' splice site donor, alternative 5' splice site acceptor, and exon skipping, respectively.

Figure 3. The evolutionary analysis in plant species across bamboo

(A) The phylogenetic relationship of *Amborella trichopoda*, *Elaeis guineensis*, *Arabidopsis thaliana*, *Brachypodium distachyon*, *Oryza sativa*, *Spirodela polyrhiza*, *Sorghum bicolor* and *Ph. edulis*. Phylogenetic tree of the selected 8 plant species with branches leading to bamboo as red line. The notation indicates the unqiue eight orthologous gene datasets (D8-D1) identified in our study. (B) a Venn diagram of orthologous genes in related eight species was exhibited. (C) AS percentage and AS type were provided in two types of eight orthologous genes datasets. (D) increasing AS abundance and the decreasing tissues specificity were displayed in D8-D1.

Figure 4. The gene family expansion and AS abundance of bamboo in lignin biosynthetic pathway

A) A total of 13 families in lignin biosynthetic pathway were identified using six genomes, i.e., *A. thaliana*, *B. distachyon*, *O. sativa*, *Ph. edulis*, *P. trichocarpa*, and *S. bicolor*. Copy number and genes under

positive selection were added. B) The structure, distribution and types of AS and related gene expression level were exhibited in six gene families (4CL, C3H, CCR, HCT, LAC, and POD). The lignin biosynthetic enzymes are: PAL phenylalanine ammonia-lyase; TAL tyrosine ammonia-lyase; C4H cinnamate 4-hydroxylase; C3H 4-hydroxycinnamate 3-hydroxylase; COMT caffeic acid 3-O-methyltransferase; F5H ferulate 5-hydroxylase; 4CL 4-coumarate: CoA ligase; CCoA-3H coumaroyl-coenzyme A 3-hydroxylase; CCoA-OMT caffeoyl-coenzyme A O-methyltransferase; CCR cinnamoyl-CoA reductase; CAD cinnamyl alcohol, and HCT dehydrogenase hydroxycinnamoyl transferase.

Table 1. Statistics for the assembly of the moso bamboo genome using different sequence data

Statistics	WGS assembly		Hi-C assembly	
	Scaffold	Contig	Scaffold	Contig
Total number	19,285	76,900	19,684	84,758
Genome size (bp)	1,908,074,089	1,795,528,836	1,907,603,590	1,795,510,437
Gap number (bp)	112,545,253	0	112,093,153	0
Average length (bp)	98,940.84	23,348.88	96,911.38	21,183.96
N50 length (bp)	894,858	54,955	79,898,979	53,293
N90 length (bp)	115,487	11,757	44,603,463	10,445
Maximum length (bp)	5,406,526	738,589	137,299,170	738,589
Minimum length (bp)	926	157	318	1
GC content (%)	44.2	44.2	44.2	44.2

Figure 1

Click here to access/download;Figure;Figure1_ch_hic4.pdf

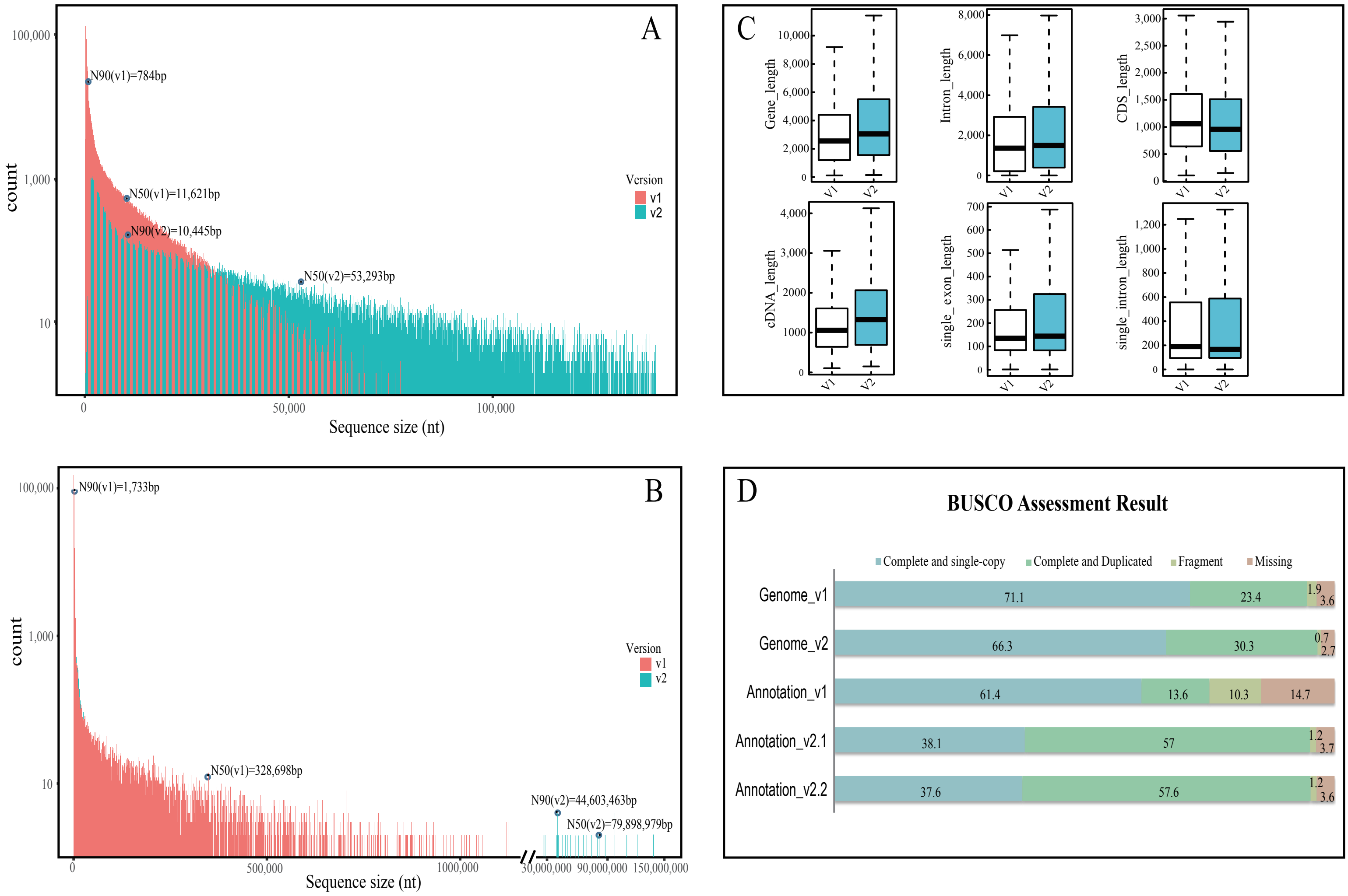

Figure 2

Click here to access/download;Figure;Figure2-revised2.pdf

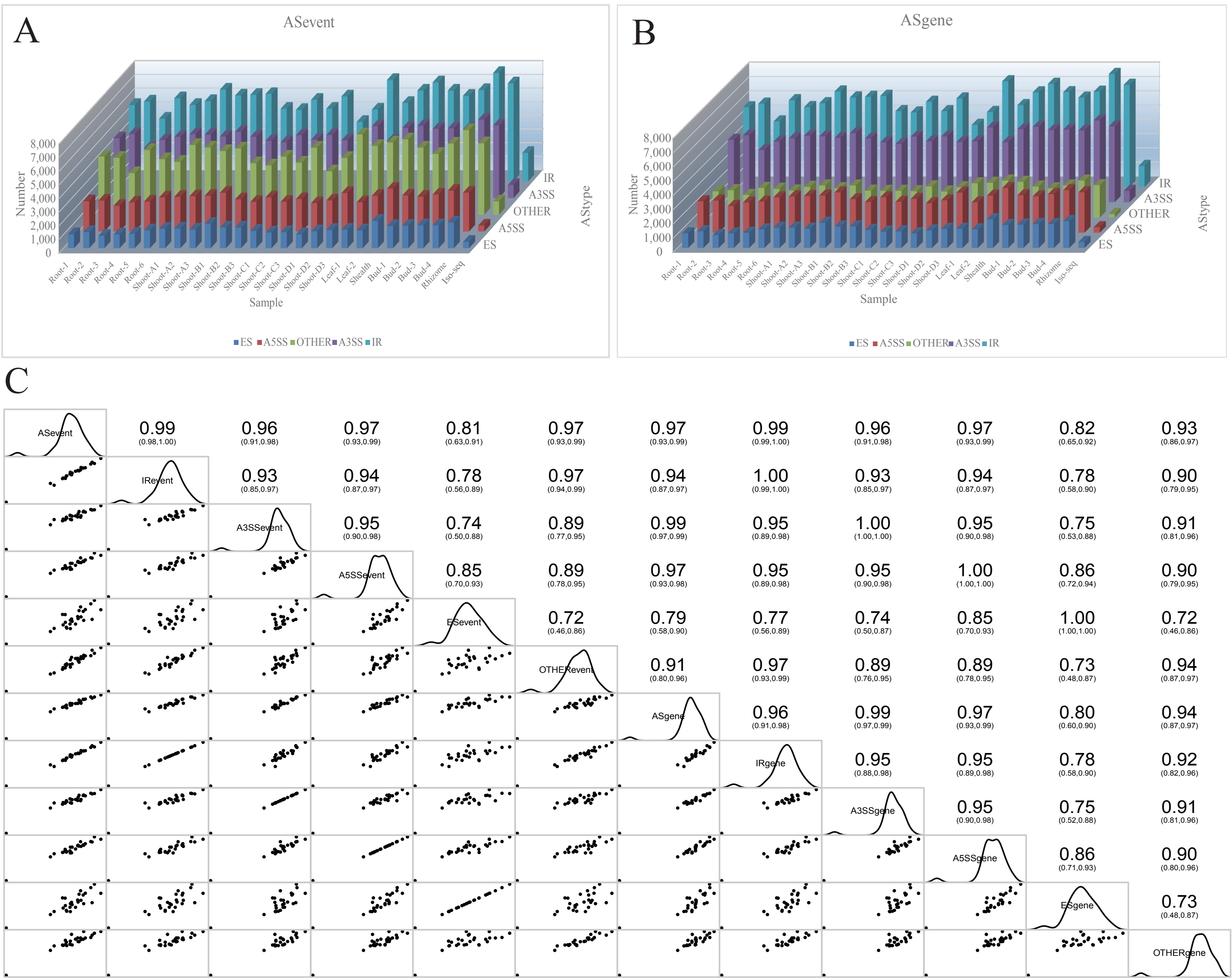

Figure 3 Click here to access/download;Figure;Figure3-revised2-n.pdf

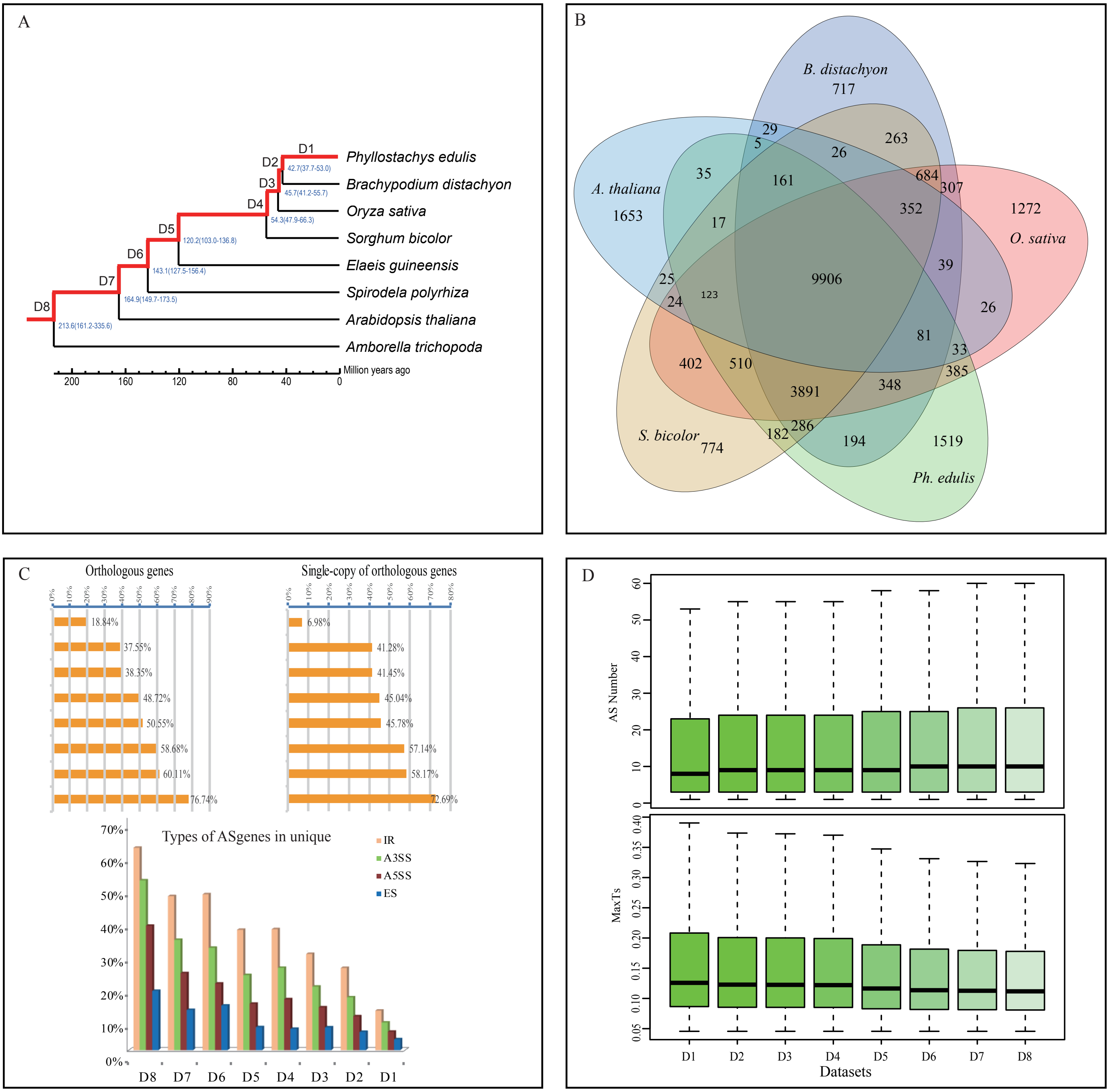

Click here to access/download;Figure;Figure4.pdf

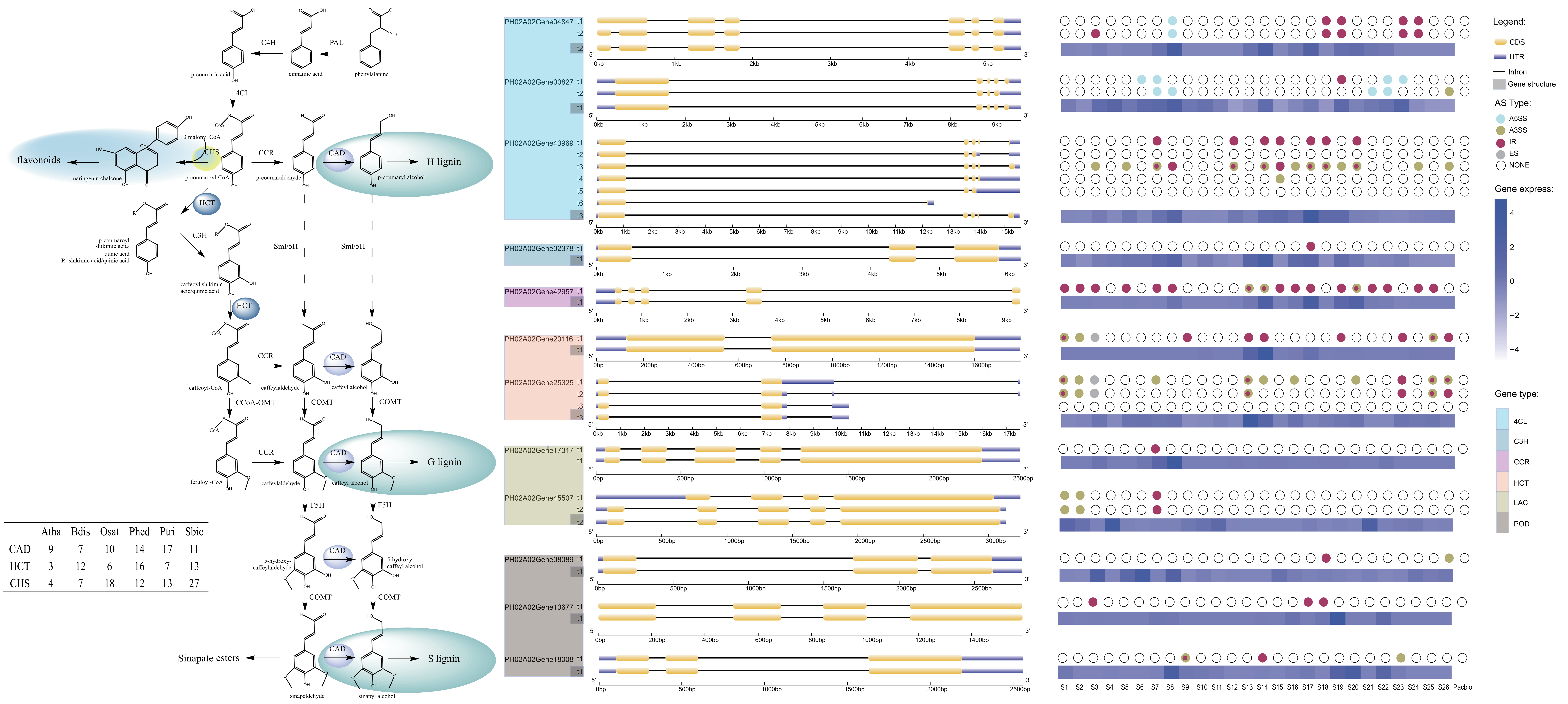

	Atha	Bdis	Osat	Phed	Ptri	Sbic
CAD	9	7	10	14	17	11
HCT	3	12	6	16	7	13
CHS	4	7	18	12	13	27

Supplemental file 1

Click here to access/download
Supplementary Material
Additional File-Revised2-30Aug-zhs.docx

Additional Table S25

Click here to access/download
Supplementary Material
Additional Table 25.xls

Additional Table S28

Click here to access/download
Supplementary Material
Additional Table 28.xls
