# Supplementary material for: Chromosome-level reference genome and alternative splicing atlas of moso bamboo (Phyllostachys edulis)
Source: Gigascience. 2018 Sep 8;7(10):giy115. doi: 10.1093/gigascience/giy115 (PMC6204424; doi:10.1093/gigascience/giy115)
Supplement: Reviewer_2_Revision_1_attachment.pdf [file giy115_reviewer_2_revision_1_attachment.pdf]

Chromosome-level reference genome and alternative splicing atlas of moso bamboo (*Phyllostachys edulis*)

Hansheng Zhao[1#], Chunhai Chen[2#], Benhua Fei[1#], Songbo Wang[2#], Chengcheng Shi[3], Jiongliang Wang[1], Xiaochuan Liu[3], Hailin Zhang[2], Yongfeng Lou[1], LianFu Chen[1], Huayu Sun[1], Xianqiang Zhou[2], Sining Wang[1], Chi Zhang[2], Hao Xu[1], Lichao Li[1], Yihong Yang[1], Yanli Wei[2], Wei Yang[2], Qiang Gao[2], Huanming Yang[2], Zhimin Gao[1+], Shancen Zhao[2+] and Zehui Jiang[1+]

[1] State Forestry Administration Key Open Laboratory on the Science and Technology of Bamboo and Rattan, Institute of Gene Science for Bamboo and Rattan Resources, International Center for Bamboo and Rattan, Futongdong Rd, WangJing, Chaoyang District Beijing 100102, China;

[2] BGI Genomics, BGI-Shenzhen, Building NO.7, BGI Park, No. 21 Hongan 3rd Street, Yantian District, Shenzhen 518083, China;

[3] BGI-Qingdao, No. 2877, Tuanjie Road, Sino-German Ecopark, Qingdao, Shandong Province, 266555, China;

[+] Co-corresponding author: gaozhimin@icbr.ac.cn, zhaoshancen@genomics.cn, and jiangzehui@icbr.ac.cn

[#] These authors contributed equally to this work.

Abstract

Background

Bamboo is one of the most important non-timber forest products worldwide. However, a chromosome-level reference genome is lacking, and the evolutionary landscape of alternative splicing (AS) in bamboo remains unclear.

Results

Here, we provide a chromosome-level *de novo* genome assembly of the moso bamboo (*Phyllostachys edulis*) using additional sequencing data and a HiC scaffolding strategy. The significantly improved genome is a scaffold N50 of 79.90 Mb, approximately 243 times longer than the previous version. A total of 51,074 high-quality protein-coding loci with intact structures were identified using single-molecule real-time sequencing and manual verification. Moreover, we provide a comprehensive AS profile based on the identification of 266,711 uniform AS events in 25,225 AS genes by large-scale transcriptomic sequencing of 26 representative bamboo tissues using both the Illumina and PacBio sequencing platforms. Via comparison with orthologous genes in related plant species, we observed that the AS genes are concentrated in more conserved genes that tend to accumulate higher expressed transcripts and share less specificity. Furthermore, gene family expansion, abundant AS and positive selection were identified in crucial genes involved in lignin biosynthesis, indicating that moso bamboo is a woody plant in the grass family.

Conclusions

These fundamental studies provide useful information for future studies performing in-depth analyses of comparative genome and AS features. Additionally, our results highlight a global perspective of AS during evolution and diversification in bamboo.

Keywords: Moso Bamboo, Genome, Annotation, Alternative Splicing, Transcriptome, Evolution

Background

Bamboo (Bambusoideae) is a fast-growing plant with substantial potential for generating income, restoring degraded landscapes and combating climate change in numerous Asian and African countries. Approximately 2.5 billion people economically depend on bamboo, reaching an annual international trade of over 2.5 billion US dollars [1]. Bamboo is a perennial grass in temperate and tropical forests worldwide. Its cellulose and hemicelluloses content is comparable to that of woody trees [2]. Moso bamboo (*Phyllostachys edulis*) accounts for ~73.76% of the bamboo growing region in China (4.43 million ha), constitutes the most abundant natural resource of non-wood products and plays significant roles in economics, ecology, culture, aesthetics and technology [3].

Only a limited number of genome-wide studies have been investigated in bamboo. We first reported a draft genome of moso bamboo in 2013 and released 2.05 Gb of the draft genome with 328 Kb of Scaffold N50 and 31,987 predicted genes [4]. Due to the development of sequencing technology and analytical methods, a chromosome-level reference genome with improved precision and contiguity could facilitate functional and evolutionary analyses of bamboo.

Alternative splicing (AS) is a major mechanism underlying the increased complexity and diversity of proteins made from a limited number of genes in eukaryotes [5]. More than 95% of human multi-exon genes have been predicted to express multiple splice isoforms [6,7], and the occurrence of AS events in plants is reported to be ~61%, ~52%, ~42%, ~40%, ~40% and 33% in *Arabidopsis thaliana* [8,9], *Glycine max* [10], *Brachypodium distachyon* [11], *Gossypium raiimondi* [12], *Zea mays* [13] and *Oryza sativa* [14], respectively. The different splicing products of a single gene represent major sources of functional plasticity and supposedly play important roles in plant growth, development, defense responses, signal transduction and flowering time [15-19]. Species-specific AS is partly responsible for generating a wide variety of functional diversity with limited repertoires of protein coding genes [20-22]. However, the mechanism by which AS modulates the evolutionary process in plants is unclear. Moreover, the AS characteristics of genes with a diverse conservative status remains elusive.

In this study, we substantially improved the moso bamboo genome assembly and gene annotation. Based on the improved genome reference, we performed a comprehensive genome-wide analysis to uncover the AS landscapes in bamboo using transcriptome data from 26 mixed samples collected from six main

bamboo producing areas in China. These transcriptome data were generated using the Illumina and PacBio platforms. Numerous AS genes and events were detected, and various types of AS events were identified. We performed a genome-wide investigation to determine the relationship between amino acid conservation and AS and examine the evolution of AS status of genes that are involved in the lignin biosynthesis. In conclusion, our analysis not only provides a global profile of AS in bamboo for further experimental studies investigating the functions of genes and regulatory networks, but also reveals the roles of AS in the evolutionary landscape.

Data description

For the assembly of the moso bamboo genome, approximately 603.3 Gb genome data with different sequencing strategies were generated. The WGS assembly was performed using ~154 Gb of newly acquired and ~220 Gb of previously acquired clean data [4]. The Hi-C assembly was using ~157 Gb raw data from Hi-C library and 17.58 Gb valid reads were obtained after quality control (Additional Table S1). Additionally, for the transcriptomic analysis, approximately 379 Gb and 5 Gb of raw data were produced from the Illumina and PacBio platforms, respectively (Additional Tables S2-7). Thus, we identified 266,711 unique AS events in 25,225 AS genes in moso bamboo according to the chromosome-level genome reference and the high-throughput transcriptome data.

Analyses

Chromosome-level genome assembly and gene annotation in moso bamboo

In order to enhance the quality of the moso bamboo genome, a total of 61 libraries were used and subjected to sequencing according to the instructions of the sequencer manufacturer (Additional Table S1). In total, we obtained ~ 603.3 Gb genome data with read length ranging from 76 bp to 250 bp. Subsequently, we performed different assembly strategies to obtain a better genome assembly (see the Additional File for details). First, the WGS assembly reached 1.91 Gb with a contig and scaffold N50 length of 55 Kb and 894 Kb, respectively (Additional Table S8). Compared with those of our previous version [4], the assembly statistics and quality of the new WGS assembly were obviously improved (Additional Tables S9-10). For example, the length of scaffold N50 and contig N50 were increased by 172% and 358%, respectively, and the ‘N’ base rate was decreased by 43%. Then, the Hi-C assembly was generated with total length reached

1.91 Gb as well as contig and scaffold N50 length with 53.29 Kb and 79.90 Mb (Fig. 1a and 1b). About 93.17% scaffolds from the WGS assembly were anchored onto 24 chromosomes (Additional Table S10) [23] and the scaffold N50 was increased by ~89-folds (Table 1). According to the contact map (Additional Fig. S1) and the assembly results, the boundaries between 24 chromosomes were observed clearly. Then we aligned the moso chromosomes to the rice genome and we found a mean coverage of ~59.77% (Additional Fig. S2 and Additional Table S11). Additionally, we evaluated the chromosome-level assembly using bamboo-derived BAC sequences, full-length cDNAs [24] and some known genes (Additional Fig. S3 and Additional Tables S12-14). The chromosome-level assembly had a more extensive genome coverage, and the accuracy was higher than that of the first assembly.

The chromosome-level assembly generated could facilitate gene prediction in subsequent analyses after annotating repetitive sequences (Additional Table S15). Based on numerous transcriptomic data (Additional Table S16), full-length cDNAs [24], and homologous proteins, we predicted 51,074 high-quality protein-coding loci with intact structures in moso bamboo (Additional Table S17). The average introns and exons were 668 bp and 284 bp in length, respectively (Fig. 1c and Additional Table S18). A combination of single-molecule real-time sequencing and manual verifications was carried out to confirm or correct certain irregular predictions. About 17% of the gene models were improved by the UTR addition and internal structural adjustment (Additional Table S19). According to the completeness assessment of the annotation using BUSCO [25], moso bamboo (95.2%) was more complete than *Z. mays* (92.2%) but close to *O. sativa* (95.6%) (Fig. 1d and Additional Table S20). Compared with the previous annotation, 97.23% of the gene models in our analysis were identified in public databases, which facilitated the accurate detection of alternative splicing events (Additional Table S21). Detailed information regarding the gene model prediction and genome evolution are presented in Additional Tables S22-24 and Figs. S3-9. Additionally, the latest genome assembly and gene annotation were released at the *Giga*DB [26]. The entire dataset comprises genome assemblies, gene sets, a list of repeat elements, tRNAs, miRNAs, gene clusters, and the newly released bamboo genome, providing a reliable resource for many analyses, including genomic, genetic, and molecular biology experiments

Vast transcriptomic data generated using the Illumina and PacBio platforms

To facilitate the genome-wide investigation of the AS landscape in moso bamboo and comprehensively

identify the factors that influence AS at the post-translational level, we performed high-throughput RNA sequencing (RNA-Seq) using the Illumina HiSeq-4000 platform. In total, 26 individual representative RNA samples were sequenced (150 bp paired ends; Additional Table S2 and Figs. S10-11). After preprocessing, we obtained an average of 90 million high-quality reads (~13.6 Gb) per sample, accounting for 92.78% of the raw reads. Approximately 80.57% of the high-quality reads were mapped to the reference genome at a unique position and designated unique reads (Additional Tables S3-4). According to the alignment distribution, most sequences were mapped in exonic regions. The exon-mapping rate was on average 81.94%. The remaining reads were mapped in intronic regions (8.46%) and intergenic regions (9.6%) (Additional Table S5 and Figs. S12-13). The exonic coverage was found to be ~2,521× per sample (Additional Fig. S14). Therefore, this large-scale, in-depth high-quality transcriptomic data, together with a high-quality reference genome, contributed to an accurate AS identification in moso bamboo.

To accurately identify the full-length splice isoforms, we sequenced the bamboo transcriptome using the PacBio platform. The full-length cDNA sequencing of alternatively spliced isoforms (Iso-Seq) used RNA from a mixture of 26 samples. According to the length distribution of the transcripts in all samples (Additional Table S6), we constructed 3 SMRTbell libraries (1-2 kb, 2-3 kb, and >3 kb) for the mixed sample and sequenced 9 cells, generating ~562 Mb of raw data and 214,372 reads-of-insert (ROIs), including 133,599 full-length ROIs (containing a 5' primer, 3' primer and a poly(A) tail); the remaining ROIs were non-full-length ROIs (Additional Table S7 and Fig. S15). The accuracy evaluation based on aligning the ROIs against the new genome showed that the per-nucleotide error was approximately 2.05% and consisted of mismatches (0.32%), insertion (0.98%) and deletions (0.75%).

Numerous genes underwent AS in moso bamboo

Based on the improved reference genome and large-scale transcriptome data, we performed a genome-wide analysis to identify AS in moso bamboo using the previous pipeline [10]. In total, 266,711 unique AS events were identified in 25,225 AS genes, accounting for *ca.* 49.39% of all annotated genes. Except for the 12,653 AS genes identified in the gene annotation, the remaining (12,572) genes were considered novel AS genes (Additional Fig. S16).

The Iso-Seq data were also utilized to detect AS in an analysis parallel to the Illumina RNA-Seq analysis. In total, 4,246 AS events and 2,218 AS genes were identified (Fig. 2a, b). According to the

PacBio-Illumina overlapping analysis, which was performed to assess the validity of the AS prediction, 81.21% of the AS events and 97.34% of the AS genes identified in the Iso-Seq analysis completely overlapped with those in the RNA-Seq analysis. Among the four main AS types, on average, 80.37% of the AS events and 95.59% of the AS genes also overlapped (Additional Fig. S17). This high proportion of the PacBio-Illumina overlapping AS genes is a strong indicator of the validity of the computationally predicted AS.

The AS gene number was strongly and positively correlated with the AS event number (correlation coefficient = 0.97, Mann-Whitney U test with p-value <0.05) (Fig. 2c). In subsequent analyses, we defined the four main AS types as: intron retention (IR), alternative 3' splice site donor (A3SS), alternative 5' splice site acceptor (A5SS), and exon skipping (ES), and we also defined the other AS types represented some AS types except the above four main AS types. The four main AS types were detected in the AS events in moso bamboo according to the canonical splicing patterns (GT-AG, GC-AG, and AT-AC splice sites). As shown in Fig. 2b, intron retention (IR, 38.22%) represented the most abundant type of AS event, followed by alternative 3′ splice site acceptor (A3SS, 20.20%) and alternative 5′ splice site acceptor (A5SS, 10.48%). Exon skipping (ES, 2.92%) was the least prevalent type among the four main AS types.

As the functional implication of AS genes, the enrichment analysis result showed 885 genes, which alternatively spliced in all samples, significantly enriched in RNA metabolic processing, mRNA processing, RNA processing and RNA splicing (Additional Table S25). Since AS possess strong specificity to different tissues or developmental stages, we identified 181,105 tissue-specific AS events (67.57%), which account for two-thirds of the AS events (termed as among-tissue). Then, the remaining two-third of the AS events were detected based on comparisons of the transcript isoforms within individual tissues (termed as within-tissue) (Additional Fig. S18).

The transposable element (TE) analysis showed 26,366 genes have TE insertion, accounted for 51.62% of all genes, and the total length of TE-insertion in genes was ~46 Mb. According to the different position of the TE-inserted intron, TE-introns mainly concentrated in the front and rear of a gene (Additional Fig. S19). Additionally, the usage and distribution of splice sites demonstrated GT-AG splice sites were the most abundant, corresponding to 97.31% of entire AS events, followed by GC-AG (2.33%) and GT-AT (0.32%) splice sites (Additional Fig. S20). Except for the canonical splice site (GT-AG, GC-AG, and AT-AC), the remaining 2,406 splice sites were identified as non-canonical splice sites, contained 2,373 GT-AT

splice sites and 33 splice sites of other types.

Evolutionary analysis of AS in moso bamboo

Based on the genome-wide identification of orthologous genes in the selected 8 plant species (*Amborella trichopoda*, *A. thaliana*, *Elaeis guineensis*, *B. distachyon*, *O. sativa*, *Spirodela polyrhiza*, *S. bicolor* and *Ph. edulis*) and the species divergence time in a phylogeny tree (Fig. 3a), we identified eight orthologous gene datasets. For instance, dataset8 (D8) represented orthologous genes that are present in all the eight plant species. D7 represented the common orthologous genes in all but *A. trichopoda* (i.e. the earliest-diverging species) and D7 does not contain orthologues genes in D8. D1, on the other hand, represented the bamboo-specific genes. We considered the bamboo-specific genes (i.e. D1) are poorly conserved, whereas the genes present in all the selected plant species (D8) are highly conserved. The degree of conservation decreased monotonically from D8 to D1. AS was detected in all the datasets, but the proportion of AS genes in each dataset gradually decreased from D8 to D1 (Mann-Whitney U test with $p<0.05$). This trend was also observed in the two other datasets, i.e., removing common genes in more than two gene datasets in eight original datasets and using single-copy genes in eight original datasets. The eight original datasets were derived from the genome-wide identification of orthologous genes in the selected 8 plants. Therefore, the robust pattern, i.e., more conserved genes having more AS genes, should also exist in bamboo.

We investigated the distribution pattern of the four focal AS types in each dataset and found the identical trend (Fig. 3b), but the proportion of the AS types significantly differed (IR>A3SS>A5SS>ES). The proportion of IR in D8 was 60.80%, which was ~6-folds of that in D1 (11.88%). The ratio of the other AS types increased as the level of conservation decreased. In all datasets, the number of AS events gradually decreased from D8 to D1 (Fig. 3c). The most abundant AS events appeared in D8, and the least abundant AS events were detected in D1. Additionally, compared with the AS events among the genes expressed in samples with different specificities (maxTs) (for details, see Methods), the maxTs obviously increased from D8 to D1, representing an enhancement in the sample specificity from a highly conserved gene dataset to a poorly conserved dataset (Fig. 3d). Altogether, the conserved genes tended to have more AS genes, more AS events and less tissue? specificity.

We also examined the correlations among the gene length, CDS length, intron length, exon number, exon cassette length, and intron cassette length in all datasets (Additional Fig. S21). All genes in the

different datasets (D1-D8?) were positively correlated with the gene length, CDS size, intron size, and exon number and negatively correlated with the exon cassette length and intron cassette length. Moreover, the distribution of the TE genes in the eight datasets was examined and a substantially negative correlation was observed, indicating that the more conserved genes had more TE insertions.

Expansion of the gene family involved in the lignin biosynthesis pathway and implications for gene functional diversity

We systematically identified 13 gene families involved in the lignin biosynthesis pathway using the six genome sequences of *A. thaliana*, *B. distachyon*, *O. sativa*, *Ph. edulis*, *P. trichocarpa*, and *S. bicolor*. The expansion of most families was detected in bamboo (Additional Table S26). Each gene had multiple copies in the bamboo genome, and the total size of the gene families in the lignin biosynthesis pathway was the largest in bamboo, with an average of ~19 copies per family. The highest and lowest copy numbers were detected in the peroxidase gene family (*POD*, 77 genes) and *p*-coumarate 3-hydroxylase gene family (*C3H*, 3 genes), respectively. Additionally, we calculated the synonymous substitution rate analysis for 13 gene families evolved in the lignin biosynthesis using the yn00, which was a package in PAML to estimate synonymous and nonsynonymous substitution rates. Then, the Ks rate was translated to the divergence time by the formula $T=Ks/2r$ ($r=6.5\times10^{-9}$). As shown in Additional Fig. S22, the result indicated that the divergence time of the lignin biosynthesis genes occurred at the 5~16 million year ago (Mya), which correspond to the whole genome duplication (WGD) time 7~12 Mya in the moso bamboo genome [4].

Moreover, we performed an AS analysis of the genes in the lignin biosynthetic pathway. In total, 10 of the 13 families had AS genes accounting for more than half of the total, except for the ferulate 5-hydroxylase (F5H) gene family, which had a low proportion, and the CHS and caffeic acid *o*-methyltransferase (COMT) gene families, in which AS genes were not detected. A high percentage (>75%) of AS events was observed in the 4-coumarate: CoA ligase (4CL), hydroxycinnamoyl transferase (HCT) and cinnamyl alcohol dehydrogenase (CAD) gene families. In addition, we tested for positive selection in the gene families involved in the lignin biosynthetic pathway using a branch-site model. Several genes in two gene families, i.e., *HCT* and *CAD*, exhibited positive selection. The information provided by the phylogenetic relationship using the best model and log likelihood ratio (lnL) was provided in Additional Table S27.

Discussion

High-throughput genome sequencing and assembly strategy were broadly applied in current plant genomic studies with the development of new technologies and more useful data. In 2013, our initial analysis of the *Ph. edulis* genome provided a genome-wide perspective of the structures of the genome and genes, the history of the whole-genome duplication events, and the functional genes in critical functional categories[4]. In the present study, we enhanced both the precision and contiguity of the *Ph. edulis* genome and updated its annotation, accurately positioning the bamboo genome in an evolutionary landscape by performing comparative studies involving different species. Additionally, various biological characteristics of bamboo were studied in great detail using knowledge obtained from the latest version. Therefore, the chromosome-level reference genome and refined annotation paved the way for future genomic studies of bamboo and other related plants.

We provided the global AS landscape in bamboo based on a large amount of high-throughput data from RNA-Seq and Iso-Seq. These data enabled the accurate detection of transcripts with a low expression level and the acquisition of the complete gene structure, particularly in the AS analysis. A series of AS analyses improved our understanding of AS in bamboo during post-transcriptional regulation, including the identification of AS genes and AS events, the distribution of the AS types, the use of a splice site, the length distribution of an alternative exon, etc. AS is considered a major mechanism responsible for creating the multicellular diversity from a limited repertoire of genes. For example, by combining one exon of four alternatively spliced regions that contain 12, 48, 33, and 2 alternative exons each, it is possible to generate, at most, 38,016 protein isoforms (12×48×33×2) from the *Dscam* gene in *Drosophila* [28]. In bamboo, we identified 266,711 AS events and 25,225 genes in all samples, and on average, 15,971 AS events and 9,080 AS genes were detected in each sample. Thus, AS might be tissue specific, and the actual AS percentages in bamboo might be underestimated. More AS events, supported by transcripts with a low expression level, can be detected as the sequencing depth increases [29]. Additionally, the distribution of the AS type is consistent with that in *Arabidopsis* [5,9,29], soybean [10], and maize [13]. Nevertheless, a higher percentage of IR (38.22%) and other AS types (total 28.18%) were observed in bamboo. This higher percentage may be due to the unique features of bamboo and/or the depth of the

sequencing, which can be tested in future comparative analyses. In addition, TEs constitute crucial gene regulatory elements and influence gene transcription and gene expression [30]. We did not detect a noticeable relationship between the TE genes and AS genes. Although previous reports have indicated that TE inserted within an intron interferes with the normal splicing pattern of pre-mRNA, resulting various forms of alternative splicing [31], our result implied that TE might be a driving force during the formation process of AS in bamboo. Furthermore, the identification of splice sites in an individual gene may provide an essential resource for fully understanding alternative splicing and isoform construction [32,33]. With respect to their distribution, the main AS types (i.e., GT-AG, GC-AG, and AT-AC) were consistent with those previously observed in animals and other plants [19].

More AS events were identified in the sample with vigorous growth, which is consistent with the previous studies [29,34]. However, according to our observations, the rhizome tissue had more AS events than the root tissue in moso bamboo, which may be because the two tissues play different roles during bamboo development. Photoassimilates were unavailable during the rapid growth of the moso bamboo shoots since no leaves were growing [35], and thus, large amount of nutrients and energy in the shoot would have to come from the attached matured bamboos through underground rhizomes. Therefore, as a rhizomatous plant, the rhizome in moso bamboo plays a critical role in the transportation of nutrients and energy, which might explain the higher number of AS events detected in the rhizome. Moreover, to investigate the relationship between the incredible growth speed and AS in the shoots of moso bamboo, we selected shoots with 4 different heights and sampled 3 internodes (i.e., top, middle, and base) from each shoot according to the classification of shoot development. Obvious differences were observed in the AS event numbers in the final three shoot developmental stages, likely contributing to the fast growth during shoot development.

We performed an evolutionary analysis to examine the relationship between AS and evolution using a comparative genome analysis. To date, the relationship between gene conservation and AS remains unknown. To explore this issue, we performed a genome-wide analysis to examine AS in eight gene datasets with different degrees of conservation. The AS genes were more likely to be enriched in the highly conserved gene datasets, and these AS genes had more AS events. This finding was robust because we analyzed using the orthologous genes only in one dataset and using single-copy genes in selected species, respectively. Previous reports have demonstrated that duplication is a major source of functional diversity

and the generation of new genes in plants [36], and new genes have generally low expression and suffer certain restrictions [37]. Altogether, we proposed that the relationship between conservation and AS may be associated with gene evolution and the generation of new genes. New genes might first generate a single-functional gene without an AS event and then gradually form multifunctional and conserved genes with many AS events [22]. Conserved genes tend to be the hubs in gene-gene interaction networks, indicating their functional diversity, and during the gradual evolutionary process, newly generated genes are gradually added to this network and acquire pleiotropic roles [38]. Additionally, the four main AS types were abundant in the highly conserved gene datasets, and many other AS types appeared in the poorly conserved datasets. Thus, the four main AS types were conserved, and other types might represent an intermediate stage. The distribution of the AS types depicted that IR occupied the dominant position, indicating that the importance of IR could be inferred from inspecting its prevalence throughout evolution in plants. The allocation in animals and yeast differs from that in plants. The most abundant AS event is ES, followed by AA and AD, while IR is the least common [39]. The discrepancies in the occurrence of the AS models between plants and animals suggest that differences exist between plants and animals in the genomic structure and mechanism of splice site recognition [40].

According to our results, the highly conserved gene datasets had more AS genes and events, which either produce functional alternative protein-coding transcripts with distinct functions in biological processes or modulate the functional spliced transcript level by producing certain non-coding transcripts [22]. We hypothesize that the highly conserved genes with more AS events might be critical for evolution and function in generating gene functional diversity and the generation process of the highly conserved genes might undergo rigorous regulation during long-term evolution since the poorly conserved genes had less AS events than the highly conserved genes. Additionally, compared with the poorly conserved gene datasets, the highly conserved AS gene datasets had a low tissue-specific expression profile, indicating these genes might be core genes in fundamental functions, such as serving as hubs in gene-gene networks. Therefore, we proposed that functionally important genes are generated by more frequent AS events. As an essential biological process, AS plays a crucial role in acquiring more functions, which might explain why the highly conserved AS possesses more AS events. We hypothesize that this phenomenon likely applies not only to bamboo but also to other plants or even animals.

Furthermore, we observed the relationship between the AS genes in different conserved datasets and

gene structure features. The AS genes in the highly conserved gene datasets possessed a longer gene length and more CDS, introns and exons and a shorter exon and intron cassette length. The architecture of the longer introns and shorter exons detected in the highly conserved gene dataset might be helpful in matching the constraints imposed by splicing recognition in the evolutionary process [21]. The exon-intron architecture in the different conserved genes might indicate that the splice-site choice and transcription by RNA polymerase II is changed during evolution [41,42]. During the evolutionary process, a new gene might be generated by duplication, which then forms less AS under strict constraints. Subsequently, functional AS is gradually generated and then evolves more functions largely through inducing changes in the gene structure, such as increasing the gene lengths, shorting the lengths of the exon cassette, inducing site mutations, etc.

Lignin represents a class of complex aromatic heteropolymers of monolignols that encrusts and interacts with the cellulose/hemicellulose matrix of the secondary cell wall [43]. Lignin accounts for up to ~25% of the total dry weight in bamboo [2]. We performed a thorough examination by combining AS and evolution analyses of the lignin biosynthesis pathway. The expansion of the gene families in the lignin biosynthesis pathway was detected in bamboo. Combined with the results of the divergence time of the lignin biosynthesis genes and our previous study [4], we estimated the occurrence of a putative WGD event at 7~12 Mya in the moso bamboo genome, suggesting that there might have been a tetraploidization event during bamboo history [4]. Then, the ancient tetraploid evolved into a current diploid moso bamboo. Additionally, WGD could provide more gene copies, which facilitated evolving the genes with new functions [44]. Therefore, the expansion of the lignin biosynthesis genes in moso bamboo could be due to the occurrence of WGD event. Additionally, the two gene families (i.e., *HCT* and *CAD*) underwent more AS events and positive selection. HCT generates lignin by catalyzing *p*-coumaroyl CoA [45]. Then, *p*-coumaroyl CoA is also catalyzed by CHS to generate flavonoids. HCT and CHS compete with each other to bind *p*-coumaroyl CoA. In bamboo, the *HCT* family has more members and AS events than the *CHS* family, which indicate that the *HCT* family might be in a dominant position in the competition to bind *p*-coumaroyl CoA. CAD catalyzes many different substrates to generate different types of lignin. The aromatic lignin polymers commonly found in bamboo are composed of three monolignols, namely, *p*-hydroxyphenyl (H), vanillin (G), and syringaldehyde (S). Previous studies have shown the abundance of G and S lignin and a small amount of H lignin in bamboo [2]. The *CAD* family expansion in bamboo and

positive selection may explain the different preferences of substrates to generate different proportions of monolignols in bamboo. The abundance of AS events, gene expansion, and positive selection were all consistent with the phenomenon that bamboo is remarkably adaptive to produce lignin.

Conclusions

To deeply explore the AS profile in the evolutionary landscape in bamboo, we improved reference genome and refined the annotation of moso bamboo. Based on the chromosome-level genome sequence and the abundant transcriptomic data from multiple tissues from six main bamboo producing areas in China, we provide a comprehensive AS perspective of moso bamboo by identifying 266,711 unique AS events in 25,225 AS genes using both the Illumina and PacBio sequencing technology platforms. Moreover, the integrated analysis of the AS results in bamboo and comparative analysis among eight representative plant species exhibited that the more conserved genes tended to accumulate higher transcript levels and exhibit less specificity. Finally, by studying the lignin biosynthesis based on AS and evolution, we observed several characteristics of crucial genes related to lignin biosynthesis in bamboo, including gene family expansion, abundant AS and positive selection. In summary, these results will likely provide important resources for studies investigating bamboo's unique woodiness in the grass family and exploring AS in the bamboo evolutionary landscape.

Method

Plant material collection

To obtain a comprehensive AS profile, the moso bamboo (*Phyllostachys edulis*) samples used in these experiments were collected from six main bamboo producing areas in China during the Spring of 2105, including (1) YiXing, JiangSu Province (N:31°15′08.41″, E:119°43′42.55″, 212 M), (2) TianMu Mountain, ZheJiang Province (N:30°19′13.42″, E:119°26′55.21″, 480 M), (3) XianNing, HuBei Province (N:29°81′10.02″, E:114°31′21.12″ 150 M), (4) TaoJiang, HuHan Province (N:28°28′39.74″, E:112°11′18.62″, 320 M), (5) GuiLin, GuangXi Province (N:28°28′39.74″, E:112°11′18.62″, 216 M) and (6) ChiShui, GuiZhou Province (N:28°28′15.27″, E:105°59′41.43″, 120 M). Twenty-six tissues were collected, including the rhizome, root, shoot, leaf, sheath, and bud, during different developmental stages.

Each mixed sample was collected from the above six areas. Detailed information regarding the biological samples is provided in Additional Table S19.

Genome sequencing, assembly and annotation

We assembled the moso bamboo genome using WGS and Hi-C strategies and annotated the new genome sequence as described in a previous study [46] and Additional Files. The detailed descriptions of this section were provided in Protocol.io [47].

Hi-C library preparation, sequencing and assembling

The construction of Hi-C library was prepared as previously described [46] and the detailed descriptions were presented in Additional Files.

RNA isolation and Illumina RNA-Seq library construction

We used standard methods of RNA isolation, purity, concentration, reverse transcription, and cDNA library construction, as described in the previous study [48]. All cDNA libraries were constructed and normalized as described in the Additional File.

RNA-Seq using the Illumina platform

After passing quality control, the pooled libraries were optically examined using an Illumina Cluster Station and were then sequenced on the Illumina HiSeq-4000 platform (150bp paired-end) according to the manufacturer's protocols. Finally, the quality of the reads was evaluated, and the low-quality reads were filtered using FastQC (version 0.11.3, http://www.bioinformatics.babraham.ac.uk/projects/fastqc/) with the default parameters. The statistics of the key metrics applied to the RNA-Seq data were calculated using RNA-SeQC (version1.1.8) [49] with the default parameter.

RNA-Seq data analysis

The detailed description of this section was provided in Protocol.io. Briefly, the adaptor sequences and low-quality sequences were trimmed using Trimmomatic (version 0.33)[50] during the preprocessing of the RNA-Seq data. Then, the cleaned data were mapped to the improved genome using HISAT2 (version 2.0.2) [51] with the following modifications from the default parameters: maximum intron length (4,000); specify strand-specific information (RF); and minimum score (L, -0.1, -0.1); report alignments tailored to

transcript assemblers were allowed. The empirical transcripts in each sample were obtained using Cufflinks (version 2.2.1) [52] after the reads were aligned. The default parameters were used, except for the following parameters: the minimum isoform fraction (0.05); the small anchor fraction of the spliced reads (0.05); the minimum intron length (20); the maximum intron length (4,000); the library type (fr-firststrand); the corrected frag bias; and the corrected multi-read. ASTALAVISTA (version 4.0) [53,54] was used with the default parameters to identify the AS genes and events after the different assembled transcript isoforms were mapped to the corresponding gene model using Cuffcompare, which is a component of the Cufflink program. The main four types, *i.e.,* IR, A3SS, A5SS, and ES, were analyzed and compared?. In addition, an enrichment analysis of the different genes was conducted using Ontologizer (version 2.0) [55] with the annotations from the Gene Ontology (GO) database (www.geneontology.org). We also calculated the sample specificity (Ts) values in each sample and each gene based on the expression level (FPKM values, the total number of fragments per kilobase of sequence per million reads mapped). A detailed description is provided in the previous report [56]. Briefly, Ts is defined as the fractional expression of a gene in one sample tissue relative to the sum of its expression in all samples. Thus, the maximum Ts value (maxTs) of a gene serves as an indicator of the sample specificity. Higher specificity values represent more tissue-specific expression [57].

Construction and sequencing of the Iso-Seq library

The construction of Iso-Seq library and sequencing were performed based on the PacBio manufacturer's protocol as previously described [58]. According to the length distribution of the transcripts predicted by bioinformatics (Additional Table S22), three SMRTBell libraries (1-2 kb of 3 cells, 2-3 kb of 2 cells, and >3 kb of 4 cells) were size-selected and a total of 9 SMRT cells were sequenced on the PacBio platform.

Iso-Seq data analysis

The sequencing data produced using PacBio RS II were processed to obtain consensus full-length isoforms. The isoforms from the multiple libraries were merged, and redundancy was removed to obtain the final consensus isoforms after processing the reads of the insert, classifying, and clustering. The assembled transcripts were mapped to the reference genome using PASA (version 2.0.2, http://pasapipeline.github.io/) with the default parameters. Then, similar to the short-read data, the output

file of the gtf was analyzed using ASTALAVISTA with the default parameters to identify the AS.

Evolutionary analysis

We identified gene families, constructed a phylogenetic tree, predicted divergence times as a previously study [4] and the detailed information was provided in Additional Files and Protocol.io.

Genome-wide identification of genes involved in the lignin biosynthetic pathway

The five genome sequences of *A. thaliana* (TAIR10), *B. distachyon* (v3.1), *O. sativa* (v7.0), *Populus trichocarpa* (JGI2.0.31), and *S. bicolor* (v3.1) were downloaded from the ENSEMBL database [59]. According to our literature-based investigations, 140 genes from the lignin biosynthetic pathway was experimentally validated from previous studies (Additional Table S28), and then, these known genes were collected and used as the query sequences for further identification. We identified lignin biosynthetic genes using a BLAST search and domain analysis as described in the previous article[60]. Briefly, we performed standard nucleotide BLAST searches (version 2.2.26) against the six genome sequences including moso bamboo using the coding sequence of the known genes with the following cut-off values: E-value $<1e^{-10}$; identity >95%; and coverage rate >40% query sequence. The filtered sequences were subsequently analyzed by hmmsearch (version 3.1b2) using the Pfam-A.hmm database (released 2017/03/31). Sequences with incomplete domains were discarded after? manual correction. Phylogenetic analyses were carried out following [4].

Positive selection analysis

We performed a positive selection analysis on the coding sequences of the lignin biosynthetic pathway genes. In each family, protein sequences were first aligned by PROBCONS (version 1.12) [61] using the default parameters, except for the option of iterative refinement, for which we used 1,000 iterations. Then, we back-translate? the protein alignment to its corresponding coding sequences. After obtaining the conserved blocks from the sequence alignment using Gblocks (version 0.91b) [62], jModelTest (version 2.1.6) [63] was used to find the best model according to the Bayesian Information Criterion. Subsequently, PhyML (version 3.0) [64] was used to reconstruct the phylogenetic tree under the best model, with bootstrapping of 1,000 replicates. Finally, certain branches selected from the phylogenetic tree were examined in a positive selection analysis using PAML (version 4.8) [65] with a branch-site model.

Availability of data and materials

Short-read sequencing data from this whole-genome shotgun project can be deposited at European Molecular Biology Laboratory (EMBL) under the accession ERP001340. RNA-Seq raw sequence data and Iso-Seq raw sequence data for a mixture sample were deposited in NCBI Short Read Archive database under the accession numbers: SRX2408703-28 and SRR7032261-69, respectively. The chromosome-level genome and the latest annotation were provided in *Giga*DB. Additionally, protocols to the methods are uploaded to Protocols.io (https://www.protocols.io/researchers/hansheng-zhao).

Declarations

Author's Contribution

Experimental design: H.Z., Z.G., C.C., B.F. S.W., Z.C., H.Y. and Z.J. Experimental preformation: H.Z., J.W.; H.Z., L.C., Z.X., C.Z. and Y.W. Data analysis: W.Y., H.S., L.L., S.W., Y.Y., Y.L., Q.G., C.C., X.C. and H.X. The providing of reagents, materials and analysis tools: H.Z. and Z.G. Article writing: H.Z., Z.C., Z.G. and B.F. All of the authors read and approved the final manuscript.

Competing interests

The authors declare that they have no competing interests.

Acknowledgements

This work received financial support from the Special Fund for Forest Scientific Research in the Public Welfare from State Forestry Administration of China (No. 201504106), and the Sub-Project of National Science and Technology Support Plan of the Twelfth Five-Year in China (No. 2015BAD04B03 and No. 2015BAD04B01).

References

1. Zhao H, Zhao S, International Network for Bamboo and Rattan, Fei B, Liu H, Yang H, et al.

Announcing the Genome Atlas of Bamboo and Rattan (GABR) project: promoting research in evolution and in economically and ecologically beneficial plants. GigaScience. 2017;6:1–7.

2. Bai Y-Y, Xiao L-P, Shi Z-J, Sun R-C. Structural variation of bamboo lignin before and after ethanol organosolv pretreatment. International Journal of Molecular Sciences. 2013;14:21394–413.

3. Jiang Z. Bamboo and Rattan in the World. Beijing: China Forestry Publishing House.

4. Peng Z, Lu Y, Li L, Zhao Q, Feng Q, Gao Z, et al. The draft genome of the fast-growing non-timber forest species moso bamboo (*Phyllostachys heterocycla*). Nature Genetics. 2013;45:456–61.

5. Filichkin SA, Priest HD, Givan SA, Shen R, Bryant DW, Fox SE, et al. Genome-wide mapping of alternative splicing in *Arabidopsis thaliana*. Genome research. 2010;20:45–58.

6. Pan Q, Shai O, Lee LJ, Frey BJ, Blencowe BJ. Deep surveying of alternative splicing complexity in the human transcriptome by high-throughput sequencing. Nature Genetics. 2008;40:1413–5.

7. Wang ET, Sandberg R, Luo S, Khrebtukova I, Zhang L, Mayr C, et al. Alternative isoform regulation in human tissue transcriptomes. Nature. 2008;456:470–6.

8. Zhang PG, Huang SZ, Pin A-L, Adams KL. Extensive divergence in alternative splicing patterns after gene and genome duplication during the evolutionary history of Arabidopsis. Molecular Biology and Evolution. 2010;27:1686–97.

9. Marquez Y, Brown JWS, Simpson C, Barta A, Kalyna M. Transcriptome survey reveals increased complexity of the alternative splicing landscape in Arabidopsis. Genome research. Cold Spring Harbor Lab; 2012;22:1184–95.

10. Shen Y, Zhou Z, Wang Z, Li W, Fang C, Wu M, et al. Global dissection of alternative splicing in paleopolyploid soybean. Plant Cell. 2014;26:996–1008.

11. Mandadi KK, Scholthof K-BG. Genome-wide analysis of alternative splicing landscapes modulated during plant-virus interactions in *Brachypodium distachyon*. Plant Cell. 2015;27:71–85.

12. Li Q, Xiao G, Zhu Y-X. Single-nucleotide resolution mapping of the *Gossypium raimondii* transcriptome reveals a new mechanism for alternative splicing of introns. Molecular Plant. 2014;7:829–40.

13. Thatcher SR, Zhou W, Leonard A, Wang B-B, Beatty M, Zastrow-Hayes G, et al. Genome-wide analysis of alternative splicing in *Zea mays*: landscape and genetic regulation. Plant Cell. 2014;26:3472–87.

14. Zhang G, Guo G, Hu X, Zhang Y, Li Q, Li R, et al. Deep RNA sequencing at single base-pair resolution reveals high complexity of the rice transcriptome. Genome research. 2010;20:646–54.

15. Rühl C, Stauffer E, Kahles A, Wagner G, Drechsel G, Rätsch G, et al. Polypyrimidine tract binding protein homologs from Arabidopsis are key regulators of alternative splicing with implications in fundamental developmental processes. Plant Cell. 2012;24:4360–75.

16. Staiger D, Brown JWS. Alternative splicing at the intersection of biological timing, development, and stress responses. Plant Cell. 2013;25:3640–56.

17. Li W, Lin W-D, Ray P, Lan P, Schmidt W. Genome-wide detection of condition-sensitive alternative splicing in Arabidopsis roots. Plant Physiology. 2013;162:1750–63.

18. Cui P, Zhang S, Ding F, Ali S, Xiong L. Dynamic regulation of genome-wide pre-mRNA splicing and stress tolerance by the Sm-like protein LSm5 in Arabidopsis. Genome Biology. 2014;15:R1.

19. Reddy ASN. Alternative splicing of pre-messenger RNAs in plants in the genomic era. Annual Review Plant Biology. 2007;58:267–94.

20. Barbosa-Morais NL, Irimia M, Pan Q, Xiong HY, Gueroussov S, Lee LJ, et al. The Evolutionary Landscape of Alternative Splicing in Vertebrate Species. Science. 2012;338:1587–93.

21. Keren H, Lev-Maor G, Ast G. Alternative splicing and evolution: diversification, exon definition and function. Nature Reviews Genetics. 2010;11:345–55.

22. Roy SW, Irimia M. Splicing in the eukaryotic ancestor: form, function and dysfunction. Trends in Ecology Evolution. 2009;24:447–55.

23. Chen RY, Li XL, Song WQ, Liang GL, Zhang PX, Lin RS, et al. Chromosome atlas of major economic plants genome in China. Tomus 4. Chromosome atlas of various bamboo species. Beijing: Science Press xxx, 646p.-illus.. ISBN 7030108353 Ch, En Chromosome numbers. Geog= 0 Systematics: ANGIOSPERMAE (GRAMINEAE)(KR, 200303867), 2003.

24. Peng Z, Lu T, Li L, Liu X, Gao Z, Hu T, et al. Genome-wide characterization of the biggest grass, bamboo, based on 10,608 putative full-length cDNA sequences. BMC plant biology. 2010;10:116.

25. Simão FA, Waterhouse RM, Ioannidis P, Kriventseva EV, Zdobnov EM. BUSCO: assessing genome assembly and annotation completeness with single-copy orthologs. Bioinformatics. 2015;31:3210–2.

26. Sneddon TP, Li P, Edmunds SC. GigaDB: announcing the GigaScience database. GigaScience. 2012;1:11.

27. Zhang YE, Vibranovski MD, Landback P, Marais GAB, Long M. Chromosomal redistribution of male-biased genes in mammalian evolution with two bursts of gene gain on the X chromosome. Barton NH, editor. PLoS Biol. 2010;8:e1000494.

28. Celotto AM, Graveley BR. Alternative splicing of the *Drosophila Dscam* pre-mRNA is both temporally and spatially regulated. Genetics. 2001;159:599–608.

29. Wang B-B, Brendel V. Genomewide comparative analysis of alternative splicing in plants. Proceedings of the National Academy of Sciences. 2006;103:7175–80.

30. Slotkin RK, Martienssen R. Transposable elements and the epigenetic regulation of the genome. Nature Reviews Genetics. 2007;8:272–85.

31. Feschotte C. Transposable elements and the evolution of regulatory networks. Nature Reviews Genetics. 2008;9:397–405.

32. Li Y, Li-Byarlay H, Burns P, Borodovsky M, Robinson GE, Ma J. TrueSight: a new algorithm for splice junction detection using RNA-seq. Nucleic Acids Research. 2013;41:e51–1.

33. Nilsen TW, Graveley BR. Expansion of the eukaryotic proteome by alternative splicing. Nature. 2010;463:457–63.

34. Barbazuk WB, Fu Y, McGinnis KM. Genome-wide analyses of alternative splicing in plants: opportunities and challenges. Genome research. 2008;18:1381–92.

35. Song X, Peng C, Zhou G, Gu H, Li Q, Zhang C. Dynamic allocation and transfer of non-structural carbohydrates, a possible mechanism for the explosive growth of Moso bamboo (*Phyllostachys heterocycla*). Scientific Reports. 2016;6.

36. Flagel LE, Wendel JF. Gene duplication and evolutionary novelty in plants. New Phytologist. 2009;183:557–64.

37. Lan X, Pritchard JK. Coregulation of tandem duplicate genes slows evolution of subfunctionalization in mammals. Science. 2016;352:1009–13.

38. Zhang W, Landback P, Gschwend AR, Shen B, Long M. New genes drive the evolution of gene interaction networks in the human and mouse genomes. Genome Biology. 2015;16:202.

39. Kim E, Magen A, Ast G. Different levels of alternative splicing among eukaryotes. Nucleic Acids Research. 2007;35:125–31.

40. Nakai K, Sakamoto H. Construction of a novel database containing aberrant splicing mutations of mammalian genes. Gene. 1994;141:171–7.

41. Chen M, Manley JL. Mechanisms of alternative splicing regulation: insights from molecular and genomics approaches. Nature Review Molecular Cell Biology. 2009;10:741–54.

42. Licatalosi DD, Darnell RB. RNA processing and its regulation: global insights into biological networks. Nature Reviews Genetics. Nature Publishing Group; 2010;11:75–87.

43. Martone PT, Estevez JM, Lu F, Ruel K, Denny MW, Somerville C, et al. Discovery of lignin in seaweed reveals convergent evolution of cell-wall architecture. Current Biology. 2009;19:169–75.

44. Taylor JS, Raes J. Duplication and divergence: the evolution of new genes and old ideas. Annual Review of Genetics. 2004;38:615–43.

45. Li X, Bonawitz ND, Weng J-K, Chapple C. The growth reduction associated with repressed lignin biosynthesis in Arabidopsis thaliana is independent of flavonoids. Plant Cell. 2010;22:1620–32.

46. Dudchenko O, Batra SS, Omer AD, Nyquist SK, Hoeger M, Durand NC, et al. *De novo* assembly of the Aedes aegypti genome using Hi-C yields chromosome-length scaffolds. Science. 2017;356:92–5.

47. Teytelman L, Stoliartchouk A, Kindler L, Hurwitz BL. Protocols.io: Virtual Communities for Protocol Development and Discussion. PLoS Biology. 2016;14:e1002538.

48. Zhao H, Sun H, Li L, Lou Y, Li R, Qi L, et al. Transcriptome-based investigation of cirrus development and identifying microsatellite markers in rattan (*Daemonorops jenkinsiana*). Scientific Reports. 2017;7:46107.

49. DeLuca DS, Levin JZ, Sivachenko A, Fennell T, Nazaire M-D, Williams C, et al. RNA-SeQC: RNA-seq metrics for quality control and process optimization. Bioinformatics. 2012;28:1530–2.

50. Bolger AM, Lohse M, Usadel B. Trimmomatic: a flexible trimmer for Illumina sequence data. Bioinformatics. 2014;30:2114–20.

51. Kim D, Langmead B, Salzberg SL. HISAT: a fast spliced aligner with low memory requirements. Nature Methods. 2015;12:357–60.

52. Trapnell C, Williams BA, Pertea G, Mortazavi A, Kwan G, van Baren MJ, et al. Transcript assembly and quantification by RNA-Seq reveals unannotated transcripts and isoform switching during cell differentiation. Nature Biotechnology. 2010;28:511–5.

53. Foissac S, Sammeth M. Analysis of alternative splicing events in custom gene datasets by AStalavista. Methods in Molecular Biology. 2015;1269:379–92.

54. Foissac S, Sammeth M. ASTALAVISTA: dynamic and flexible analysis of alternative splicing events in custom gene datasets. Nucleic Acids Research. 2007;35:W297–9.

55. Bauer S, Grossmann S, Vingron M, Robinson PN. Ontologizer 2.0--a multifunctional tool for GO term

enrichment analysis and data exploration. Bioinformatics. 2008;24:1650–1.

56. Marques AC, Tan J, Lee S, Kong L, Heger A, Ponting CP. Evidence for conserved post-transcriptional roles of unitary pseudogenes and for frequent bifunctionality of mRNAs. Genome Biology. 2012;13:R102.

57. Winter EE, Goodstadt L, Ponting CP. Elevated rates of protein secretion, evolution, and disease among tissue-specific genes. Genome research. 2004;14:54–61.

58. Wang B, Tseng E, Regulski M, Clark TA, Hon T, Jiao Y, et al. Unveiling the complexity of the maize transcriptome by single-molecule long-read sequencing. Nature Communications. 2016;7:11708.

59. Kersey PJ, Allen JE, Allot A, Barba M, Boddu S, Bolt BJ, et al. Ensembl Genomes 2018: an integrated omics infrastructure for non-vertebrate species. Nucleic Acids Research. 2018;46:D802–8.

60. Fischer S, Brunk BP, Chen F, Gao X, Harb OS, Iodice JB, et al. Using OrthoMCL to assign proteins to OrthoMCL-DB groups or to cluster proteomes into new ortholog groups. Current Protocol of Bioinformatics. Hoboken, NJ, USA: John Wiley & Sons, Inc; 2011;Chapter 6:Unit6.12.1–19.

61. Roshan U. Multiple sequence alignment using Probcons and Probalign. Methods in Molecular Biology. 2014;1079:147–53.

62. Talavera G, Castresana J. Improvement of phylogenies after removing divergent and ambiguously aligned blocks from protein sequence alignments. Systematic Biology. 2007;56:564–77.

63. Darriba D, Taboada GL, Doallo R, Posada D. jModelTest 2: more models, new heuristics and parallel computing. Nature Methods. 2012;9:772–2.

64. Guindon S, Dufayard J-F, Lefort V, Anisimova M, Hordijk W, Gascuel O. New algorithms and methods to estimate maximum-likelihood phylogenies: assessing the performance of PhyML 3.0. Systematic Biology. 2010;59:307–21.

65. Yang Z. PAML 4: phylogenetic analysis by maximum likelihood. Molecular Biology and Evolution. 2007;24:1586–91.

Figure legends:

Figure 1. The comparative results based on two versions of the moso bamboo genome.

(A) The distribution of the contigs between two versions of the moso bamboo genome. Contig N50 and N90 were marked. (B) The distribution of the scaffolds between two versions of the moso bamboo genome. Scaffold N50 and N90 were marked. (C) Bbox plots comparing the two versions of the moso bamboo genome, including gene length, intron length, CDS length, cDNA length, single exon length, and single intron length. (D) The BUSCO assessment result. Annotation v1 was based on the version 1 of the moso bamboo genome. Annotation v2.1 and Annotation v2.2 were based on the version 2, and Annotation v2.2 was the manually verified version of Annotation v2.1.

Figure 2. The distribution of AS genes and events and their correlation

(A) The distribution of AS genes in bamboo, including the four main types and Iso-Seq result. (B) The distribution of AS events in bamboo, including four main types and Iso-Seq result. (C) the correlation between AS genes and events was provided. IR, A3SS, A5SS, and ES represents intron retention, alternative 3' splice site donor, alternative 5' splice site acceptor, and exon skipping, respectively.

Figure 3. The evolutionary analysis in plants across bamboo

(A) The phylogenetic relationship of *Amborella trichopoda*, *Elaeis guineensis*, *Arabidopsis thaliana*, *Brachypodium distachyon*, *Oryza sativa*, *Spirodela polyrhiza*, *Sorghum bicolor* and *Ph. edulis*. Phylogenetic tree of the selected 8 plant species with branches leading to bamboo as red line. The notation indicates the eight orthologous gene datasets (D8-D1) identified in our study. (B) a Venn diagram of orthologous genes in related eight species was exhibited. (C) AS percentage and AS type were provided in D1 to D8 datasets, including redundant/non-redundant and multi-copy/single-copy. (D) increasing AS abundance and the decreasing specificity were displayed from D1 to D8.

Figure 4. The gene family expansion and AS abundance of bamboo in lignin biosynthetic pathway

A) A total of 13 families in lignin biosynthesis pathway were identified using six genomes of *A. thaliana*, *B. distachyon*, *O. sativa*, *Ph. edulis*, *P. trichocarpa*, and *S. bicolor*. Copy number and genes under positive selection were added. B) The structure, distribution and types of AS and related gene expression level were exhibited in six gene families (4CL, C3H, CCR, HCT, LAC, and POD). The lignin biosynthetic enzymes

are: PAL phenylalanine ammonia-lyase; TAL tyrosine ammonia-lyase; C4H cinnamate 4-hydroxylase; C3H 4-hydroxycinnamate 3-hydroxylase; COMT caffeic acid 3-O-methyltransferase; F5H ferulate 5-hydroxylase; 4CL 4-coumarate: CoA ligase; CCoA-3H coumaroyl-coenzyme A 3-hydroxylase; CCoA-OMT caffeoyl-coenzyme A O-methyltransferase; CCR cinnamoyl-CoA reductase; CAD cinnamyl alcohol, and HCT dehydrogenase hydroxycinnamoyl transferase.

Table 1. Statistics for the assembly of the moso bamboo genome using different sequence data

**Statistics**	**WGS assembly**		**Hi-C assembly**	
	Scaffold	Contig	Scaffold	Contig
Total number	19,285	76,900	19,684	84,758
Genome size (bp)	1,908,074,089	1,795,528,836	1,907,603,590	1,795,510,437
Gap number (bp)	112,545,253	0	112,093,153	0
Average length (bp)	98,940.84	23,348.88	96,911.38	21,183.96
N50 length (bp)	894,858	54,955	79,898,979	53,293
N90 length (bp)	115,487	11,757	44,603,463	10,445
Maximum length (bp)	5,406,526	738,589	137,299,170	738,589
Minimum length (bp)	926	157	318	1
GC content (%)	44.2	44.2	44.2	44.2
