# Supplementary material for: Chromosome-level reference genome and alternative splicing atlas of moso bamboo (Phyllostachys edulis)
Source: Gigascience. 2018 Sep 8;7(10):giy115. doi: 10.1093/gigascience/giy115 (PMC6204424; doi:10.1093/gigascience/giy115)
Supplement: Supplemental Files [file giy115_supplemental_files.zip › Additional File-Revised2-30Aug-zhs.docx]

# *Additional Files*

**Chromosome-level reference genome and alternative splicing atlas of moso bamboo (*Phyllostachys edulis*)**

Hansheng Zhao^1#^, Zhimin Gao^1#^, Le Wang^2,3#^, Jiongliang Wang^1^, Songbo Wang^4^, Benhua Fei^1^, Chunhai Chen^2^, Chengcheng Shi^5^, Xiaochuan Liu^5^, Hailin Zhang^2^, Yongfeng Lou^1^, LianFu Chen^1^, Huayu Sun^1^, Xianqiang Zhou^2^, Sining Wang^1^, Chi Zhang^2^, Hao Xu^1^, Lichao Li^1^, Yihong Yang^1^, Yanli Wei^2^, Wei Yang^2^, Qiang Gao^2^, Huanming Yang^2^, Shancen Zhao^4+^ and Zehui Jiang^1+^

^1^ State Forestry Administration Key Open Laboratory on the Science and Technology of Bamboo and Rattan, Institute of Gene Science for Bamboo and Rattan Resources, International Center for Bamboo and Rattan, Futongdong Rd, WangJing, Chaoyang District Beijing 100102, China;

^2^ BGI Genomics, BGI-Shenzhen, Building NO.7, BGI Park, No. 21 Hongan 3rd Street, Yantian District, Shenzhen 518083, China;

^3^ Department of Plant Sciences, University of California, Davis, One Shield Avenue, Davis, CA 95617, USA;

^4^ BGI Institute of Applied Agriculture, BGI-Shenzhen, No.7 PengFei Rd, Dapeng District, Shenzhen 518120, China;

^5^ BGI-Qingdao, No. 2877, Tuanjie Rd, Sino-German Ecopark, Qingdao, Shandong Province, 266555, China.

^+ Co-corresponding author: zhaoshancen@genomics.cn and jiangzehui@icbr.ac.cn^

^# These authors contributed equally to this work.^

Additional Note

S1. Genome sequencing and assembly

## S1.1 Sequenced material

Moso bamboo (*Phyllostachys edulis*) is a large woody bamboo with the highest ecological, economic, and cultural values of all bamboos in Asia and accounting for ~70% of total area of bamboo growth and 5 billion US dollars of annual forest production in China [1,2]. The moso bamboo genome contains 24 pairs of chromosomes (2n=48) and was characteristic of a diploid [2]. For the moso bamboo genome sequencing project, the material was obtained from the Tianmu-Mountain National Nature Reserve in Zhejiang Province of Eastern China (N:30º19'13.4", E:119º26'55.0"; 480 M), because its growth had not been interrupted by human activities for a long time. The genomic DNA was extracted from the leaves in a hydroponic system using Hoagland′s solution (Hoagland and Arnon 1950) by a standard CTAB method (Doyle and Doyle 1987).

## S1.2 WGS library construction

We have adopted the Illumina sequencing platform based on whole genome sequencing (WGS) strategy. According to the standard manufacturer’ protocol of Illumina, 42 paired-end (PE) libraries and 18 mated-end (ME) libraries were constructed with short insert size (350 to 400 bp) and long insert size (3, 8 and 15 kb), respectively (**Additional Table S1**). Then, the PE sequencing was performed after the library construction based on the workflow: cluster generation, template hybridization, isothermal amplification, linearization, blocking, denaturation, and hybridization of sequencing primers. Subsequently, the base-calling pipeline (Solexa Pipeline-0.3) was applied to detect bases from the raw fluorescent images. In total, we obtained genomic DNA sequencing data from all 60 libraries, except for one Hi-C library (**Additional Table S1**). The details of Hi-C were provided in the following section.

## S1.3 Quality control of WGS data

As an essential step in data preprocessing, the raw data were filtered using the two software with the default parameters, i.e., NGS QC Toolkit (version 2.3.3) [3] and FastUniq (version 1.1) [4]. For NGS QC Toolkit software,

1) Removing adapters

to remove the reads contaminated by adapters using the ‘TrimmingReads.pl’ script;

2) Trimming

to trim continuous low-quality bases (Q<=13) on both 5’ and 3’ ends and to remove the reads with more than 40% low-quality bases (Q<=13) using the ‘IlluQC_PRLL.pl’ script;

3) Removing low-quality reads

to remove the reads that contain Ns>5% of the read length using the ‘AmbigutyFiltering.pl’ script;

4) Removing duplicated reads

FastUniq (version 1.1, http://sourceforge.net/projects/fastuniq/) was used to remove the duplicated reads caused by PCR amplification.

In total, a total of 373.3 Gb high-quality data (clean data) from 446.7 Gb raw data, corresponding to ~173× coverage of the moso bamboo genome after removing the low-quality reads (**Additional Table S1**).

## S1.4 WGS Assembly

As an genome assembler, ALLPATHS-LG (version r52488) [5] was mainly used with the default parameters and it was applied on the *de novo* assembly for the large genomes using short reads. The briefly introduction of the assembly procedure in our study was as follows:

1) Calculating insert size

For detecting insert sizes and their standard deviations in each library, Bowtie 2 (version 2.2.9 http://bowtie-bio.sourceforge.net/bowtie2) and Picard (version 2.7 http://broadinstitute.github.io/picard/) were used with the default parameter. The results, thus, were attached in **Additional Table S1**.

2) Constructing contigs and scaffolds

As two indispensable configuration files of ALLPATHS-LG, ′in_groups.csv′ and ′in_libs.csv′ were constructed according to the manual of ALLPATHS-LG and the information of insert sizes in each library. Then, ALLPATHS-LG was performed to construct contigs and scaffolds.

3) Linking the scaffolds

For effectively linking scaffolds based on ME reads and BAC data, SSPACE (version 1 http://www.baseclear.com/genomics/bioinformatics/basetools/SSPACE) with the default parameters was used only once.

4) Filling the gaps

For filling the gaps inside the scaffolds, two software were used with the default parameters, i.e*.*, GapFiller (version 1.11) [6] and GapCloser (version 1.12) [7] and they need two input files, the library.txt and config.txt. We calculated them based on the DNA libraries information of the moso bamboo genome and the manual of ALLPATHS-LG.

Thus, the entire scaffolds were reserved in final assembly because the length of all scaffolds was more than 500 bp. The detailed steps were provided in the previous studies [8,9]. In sum, as shown in **Additional Table S8**, the contig length reached ~1.80 Gb with an N50 length of ~55 kb, and the genome assembly was ~1.91 Gb with a scaffold N50 length of ~894 kb. The scaffold more than 100 kb accounted for more than 91.04% of the assembly (**Additional Table S9**). Moreover, the single-base accuracy of the genome was verified by aligning the genomic reads against the assembly using SOAP2 [7] with the default parameters.

S1.5 Hi-C library preparation, sequencing and assembling

Fresh moso bamboo leaves were cut into 10 mm fragments then fixed in 1% formaldehyde (SIGMA, St. Louis, USA) and the reaction was stopped with glycine (SIGMA, St. Louis, USA). The leaves were grounded to fine powder in liquid nitrogen. To destroy the cell wall, the formaldehyde fixed powder was added BufferI solution (0.4M sucrose (SIGMA, St. Louis, USA), 10mM Tris-HCl pH8(SIGMA, St. Louis, USA)), BufferII solution (Sucrose 0.25 M, 10mM Tris-HCl pH8, l5mM 1% Triton X-100(SIGMA, St. Louis, USA)) and BufferIII solution (1.7M sucrose,10mM Tris-HCl pH8, 0.15% Triton X-100) in order. The pellet obtained after centrifugation is the cross-linked nucleus. Then, the restriction enzyme (Mbo I) (New England BioLabs, Beverly, MA, USA) was added to digest the DNA, followed by repairing the 5′ overhang (10 mM dCTP, 10 mM dGTP, 10 mM dTTP, (Invitrogen, Carlsbad, CA) 5U/μl DNA Polymerase I, Large (Klenow) Fragment (New England BioLabs, Beverly, MA, USA)) using a biotinylated residue (0.4 mM biotin-14-dATP(Invitrogen, Carlsbad, CA)), and the resulting blunt-end fragments were ligated in situ (10X NEB T4 DNA ligase buffer (New England BioLabs, Beverly, MA, USA), 10% Triton X-100, 10 mg/ml BSA (New England BioLabs, Beverly, MA, USA), T4 DNA ligase (New England BioLabs, Beverly, MA, USA)). Finally, the isolated DNA was reverse-crosslinked (Add 10 mg/ml proteinase K (New England BioLabs, Beverly, MA, USA) and 0.5 ml 2% CTAB (AMBION, Waltham, USA) to the tube and incubate at 56**°** for overnight) and purified (put the Reverse-crosslinked DNA liquid into three tube equally, add equal volume of Chloroform and Isoamyl alcohol (24:1) (SIGMA, St. Louis, USA) mixture to each tube, spin down 5min, the upper aqueous phase was obtained by centrifuge and precipitation using isopropyl alcohol for 2 hours, then precipitation, wash the precipitate twice with 1 ml of freshly made 70% ethanol (SINOPHARM, Shanghai, China), air-dry the precipitate completely and re-suspend the precipitate in 30 µl of ddH2O). The Hi-C library was created by shearing 20 μg of DNA and capturing the biotin-containing fragments on streptavidin-coated beads using Dynabeads MyOne Streptavidin T1 (Invitrogen, Carlsbad, CA). DNA fragment end repair (10X NEB T4 DNA ligase buffer with 10 mM ATP (New England BioLabs, Beverly, MA, USA), 25 mM dNTP mix (ENZYMATICS, Beverly, USA), 10 U/μl NEB T4 PNK (New England BioLabs, Beverly, MA, USA), 3 U/μl NEB T4 DNA polymerase I New England BioLabs, Beverly, MA, USA), 5 U/μl NEB DNA polymerase I, Large (Klenow) Fragment (New England BioLabs, Beverly, MA, USA)), adenylation (10X NEBuffer 2 (New England BioLabs, Beverly, MA, USA), 10 mM dATP (Invitrogen, Carlsbad, CA), 5 U/μl NEB Klenow exo minus New England BioLabs, Beverly, MA, USA)), and adaptor ligation were performed using 10X T4 PNK Reaction Buffer(New England BioLabs, Beverly, MA, USA), 100mM ATP(FERMENTAS, USA), 600 U/ul T4 DNA Ligase (New England BioLabs, Beverly, MA, USA), 50% PEG8000 (RIGAKU, Tokyo, Japan), 50 uM Ad153 barcode oligo_2B mix (BGI, Shenzhen, China), and followed by PCR (95℃ 3 min.; [98℃ 20 sec., 60℃ 15 sec., 72℃ 15 sec.] (8 cycles); 72℃ 10 min.). Following PCR, the standard circularization step required for BGISEQ-500 was carried out and DNB were prepared as previously described [10].

The Hi-C library was sequenced on BGISEQ-500 platform with 100 bp PE reads. Raw data produced by Hi-C library were evaluated and qualified with HiC-Pro (version 2.8.0_devel) [11]. Then the valid reads together with the WGS assembly were processed by 3D-DNA pipeline (version 170123) [12] to anchor the scaffolds onto chromosomes (**Additional Fig. S1**). The contact map was visualized by Juicerbox (version 1.5.2) [13]. To evaluate the Hi-C assembly, the chromosome sequences of moso bamboo were aligned with rice (*Oryza sativa*) genome using Lastz (version 1.02.00) [14] with the parameters: T=2 C=2 H=2000 Y=3400 L=6000 K=2200. Syntenic blocks longer than 2kb were plotted using Circos (version 0.69) [15] (**Additional Fig. S2**). The chromosomes length of the Hi-C assembly and the collinearity between the moso bamboo and rice chromosomes were provided in **Additional Tables S10-11.**

## S1.6 Assembly evaluation

We performed the compared analyses with BAC, EST and known genome sequences to evaluate the chromosome-level assembly quality of the moso bamboo genome. First, according to our 8 BAC libraries deposited at the European Molecular Biology Laboratory (EMBL) (accession number: FO203436, FO203437, FO203439, FO20341, FO203443, FO203434, FO203447 and FO203448), the alignment result shown all BAC sequences were mapping to 10 scaffolds completely or partially, on average, with 93.73% coverage by BLATN with a cutoff of E-value<1e^-10^ (**Additional Fig. S3** and **Additional Table S12**). Second, according to our 10,608 full-length cDNAs descripted previously [16], the alignment using BLAT (version 3.2.1) [17] with the default parameters showed ~87.94% were mapped to 9,329 scaffolds with >=95% of identify and >= 99% of coverage (**Additional Table S13**). Last, fifteen mRNA sequences in GenBank were compared to the assembled genome. As shown in **Additional Table S14**, the comparison of assembled genome and mRNA sequences indicated, on average, 98.81% of coverage. Taken together, the above evidences indicated that the chromosome-level genome of moso bamboo has a reliable quality and comprehensive coverage.

## S1.7 GC comparison

The distribution of GC content was analyzed by compared the new moso bamboo genome to other five grasses: *Brachypodium distachyon, Sorghum bicolor, Oryza sativa, Triticum Urartu* and *Zea mays*, using 500 bp non-overlapping sliding windows along the genomes to investigate the GC content. The results (**Additional Fig. S4**) indicated the peak of GC was distributed between 0.4 and 0.5. The big peak was 0.42 in the moso bamboo genome. Comparison with the small peaks appeared in *Z. may*, one was absented in moso bamboo. This result may associate with repetitive contents and distribution [8].

# S2 Genome Annotation

## S2.1 transcriptome sequencing and assembly

RNA-Seq data plays an essential role in assisting genome annotation. We collected 26 samples from 6 moso bamboo tissues (rhizome, root, shoot, leaf, sheaths, bud) during different developmental stages. Then, their samples were performed high-throughput transcriptome sequencing using the Illumina platform. These samples grown in six main bamboo producing areas in China during May in 2015, including:

1) YiXing, JiangSu Province (N:31º15′08.41″, E:119º43′42.55″, 212 M),

2) TianMu Mountain, ZheJiang Province (N:30º19′13.42″, E:119º26′55.21″, 480 M),

3) XianNing, HuBei Province (N:29º81′10.02″, E:114º31′21.12″ 150 M),

4) TaoJiang, HuHan Province (N:28º28′39.74″, E:112º11′18.62″, 320 M),

5) GuiLin, GuangXi Province (N:28º28′39.74″, E:112º11′18.62″, 216 M),

6) ChiShui, GuiZhou Province (N:28º28′15.27″, E:105º59′41.43″, 120 M).

The mixed bundle was quickly frozen in liquid nitrogen for RNA isolation. The total RNA was isolated from 6 tissues using TRIZOL Reagent Solution (Invitrogen, Carlsbad, CA, USA) based on the manufacturer’s instructions. The purity and concentration were detected using a NanoDrop 2000 spectrophotometer. Reverse transcription was conducted with Reverse Transcription System (Promage, USA). The extracted RNA was treated with RNase-free DNase I for 30 min at 37 ºC to remove the residual DNA. The cDNA library construction and normalization were performed as described previously [18]. Then, the pooled libraries were 2×100 bp by Illumina HiSeq 4000 platform (Illumina, San Diego, CA, USA). In sum, we obtained 378.64 Gb raw data from 26 libraries.

Trinity (version 2.0.6) [19], a transcriptomic assembler based on a given non-reference genome, was used to assemble transcriptomes to provide gene expressions in further annotation. Most default parameters of Trinity were used except for the two parameters (--normalize_reads –min_kmer_cov 2 –min_contig_length 400). As shown in **Additional Table S16**, the largest transcriptome size and the most transcripts were detected in the bud of rhizome, followed by sheath sheet. After merging the individual tissues transcriptomic data, we identified 633,279 transcripts and ~627.2 Mb in size.

## S2.2 Repeat annotation

Due to various repeated sequences in the assembled genome, we have firstly detected repeat sequences, including transposable elements (TEs) and tandem repeat sequences at both DNA and protein levels before the genome annotation. Tandem repeat sequences were identified using Tandem Repeat Finder (version 4.0.9) [20]. TEs were identified using two homolog-based methods, i.e., RepeatMasker (version 4.0.5) [21] and *de novo* methods, i.e., RepeatModeler (version 1.0.8, http://repeatmasker.org/RepeatModeler.html), respectively. For the homology approach, two databases of TE library, i.e., Repbase (version 15.02, http://www.girinst.org/repbase/) and TIGE (version 3.0), were used.

As shown in **Additional Table S15**, the results indicated the new moso bamboo genome comprised approximately 63.24% TEs, of which 45.67% were retrotransposons, 10.41% were DNA transposons and 7.16% were unclassified repetitive elements. Moreover, we aligned classified TE families to the consensus sequences in the Repbase library. The sequence divergence rate (**Additional Fig. S5**) was calculated between the identified TE elements in the genome by the homology-based method and the consensus sequence in the Repbase. The result indicated a numerous divergence rate of 30% was revealed, indicating that these TEs were derived ancient species.

## S2.3 Gene modeling and prediction

We integrated multiply approaches, i.e., *de novo* method, homology-based method, EST-based method, and RNA-Seq approach, to accurately predict gene models based on a previous report [8].

1) *De novo* prediction

The two *de novo* predictors based on self-trained models, Fgenesh (version 1) [22] and Augustus (version 3.3) [23], were used to predict protein-coding gene models in the moso bamboo genome. Via optimizing train data and multiply train, the result showed that 114,568 and 141,852 protein-coding gene models were predicted by Augustus and Fgenesh, respectively.

2) Homolog prediction

The six-grass species (*brachypodium distachyon*, *Oryza sativa*, *Setaria italic*, *Sorghum bicolor*, *Triticum aestivum*, and *Zea mays*) were used in the homolog prediction. Their protein sequences were download for ENSEMBL database, then these was mapped to the new moso bamboo genome using TBLASTN (e-value<=1e^-5^) and splicing patterns were accurately generated with GeneWise (version 2.0) [24]. The genome version of the six-grass species was provided in the main text.

3) EST prediction

We performed BLAT to align the ESTs to the new moso bamboo genome with >=98% of identify and >=95% of coverage. Then, we used PASA (version 2.0.2 http://pasapipeline.github.io/) to link the spliced alignments for accurate gene structures. We thus predicted 74,685 protein-coding gene models after filtering low-quality gene models.

4) RNA-Seq data

Similarly, RNA-Seq data, a kind of high-throughput expressed data, were mapped to the genome to identify exon-intron splicing junctions and refine the alignment of RNA-Seq reads to the genome, using HISAT2 (version 2.0.4) [25] was used to identify exon-intron splicing junctions and refine the alignment of RNA-Seq reads to the genome. Finally, we used Cufflinks (version 2.0.2) [26] to define a final set of 38,608 predicted protein-coding gene models.

Taken together, the above evidences were integrated with the method described in a previous study [8]. Totally, 51,523 genes were obtained as a consensus gene set. Moreover, using PASA with default parameters as well as PacBio data and full-length cDNA data, we refined the annotation and then finally obtained 51,661 protein-coding models. As shown in **Additional Fig. S6**, the distribution comparison of gene length, CDS length, intron length, and exon length of *Ph. edulis* to the five sequenced monocots. The comparison of gene numbers and features was provided in **Additional Table S18.** In **Additional Table S21**, only 2.78% of the predicted gene models was considered as unannotated genes by comparing with the seven databases of annotation (Nr [27], IntroPro [28], GO [29], COG [30], KEGG [31], Swiss-Prot [32], and TrEMBL [33]), suggesting the predicted gene models are reliable.

## S2.4 Analysis of non-coding RNAs and prediction of transcription factor

As non-coding RNA genes, the main four categories, i.e., tRNA, rRNA, miRNA and snRNA, were predicted based on the genome. As shown in **Additional Table S22**, the statistics of non-coding RNAs in moso bamboo were summarized. Moreover, transcription factors (TFs) play diverse roles in transcriptional regulation and signal transduction pathways of plant growth and development, detoxification, abiotic and biotic stresses-related response process [34,35]. As shown in **Additional Tables S23,** we have predicted 69 TF families and 3,497 TFs in *Ph. edulis* by iTAK web services (http://bioinfo.bti.cornell.edu/cgi-bin/itak/index.cgi), as well as compared with other grasses.

S3 Genome Evolution

## S3.1 Orthologous Gene and Phylogenetic

The identification of orthologous gene clusters was considered as a fundamental aspect of genome evolution. Single-copy gene families and multi-copy gene families were identified by orthMCL (version 2.0.9) [36] among *Ph. edulis* and other 7 plant species, including *Amborella trichopoda* (version 1.0) from Amborella Genome Database (amborella.huck.psu.edu), *Elaeis guineensis* (GCF_000442705.1) from NCBI database, *Arabidopsis thaliana* (TAIR10), *Brachypodium distachyon* (verison 3.1), *Oryza sativa* (version 7.0), *Spirodela polyrhiza* (version 2) and *Sorghum bicolor* (version 3.1) from the ENSEMBL database. The statistic of the gene family clustering in the 8 species was provided in **Additional Table S24**. The comparison of gene family clustering was provided in **Additional Fig. S7**. Afterwards, all single-copy genes were used to construct the phylogenetic tree by PhyML (version 3.0) [37] specifying a HKY85 substitution model with a gamma distribution across sites (**Additional Fig. S8**).

## S3.2 Phylogenetic Analyses

We constructed the phylogenetic tree of *Ph. edulis* and the other seven sequenced plant genomes using single-copy orthologous genes (**Additional Fig. S8**). The different molecular clocks (divergence rate) might be explained by the body size hypothesis or the generation-time hypothesis, which propose that the larger the body size is or the longer the generation-time is, the slower the molecular clock.

## S3.3 Estimation of Divergence Time

In order to estimate the divergence time between *Ph. edulis* and the other 7 sequenced plant genomes, a Bayesian relaxed molecular clock approach was used to estimate the divergence time using MCMCTREE in PAML (version 4) [38]. Calibration times were gained from a previous study [39] (*O. sativa vs. B. distachyon*: 40-54 Mya; *O. sativa* *vs. S. bicolor*: 45-60 Mya; *A. trichopoda* *vs.* *S. bicolor*: 119.7-199.3 Mya).

S4 Other analyses

The detailed descriptions of other analyses were provided in the main text and some supplementary tables and figures were presented in this Additional Files.

**Reference**

1. Jiang Z. Bamboo and Rattan in the World. Beijing: China Forestry Publishing House.

2. Peng Z, Lu Y, Li L, Zhao Q, Feng Q, Gao Z, et al. The draft genome of the fast-growing non-timber forest species moso bamboo (*Phyllostachys heterocycla*). Nature Genetics. 2013;45:456–61.

3. Patel RK, Jain M. NGS QC Toolkit: A Toolkit for Quality Control of Next Generation Sequencing Data. Liu Z, editor. PloS one. 2012;7:e30619.

4. Xu H, Luo X, Qian J, Pang X, Song J, Qian G, et al. FastUniq: a fast de novo duplicates removal tool for paired short reads. Doucet D, editor. PloS one. 2012;7:e52249.

5. Maccallum I, Przybylski D, Gnerre S, Burton J, Shlyakhter I, Gnirke A, et al. ALLPATHS 2: small genomes assembled accurately and with high continuity from short paired reads. Genome Biology. 2009;10:R103.

6. Nadalin F, Vezzi F, Policriti A. GapFiller: a *de novo* assembly approach to fill the gap within paired reads. BMC Bioinformatics. 2012;13 Suppl 14:S8.

7. Luo R, Liu B, Xie Y, Li Z, Huang W, Yuan J, et al. Erratum: SOAPdenovo2: an empirically improved memory-efficient short-read de novo assembler. GigaScience. 2015;4:30.

8. Ling H-Q, Zhao S, Liu D, Wang J, Sun H, Zhang C, et al. Draft genome of the wheat A-genome progenitor *Triticum urartu*. Nature. 2013;496:87–90.

9. Jia J, Zhao S, Kong X, Li Y, Zhao G, He W, et al. *Aegilops tauschii* draft genome sequence reveals a gene repertoire for wheat adaptation. Nature. 2013;496:91–5.

10. Drmanac R, Sparks AB, Callow MJ, Halpern AL, Burns NL, Kermani BG, et al. Human genome sequencing using unchained base reads on self-assembling DNA nanoarrays. Science. 2010;327:78–81.

11. Servant N, Varoquaux N, Lajoie BR, Viara E, Chen C-J, Vert J-P, et al. HiC-Pro: an optimized and flexible pipeline for Hi-C data processing. Genome Biology. 2015;16:259.

12. Dudchenko O, Batra SS, Omer AD, Nyquist SK, Hoeger M, Durand NC, et al. *De novo* assembly of the *Aedes aegypti* genome using Hi-C yields chromosome-length scaffolds. Science. 2017;356:92–5.

13. Zhao H, Dong L, Sun H, Li L, Lou Y, Wang L, et al. Comprehensive analysis of multi-tissue transcriptome data and the genome-wide investigation of GRAS family in Phyllostachys edulis. Scientific Reports. 2016;6:27640.

14. Harris RS. Improved pairwise alignment of genomic DNA. The Pennsylvania State University, 2007.

15. Krzywinski M, Schein J, Birol I, Connors J, Gascoyne R, Horsman D, et al. Circos: an information aesthetic for comparative genomics. Genome research. 2009;19:1639–45.

16. Peng Z, Lu T, Li L, Liu X, Gao Z, Hu T, et al. Genome-wide characterization of the biggest grass, bamboo, based on 10,608 putative full-length cDNA sequences. BMC plant biology. 2010;10:116.

17. Kent WJ. BLAT--the BLAST-like alignment tool. Genome research. 2002;12:656–64.

18. Zhao H, Sun H, Li L, Lou Y, Li R, Qi L, et al. Transcriptome-based investigation of cirrus development and identifying microsatellite markers in rattan (*Daemonorops jenkinsiana*). Scientific Reports. 2017;7:46107.

19. Haas BJ, Papanicolaou A, Yassour M, Grabherr M, Blood PD, Bowden J, et al. *De novo* transcript sequence reconstruction from RNA-seq using the Trinity platform for reference generation and analysis. Nature Protocol. 2013;8:1494–512.

20. Benson G. Tandem repeats finder: a program to analyze DNA sequences. Nucleic Acids Research. 1999;27:573–80.

21. Tempel S. Using and understanding RepeatMasker. Methods Mol. Biol. Totowa, NJ: Humana Press; 2012;859:29–51.

22. Solovyev V, Kosarev P, Seledsov I, Vorobyev D. Automatic annotation of eukaryotic genes, pseudogenes and promoters. Genome Biology. 2006;7 Suppl 1:S10.1–12.

23. Stanke M, Keller O, Gunduz I, Hayes A, Waack S, Morgenstern B. AUGUSTUS: *ab initio* prediction of alternative transcripts. Nucleic Acids Research. 2006;34:W435–9.

24. Birney E. GeneWise and Genomewise. Genome research. 2004;14:988–95.

25. Kim D, Langmead B, Salzberg SL. HISAT: a fast spliced aligner with low memory requirements. Nature Methods. 2015;12:357–60.

26. Ghosh S, Chan C-KK. Analysis of RNA-Seq Data Using TopHat and Cufflinks. Methods in Molecular Biology. 2016;1374:339–61.

27. O'Leary NA, Wright MW, Brister JR, Ciufo S, Haddad D, McVeigh R, et al. Reference sequence (RefSeq) database at NCBI: current status, taxonomic expansion, and functional annotation. Nucleic Acids Resarch. 2016;44:D733–45.

28. Finn RD, Attwood TK, Babbitt PC, Bateman A, Bork P, Bridge AJ, et al. InterPro in 2017-beyond protein family and domain annotations. Nucleic Acids Research. 2017;45:D190–9.

29. Gene Ontology Consortium. Gene Ontology Consortium: going forward. Nucleic Acids Research. 2015;43:D1049–56.

30. Tatusov RL, Galperin MY, Natale DA, Koonin EV. The COG database: a tool for genome-scale analysis of protein functions and evolution. Nucleic Acids Research. Oxford University Press; 2000;28:33–6.

31. Kanehisa M, Furumichi M, Tanabe M, Sato Y, Morishima K. KEGG: new perspectives on genomes, pathways, diseases and drugs. Nucleic Acids Research. 2017;45:D353–61.

32. Boutet E, Lieberherr D, Tognolli M, Schneider M, Bansal P, Bridge AJ, et al. UniProtKB/Swiss-Prot, the Manually Annotated Section of the UniProt KnowledgeBase: How to Use the Entry View. Methods in Molecular Biology. 2016;1374:23–54.

33. The UniProt Consortium. UniProt: the universal protein knowledgebase. Nucleic Acids Research. 2017;45:D158–69.

34. Tong H, Liu L, Jin Y, Du L, Yin Y, Qian Q, et al. DWARF and low-tilleing acts as a direct downstream target of a GSK3/SHAGGY-like kinase to mediate brassinosteroid responses in rice. Plant Cell. 2012;24:2562–77.

35. Zhang Z-L, Ogawa M, Fleet CM, Zentella R, Hu J, Heo J-O, et al. Scarecrow-like 3 promotes gibberellin signaling by antagonizing master growth repressor DELLA in Arabidopsis. Proceedings of the National Academy of Sciences of the United States of America. 2011;108:2160–5.

36. Chen F, Mackey AJ, Stoeckert CJ, Roos DS. OrthoMCL-DB: querying a comprehensive multi-species collection of ortholog groups. Nucleic Acids Research. 2006;34:D363–8.

37. Guindon S, Dufayard J-F, Lefort V, Anisimova M, Hordijk W, Gascuel O. New algorithms and methods to estimate maximum-likelihood phylogenies: assessing the performance of PhyML 3.0. Systematic Biology. 2010;59:307–21.

38. Yang Z. PAML 4: phylogenetic analysis by maximum likelihood. Molecular Biology and Evolution. 2007;24:1586–91.

39. International Brachypodium Initiative. Genome sequencing and analysis of the model grass *Brachypodium distachyon*. Nature. 2010;463:763–8.

Additional Figure


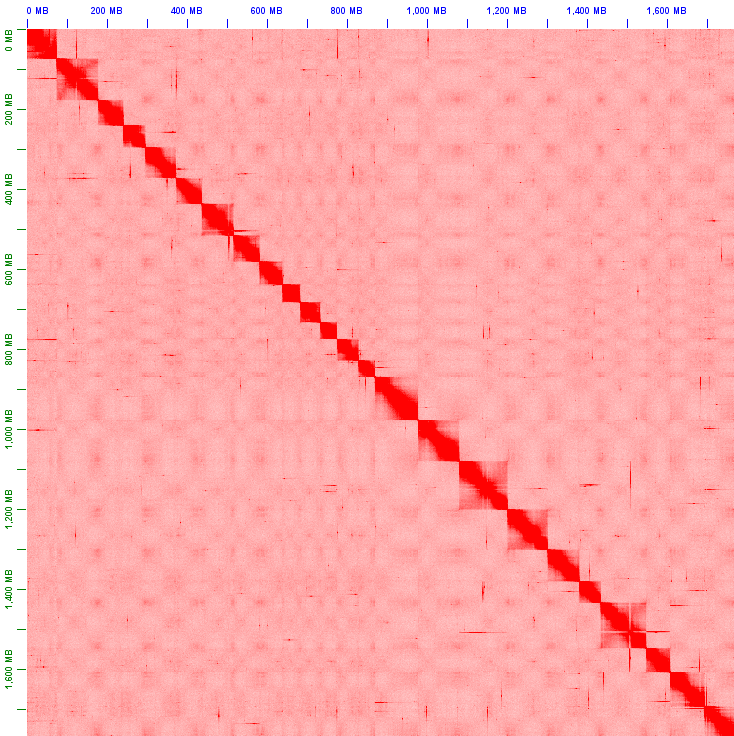


**Additional Fig. S1. Contact map of the Hi-C assembly**


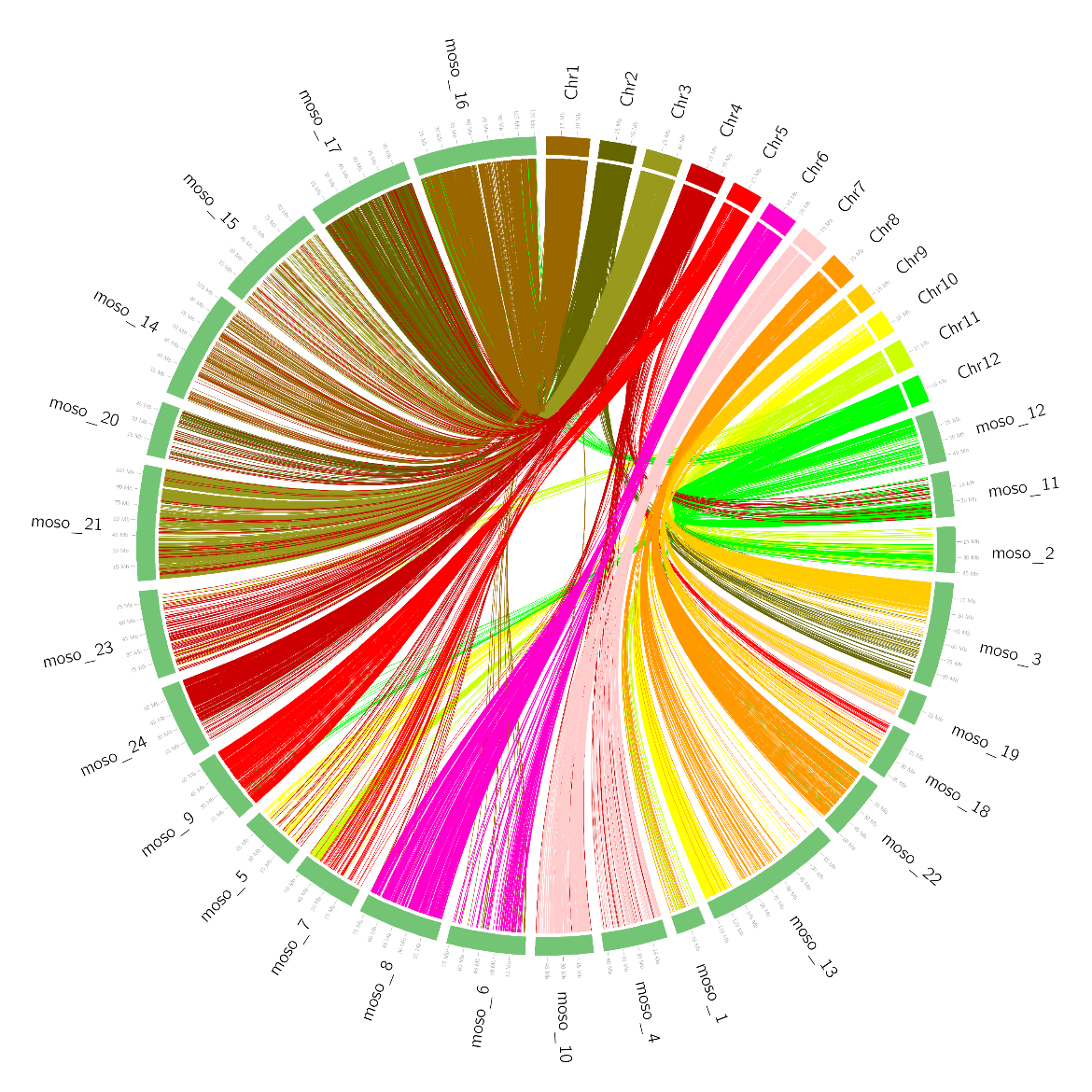


**Additional Fig. S2. Genome comparison between moso bamboo and rice.**

Green rectangles which labelled “moso" on the out ring of the circle represent the moso bamboo chromosomes, and rectangles of other colors which labelled “Chr” represent rice chromosomes


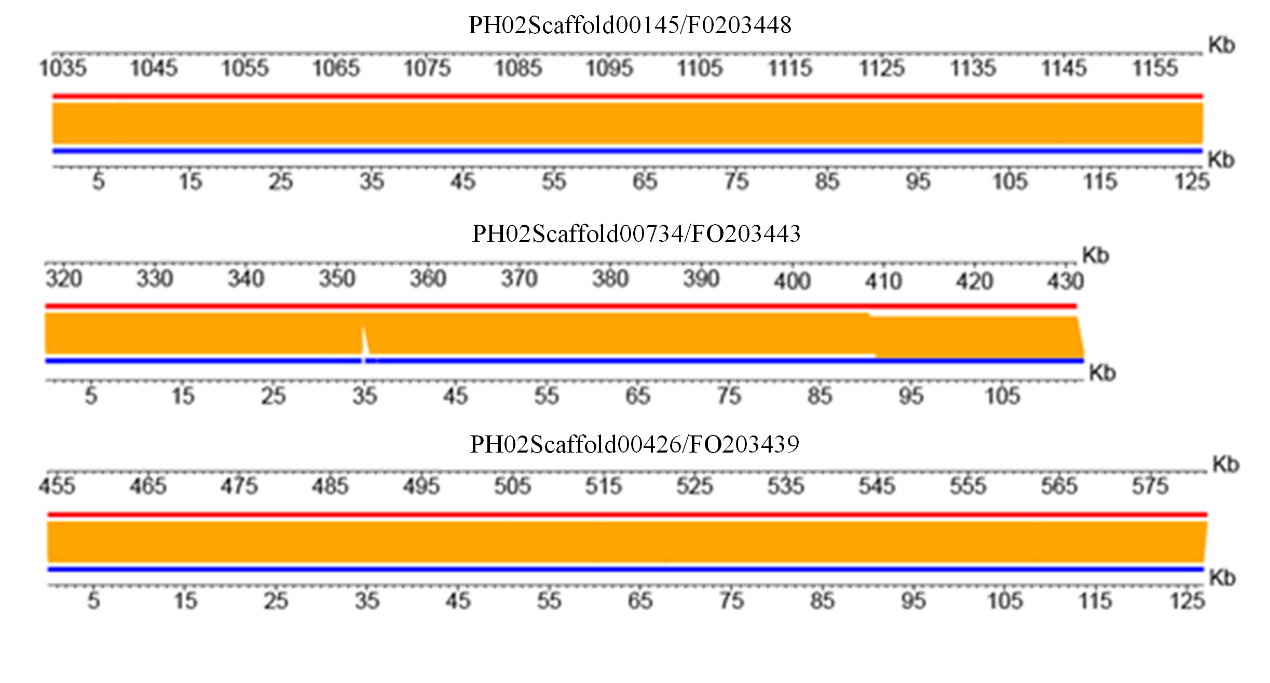


**Additional** **Fig. S3. Synteny block of the alignment between three BACs and the scaffold of *Ph. edulis*.**

In the comparison, the upper red lines indicate the moso bamboo scaffold, and the lower blue lines indicate the BAC sequences. The white boxes in the BAC represent ambiguous bases (Ns) and the yellow line represent well aligned sequences between the BAC and the sequences.


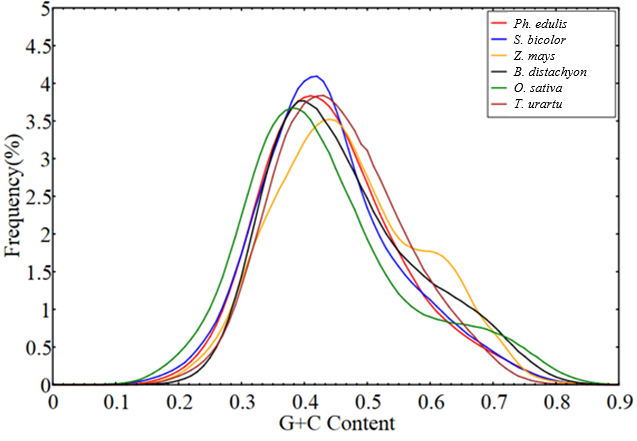


**Additional Fig. S4. Comparison of the GC content distribution among *Ph. edulis* and the other five grasses**


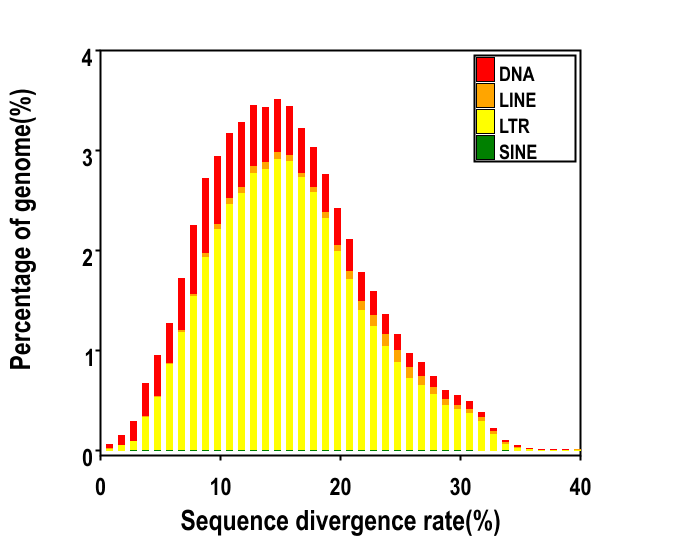


**Additional Fig. S5. The distribution of sequence divergence rate of different TE types in the *Ph. edulis* genome.**

DNA, DNA elements; LINE, long interspersed nuclear elements; LTR, long terminal repeat transposable elements; SINE. Short interspersed nuclear elements.

**Additional Fig. S6. The distribution comparison of (a) Gene length, (b) CDS length, (c) Intron length, and (d) Exon length of *Ph. edulis* to the five sequenced monocots.**


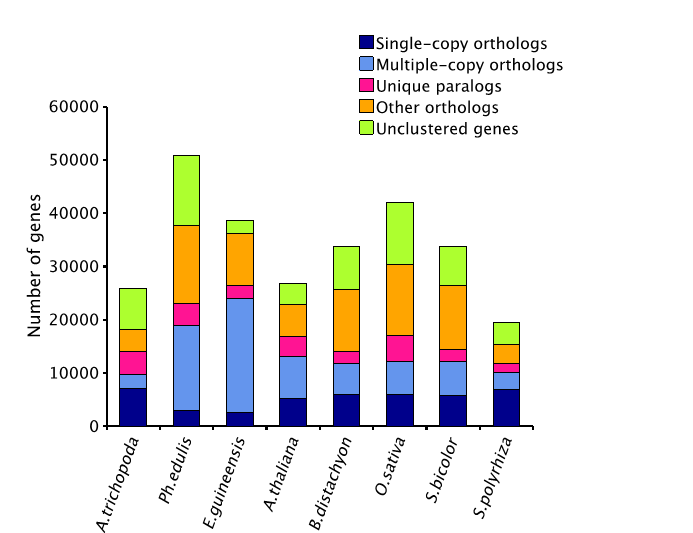


**Additional Fig. S7. Orthologous genes comparison among the ten genomes**


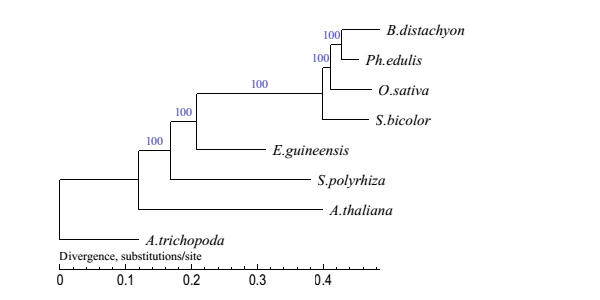


**Additional Fig. S8. A phylogenetic tree constructed with single-copy orthologous genes on 4-fold degenerate sites by maximum likelihood method.**

The branch length represents the neutral divergence rate.


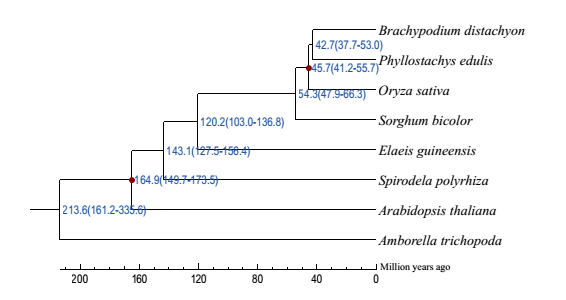


**Additional Fig. S9. Estimation of divergence time and substitution rate.**

The blue numbers on the nodes are the divergence time from present (million years ago, Mya). A Bayesian relaxed molecular clock approach was used to estimate the divergence time using MCMCTREE in PAML (version 4). Calibration times were gained from a previous study (*O. sativa vs. B. distachyon*: 40-54 Mya; *O. sativa* *vs. S. bicolor*: 45-60 Mya; *A. trichopoda* *vs.* *S. bicolor*: 119.7-199.3 Mya).


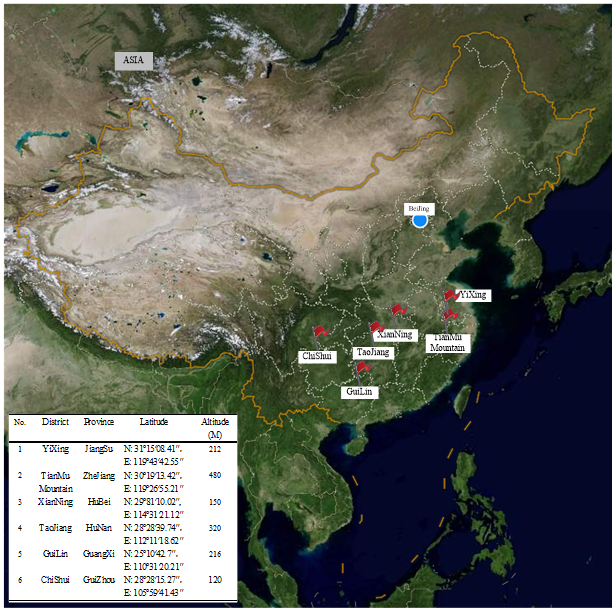


**Additional Fig. S10. The location information on the 6 sampling districts in China.**

For the 6 sampling districts, their longitude, latitude and altitude were provided in the Table


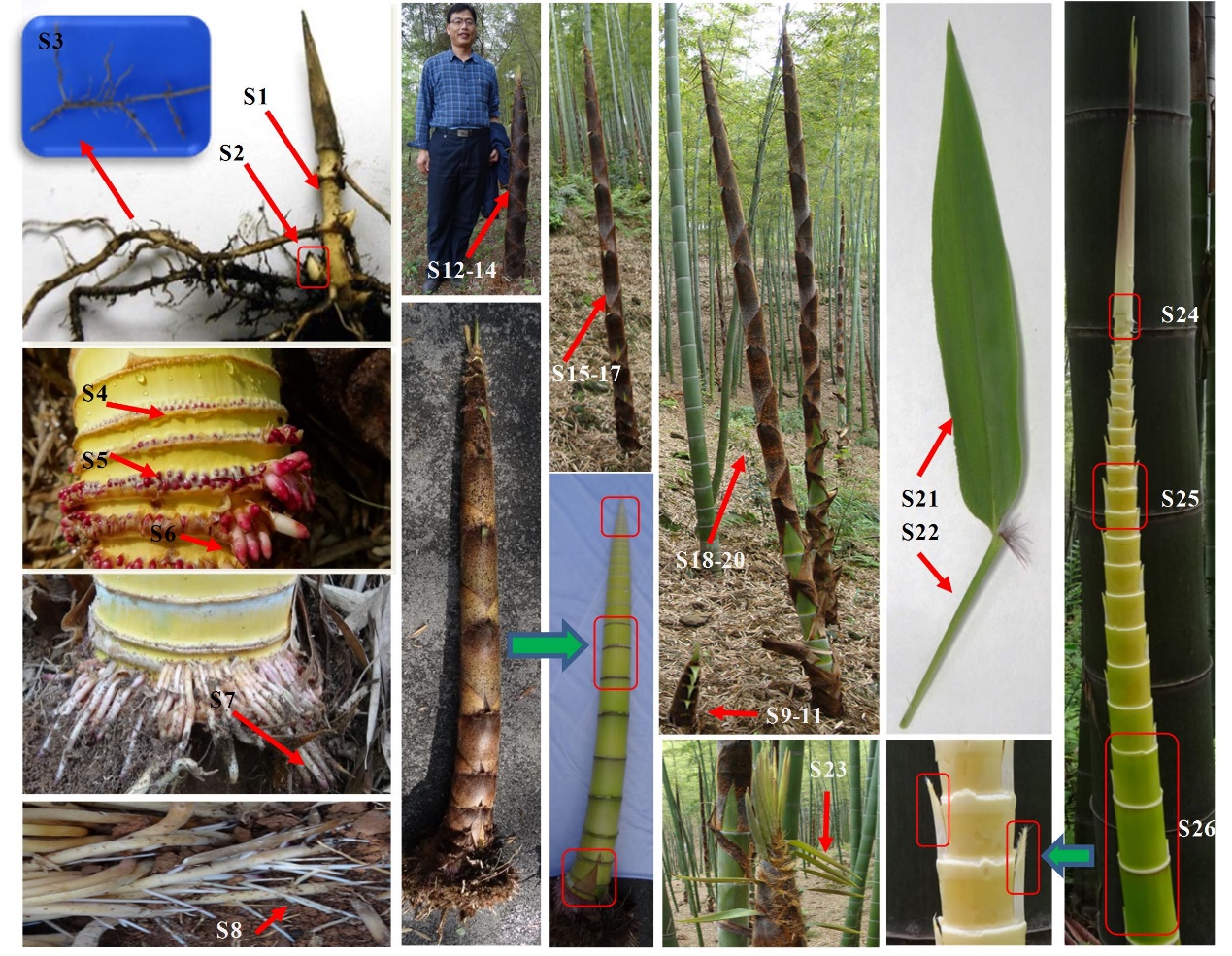


**Additional Fig. S11. Tissues collected from different developmental stages for RNA-Seq.**

**Additional Fig. S12. Coverage statistics of all expressed transcripts.**

Transcript detected is the number of transcripts with at least 5 reads. Coverage Per Base, No. Covered 5’ and No. Covered 3’are per-base coverage averaged across all top transcripts. Gap% is the total cumulative gap length divided by the total cumulative transcript lengths

**Additional Fig. S13. The distribution of reads based on the improved moso bamboo genome annotation.**

**Additional Fig. S14. The coverage of reads based on the improved moso bamboo genome annotation.**


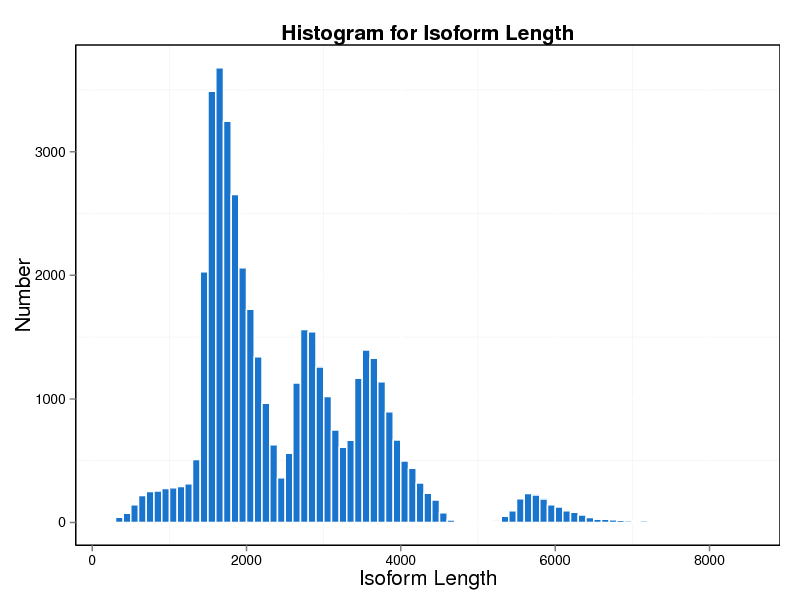


**Additional Fig. S15. The length distribution of Iso-Seq reads**

Novel AS genes: 12,572

AS genes Prediction by RNA: 25,558

AS in annotation:

12,653

Mulit-exonic genes: 49,631

**Additional Fig. S16: AS genes by Compared with the results of genome annotation and RNA prediction**


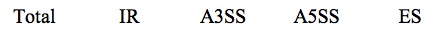


**Additional Fig. S17: The percentage of PacBio-Illumina Overlap related to PacBio result.**


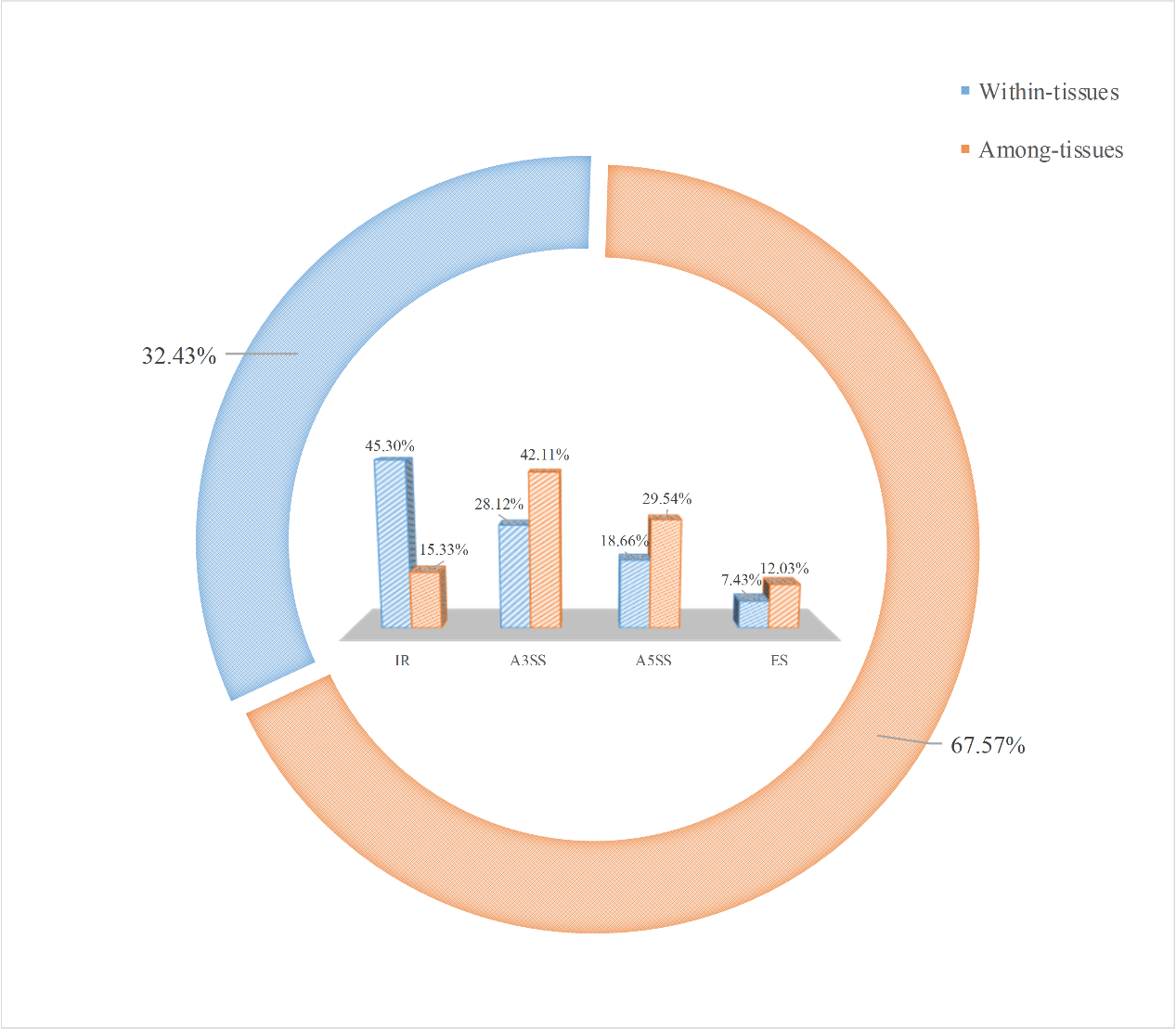


**Additional Fig. S18: The distribution of AS events within individual tissues (within-tissue) and among different tissues (among-tissue).**

Pie showed the total number of AS event within-tissue and among-tissue. Bar chart indicated the different types of AS events within-tissue and among-tissue.

**Additional Fig. S19:** **The distribution of the number and length of TE gene in bamboo.**

F1, F2, F3 represents the genes with TE insertions into the first, second, and third introns, respectively; R1, R2, R3 indicates the genes with TE insertions into the last, second last and third last introns.

**Additional Fig. S20: Splice site usage of the main four types of AS events.**


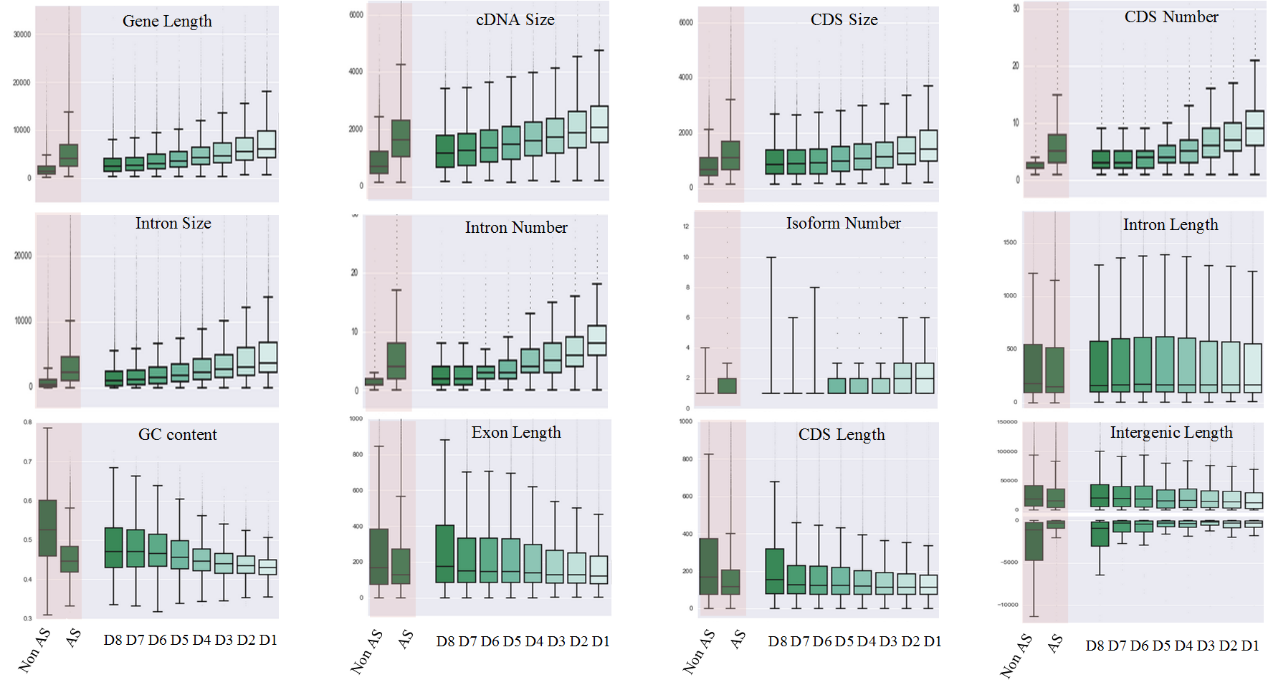
**Additional Fig. S21: The correlations between gene length, CDS length, intron length, exon number, exon cassette length, and intron cassette length in the different orthologous gene datasets.**


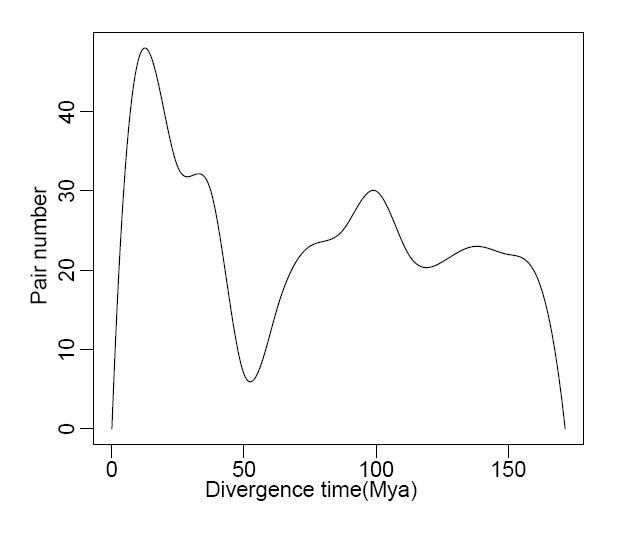


**Additional Fig. S22. The divergence time of the lignin biosynthesis genes in the moso bamboo genome.**

Additional Table

| **Additional Table S1.** Global statistics of the *Ph. edulis* genome sequencing | | | | | | | | |
| --- | --- | --- | --- | --- | --- | --- | --- | --- |
| **Library Name** | **Read Orientation** | **Insert Size (bp)** | **Insert Size STDDEV (bp)** | **Read Length (bp)** | **Amount** | **Raw data (Gb)** | **Usable data (Gb)** | **Effective depth*** |
| SRR4120067^#^ | inward | 423 | 40 | 250 | 1 | 79 | 76.1 | 35.2 |
| SRR4120069^#^ | inward | 423 | 40 | 250 | 1 | 80.6 | 77.6 | 35.9 |
| ERR105039 | inward | 461 | 38 | 125 | 1 | 5.8 | 4.3 | 2 |
| ERR105040 | inward | 461 | 38 | 125 | 1 | 5.8 | 4.4 | 2 |
| ERR105038 | inward | 461 | 38 | 125 | 1 | 6.1 | 4.2 | 1.9 |
| ERR105041 | inward | 461 | 38 | 125 | 1 | 5.8 | 4.5 | 2.1 |
| ERR105037 | inward | 461 | 38 | 125 | 1 | 5.8 | 4.1 | 1.9 |
| ERR105036 | inward | 461 | 38 | 125 | 1 | 6.2 | 4.8 | 2.2 |
| ERR105033 | inward | 461 | 38 | 120 | 1 | 5.3 | 3.9 | 1.8 |
| ERR105024 | inward | 461 | 38 | 120 | 1 | 5.4 | 4 | 1.8 |
| ERR105035 | inward | 461 | 38 | 120 | 1 | 4.9 | 3.7 | 1.7 |
| ERR105030 | inward | 461 | 38 | 120 | 1 | 5 | 3.7 | 1.7 |
| ERR105031 | inward | 461 | 38 | 120 | 1 | 5.2 | 3.9 | 1.8 |
| ERR105025 | inward | 461 | 38 | 120 | 1 | 5.2 | 3.7 | 1.7 |
| ERR105032 | inward | 461 | 38 | 120 | 1 | 5.3 | 3.9 | 1.8 |
| ERR105026 | inward | 461 | 38 | 120 | 1 | 5.3 | 2.9 | 1.4 |
| ERR105027 | inward | 461 | 38 | 120 | 1 | 5.2 | 4 | 1.8 |
| ERR105017 | inward | 462 | 37 | 119 | 1 | 5.4 | 1.3 | 0.6 |
| ERR105016 | inward | 462 | 37 | 119 | 1 | 5.3 | 1.8 | 0.8 |
| ERR105034 | inward | 462 | 38 | 120 | 1 | 5.2 | 4 | 1.8 |
| ERR105029 | inward | 462 | 38 | 120 | 1 | 5.3 | 4.2 | 1.9 |
| ERR105028 | inward | 462 | 38 | 120 | 1 | 5.3 | 4.1 | 1.9 |
| ERR105023 | inward | 462 | 38 | 120 | 1 | 5.3 | 4.2 | 1.9 |
| ERR105022 | inward | 463 | 36 | 119 | 1 | 3.9 | 0.3 | 0.2 |
| ERR105021 | inward | 463 | 36 | 119 | 1 | 3.9 | 0.4 | 0.2 |
| ERR105018 | inward | 463 | 36 | 119 | 1 | 5.3 | 1.2 | 0.5 |
| ERR105019 | inward | 463 | 36 | 119 | 1 | 4.9 | 1 | 0.5 |
| ERR105020 | inward | 463 | 37 | 119 | 1 | 3.1 | 0.8 | 0.4 |
| ERR105048 | inward | 476 | 44 | 120 | 1 | 4.5 | 3.6 | 1.7 |
| ERR105047 | inward | 476 | 44 | 120 | 1 | 4.6 | 3.7 | 1.7 |
| ERR105046 | inward | 476 | 44 | 120 | 1 | 4.7 | 3.9 | 1.8 |
| ERR105045 | inward | 476 | 44 | 120 | 1 | 4.7 | 3.9 | 1.8 |
| ERR105044 | inward | 476 | 44 | 120 | 1 | 4.6 | 3.8 | 1.8 |
| ERR105043 | inward | 476 | 44 | 120 | 1 | 4.6 | 3.8 | 1.7 |
| ERR105042 | inward | 476 | 44 | 120 | 1 | 4.5 | 3.8 | 1.8 |
| ERR105009 | inward | 491 | 54 | 120 | 1 | 10.5 | 9.7 | 4.5 |
| ERR105010 | inward | 491 | 54 | 100 | 1 | 9.5 | 8.5 | 3.9 |
| ERR105011 | inward | 491 | 54 | 100 | 1 | 10.5 | 9.6 | 4.4 |
| ERR105015 | inward | 491 | 54 | 100 | 1 | 10.6 | 9.7 | 4.5 |
| ERR105013 | inward | 491 | 54 | 100 | 1 | 10.5 | 9.5 | 4.4 |
| ERR105012 | inward | 491 | 54 | 100 | 1 | 9.9 | 8.9 | 4.1 |
| ERR105014 | inward | 491 | 54 | 100 | 1 | 9.7 | 8.8 | 4.1 |
| ERR105058 | outward | 2,857 | 797 | 76 | 1 | 2.9 | 2.7 | 1.2 |
| ERR105057 | outward | 2,858 | 788 | 76 | 1 | 2.8 | 2.7 | 1.2 |
| ERR105054 | outward | 2,859 | 777 | 76 | 1 | 2.2 | 2.1 | 1 |
| ERR105056 | outward | 2,859 | 784 | 76 | 1 | 2.8 | 2.7 | 1.2 |
| ERR105053 | outward | 2,861 | 769 | 76 | 1 | 1.9 | 1.9 | 0.9 |
| ERR105055 | outward | 2,861 | 771 | 76 | 1 | 2.7 | 2.6 | 1.2 |
| ERR105052 | outward | 2,951 | 864 | 50 | 1 | 4 | 3.6 | 1.6 |
| ERR105060 | outward | 7,297 | 2,704 | 76 | 1 | 4.9 | 4.4 | 2 |
| ERR105062 | outward | 7,331 | 2,312 | 76 | 1 | 2.6 | 2.5 | 1.2 |
| ERR105064 | outward | 7,333 | 2,288 | 76 | 1 | 2.8 | 2.6 | 1.2 |
| ERR105063 | outward | 7,335 | 2,260 | 76 | 1 | 2.6 | 2.5 | 1.2 |
| ERR105061 | outward | 7,337 | 2,276 | 76 | 1 | 2.7 | 2.5 | 1.2 |
| ERR105065 | outward | 7,640 | 2,343 | 76 | 1 | 1.9 | 1.8 | 0.8 |
| ERR105066 | outward | 7,640 | 2,346 | 76 | 1 | 1.8 | 1.7 | 0.8 |
| ERR105059 | outward | 8,030 | 3,872 | 76 | 1 | 5.7 | 5.3 | 2.5 |
| ERR105051 | outward | 14,693 | 7,094 | 76 | 1 | 1.4 | 1.2 | 0.6 |
| ERR105050 | outward | 14,694 | 7,086 | 76 | 1 | 1.4 | 1.2 | 0.6 |
| ERR105049 | outward | 14,708 | 7,075 | 76 | 1 | 1.4 | 1.2 | 0.6 |
| CL100050325^#^ | inward | \\ | \\ | 100 | 1 | 156.6 | 17.6 | 9.2 |
| Total | | | | | 61 | 603.3 | 390.9 | 204.8 |
| *Calculated with the estimated genome size of 1,908,074,089 bp | | | | | | | | |
| #Novel additional data | | | | | | | | |

| **Additional Table S2.** The list of the materials for RNA-Seq analysis | | | | |
| --- | --- | --- | --- | --- |
| Sample | Tissues | | Sample Number | Sample ID |
| Root-1 | Root | 0.1 cm root on shoot | 1 | S4 |
| Root-2 |  | 0.5 cm root on shoot | 1 | S5 |
| Root-3 |  | 2 cm root on shoot | 1 | S6 |
| Root-4 |  | 10 cm root on shoot | 1 | S7 |
| Root-5 |  | new root with lateral roots | 1 | S8 |
| Root-6 |  | root on rhizome | 1 | S3 |
| Shoot-A1/2/3 | Shoot | top/middle/lower portion of 0.2 m shoot | 3 | S9-S11 |
| Shoot-B1/2/3 |  | top/middle/lower portion of 1.5 m shoot | 3 | S12-S14 |
| Shoot-C1/2/3 |  | top/middle/lower portion of 3 m shoot | 3 | S15-S17 |
| Shoot-D1/2/3 |  | top/middle/lower portion of 6.7 m shoot | 3 | S18-S20 |
| Leaf-1 | Leaf | blade | 1 | S21 |
| Leaf-2 |  | leaf sheath | 1 | S22 |
| Sheath | Sheath sheet | Sheath sheet | 1 | S23 |
| Bud-1/2/3 | Bud | bud on top/middle/lower portion of 3 m shoot | 3 | S24-S26 |
| Bud-4 |  | bud on rhizome | 1 | S2 |
| Rhizome | Rhizome | rhizome | 1 | S1 |

| **Additional Table S3.** The statistics of RNA-Seq data in each sample | | | | | | | | |
| --- | --- | --- | --- | --- | --- | --- | --- | --- |
| **Sample** | **Total reads** | **Total Basepairs** | **Total Mapped reads** | **<=2bp mismatch** | **Unique match** | **Multi-position match** | **One Best Score match** | **Total unmatched reads** |
| S1 | 93,551,416 | 13,545,322,033 | 88,286,574 | 18,851,777 | 77,300,694 | 10,985,880 | 84,753,411 | 5,264,842 |
|  | 100.00% | 100.00% | 98.19% | 21.07% | 77.83% | 20.36% | 93.87% | 1.81% |
| S2 | 91,213,308 | 13,219,646,705 | 85,476,146 | 18,162,691 | 74,576,096 | 10,900,050 | 81,782,895 | 5,737,162 |
|  | 100.00% | 100.00% | 93.71% | 19.91% | 81.76% | 11.95% | 89.66% | 6.29% |
| S3 | 82,887,670 | 12,046,258,515 | 78,638,599 | 15,764,999 | 68,615,453 | 10,023,146 | 75,200,978 | 4,249,071 |
|  | 100.00% | 100.00% | 94.87% | 19.02% | 82.78% | 12.09% | 90.73% | 5.13% |
| S4 | 74,356,634 | 10,804,263,007 | 69,205,095 | 14,082,654 | 60,530,395 | 8,674,700 | 66,184,814 | 5,151,539 |
|  | 100.00% | 100.00% | 93.07% | 18.94% | 81.41% | 11.67% | 89.01% | 6.93% |
| S5 | 96,182,892 | 13,969,708,266 | 90,919,997 | 18,505,706 | 78,691,146 | 12,228,851 | 86,511,717 | 5,262,895 |
|  | 100.00% | 100.00% | 94.53% | 19.24% | 81.81% | 12.71% | 89.95% | 5.47% |
| S6 | 86,511,920 | 12,546,334,075 | 78,957,317 | 18,634,412 | 63,763,087 | 15,194,230 | 71,897,836 | 7,554,603 |
|  | 100.00% | 100.00% | 91.27% | 21.54% | 73.70% | 17.56% | 83.11% | 8.73% |
| S7 | 85,873,552 | 12,489,189,713 | 80,551,626 | 16,069,274 | 70,632,084 | 9,919,542 | 77,301,300 | 5,321,926 |
|  | 100.00% | 100.00% | 93.80% | 18.71% | 82.25% | 11.55% | 90.02% | 6.20% |
| S8 | 84,590,188 | 12,286,105,981 | 78,647,896 | 16,130,513 | 68,771,475 | 9,876,421 | 75,577,759 | 5,942,292 |
|  | 100.00% | 100.00% | 92.98% | 19.07% | 81.30% | 11.68% | 89.35% | 7.02% |
| S9 | 93,600,098 | 13,604,667,709 | 88,631,220 | 18,374,661 | 77,755,936 | 10,875,284 | 85,237,008 | 4,968,878 |
|  | 100.00% | 100.00% | 94.69% | 19.63% | 83.07% | 11.62% | 91.07% | 5.31% |
| S10 | 81,778,808 | 11,552,554,914 | 73,816,246 | 20,144,353 | 62,080,626 | 11,735,620 | 70,837,521 | 7,962,562 |
|  | 100.00% | 100.00% | 90.26% | 24.63% | 75.91% | 14.35% | 86.62% | 9.74% |
| S11 | 95,202,044 | 13,822,693,238 | 89,692,754 | 18,731,461 | 78,215,224 | 11,477,530 | 85,840,881 | 5,509,290 |
|  | 100.00% | 100.00% | 94.21% | 19.68% | 82.16% | 12.06% | 90.17% | 5.79% |
| S12 | 71,233,172 | 9,978,075,631 | 65,739,999 | 20,113,431 | 53,794,910 | 11,945,089 | 62,430,330 | 5,493,173 |
|  | 100.00% | 100.00% | 92.29% | 28.24% | 75.52% | 16.77% | 87.64% | 7.71% |
| S13 | 110,925,762 | 16,075,836,122 | 102,988,795 | 26,461,523 | 88,868,809 | 14,119,986 | 98,367,685 | 7,936,967 |
|  | 100.00% | 100.00% | 92.84% | 23.86% | 80.12% | 12.73% | 88.68% | 7.16% |
| S14 | 88,797,488 | 12,871,477,832 | 81,388,249 | 20,785,579 | 71,180,890 | 10,207,359 | 78,397,719 | 7,409,239 |
|  | 100.00% | 100.00% | 91.66% | 23.41% | 80.16% | 11.50% | 88.29% | 8.34% |
| S15 | 79,779,890 | 11,624,055,656 | 75,575,315 | 16,935,614 | 64,842,467 | 10,732,848 | 71,221,719 | 4,204,575 |
|  | 100.00% | 100.00% | 94.73% | 21.23% | 81.28% | 13.45% | 89.27% | 5.27% |
| S16 | 90,271,404 | 13,152,170,315 | 85,556,156 | 18,805,513 | 75,133,312 | 10,422,844 | 82,179,400 | 4,715,248 |
|  | 100.00% | 100.00% | 94.78% | 20.83% | 83.23% | 11.55% | 91.04% | 5.22% |
| S17 | 103,876,562 | 15,103,807,969 | 96,801,721 | 19,463,516 | 85,670,626 | 11,131,095 | 93,529,183 | 7,074,841 |
|  | 100.00% | 100.00% | 93.19% | 18.74% | 82.47% | 10.72% | 90.04% | 6.81% |
| S18 | 91,429,716 | 13,364,453,250 | 86,470,822 | 18,395,744 | 74,715,059 | 11,755,763 | 81,603,002 | 4,958,894 |
|  | 100.00% | 100.00% | 94.58% | 20.12% | 81.72% | 12.86% | 89.25% | 5.42% |
| S19 | 76,483,990 | 10,513,747,232 | 71,236,372 | 20,026,029 | 59,929,279 | 11,307,093 | 68,642,449 | 5,247,618 |
|  | 100.00% | 100.00% | 93.14% | 26.18% | 78.36% | 14.78% | 89.75% | 6.86% |
| S20 | 110,880,724 | 16,159,883,062 | 102,314,699 | 22,662,049 | 90,201,207 | 12,113,492 | 98,343,684 | 8,566,025 |
|  | 100.00% | 100.00% | 92.27% | 20.44% | 81.35% | 10.92% | 88.69% | 7.73% |
| S21 | 80,571,508 | 11,708,328,536 | 73,609,884 | 19,904,432 | 62,757,229 | 10,852,655 | 70,089,945 | 6,961,624 |
|  | 100.00% | 100.00% | 91.36% | 24.70% | 77.89% | 13.47% | 86.99% | 8.64% |
| S22 | 86,703,348 | 12,626,090,489 | 75,109,485 | 20,369,319 | 65,250,048 | 9,859,437 | 72,015,462 | 11,593,863 |
|  | 100.00% | 100.00% | 86.63% | 23.49% | 75.26% | 11.37% | 83.06% | 13.37% |
| S23 | 103,447,476 | 15,058,272,268 | 96,784,737 | 22,335,589 | 84,120,928 | 12,663,809 | 92,531,866 | 6,662,739 |
|  | 100.00% | 100.00% | 93.56% | 21.59% | 81.32% | 12.24% | 89.45% | 6.44% |
| S24 | 101,970,856 | 14,837,974,481 | 95,556,676 | 21,436,132 | 82,784,343 | 12,772,333 | 90,612,621 | 6,414,180 |
|  | 100.00% | 100.00% | 93.71% | 21.02% | 81.18% | 12.53% | 88.86% | 6.29% |
| S25 | 86,925,446 | 12,684,175,815 | 81,726,842 | 17,802,599 | 70,520,377 | 11,206,465 | 77,156,175 | 5,198,604 |
|  | 100.00% | 100.00% | 94.02% | 20.48% | 81.13% | 12.89% | 88.76% | 5.98% |
| S26 | 92,964,892 | 13,507,771,363 | 87,965,347 | 20,047,665 | 76,157,379 | 11,807,968 | 83,819,131 | 4,999,545 |
|  | 100.00% | 100.00% | 94.62% | 21.56% | 81.92% | 12.70% | 90.16% | 5.38% |

| **Additional Table S4. The overall statistics of RNA-Seq data** | |
| --- | --- |
| Map To Genome | Data Size |
| Total Raw Reads/Basepairs | 2,524,237,542 / 378.64 G |
| Total Clean Reads/Basepairs | 2,342,010,789 / 339.15 G |
| Mean of Clean Reads/Basepairs Per Sample* | 900,773,338 / 13.044 G |
| Median of Clean Reads/Basepairs Per Sample* | 899,534,446 / 13.012 G |
| Total Mapped Reads* | 2,181,648,569 (93.15%) |
| Unique Match Reads* | 1,886,859,079 (80.57%) |
| Less than 2 bp Mismatch Reads* | 498,997,235 (21.31%) |
| Multi-position Match Reads* | 294,789,490 (12.59%) |
| Total Unmatched Reads* | 160,362,195 (6.85%) |
| *Statistic based on clean data |  |

| **Additional Table S5.** The mapping statistics of transcript-associated reads based on RNA-Seq data | | | | | | | | | | | | | | | | | | |
| --- | --- | --- | --- | --- | --- | --- | --- | --- | --- | --- | --- | --- | --- | --- | --- | --- | --- | --- |
| **Sample** | **Mapping Rate** | | | **Intragenic Rate** | | | **Intergenic Rate** | | | **Exonic Rate** | | | **Intronic Rate** | | | **Expression Profiling Efficiency** | | |
|  | **Ver.2.2** | **Ver.2.1** | **Ver.1** | **Ver.2.2** | **Ver.2.1** | **Ver.1** | **Ver.2.2** | **Ver.2.1** | **Ver.1** | **Ver.2.2** | **Ver.2.1** | **Ver.1** | **Ver.2.2** | **Ver.2.1** | **Ver.1** | **Ver.2.2** | **Ver.2.1** | **Ver.1** |
| S1 | 94.37% | 94.37% | 94.92% | 90.42% | 90.42% | 74.74% | 8.20% | 9.58% | 25.26% | 81.79% | 78.50% | 62.71% | 10.01% | 11.92% | 12.03% | 77.19% | 74.08% | 59.53% |
| S2 | 93.71% | 93.71% | 94.35% | 90.06% | 90.06% | 71.34% | 8.63% | 9.94% | 28.66% | 80.07% | 76.93% | 58.94% | 11.29% | 13.13% | 12.41% | 75.04% | 72.09% | 55.61% |
| S3 | 94.87% | 94.87% | 95.02% | 92.64% | 92.64% | 75.86% | 5.99% | 7.36% | 24.14% | 86.07% | 82.54% | 64.88% | 7.95% | 10.09% | 10.98% | 81.65% | 78.31% | 61.65% |
| S4 | 93.07% | 93.07% | 93.81% | 89.59% | 89.59% | 73.35% | 8.79% | 10.41% | 26.65% | 80.24% | 76.69% | 60.50% | 10.97% | 12.91% | 12.85% | 74.68% | 71.38% | 56.75% |
| S5 | 94.53% | 94.53% | 94.59% | 91.94% | 91.94% | 74.92% | 6.59% | 8.06% | 25.08% | 85.36% | 81.75% | 64.00% | 8.05% | 10.19% | 10.92% | 80.69% | 77.28% | 60.54% |
| S6 | 91.27% | 91.27% | 80.81% | 78.56% | 78.56% | 74.23% | 18.55% | 21.44% | 25.77% | 74.77% | 70.23% | 63.94% | 6.68% | 8.34% | 10.29% | 68.24% | 64.10% | 51.67% |
| S7 | 93.80% | 93.80% | 93.53% | 91.47% | 91.47% | 76.73% | 7.19% | 8.53% | 23.27% | 83.38% | 79.97% | 65.19% | 9.43% | 11.50% | 11.55% | 78.21% | 75.01% | 60.97% |
| S8 | 92.98% | 92.98% | 92.48% | 91.12% | 91.12% | 75.53% | 7.48% | 8.88% | 24.47% | 84.02% | 80.58% | 64.45% | 8.50% | 10.54% | 11.07% | 78.12% | 74.92% | 59.60% |
| S9 | 94.69% | 94.69% | 95.06% | 90.37% | 90.37% | 73.88% | 7.87% | 9.63% | 26.12% | 81.25% | 77.33% | 60.65% | 10.87% | 13.05% | 13.23% | 76.94% | 73.22% | 57.65% |
| S10 | 90.26% | 90.26% | 90.77% | 90.86% | 90.86% | 74.39% | 7.50% | 9.14% | 25.61% | 82.34% | 78.66% | 62.01% | 10.16% | 12.20% | 12.38% | 74.33% | 71.00% | 56.29% |
| S11 | 94.21% | 94.21% | 94.56% | 89.89% | 89.89% | 73.82% | 8.47% | 10.11% | 26.18% | 80.61% | 76.86% | 61.48% | 10.92% | 13.03% | 12.34% | 75.94% | 72.41% | 58.13% |
| S12 | 92.29% | 92.29% | 95.22% | 91.64% | 91.64% | 72.10% | 6.66% | 8.36% | 27.90% | 84.31% | 79.87% | 60.86% | 9.03% | 11.77% | 11.24% | 77.81% | 73.71% | 57.95% |
| S13 | 92.84% | 92.84% | 94.63% | 92.34% | 92.34% | 77.23% | 6.08% | 7.66% | 22.77% | 86.53% | 82.70% | 66.41% | 7.39% | 9.63% | 10.82% | 80.34% | 76.78% | 62.85% |
| S14 | 91.66% | 91.66% | 93.99% | 91.85% | 91.85% | 77.99% | 6.20% | 8.15% | 22.01% | 85.67% | 81.50% | 66.38% | 8.13% | 10.35% | 11.61% | 78.52% | 74.70% | 62.39% |
| S15 | 94.73% | 94.73% | 95.45% | 90.72% | 90.72% | 71.58% | 7.75% | 9.28% | 28.42% | 83.24% | 78.82% | 60.37% | 9.00% | 11.90% | 11.21% | 78.86% | 74.67% | 57.62% |
| S16 | 94.78% | 94.78% | 94.83% | 91.58% | 91.58% | 77.12% | 6.98% | 8.42% | 22.88% | 84.32% | 80.80% | 65.94% | 8.70% | 10.79% | 11.18% | 79.91% | 76.58% | 62.52% |
| S17 | 93.19% | 93.19% | 93.15% | 89.81% | 89.81% | 76.33% | 8.50% | 10.19% | 23.67% | 82.09% | 78.12% | 63.86% | 9.41% | 11.69% | 12.47% | 76.50% | 72.80% | 59.48% |
| S18 | 94.58% | 94.58% | 95.23% | 90.72% | 90.72% | 73.22% | 7.74% | 9.28% | 26.78% | 83.48% | 79.09% | 62.44% | 8.78% | 11.63% | 10.78% | 78.95% | 74.80% | 59.46% |
| S19 | 93.14% | 93.14% | 94.33% | 89.66% | 89.66% | 76.01% | 8.85% | 10.34% | 23.99% | 80.88% | 77.38% | 64.74% | 10.27% | 12.28% | 11.28% | 75.33% | 72.07% | 61.07% |
| S20 | 92.27% | 92.27% | 92.49% | 86.10% | 86.10% | 73.36% | 12.44% | 13.90% | 26.64% | 74.38% | 70.99% | 60.65% | 13.18% | 15.10% | 12.71% | 68.63% | 65.51% | 56.09% |
| S21 | 91.36% | 91.36% | 92.87% | 91.05% | 91.05% | 77.39% | 7.60% | 8.95% | 22.61% | 81.69% | 78.74% | 66.09% | 10.71% | 12.32% | 11.30% | 74.63% | 71.94% | 61.38% |
| S22 | 86.63% | 86.63% | 89.43% | 87.40% | 87.40% | 71.53% | 10.58% | 12.60% | 28.47% | 78.13% | 73.73% | 58.55% | 11.28% | 13.67% | 12.97% | 67.69% | 63.87% | 52.36% |
| S23 | 93.56% | 93.56% | 93.96% | 88.81% | 88.81% | 75.09% | 9.83% | 11.19% | 24.91% | 80.52% | 77.34% | 62.90% | 9.65% | 11.47% | 12.19% | 75.33% | 72.36% | 59.11% |
| S24 | 93.71% | 93.71% | 93.65% | 89.98% | 89.98% | 73.64% | 8.48% | 10.02% | 26.36% | 81.77% | 77.96% | 62.19% | 9.75% | 12.02% | 11.45% | 76.63% | 73.06% | 58.24% |
| S25 | 94.02% | 94.02% | 93.84% | 88.88% | 88.88% | 71.70% | 9.29% | 11.12% | 28.30% | 80.78% | 76.10% | 60.16% | 9.93% | 12.78% | 11.54% | 75.95% | 71.55% | 56.45% |
| S26 | 94.62% | 94.62% | 94.22% | 90.88% | 90.88% | 74.00% | 7.65% | 9.12% | 26.00% | 82.85% | 79.19% | 62.55% | 9.50% | 11.69% | 11.45% | 78.39% | 74.93% | 58.94% |
| **Total** | **93.12%** | **93.12%** | **93.35%** | **89.94%** | **89.94%** | **74.50%** | **8.46%** | **10.06%** | **25.50%** | **81.94%** | **78.17%** | **62.80%** | **9.60%** | **11.77%** | **11.70%** | **76.33%** | **72.81%** | **58.63%** |
| Mapping Rate is per total reads. | | | | | | | | | | | | | | | | | | |
| Intragenic Rate refers to the fraction of reads the map within genes (within introns or exons). | | | | | | | | | | | | | | | | | | |
| Exonic Rate is the fraction mapping within exons. | | | | | | | | | | | | | | | | | | |
| Intronic Rate is the fraction mapping within introns | | | | | | | | | | | | | | | | | | |
| Intergenic Rate is the fraction mapping in the genomic space between genes. | | | | | | | | | | | | | | | | | | |
| Expression Profile Efficiency is the ratio of exon reads to total reads. | | | | | | | | | | | | | | | | | | |
| Ver. 2 and Ver. 1 is the two versions of moso bamboo genome, respectively. | | | | | | | | | | | | | | | | | | |

| **Supplemental Table S6.** The length distribution of transcripts | | | |
| --- | --- | --- | --- |
| transcript length | AS genes detected in transcripts | | Non-AS genes detected in transcripts |
|  | Redundancy | Non-redundancy |  |
| 4 Kb+ | 80,865 | 6,286 | 93 |
| 3-4 Kb | 131,541 | 28,166 | 393 |
| 2-3 Kb | 262,783 | 51,829 | 1,898 |
| 1-2 Kb | 306,840 | 56,803 | 6,708 |
| 0.5-1 Kb | 57,673 | 13,147 | 7,937 |
| <=500 bp | 11,034 | 2,985 | 6,516 |

| **Additional Table S7.** Summary of PacBio libraries | | | | | | |
| --- | --- | --- | --- | --- | --- | --- |
| Library | Cell Number | Reads of Insert | Read Bases of Insert(bp) | Mean Read Length of Insert(bp) | Mean Read Quality of Insert | Mean Number of Passes |
| 1-2 k | 3 | 85,701 | 154,017,521 | 1,797 | 0.94 | 12 |
| 2-3 k | 2 | 59,593 | 148,382,818 | 2,489 | 0.92 | 9 |
| >3 k | 4 | 69,078 | 259,840,799 | 3,761 | 0.89 | 4 |

| **Additional Table S8.** Summary of the *Ph. edulis* genome assembly | | | |
| --- | --- | --- | --- |
|  |  | **Version 2** | **Version 1** |
| Contig | Number | 76,900 | 865,947 |
|  | Size | 1,795,528,836 | 1,839,017,779 |
|  | N50 | 54,955 | 11,621 |
|  | N90 | 11,757 | 784 |
| Scaffold | Number | 19,285 | 277,278 |
|  | Size | 1,908,074,089 | 2,051,719,643 |
|  | N50 | 894,858 | 328,698 |
|  | N90 | 115,487 | 1,733 |
| Total number (>=1 kb) | Number | 19,266 (99.90%) | 128,306 (46.27%) |
|  | Size | 1,908,055,690 | 1,950,287,080 |
| Total number (>=2 kb) | Number | 14,826 (76.88%) | 33,204 (11.98%) |
|  | Size | 1,901,188,518 | 1,826,358,859 |
| Total number (>=3 kb) | Number | 11,528 (59.78%) | 17,727 (6.40%) |
|  | Size | 1,893,060,130 | 1,789,416,159 |
| The longest sequence | | 4,993,432 | 4,869,017 |
| The shortest sequence | | 926 | 500 |
| N ratio (%) | | 5.90% | 10.37% |
| GC ratio (%) | | 44.20% | 43.90% |

| **Additional Table S9**. Distribution of the scaffold length for the *Ph. edulis* genome assembly | | | | | | | |  |
| --- | --- | --- | --- | --- | --- | --- | --- | --- |
| **Scaffold length** | **Version 2** | | | | **Version 1** | | | |
|  | **Number** | **Subtotal length (bp)** | **Average length (bp)** | **Percentage*** | **Number** | **Subtotal length (bp)** | **Average length (bp)** | **Percentage*** |
| >1,000 kb | 417 | 676,969,237 | 1,623,427 | 35.48% | 173 | 246,167,126 | 1,422,931 | 12.00% |
| >500 kb | 1,171 | 1,203,564,621 | 1,027,809 | 63.08% | 875 | 723,606,853 | 826,979 | 35.27% |
| >100 kb | 3,300 | 1,737,160,118 | 526,412 | 91.04% | 4,423 | 1,556,233,931 | 351,850 | 75.85% |
| >50 kb | 4,359 | 1,814,057,396 | 416,164 | 95.07% | 5,982 | 1,668,395,794 | 278,903 | 81.32% |
| >30 kb | 5,073 | 1,841,929,784 | 363,085 | 96.53% | 7,009 | 1,708,661,320 | 243,781 | 83.28% |
| >20 kb | 5,588 | 1,854,668,015 | 331,902 | 97.20% | 7,800 | 1,728,328,721 | 221,581 | 84.24% |
| >10 kb | 6,679 | 1,869,846,230 | 279,959 | 98.00% | 9,396 | 1,750,712,067 | 186,325 | 85.33% |
| *Calculated with the estimated genome size: version 2 of 1,908,074,089 bp; version 1 of 2,051,719,643 bp | | | | | | | |  |

**Additional Table S10. Chromosomes length of Hi-C assembly**

| **Scaffold ID** | **Length(bp)** |
| --- | --- |
| moso_draft_hic_scaffold_1 | 29,393,643 |
| moso_draft_hic_scaffold_2 | 45,530,765 |
| moso_draft_hic_scaffold_3 | 103,881,109 |
| moso_draft_hic_scaffold_4 | 63,624,372 |
| moso_draft_hic_scaffold_5 | 55,715,837 |
| moso_draft_hic_scaffold_6 | 77,743,242 |
| moso_draft_hic_scaffold_7 | 64,081,027 |
| moso_draft_hic_scaffold_8 | 79,898,979 |
| moso_draft_hic_scaffold_9 | 65,709,403 |
| moso_draft_hic_scaffold_10 | 57,248,354 |
| moso_draft_hic_scaffold_11 | 44,603,463 |
| moso_draft_hic_scaffold_12 | 50,795,940 |
| moso_draft_hic_scaffold_13 | 137,299,170 |
| moso_draft_hic_scaffold_14 | 108,238,415 |
| moso_draft_hic_scaffold_15 | 103,306,719 |
| moso_draft_hic_scaffold_16 | 121,622,346 |
| moso_draft_hic_scaffold_17 | 100,753,730 |
| moso_draft_hic_scaffold_18 | 4,956,4831 |
| moso_draft_hic_scaffold_19 | 30,060,168 |
| moso_draft_hic_scaffold_20 | 53,830,895 |
| moso_draft_hic_scaffold_21 | 114,306,658 |
| moso_draft_hic_scaffold_22 | 60,531,284 |
| moso_draft_hic_scaffold_23 | 87,068,002 |
| moso_draft_hic_scaffold_24 | 72,468,335 |
| Total | 1,750,047,892 |

**Additional Table S11. Collinearity between the moso bamboo and rice chromosomes**

| The moso bamboo chromosomes | Best blast hits in the rice chromosomes | Coverage | 2^nd^-best blast hits in the rice chromosomes | Coverage |
| --- | --- | --- | --- | --- |
| moso_draft_hic_scaffold_1 | Chr11 | 21.03% | Chr8 | 10.25% |
| moso_draft_hic_scaffold_10 | Chr7 | 69.31% | Chr4 | 3.62% |
| moso_draft_hic_scaffold_11 | Chr12 | 55.55% | Chr4 | 7.20% |
| moso_draft_hic_scaffold_12 | Chr12 | 49.85% | Chr11 | 7.31% |
| moso_draft_hic_scaffold_13 | Chr10 | 35.49% | Chr8 | 15.86% |
| moso_draft_hic_scaffold_14 | Chr1 | 53.15% | Chr4 | 5.45% |
| moso_draft_hic_scaffold_15 | Chr3 | 49.64% | Chr4 | 7.46% |
| moso_draft_hic_scaffold_16 | Chr1 | 78.50% | Chr12 | 2.62% |
| moso_draft_hic_scaffold_17 | Chr2 | 74.93% | Chr4 | 3.14% |
| moso_draft_hic_scaffold_18 | Chr9 | 24.41% | Chr5 | 18.49% |
| moso_draft_hic_scaffold_19 | Chr9 | 28.60% | Chr7 | 14.00% |
| moso_draft_hic_scaffold_2 | Chr11 | 41.86% | Chr12 | 11.37% |
| moso_draft_hic_scaffold_20 | Chr2 | 38.12% | Chr4 | 8.09% |
| moso_draft_hic_scaffold_21 | Chr3 | 75.22% | Chr4 | 3.36% |
| moso_draft_hic_scaffold_22 | Chr8 | 69.15% | Chr12 | 4.20% |
| moso_draft_hic_scaffold_23 | Chr4 | 49.20% | Chr11 | 5.58% |
| moso_draft_hic_scaffold_24 | Chr4 | 73.83% | Chr2 | 2.95% |
| moso_draft_hic_scaffold_3 | Chr9 | 46.06% | Chr2 | 16.68% |
| moso_draft_hic_scaffold_4 | Chr7 | 47.49% | Chr4 | 6.61% |
| moso_draft_hic_scaffold_5 | Chr10 | 31.38% | Chr4 | 8.86% |
| moso_draft_hic_scaffold_6 | Chr6 | 40.07% | Chr1 | 8.99% |
| moso_draft_hic_scaffold_7 | Chr11 | 28.95% | Chr5 | 28.13% |
| moso_draft_hic_scaffold_8 | Chr6 | 71.57% | Chr4 | 3.66% |
| moso_draft_hic_scaffold_9 | Chr5 | 74.24% | Chr12 | 2.99% |

| **Additional Table S12.** Assessment of sequence coverage of the *Ph. edulis* genome assembly using BAC sequences. | | | | | | | | | | | | |
| --- | --- | --- | --- | --- | --- | --- | --- | --- | --- | --- | --- | --- |
| **EMBL Accession** | **BAC** | **BAC length (bp)** | **Matched Scaffold** | **Scaffold length (bp)** | **Alignment region in BAC (bp)** | **Alignment region in Scaffold (bp)** | **Match length (bp)** | **Gap 1* (bp)** | **Gap 2* (bp)** | **Gap 3* (bp)** | **Ns* (bp)** |  |
| gi\|440577269\|emb\|FO203436.1\| | FO203436 | 167734 | PH02Scaffold03096 | 117,023 | 38,640-159,804 | 0-117,023 | 113,375 | 57 | 7732 | 3591 | 0 |  |
|  |  |  | PH02Scaffold02971 | 129,196 | 13.508-30.626 | 79.294-93.901 | 11.727 | 381 | 5,010 | 2,499 | 0 |  |
|  |  |  | PH02Scaffold02857 | 141,032 | 0-13,233 | 0-13,172 | 13,010 | 14 | 209 | 148 | 0 |  |
| gi\|440577297\|emb\|FO203437.1\| | FO203437 | 166,330 | PH02Scaffold00003 | 4,414,907 | 0-166,330 | 3,374,385-3,839,455 | 162,324 | 237 | 3,769 | 2,509 | 0 |  |
| gi\|440577325\|emb\|FO203439.1\| | FO203439 | 126,856 | PH02Scaffold00426 | 987,002 | 0-126,856 | 404,876-532,097 | 126,784 | 71 | 1 | 366 | 0 |  |
| gi\|440577350\|emb\|FO203441.1\| | FO203441 | 133,793 | PH02Scaffold00065 | 2,178,417 | 0-133,793 | 279,198-437,608 | 122,290 | 83 | 11,420 | 36,037 | 0 |  |
| gi\|440577375\|emb\|FO203443.1\| | FO203443 | 113,960 | PH02Scaffold00734 | 709,026 | 0-113.960 | 277,785-390,972 | 112,943 | 39 | 978 | 205 | 0 |  |
| gi\|440577394\|emb\|FO203444.1\| | FO203444 | 136,023 | PH02Scaffold00005 | 4,077,984 | 0-136,023 | 519,497-654,352 | 128,505 | 52 | 7,465 | 6,297 | 1 |  |
| gi\|440577422\|emb\|FO203447.1\| | FO203447 | 96,838 | PH02Scaffold00318 | 1,165,034 | 0-65,838 | 938,097-1,035,203 | 95,611 | 56 | 1,171 | 1,439 | 0 |  |
| gi\|440577441\|emb\|FO203448.1\| | FO203448 | 126,165 | PH02Scaffold00145 | 1,626,340 | 0-126,165 | 465,321-591,544 | 125,939 | 33 | 186 | 244 | 0 |  |
| * Gap1, gap counts in the aligned region between scaffold and BAC; Gap2, gap counts existed in BAC; Gap3, gap counts existed in Scaffold; Ns, the N counts in the aligned region between scaffold and BAC | | | | | | | | | | | | |

| **Additional Table S13.** Assessment of the sequence coverage of the *Ph. edulis* genome assembly by homologous search with 10,608 cDNA sequences with a cutoff of >=95% identify | | | | | | | | | | | |
| --- | --- | --- | --- | --- | --- | --- | --- | --- | --- | --- | --- |
|  |  |  |  |  |  |  |  |  |  |  |  |
| **cDNA length** | **Number** | **Coverage >0%** | | **Coverage >80%** | | **Coverage >90%** | | **Coverage >95%** | | **Coverage >99%** | |
|  |  | **Number** | **%** | **Number** | **%** | **Number** | **%** | **Number** | **%** | **Number** | **%** |
| >2000 bp | 278 | 273 | 98.20% | 259 | 93.17% | 257 | 92.45% | 256 | 92.09% | 243 | 87.41% |
| >1000 bp | 5,854 | 5,754 | 98.29% | 5,601 | 95.68% | 5,565 | 95.06% | 5,520 | 94.29% | 5,209 | 88.98% |
| >500 bp | 10,145 | 9,996 | 98.53% | 9,774 | 96.34% | 9,694 | 95.55% | 9,600 | 94.63% | 8,981 | 88.53% |
| >200 bp | 10,601 | 10,442 | 98.50% | 10,193 | 96.15% | 10,108 | 95.35% | 9,999 | 94.32% | 9,325 | 87.96% |
| All | 10,608 | 10,429 | 98.31% | 10,200 | 96.15% | 10,115 | 95.35% | 10,006 | 94.33% | 9,329 | 87.94% |

| **Additional Table S14.** Comparison of the assembled scaffolds and the 15 known genes mRNA/coding sequences of GenBank | | | | | | | | | | |
| --- | --- | --- | --- | --- | --- | --- | --- | --- | --- | --- |
| **#** | **GenBank** | | |  | **Matched Scaffold** | | | **Mapping length** | **Coverage** | **Description** |
|  | **Accession NO.** | **Size** | **Alignment region** | **Strand** | **Name** | **Size** | **Alignment region** |  |  |  |
| 1 | gi\|145845825\|gb\|EF549577.1\| | 1,019 | 0-1,019 | - | PH02Scaffold00195 | 1,441,835 | 401,216-402,235 | 1,017 | 99.80% | cinnamyl alcohol dehydrogenase |
| 2 | gi\|145845829\|gb\|EF549579.1\| | 787 | 0-787 | - | PH02Scaffold02649 | 163,289 | 17,442-18,229 | 785 | 99.75% | caffeoyl-CoA O-methyltransferase |
| 3 | gi\|162568699\|gb\|EU295482.1\| | 1,131 | 0-1,113 | - | PH02Scaffold00237 | 1,319,672 | 569,402-571,080 | 1,111 | 98.23% | DRE-binding protein DREB2 (DREB2) |
| 4 | gi\|169743367\|gb\|EU366146.1\| | 1,071 | 0-1,043 | + | PH02Scaffold00233 | 1,342,464 | 1,258,665-1,259,902 | 1,036 | 96.73% | chloroplast chlorophyll a/b binding protein |
| 5 | gi\|190694830\|gb\|EU780143.1\| | 544 | 0-542 | + | PH02Scaffold00678 | 750,950 | 696,056-696,712 | 533 | 97.98% | chloroplast chlorophyll a/b binding protein |
| 6 | gi\|195546525\|gb\|EU860441.1\| | 1,205 | 0-1,204 | + | PH02Scaffold01465 | 408,521 | 84,287-85,491 | 1,201 | 99.67% | DRE-binding protein DREB1 (DREB1) |
| 7 | gi\|222154090\|gb\|FJ594467.1\| | 2,139 | 0-2,139 | + | PH02Scaffold00633 | 783,562 | 33,423-35,683 | 2,102 | 98.27% | phenylalanine ammonia-lyase (PAL1) |
| 8 | gi\|222154092\|gb\|FJ594468.1\| | 2,260 | 0-2,260 | + | PH02Scaffold00633 | 783,562 | 33,423-35,683 | 2,225 | 98.45% | phenylalanine ammonia-lyase (PAL1) |
| 9 | gi\|237506882\|gb\|FJ495287.1\| | 3,244 | 0-3,244 | - | PH02Scaffold00808 | 669,342 | 270,515-276,393 | 3,236 | 99.75% | cellulose synthase (cesA1) |
| 10 | gi\|251766020\|gb\|FJ475350.1\| | 3,214 | 0-3,214 | + | PH02Scaffold00648 | 772,566 | 233,470-240,182 | 3,213 | 99.97% | cellulose synthase (CesA2) |
| 11 | gi\|251766022\|gb\|FJ475351.1\| | 3,257 | 0-3,257 | + | PH02Scaffold02464 | 190,723 | 79,192-84,971 | 3,200 | 98.25% | cellulose synthase (CesA4) |
| 12 | gi\|255764546\|gb\|FJ600727.1\| | 810 | 0-810 | - | PH02Scaffold00435 | 979,137 | 417,594-419,955 | 808 | 99.75% | PsbS protein |
| 13 | gi\|294818264\|gb\|GU944762.1\| | 579 | 0-579 | - | PH02Scaffold02851 | 141,381 | 23,487-25,098 | 577 | 99.65% | putative pathogenesis protein (WRKY10) |
| 14 | gi\|301071262\|gb\|GU434145.1\| | 1,225 | 4-1,223 | + | PH02Scaffold01348 | 441,751 | 285,544-288,584 | 1,215 | 99.18% | Actin |
| 15 | gi\|312232178\|gb\|HM747940.1\| | 1,766 | 0-1,747 | - | PH02Scaffold01337 | 445,349 | 404,272-406,254 | 1,707 | 96.66% | MYB protein |

| **Additional Table S15.** Repetitive sequences in the improved *Ph. edulis* genome | | | |
| --- | --- | --- | --- |
|  | Number of elements* | Length occupied (bp) | Percentage of sequence (%) |
| SINEs: | 1981 | 554016 | 0.03 |
| LINEs: | 33986 | 33247066 | 1.74 |
| LINE1 | 27472 | 30940160 | 1.62 |
| L3/CR1 | 4911 | 1676856 | 0.09 |
| LTR elements: | 668807 | 837435518 | 43.89 |
| DNA elements: | 390717 | 198674003 | 10.41 |
|  |  |  |  |
| Unclassified: | 377803 | 136691217 | 7.16 |
|  |  |  |  |
| Total interspersed repeat |  | 1206601820 | 63.24 |
|  |  |  |  |
| Small RNA: | 1700 | 311813 | 0.02 |
| Satellites: | 3616 | 1476869 | 0.08 |
| Simple repeats: | 222621 | 15849750 | 0.83 |
| Low complexity: | 33059 | 1829540 | 0.1 |
| *Most repeats fragmented by insertions or deletions have been counted as one element | | | |
|  |  |  |  |

| **Additional Table S16**. Transcripts of 26 RNA-Seq | | | | | |
| --- | --- | --- | --- | --- | --- |
| Sample | Transcripts | Total size of assembled transcripts (bp) | Average length (bp) | Meidan contig length (bp) | GC% |
| S1 | 132694 | 187914789 | 1416.15 | 1106 | 47.38 |
| S2 | 139267 | 198527147 | 1425.51 | 1095 | 46.85 |
| S3 | 114419 | 158565735 | 1385.83 | 1091 | 48.07 |
| S4 | 116558 | 163513773 | 1402.85 | 1091 | 47.18 |
| S5 | 118005 | 163288618 | 1383.74 | 1096 | 47.92 |
| S6 | 97820 | 133344650 | 1363.16 | 1073 | 48.2 |
| S7 | 115407 | 163679854 | 1418.28 | 1112 | 47.66 |
| S8 | 117068 | 161159247 | 1376.63 | 1079 | 48.24 |
| S9 | 121627 | 183797749 | 1511.16 | 1179 | 46.63 |
| S10 | 125742 | 176795121 | 1406.11 | 1081 | 46.7 |
| S11 | 123292 | 176495073 | 1431.52 | 1113 | 47.04 |
| S12 | 120142 | 169362037 | 1409.68 | 1101 | 47.29 |
| S13 | 114062 | 158899257 | 1393.1 | 1088 | 47.68 |
| S14 | 106467 | 149313012 | 1402.43 | 1105 | 47.66 |
| S15 | 116470 | 163171781 | 1400.98 | 1096 | 47.47 |
| S16 | 107424 | 150300849 | 1399.14 | 1101 | 47.89 |
| S17 | 120354 | 173461837 | 1441.26 | 1142 | 47.06 |
| S18 | 106012 | 141803170 | 1337.61 | 1052 | 48.21 |
| S19 | 104964 | 147038633 | 1400.85 | 1107 | 47.91 |
| S20 | 138115 | 199085153 | 1441.44 | 1122 | 46.85 |
| S21 | 108358 | 159654764 | 1473.4 | 1180 | 47.6 |
| S22 | 132666 | 185638296 | 1399.29 | 1100 | 47.28 |
| S23 | 132960 | 197644868 | 1486.5 | 1181 | 47.49 |
| S24 | 129155 | 184680989 | 1429.92 | 1130 | 47.84 |
| S25 | 119748 | 168283015 | 1405.31 | 1132 | 47.95 |
| S26 | 124423 | 179742605 | 1444.61 | 1144 | 47.93 |
| Integration | 633279 | 627226990 | 990.44 | 527 | 45.94 |
| S1: rhizome; S2: bud on rhizome; S3: root on rhizome; S4/S5/S6/S7: 0.1/0.5/2/10 cm root on shoot; S8: new root with lateral roots; S9/10/11: top/middle/lower portion of 0.2 m shoot; S12/13/14: top/middle/lower portion of 1.5 m shoot; S15/16/17: top/middle/lower portion of 3 m shoot; S18/19/20: top/middle/lower portion of 6.7 m shoot; S21: blade; S22: leaf sheath; S23: Sheath sheet; S24/25/26: bud on top/middle/lower portion of 3 m shoot | | | | | |
|  |  |  |  |  |  |
|  |  |  |  |  |  |
|  |  |  |  |  |  |
|  |  |  |  |  |  |
|  |  |  |  |  |  |

| **Additional Table S17.** General statistics of predicted protein-coding genes in the improved *Ph. edulis* genome | | | | | | | |
| --- | --- | --- | --- | --- | --- | --- | --- |
| Gene Set | | Number | Average Gene Length (bp) | Average CDS Length (bp) | Average Exon per Gene | Average Exon Length (bp) | Average Intron Length (bp) |
| *De novo* | Augustus | 114,568 | 2,025.02 | 662.95 | 2.76 | 240.53 | 775.59 |
|  | Fgenesh | 141,852 | 2,274.67 | 812.54 | 3.8 | 213.8 | 522.11 |
| Homolog | *B. distachyon* | 39,581 (completed) | 4,146.82 | 1,226.41 | 5.02 | 244.29 | 726.39 |
|  |  | 8,251 (partial) |  |  |  |  |  |
|  | *O. sativa* | 39,821 (completed) | 3,163.43 | 1,001.73 | 4.01 | 250.11 | 719.34 |
|  |  | 8,887 (partial) |  |  |  |  |  |
|  | *S. bicolor* | 39,425 (completed) | 3,991.52 | 1,189.63 | 4.83 | 246.43 | 732.06 |
|  |  | 7,673 (partial) |  |  |  |  |  |
|  | *S. italica* | 39,668 (completed) | 4,020.86 | 1,201.71 | 4.88 | 246.45 | 727.33 |
|  |  | 8,902 (partial) |  |  |  |  |  |
|  | *T. aestivum* | 40,766 (completed) | 3,236.48 | 972.75 | 4.18 | 232.82 | 712.31 |
|  |  | 11,903 (partial) |  |  |  |  |  |
|  | *Z. mays* | 39,803 (completed) | 3,684.14 | 1,110.75 | 4.62 | 240.25 | 710.22 |
|  |  | 8,369 (partial) |  |  |  |  |  |
| Expression data | RNA-Seq | 38,608 | 4,235.55 | 1,275.64 | 5.68 | 275.11 | 686.25 |
|  | Full-length cDNA | 7,734 | 4,213.43 | 1,123.54 | 5.45 | 256.21 | 700.12 |
|  | PacBio Data | 15,654 | 4,012.67 | 1,001.32 | 5.09 | 243.81 | 687.49 |
| Final Gene Set | | 51,661 | 4,551.35 | 1,268.62 | 5.36 | 284.22 | 668.14 |
|  |  |  | (with UTR) |  |  |  |  |

| **Additional Table S18.** Comparison of gene numbers and features in the seven genomes | | | | | | |  |  |
| --- | --- | --- | --- | --- | --- | --- | --- | --- |
| Items | *Ph. edulis* v2.2^#^ | *Ph. edulis* v2.1*^#^* | *Z. mays* | *O. sativa* | *B. distachyon* | *S. italica* | *S. bicolor* | *A. thaliana* |
| Total Gene Length | 235,956,416 | 234,499,035 | 190,558,497 | 121,157,000 | 115,735,410 | 109,989,438 | 126,998,638 | 60,480,140 |
| Total Gene Number | 51,074 | 51,523 | 63,480 | 42,189 | 34,310 | 34,584 | 34,211 | 27,416 |
| Gene Number ( >1 Kb) | 44,685 | 44,486 | 39,807 | 31,804 | 28,712 | 28,564 | 29,339 | 21,850 |
| Gene Number ( >2 Kb) | 34,514 | 34,290 | 26,147 | 22,937 | 21,004 | 20,662 | 21,976 | 12,853 |
| Gene_length | 3053/4567.40 | 3037/4551.35 | 1526/3001.87 | 2246/2871.77 | 2617/3373.23 | 2540/3180.36 | 2821/3712.22 | 1909/2206.02 |
| Isoform_number | 1/1.48 | 1/1.47 | 1/1.4 | 1/1.24 | 1/1.54 | 1/1.24 | 1/1.38 | 1/1.29 |
| AS_gene_number: | 12,986 | 12,436 | 13,561 | 6,382 | 8,010 | 5,067 | 6,886 | 5,804 |
| Intergenic_length>0 | 16781/27396.06 | 16791/27435.71 | 13414/33364.73 | 3284/6141.81 | 2172/5061.43 | 3100/8869.17 | 3835/18206.39 | 966/2332.42 |
| Intergenic_length<0 | -433/-1786.9 | -436/-1722.91 | -1225.5/-2896.23 | -902/-1505.62 | -1310.5/-2445.75 | -753/-1867.64 | -1129/-2865.79 | -93/-409.96 |
| Number of Intergenic_length<0 | 6370/1.22% | 991/1.92% | 6652/10.48% | 796/1.89% | 2438/7.11% | 1239/3.58% | 2052/6.00% | 1736/6.33% |
| cDNA_length | 1329/1544.90 | 1309/1524.16 | 962/1268.62 | 1279/1459.77 | 1511/1745.2 | 1479/1696.45 | 1600/1834.77 | 1335/1486.36 |
| Exon_number | 4/5.35 | 4/5.36 | 2/3.65 | 3/4.43 | 3/4.78 | 3/4.89 | 3/4.88 | 4/5.31 |
| CDS_length | 957/1172.32 | 957/1170.32 | 570/829.97 | 888/1112.41 | 942/1131.81 | 1005/1189.52 | 981/1161.87 | 1047/1218.4 |
| CDS_num | 4/5.19 | 4/5.21 | 2/3.55 | 3/4.22 | 3/4.44 | 3/4.64 | 3/4.51 | 3/5.13 |
| **Intron_length** | 1494/2928.90 | 1500/2941.08 | 212/1664.72 | 753/1388.41 | 853/1581.48 | 815/1457.59 | 961/1838.66 | 496/706.74 |
| **Intron_num** | 3/4.35 | 3/4.36 | 1/2.65 | 2/3.43 | 2/3.78 | 2/3.89 | 2/3.88 | 3/4.31 |
| Single_exon_length | 134/248.34 | 142/284.22 | 171/347.81 | 168/329.74 | 179/365.17 | 172/347 | 177/375.81 | 155/280.05 |
| Single_CDS_length | 123/209.40 | 125/224.58 | 138/233.49 | 143/263.36 | 139/255.06 | 138/256.62 | 138/257.51 | 134/237.59 |
| Single_intron_length | 146/490.55 | 166/668.14 | 124/548.97 | 143/378.71 | 133/398.57 | 129/358.61 | 137/452.34 | 97/155.96 |
| GC% (Gene) | 48.56 | 48.25 | 50.41 | 49.98 | 48.93 | 45.8 | 50.38 | 39.55 |
| GC% (cDNA) | 53.98 | 54.01 | 53.23 | 54.79 | 52.83 | 54.52 | 52.71 | 42.2 |
| GC% (CDS) | 56.63 | 55.75 | 55.53 | 57.13 | 56.25 | 57.3 | 56.59 | 44.35 |
| BUSCO | 95.2/3.6 | 95.1/3.7 | 92.2/2.5 | 95.6/1.9 | 98.6/0.6 | 98.4/0.7 | 98.3/0.7 | 99.3/0.4 |
| ^#^assessment based on the version 2.1 and 2.2 of the annotation of *Ph. Edulis,* other annotation from JGI database | | | | | | | | |
| *median value/average value. | | | | | | | | |
| ^$^represents the percent of completed mapping/missing mapping by BUSCO | | | | | | | | |

| **Additional Table S19.** The summary of refined annotation based on the full-length cDNA and PacBio data | | | |
| --- | --- | --- | --- |
|  | **FL-cDNA** | **PacBio data** | **Both** |
| **Sequence Summary** |  |  |  |
| Total transcript reads | 10,608 | 44,001 | 54,609 |
| Number transcripts with alignment | 10,457 | 43,941 | 54,398 |
| Number genes with alignment | 7,734 | 15,654 | 21,100 |
| **Annotation Update** |  |  |  |
| New genes | 170 | 98 | 146 |
| Gene merging | 5 | 7 | 8 |
| Gene splitting | 0 | 16 | 18 |
| Gene extension | 33 | 68 | 97 |
| Internal gene structure refined | 689 | 2,678 | 3,129 |
| UTR addition | 1,721 | 4,123 | 5,439 |
| Total | 2,618 | 6,990 | 8,837 (17.30%)* |

*All refined gene models are 8,837, accounted for ~17.30% of total gene models (51,074)

| **Additional Table S20. The evaluation summary for the different versions of the moso bamboo genome and annotation by BUSCO** | | | | | |
| --- | --- | --- | --- | --- | --- |
|  | C | S | D | F | M |
| Genome_v1 | 94.5 | 71.1 | 23.4 | 1.9 | 3.6 |
| Annotation_v1 | 75.0 | 61.4 | 13.6 | 10.3 | 14.7 |
| Genome_v2 | 96.6 | 66.3 | 30.3 | 0.7 | 2.7 |
| Annotation_v2.1 | 95.1 | 38.1 | 57.0 | 1.2 | 3.7 |
| Annotation_v2.2 | 95.2 | 37.6 | 57.6 | 1.2 | 3.6 |
| C represents complete BUSCOs | | | | | |
| S represents complete and single-copy BUSCOs | | | | | |
| D represents complete and duplicated BUSCOs | | | | | |
| F represents fragmented BUSCOs | | | | | |
| M represents missing BUSCOs | | | | | |
| Totally 1440 BUSCO groups were searched in BUSCO version 3 | | | | | |

| **Additional Table S21.** The statistics of gene annotation in *Ph. edulis* | | | |
| --- | --- | --- | --- |
|  |  | Number | Percent (%) |
| Total |  | 51,074 | -- |
| Annotated | Nr | 49,609 | 97.13% |
|  | InterPro | 48,382 | 94.73% |
|  | GO | 35,105 | 68.73% |
|  | COG | 21,403 | 41.91% |
|  | KEGG | 38,012 | 74.43% |
|  | Swiss-Prot | 41,124 | 80.52% |
|  | TrEMBL | 49,656 | 97.22% |
| Unannotated |  | 1,418 | 2.78% |

| **Additional Table S22**. Summary of predicted non-coding RNAs in the improved *Ph. edulis* genome | | | | | |
| --- | --- | --- | --- | --- | --- |
| **Type** | **Subtype** | **Copy number** | **Average length(bp)** | **Total length(bp)** | **Ratio** |
|  |  |  |  |  | **-1.00E-04** |
| miRNA |  | 349 | 119.65* | 41,759 | 21.89 |
| tRNA |  | 881 | 74.91 | 65,997 | 34.59 |
| rRNA | rRNA | 408 | 133.77 | 54,580 | 28.6 |
|  | 18S | 303 | 140.52 | 42,578 | 22.31 |
|  | 28S | 16 | 121.25 | 1,940 | 1.02 |
|  | 5.8S | 3 | 153 | 459 | 0.24 |
|  | 5S | 86 | 111.66 | 9,603 | 5.03 |
| snRNA | snRNA | 910 | 112.37 | 102,255 | 53.59 |
|  | CD-box | 639 | 97.86 | 62,530 | 32.77 |
|  | HACA-box | 72 | 120.11 | 8,648 | 4.53 |
|  | splicing | 199 | 156.17 | 31,077 | 16.29 |

*Calculated with the estimated genome size: version 2 of 1,908,074,089 bp

| **Additional Table S23**. The TF distribution of moso bamboo and other grasses | | | | | | | |
| --- | --- | --- | --- | --- | --- | --- | --- |
| TF type* | *Ph. edulis* | *A. thaliana* | *B. distachyon* | *O. sativa* | *S. bicolor* | *S. italica* | *Z. mays* |
| Alfin-like | 20 | 7 | 9 | 9 | 11 | 10 | 16 |
| AP2/ERF-AP2 | 43 | 13 | 23 | 14 | 21 | 25 | 4 |
| AP2/ERF-ERF | 244 | 124 | 135 | 139 | 148 | 164 | 219 |
| AP2/ERF-RAV | 9 | 4 | 4 | 4 | 3 | 6 | 3 |
| B3 | 69 | 66 | 51 | 54 | 60 | 59 | 52 |
| B3-ARF | 50 | 22 | 26 | 27 | 25 | 24 | 11 |
| BBR-BPC | 7 | 7 | 3 | 4 | 5 | 3 | 5 |
| BES1 | 12 | 8 | 8 | 6 | 9 | 10 | 19 |
| bHLH | 265 | 137 | 130 | 135 | 153 | 161 | 108 |
| BSD | 0 | 2 | 0 | 0 | 1 | 1 | 0 |
| bZIP | 160 | 71 | 83 | 90 | 93 | 85 | 81 |
| C2C2-CO-like | 18 | 16 | 10 | 11 | 9 | 10 | 4 |
| C2C2-Dof | 69 | 36 | 29 | 30 | 30 | 35 | 57 |
| C2C2-GATA | 49 | 30 | 29 | 25 | 30 | 31 | 41 |
| C2C2-LSD | 9 | 3 | 5 | 6 | 5 | 5 | 12 |
| C2C2-YABBY | 17 | 6 | 8 | 8 | 8 | 9 | 30 |
| C2H2 | 190 | 106 | 115 | 121 | 129 | 136 | 171 |
| C3H | 106 | 57 | 58 | 57 | 46 | 49 | 118 |
| CAMTA | 14 | 5 | 7 | 6 | 8 | 7 | 7 |
| CPP | 17 | 8 | 9 | 11 | 8 | 10 | 16 |
| CSD | 23 | 4 | 4 | 3 | 2 | 1 | 5 |
| DBB | 10 | 3 | 4 | 8 | 7 | 6 | 3 |
| DBP | 8 | 2 | 5 | 4 | 5 | 5 | 3 |
| E2F-DP | 24 | 8 | 11 | 8 | 11 | 9 | 25 |
| EIL | 16 | 6 | 6 | 9 | 8 | 6 | 10 |
| FAR1 | 57 | 17 | 123 | 74 | 49 | 45 | 26 |
| GARP-ARR-B | 11 | 8 | 7 | 6 | 9 | 7 | 30 |
| GARP-G2-like | 97 | 41 | 50 | 46 | 42 | 47 | 0 |
| GeBP | 25 | 20 | 15 | 17 | 17 | 20 | 28 |
| GRAS | 145 | 34 | 63 | 60 | 81 | 57 | 135 |
| GRF | 23 | 9 | 12 | 12 | 8 | 10 | 5 |
| HB-BELL | 31 | 13 | 14 | 13 | 13 | 13 | 0 |
| HB-HD-ZIP | 78 | 42 | 37 | 40 | 42 | 45 | 38 |
| HB-KNOX | 24 | 8 | 10 | 9 | 10 | 11 | 0 |
| HB-other | 23 | 11 | 12 | 15 | 7 | 9 | 43 |
| HB-PHD | 6 | 2 | 3 | 1 | 3 | 2 | 22 |
| HB-WOX | 25 | 16 | 13 | 14 | 12 | 13 | 1 |
| HRT | 1 | 2 | 1 | 1 | 1 | 2 | 0 |
| HSF | 54 | 24 | 24 | 25 | 24 | 27 | 34 |
| LFY | 2 | 1 | 1 | 1 | 1 | 1 | 3 |
| LIM | 12 | 6 | 6 | 6 | 5 | 6 | 3 |
| LOB | 63 | 43 | 28 | 36 | 34 | 33 | 65 |
| MADS-MIKC | 46 | 39 | 33 | 35 | 35 | 34 | 12 |
| MADS-M-type | 66 | 69 | 46 | 37 | 43 | 38 | 79 |
| MYB | 241 | 142 | 122 | 117 | 131 | 134 | 66 |
| MYB-related | 108 | 58 | 57 | 65 | 77 | 74 | 268 |
| NAC | 266 | 112 | 128 | 136 | 127 | 134 | 230 |
| NF-X1 | 3 | 2 | 2 | 2 | 3 | 2 | 5 |
| NF-YA | 20 | 10 | 7 | 11 | 9 | 10 | 31 |
| NF-YB | 25 | 13 | 17 | 13 | 14 | 16 | 11 |
| NF-YC | 25 | 14 | 15 | 16 | 15 | 16 | 15 |
| NOZZLE | 0 | 1 | 0 | 0 | 0 | 0 | 0 |
| OFP | 87 | 17 | 32 | 31 | 37 | 34 | 47 |
| PLATZ | 27 | 12 | 14 | 15 | 17 | 20 | 18 |
| RWP-RK | 20 | 14 | 16 | 12 | 13 | 17 | 18 |
| S1Fa-like | 1 | 3 | 1 | 2 | 1 | 1 | 2 |
| SAP | 0 | 1 | 0 | 0 | 0 | 0 | 0 |
| SBP | 38 | 17 | 17 | 19 | 19 | 19 | 66 |
| SRS | 10 | 10 | 6 | 5 | 6 | 5 | 21 |
| STAT | 2 | 2 | 1 | 1 | 1 | 1 | 0 |
| TCP | 46 | 24 | 21 | 21 | 20 | 22 | 56 |
| Tify | 36 | 15 | 15 | 17 | 19 | 20 | 38 |
| Trihelix | 52 | 26 | 26 | 27 | 28 | 30 | 55 |
| TUB | 26 | 11 | 12 | 15 | 13 | 16 | 29 |
| ULT | 3 | 2 | 1 | 2 | 1 | 1 | 3 |
| VOZ | 4 | 2 | 2 | 2 | 2 | 2 | 7 |
| Whirly | 6 | 3 | 2 | 2 | 2 | 2 | 4 |
| WRKY | 192 | 73 | 89 | 101 | 97 | 109 | 217 |
| zf-HD | 29 | 17 | 21 | 14 | 14 | 16 | 29 |
| *TF type was descried in http://bioinfo.bti.cornell.edu/cgi-bin/itak/index.cgi | | | | | | | |

| **Additional Table S24**. Statistical of gene families | | | | | | |
| --- | --- | --- | --- | --- | --- | --- |
| Species | Genes number | Genes in families | Unclustered genes | Family number | Unique families | Average genes per family |
| *A. trichopoda* | 25953 | 18106 | 7847 | 12077 | 1018 | 1.5 |
| *Ph. edulis* | 50748 | 37698 | 13050 | 17675 | 1460 | 2.13 |
| *E. guineensis* | 38697 | 36175 | 2522 | 13125 | 770 | 2.76 |
| *A. thaliana* | 26829 | 22869 | 3960 | 12534 | 1060 | 1.82 |
| *B. distachyon* | 33774 | 25686 | 8088 | 17288 | 699 | 1.49 |
| *O. sativa* | 42099 | 30342 | 11757 | 18382 | 1235 | 1.65 |
| *S. bicolor* | 33799 | 26387 | 7412 | 17625 | 748 | 1.5 |
| *S. polyrhiza* | 19457 | 15331 | 4126 | 11400 | 407 | 1.34 |

**Additional Table S25.** The results of GO enrichment for common AS genes

(See the excel file)

| **Additional Table S26.** The family number in lignin biosynthesis pathway | | | | | | | |
| --- | --- | --- | --- | --- | --- | --- | --- |
| Family | *A. thaliana* | *B. distachyon* | *O. sativa* | *Ph. edulis* | *P. trichocarpa* | *S. bicolor* | Total |
| 4CL: 4-coumarate CoA ligase | 12 | 13 | 12 | 15 | 13 | 16 | 81 |
| C3H: Coumarate 3-hdroxylase | 3 | 1 | 1 | 3 | 3 | 2 | 13 |
| C4H: Cinnamate 4-hydroxylase | 1 | 2 | 3 | 6 | 2 | 2 | 16 |
| CAD: Cinnamyl alcohol dehydrogenase | 9 | 7 | 10 | 14 | 17 | 11 | 68 |
| CCoAOMT: Caffeoyl-CoA 3-O-methyltransferase | 4 | 7 | 6 | 9 | 5 | 5 | 36 |
| CCR: Cinnamoyl-CoA reductase | 3 | 9 | 12 | 17 | 10 | 11 | 62 |
| COMT: Caffeic acid 3-O-methyltransferase | 11 | 4 | 6 | 4 | 11 | 5 | 41 |
| F5H: Ferulate 5-hydroxylase | 1 | 4 | 5 | 16 | 17 | 11 | 54 |
| HCT：hydroxycinnamoyl-CoA | 3 | 12 | 6 | 16 | 7 | 13 | 57 |
| LAC: Laccase | 16 | 22 | 20 | 41 | 47 | 21 | 167 |
| PAL: Phenylalanine ammonia lyase | 4 | 9 | 8 | 12 | 5 | 10 | 48 |
| CHS: Chalcone synthase | 4 | 7 | 17 | 12 | 13 | 27 | 80 |
| POD: Peroxidase | 45 | 44 | 37 | 77 | 56 | 42 | 301 |
| Total | 116 | 141 | 143 | 242 | 206 | 176 | -- |

|  | **Phylogenetic Tree under Best Model** | |  | **Branch site model (BSM)** | | |  |  |
| --- | --- | --- | --- | --- | --- | --- | --- | --- |
| **CHS** | **012013+I+G+F** | **I:0.08** |  | **Model** | **np** | **Ln L** | **Model compared** | **LRT P-value** |
|  |  | **G:1.41** |  | Model A | 164 | -23616.502085 | Model A vs.Model A null | 1.07E-05 |
|  |  | **F:0.21,0.28,0.27,0.24** |  | Model A null | 163 | -23626.196211 |  |  |
|  |  |  |  |  |  |  |  |  |
| **HCT** | **010213+I+G+F** | **I:0.12** |  | **Model** | **np** | **Ln L** | **Model compared** | **LRT P-value** |
|  |  | **G:1.03** |  | Model A | 116 | -22255.849273 | Model A vs.Model A null | 1.48E-02 |
|  |  | **F:0.18,0.35,0.31,0.17** |  | Model A null | 115 | -22258.818225 |  |  |
|  |  |  |  |  |  |  |  |  |
| **CAD** | **010213+I+G** | **I:0.17** |  | **Model** | **np** | **Ln L** | **Model compared** | **LRT P-value** |
|  |  | **G:1.37** |  | Model A | 138 | -26405.911674 | Model A vs.Model A null | 3.40E-03 |
|  |  |  |  | Model A null | 137 | -26410.200264 |  |  |
|  |  |  |  |  |  |  |  |  |

**Additional Table S27:** The result of positive selection in three gene families

**Additional Table S28.** One hundred and forty genes of lignin biosynthesis pathway experimentally validated collected from public studies

(See the excel file)
