# Supplementary material for: Chromosome-level reference genome and alternative splicing atlas of moso bamboo (Phyllostachys edulis)
Source: Gigascience. 2018 Sep 8;7(10):giy115. doi: 10.1093/gigascience/giy115 (PMC6204424; doi:10.1093/gigascience/giy115)
Supplement: Response_to_Reviewer_Comments_Report_(Original_Submission).pdf [file giy115_response_to_reviewer_comments_report_(original_submission).pdf]

Author's Response To Reviewer Comments

Close

Scott Edmunds
Executive Editor
GigaScience

19 Apr 2018

Dear Dr. Scott,

Re: Manuscript reference No. GIGA-D-18-00076
Please find attached a revised version of our manuscript "Chromosome-level reference genome and alternative splicing atlas of moso bamboo (Phyllostachys edulis)", which we would like to resubmit for publication as a research article in GigaScience.

Your comments and those of the reviewers were highly insightful and enabled us to greatly improve the quality of our manuscript. In the following pages are our point-by-point responses to each of the comments and suggestions of the reviewers.

Revisions in the text are shown using red highlight. In accordance with the two reviewers' suggestions, we carefully revised our manuscript, including modified incorrected descriptions, putting the methods into Protocols.io (https://www.protocols.io/researchers/hansheng-zhao), and adding the analyses and extensive description of alternative splicing and evolution. We hope that these revisions in the manuscript and our accompanying responses will be sufficient to make our manuscript suitable for publication in GigaScience.

We shall look forward to hearing from you at your earliest convenience.

Yours sincerely,

Prof. Hansheng Zhao
Address: No. 8, Fu Tong Dong Da Jie, Chaoyang District, Beijing 100102, P.R. China
Tel: +86-010-8478 9804
Fax: +86-010-8478 9802
E-mail: zhaohansheng@icbr.ac.cn

Responses to the comments of Reviewer #1
The authors provide a high-quality genome assembly and gene annotation of moso bamboo in order to improve the first version published in 2013. Transcriptomic analysis was performed using several tissues to identify alternative splicing events and polymorphism within gene transcription by providing a repertoire of alternative transcription that could

support tissue specialization. Additionally, an evolutionary insight, especially regarding the genes involved in lignin biosynthesis.
The genomic resource described in this manuscript will facilitate future studies on the evolution and functional genomic of moso bamboo and other grasses by providing a valuable information to the researchers interested in this area.
I recommend the manuscript for publication, following some minor revision, which I have listed below by manuscript page (p.) and line (L) numbers.

1. p. 3 - L20: Instead of "…have investigated in bamboo…" should be '…have been investigated in bamboo...'.

Response: Thank you very much for pointing out this error. We have revised the sentence, as follows:
"Only a limited number of genome-wide studies have been investigated in bamboo"

2. p. 4 - L27: Please add suppl. table reference for transcriptomic data.

Response: Thank you for this excellent suggestion. We have added additional table reference, as follows:
"Additionally, for the transcriptomic analysis, approximately 379 Gb and 5 Gb of raw data were produced from the Illumina and PacBio platforms, respectively (Additional Tables S2-7)"

3. p. 4 - L30: It was found a conflicting value "…We identified 266,711 uniform AS…". In the abstract section, the number of transcripts mentioned is 266,771. Please insert the correct value.

Response: Thank you very much for pointing out this error. The number, 266,711, is right number. We are sorry for the typo error and we have revised the sentence in the Abstract section, as follows:
"Moreover, we provide a comprehensive AS profile based on the identification of 266,711 uniform AS events in 25,225 AS genes by large-scale transcriptomic sequencing of 26 representative bamboo tissues using both the Illumina and PacBio sequencing platforms."

4. p. 4 L49: In the sentence "…we performed the genome assembly using different strategies to obtain a better genome assembly.", I suggest indicating the additional reference for detailed steps of the genome assembly.

Response: Thank you for this excellent suggestion. We have added related descriptions, as follows:
"Subsequently, we performed the genome assembly using different strategies to obtain a better genome assembly (see the Additional File for details)"

5. p. 5 L8: Instead of "…rice genome to find a mean coverage…" should be '…rice genome and we obtained a mean coverage…'.

Response: Thank you for this excellent suggestion. We have revised the sentence, as follows:
“Then we aligned the moso chromosomes to the rice genome and we obtained a mean coverage of ~59.77%”

6. p. 5 L13: About bamboo BAC sequences, I suggest mentioning that these sequences are derived from other bamboo specie (Ph. heterocycla) in this section or in the additional table S6.

Response: We appreciate this observation. In fact, the old Latin name, Ph. heterocycla, is a synonym of Ph. edulis and the both Latin names indicate the same bamboo (moso bamboo). Therefore, the BAC sequences mentioned in our manuscript are also derived from moso bamboo.

7. p. 5 L24: Provide correct reference - "…we predicted 51,074 high-quality protein-coding loci… (Additional Table S10)". Instead of Table S10, it should be Table S11.

Response: Thank you very much for pointing out this error. We have re-organized and re-numbered the Additional Table, as follows:
“we predicted 51,074 high-quality protein-coding loci with intact structures in moso bamboo (Additional Table S17)”

8. p. 5 L30: Provide correct reference - "… ~17% of the gene models were precisely refined (Additional Table S11)". Instead of Table S11, it should be Table S12.

Response: Thank you very much for pointing out this error. We have revised the table and re-organized and re-numbered the Additional Table, as follows:
“According to our results, ~17% of the gene models were precisely refined by the UTR addition and internal structural adjustment (Additional Table S19).”

9. p. 5 L36: Provide correct reference - Regarding the annotation using BUSCO, the reference should be Additional Table S13 instead of S12 in "(Fig 1d and Additional Table S12)".

Response: Thank you very much for pointing out this error. We have added the reference of BUSCO, and re-organized and re-numbered the Additional Table, as follows:
“According to the completeness assessment of the annotation using BUSCO [1], moso bamboo (95.2%) was more complete than Z. mays (92.2%) but close to O. sativa (95.6%) (Fig. 1d and Additional Table S20)”

10. p. 7 L27: The additional table reference for the enrichment analysis should be S25 instead of S26.

Response: Thank you very much for pointing out this error. We have re-organized and re-

numbered the Additional Table, as follows:
“As the functional implication of AS genes, the enrichment analysis result showed 885 genes, which alternatively spliced in all samples, significantly enriched in RNA metabolic processing, mRNA processing, RNA processing and RNA splicing in the processes (Additional Table S25).”

11. p. 7 L32: "…which account for one-third of the AS events (termed as among-tissue)." According to additional Fig S18, the AS events classified as among-tissue correspond to two-third of the AS events.

Response: Thank you very much for pointing out this error. We are sorry for the typo error and we have revised the sentence,
as follows:
“Since AS possess strong specificity to different tissues or developmental stages, we identified 181,105 tissue-specific AS events (67.57%), which account for two-thirds of the AS events (termed as among-tissue).”

12. p. 8 L43-45: The sentence "the distribution of the TE genes in the 8 datasets was examined. A substantially negative correlation" could be '…was examined and a substantially negative…'

Response: Thank you for this excellent suggestion. We have revised this sentence, as follows:
“Moreover, the distribution of the TE genes in the 8 datasets was examined and a substantially negative correlation was observed, indicating that the more conserved genes had more TE insertions.”

13. p. 10 L28: "…a higher percentage of IR (38.22%) and other AS types (total 28.18%) were observed in bamboo." In order to avoid misunderstanding, I suggest clarifying that 'other AS types' represent a set of AS events except the main AS types already mentioned in the manuscript.

Response: Thank you for this excellent suggestion. We have added the related descriptions in the Analysis part, as follows:
“In subsequent analyses, we defined the four main AS types represented intron retention (IR), alternative 3’ splice site donor (A3SS), alternative 5’ splice site acceptor (A5SS), and exon skipping (ES), and we also defined the other AS types represented some AS types except the above four main AS types.”

14. p. 16 L50: Please, provide release number of the pfam-A.hmm database.

Response: Thank you for this excellent suggestion. We have added the related information, as follows:
“The filtered sequences were subsequently analyzed by hmmsearch using the Pfam-A.hmm database (released 2017/03/31).”

Figures and Tables

15. Figure 2: The figure legend should explain the meaning of the acronyms IR, A3SS, A5SS, and ES.

Response: We appreciate this observation. We have added the related description in the figure legend of Figure 2, as follows:
"IR, A3SS, A5SS, and ES represents intron retention, alternative 3' splice site donor, alternative 5' splice site acceptor, and exon skipping, respectively."

16. Figure S3: In the figure legend "…The while boxes…" should be '…The white boxes…'

Response: Thank you very much for pointing out this error. We are sorry for the typo error and we have revised the figure legend of Figure S3, as follows:
"The white boxes in the BAC represent ambiguous bases (Ns) and the yellow line represent well aligned sequences between the BAC and the sequences."

17. Fig. S17: Is the x-axis data label named 'AS' correct?

Response: Thank you very much for pointing out this error. We are sorry for the typo error. The second pillar in the X-axis should be 'IR' instead of 'AS'. Therefore, we have revised the figure.

18. Table S1: Please provide the correct number of libraries in the 'Total' description.

Response: Thank you very much for pointing out this error. The total number should be 61 and we have revised the table

19. Table S3: Asterisk with description in the legend is not shown in the table.

Response: Thank you very much for pointing out this error. We have added asterisks in the Table S3

20. Table S28: I recommend excluding the words 'totally' and 'were' in the table legend.

Response: Thank you for this excellent suggestion. We have revised the table legend of Table S28, as follows:
"Additional Table S28. One hundred and forty genes of lignin biosynthesis pathway experimentally validated collected from public studies"

21. Some figures and tables citation are missing in the manuscript, such as Fig. 1a, 1b, and 3d; and additional table S23.

Response: Thank you for this excellent suggestion. We have added the figures and tables

citation in the manuscript and reorganized tables citation in the Additional Files, as follows:
“Then, the Hi-C assembly was generated with total length reached 1.91 Gb as well as contig and scaffold N50 length with 53.29 Kb and 79.90 Mb based on the Hi-C data and the improved WGS assembly (Fig. 1a and 1b).”
“Additionally, compared with the AS events among the genes expressed in samples with different specificities (maxTs) (for details, see Methods), the maxTs obviously increased from D8 to D1, representing an enhancement in the sample specificity from a highly conserved gene dataset to a poorly conserved dataset (Fig. 3d).”
“Additionally, for the transcriptomic analysis, approximately 379 Gb and 5 Gb of raw data were produced from the Illumina and PacBio platforms, respectively (Additional Tables S2-7)”

Dataset
22. The PacBio reads (IsoSeq) must also be submitted to GiGADB or SRA and their accession number provided in the manuscript.

Response: We appreciate this observation. We have provided the SRA accession number (SRR7032261-69) for Iso-Seq data in the manuscript, as follows:
“RNA-Seq raw sequence data for the 26 samples and Iso-Seq raw sequence data for a mixture sample were deposited in NCBI Short Read Archive database under the accession numbers: SRX2408703-28 and SRR7032261-69, respectively.”

Responses to the comments of Reviewer #2
Reviewer #2: Zhao et al reported a much improved genome assembly of moso bamboo, and characterized its alternative splicing (AS) atlas. While I find the assembly result very impressive, I have several questions on the methodology.
1. According to the method text in the "Additional File", the RNA reads were mapped onto the genome by "BLAT" and refined by HISAT. This is a rather unconventional way to map RNA-seq data to a reference genome. Why not just use HISAT2? BLAT was designed to align transcripts, not individual RNA-seq reads. Also, I'm not aware of the adjustment function in HISAT. Please make sure the read mapping was done correctly because this is fundamental to the AS analysis.

Response: Thank you very much for pointing out this error. We are sorry for the unclear and confusion description of the RNA-Seq analyses in our Additional File. Indeed, as your mentioned, correctly mapping is fundamental for AS analyses, we double-checked our shell scripts and found the aligning RNA-Seq reads only used HISAT2 (release 2.0.4) rather than HISAT and BLAT. We have revised the sentence in the Additional File, as follows:
“Similarly, RNA-Seq data, a kind of high-throughput expressed data, were mapped to the

genome to identify exon-intron splicing junctions and refine the alignment of RNA-Seq reads to the genome, using HISAT2 (version 2.0.4)[2].”

2. One important conclusion the authors made is that the conserved genes tend to have more AS events. It is however unclear to me how the authors measured the degrees of conservation. The authors did a gene family classification, and "obtained 8 datasets of orthologous genes representing different levels of conservation (Fig. 3a) designated dataset8 (more conserved genes) to dataset1 (bamboo-specific genes) based on a phylogenetic relationship of 8 selected species." But there was no further explanation. What does the "most conserved genes" in dataset8 entail? Based on presence or absence? And what is the difference between, say dataset8 and dataset7? How gene families were clustered was also unexplained (at least I couldn't find it). These are critical details that are missing.

Response: Thank you for this excellent suggestion. Based on the genome-wide identification of orthologous genes in the selected 8 plants (Amborella trichopoda, A. thaliana, Elaeis guineensis, B. distachyon, O. sativa, Spirodela polyrhiza, S. bicolor and Ph. edulis) and the species divergence time in a phylogeny tree (Fig. 3a), we identified eight orthologous gene datasets. For instance, dataset8 (D8) represents common orthologous genes in the selected 8 plants, which were located in an early divergence time in the phylogeny tree. D7 represents common orthologous genes in the selected 7 plants except A. trichopoda (the specie with the earliest divergence time) and D7 doesn’t contain orthologues genes in D8, and so on. Thus, D1 represents bamboo-specific orthologous genes, which were located in later divergence time. According to a previous study [3], we obtained the divergence times of genes based on the presence and absence of orthologs in the phylogeny. In our subsequent study, thus, we considered the bamboo-specific genes (D1) as a poorly conserved gene dataset and the common genes in all selected plants (D8) as a highly conserved gene dataset, and the degree of conservation decreased monotonically from D8 to D1. Lastly, we have revised the related descriptions and Fig. 3a, as follows:
“Evolutionary analysis of AS in moso bamboo
Based on the genome-wide identification of orthologous genes in the selected 8 plants (Amborella trichopoda, A. thaliana, Elaeis guineensis, B. distachyon, O. sativa, Spirodela polyrhiza, S. bicolor and Ph. edulis) and the species divergence time in a phylogeny tree (Fig. 3a), we identified eight orthologous gene datasets. For instance, dataset8 (D8) represented common orthologous genes in the selected 8 plants, which were located in an early divergence time in our constructed phylogeny. D7 represented common orthologous genes in the selected 7 plants except A. trichopoda (the specie with the earliest divergence time) and D7 doesn’t contain orthologues genes in D8. And so on. Thus, D1 represented bamboo-specific orthologous genes, which were located in later divergence time. According to a previous study[3], we obtained the divergence times of genes based on the presence and absence of orthologs in the phylogeny. In our subsequent study, thus, we considered the bamboo-specific genes (D1) as a poorly conserved gene dataset and the common genes in all selected plants (D8) as a highly conserved gene dataset, and the degree of conservation decreased monotonically from D8 to D1.”

3. A species phylogeny was reconstructed from 8 genomes, but no information is provided about how this was done. What are the "single-copy orthologous genes"? What methods and programs you used for phylogenetic reconstruction?

Response: Thank you for this excellent suggestion. We have revised the related methods in the Additional File, as follows:
"S3.1 Orthologous Gene and Phylogenetic
The identification of orthologous gene clusters was considered as a fundamental aspect of genome evolution. Single-copy gene families and multi-gene families were identified by orthMCL (version 2.0.9) [4] among Ph. edulis and other 7 plant species, including Amborella trichopoda (version 1.0) from Amborella Genome Database (amborella.huck.psu.edu), Elaeis guineensis (GCF_000442705.1) from NCBI database, Arabidopsis thaliana (TAIR10), Brachypodium distachyon (version 3.1), Oryza sativa (version 7.0), Spirodela polyrhiza (version 2) and Sorghum bicolor (version 3.1) from the ENSEMBL database. The statistic of the gene family clustering in the 8 species was showed in Additional Table S24. The comparison of gene family clustering was provided in Additional Fig. S7. Afterwards, all single-copy genes were used to construct the phylogenetic tree by PhyML (version 3.0) [5] specifying a HKY85 substitution model with a gamma distribution across sites (Additional Fig. S8)."

4. Species divergence time was estimated, but again, the authors provided no methodological detail. How was the molecular clock estimated? Did you test the validity of assuming a molecular clock (e.g. relative rate test)? Further, the time calibrations listed in the Additional File need citations; they also to me look like secondary calibrations rather than "fossil time".

Response: Thank you for this excellent suggestion. We are sorry for the unclear and confusion description in the analysis of the species divergence time. Indeed, we estimated the species divergence time using calibration time rather than fossil time and we have revised the related Method in the Additional File, as follows:
"S3.3 Estimation of Divergence Time
In order to estimate the divergence time between Ph. edulis and the other 7 sequenced plant genomes, a Bayesian relaxed molecular clock approach was used to estimate the divergence time using MCMCTREE in PAML (version 4)[6]. Calibration times were gained from a previous study [7] (O. sativa vs. B. distachyon: 40-54 Mya; O. sativa vs. S. bicolor: 45-60 Mya; A. trichopoda vs. S. bicolor: 119.7-199.3 Mya)."

5.Though I appreciate the artistic value of Fig. 3A, it is scientifically incorrect (or at least very confusing). The x-axis is apparently in unit of substitution/site, which is a branch length measurement. It does not make sense to have a terminal tip linked (by vertical dashed line) to a branch length value. There was also no "divergence times" information in this figure and the legend should be revised.

Response: Thank you for this excellent suggestion. we have re-made the Fig.3a.

6. The expansion of lignin biosynthesis genes could be due to whole genome duplication (WGD), but WGD was not discussed. Are the two decoupled?

Response: Thank you for this excellent suggestion. According to the additional analysis of the divergence time of lignin biosynthesis genes, we have added an explanation about the expansion of lignin biosynthesis genes in the aspect of WGD in the Analysis and

Discussion, respectively, as follows:
In the section of Analysis
"Additionally, we calculated the synonymous substitution rate analysis for 13 gene families evolved in the lignin biosynthesis using the yn00, which was a package in PAML to estimate synonymous and nonsynonymous substitution rates. Then, the Ks rate was translated to the divergence time by the formula T=Ks/2r (r=6.5×10-9). As shown in Additional Fig. S22, the result indicated that the divergence time of the lignin biosynthesis genes occurred at the 5~16 million year ago (Mya), which correspond to the whole genome duplication (WGD) time 7~12 Mya in the moso bamboo genome [8]."
In the section of Discussion
"Combined with the results of the divergence time of the lignin biosynthesis genes and our previous study [8], we estimated the occurrence of a putative WGD event at 7~12 Mya in the moso bamboo genome, suggesting that there might have been a tetraploidization event during bamboo history [8]. Then, the ancient tetraploid moso bamboo evolved into a current diploid moso bamboo. Additionally, WGD could provide more gene copies, which facilitated evolving the genes with new functions [9]. Therefore, the expansion of the lignin biosynthesis genes in moso bamboo could be due to the occurrence of WGD event."

Some other comments are listed below. The manuscript has no page number, and the line numbers does not match the actual lines and restart in each page, which make the review difficult. Anyway, I tried my best to point out where in the text I was referring to.
Abstract
7. Line 18 - what is "additional abundance data"? You meant sequencing data?

Response: We appreciate this observation. Indeed, the data means the sequencing data and we have revised the sentence in the Abstract, as follows:
"Here, we provide a chromosome-level de novo genome assembly of the moso bamboo (Phyllostachys edulis) using additional abundance sequencing data and hybrid-combined de novo assembly strategies."

8. Line 31 - "dramatic evolutionary characteristics" is too dramatic and unclear. Please be specific or take out this sentence.

Response: Thank you for this excellent suggestion. We have removed the sentence in the Abstract, as follows:
"Via comparison with orthologous genes in related plants, we observed that the AS genes are concentrated in more conserved genes that tend to accumulate higher expressed transcripts and share less specificity.

9. Line 39 - what does "bamboo's specificity in being a woody plant" mean? Please clarify "specificity".

Response: Thank you for this excellent suggestion. Our result indicated moso bamboo has the features of woody bamboo in the grass family based on the analysis of the lignin biosynthesis pathway. To properly express the meaning, we have revised the sentence in the Abstract, as follows:
"Furthermore, gene family expansion, abundant AS and positive selection were identified in

crucial genes involved in lignin biosynthesis, indicating that moso bamboo is a woody plant in the grass family.

Background

10. Line 20 - change "investigated" to "been carried out".

Response: Thank you very much for pointing out this error. We have revised the sentence, as follows:
“Only a limited number of genome-wide studies have been investigated in bamboo.”

11. Line 46 - change "is responsible" to "is partly responsible".

Response: Thank you for this excellent suggestion. We have revised the sentence, as follows:
“Species-specific AS is partly responsible for a wide variety of biodiversity with limited repertoires of protein coding genes”

12. Line 46 - take out "our colorful dynamic world full of".

Response: Thank you for this excellent suggestion. We have removed the part, as follows:
“Species-specific AS is partly responsible for a wide variety of biodiversity with limited repertoires of protein coding genes.”

13. Line 6 "between conservation and AS" - What conservation? Sequence conservation? Gene functional conservation? Amino acid conservation? Protein structural conservation?

Response: Thank you for this excellent suggestion. We have revised the sentence, as follows:
“We performed a genome-wide investigation to determine the relationship between amino acid conservation and AS and examine the evolution of AS status of genes that are involved in the lignin biosynthesis.”

14. Line 6 - change "between evolution and the AS status of genes …" to "examine the evolution of AS status of genes …"

Response: Thank you for this excellent suggestion. We have revised the sentence, as follows:
“We performed a genome-wide investigation to determine the relationship between amino acid conservation and AS and examine the evolution of AS status of genes that are involved in the lignin biosynthesis.”

Data description

15. Line 21 - change "different strategies" to "different sequencing strategies".

Response: Thank you for this excellent suggestion. We have revised the sentence, as follows:
“For the assembly of the moso bamboo genome, approximately 603.3 Gb genome data with different sequencing strategies were generated.”

Analyses
16. Line 53 - "assembly" statistics.

Response: Thank you for this excellent suggestion. We have revised the sentence, as follows:
“Compared with those of our previous version[8], the assembly statistics and quality of the new WGS assembly were obviously improved (Additional Tables S9-10).”

17. Line 34 - change "was higher than" to "more complete than"

Response: Thank you for this excellent suggestion. We have revised the sentence, as follows:
“According to the completeness assessment of the annotation using BUSCO, moso bamboo (95.2%) was more complete than Z. mays (92.2%) but close to O. sativa (95.6%).”

18. Line 58 - what is "post-regulation level"? you meant post-translational level?

Response: Thank you for this excellent suggestion. We have revised the sentence, as follows:
“To facilitate the genome-wide investigation of the AS landscape in moso bamboo and comprehensively identify the factors that influence AS at the post-translational level, we performed high-throughput RNA sequencing (RNA-Seq) using the Illumina HiSeq-4000 platform.”

19. Line 25 - "RNA from a mixture of … " this sentence is unclear.

Response: Thank you for this excellent suggestion. We have revised the sentence, as follows:
“The full-length cDNA sequencing of alternatively spliced isoforms (Iso-Seq) used RNA from a mixture of 26 samples.”

20. Line 48 - "…uniform AS events…" You meant "unique AS events"?

Response: We appreciate this observation. The number of the total AS events identified in our study were counted after removing repeated AS events in all 26 samples. Therefore, the word “unique” properly expressed the meaning and we have revised the sentence, as follows:
“In total, 266,711 unique AS events were identified in 25,225 AS genes, accounting for ca. 49.39% of all annotated genes.”

21. Line 4 - what are the four AS types? You need to introduce them first.

Response: Thank you for this excellent suggestion. We have added the introduction in Page 7, as follows:
"In subsequent analyses, we defined the four main AS types represented intron retention (IR), alternative 3' splice site donor (A3SS), alternative 5' splice site acceptor (A5SS), and exon skipping (ES) [10], and we also defined the other AS types represented some AS types except the above four main AS types."

22. Line 6 - "A higher accuracy is a strong indicator of …" A higher accuracy of what?

Response: Thank you for this excellent suggestion. We have revised the sentence, as follows:
"Thus, a higher proportion of the PacBio-Illumina overlapping AS genes is a strong indicator of the validity of the computationally predicted AS"

23. Line 38 - change "were detected to TE-insertion" to "have TE insertion"

Response: Thank you for this excellent suggestion. We have revised the sentence, as follows:
"The transposable element (TE) analysis showed 26,366 genes have TE insertion, accounted for 51.62% of all genes, and the total length of TE-insertion in genes was ~46 Mb."

24. Line 1 - Define D1-D8

Response: Thank you for this excellent suggestion. Based on the genome-wide identification of orthologous genes in the selected 8 plants (Amborella trichopoda, A. thaliana, Elaeis guineensis, B. distachyon, O. sativa, Spirodela polyrhiza, S. bicolor and Ph. edulis) and the species divergence time in a phylogeny tree (Fig. 3a), we identified eight orthologous gene datasets. For instance, dataset8 (D8) represents common orthologous genes in the selected 8 plants, which were located in an early divergence time in the phylogeny tree. D7 represents common orthologous genes in the selected 7 plants except A. trichopoda (the specie with the earliest divergence time) and D7 doesn't contain orthologues genes in D8, and so on. Thus, D1 represents bamboo-specific orthologous genes, which were located in later divergence time. According to a previous study [3], we obtained the divergence times of genes based on the presence and absence of orthologs in the phylogeny. In our subsequent study, thus, we considered the bamboo-specific genes (D1) as a poorly conserved gene dataset and the common genes in all selected plants (D8) as a highly conserved gene dataset, and the degree of conservation decreased monotonically from D8 to D1.

25. Line 6 - which statistic test you used to derive this p value?

Response: Thank you for this excellent suggestion. We used Mann-Whitney U test for P value and we have revised the sentence, as follows:
"AS was detected in all datasets, but the proportion of AS genes in each dataset gradually

decreased from D8 to D1 (Mann-Whitney U test with $p<0.05$).”

26. Line 8 - what do the "original dataset", "overlapping genes", and "duplicated genes" mean here?

Response: Thank you for this excellent suggestion. We have revised the part, as follows: “This trend was also observed in the two other datasets, i.e., removing common genes in more than two gene datasets in eight original datasets and using single-copy genes in eight original datasets. The eight-original dataset was derived from the genome-wide identification of orthologous genes in the selected 8 plants.”

27. Line 15 - change "abundance" to "percentage"

Response: Thank you for this excellent suggestion. We have revised the sentence, as follows:
“A high percentage (>75%) of AS events was observed in the 4-coumarate: CoA ligase (4CL), hydroxycinnamoyl transferase (HCT) and cinnamyl alcohol dehydrogenase (CAD) gene families.”

Discussion
28. Line 33 - please rephrase this sentence.

Response: Thank you for this excellent suggestion. We have revised the sentence, as follows:
“High-throughput genome sequencing and assembly strategy were broadly applied in current plant genomic studies with the development of new technologies and more useful data.”

29. Line 13 - "in addition to the protein-coding genes AS generates diverse transcripts of non-coding genes" Citation is needed here

Response: Thank you for this excellent suggestion. We have removed the sentence.

29. Line 35 - I do not follow the logic here. You found "no noticeable relationship between TE genes and AS genes", but you suggested that "TE might be a driving force during the formation process of AS in bamboo"?

Response: Thank you for this excellent suggestion. A previous study [11] shown TEs constitute crucial gene regulatory elements and influence gene transcription and gene expression. However, the noticeable relationship between TE genes and AS genes was unavailable in our study. Therefore, combined with our result and the previous study, we had implied that TE might be not a main reason of generating alternative splicing and might be a driving force during the formation process of AS in bamboo, although the mechanism of AS formation is still unknown.

30. Line 28 - what do you mean by "redundancy" here?

Response: Thank you for this excellent suggestion. The redundancy means some genes appeared in more than gene datasets and we have revised the sentence, as follows:
“This finding was robust based on we analyzed using the orthologous genes only in one dataset and using single-copy genes in selected species, respectively.”

31. Line 34 - take out "As a necessary substrate for the evolution of AS".

Response: Thank you for this excellent suggestion. We have removed the part, as follows:
“New genes might first generate a single-functional gene without an AS event and then gradually form multifunctional and conserved genes with many AS events [12]”

32. Line 47 - what are the "many other AS types"?

Response: We appreciate this observation. Many AS types represented some AS types except the main four AS types and we have added the introduction in Page 7, as follows:
“In subsequent analyses, we defined the four main AS types represented intron retention (IR), alternative 3’ splice site donor (A3SS), alternative 5’ splice site acceptor (A5SS), and exon skipping (ES) [10], and we also defined the other AS types represented some AS types except the above four main AS types.”

33. Line 49 - there is no way you could infer the "intermediate evolutionary stage". Plus I couldn't figure out what are the other AS types.

Response: We appreciate this observation. We have added the introduction to the other AS types in Page 7 (see the above answer for details) and revised the sentence, as follows:
“Thus, the four main AS types were conserved, and other types might represent an intermediate stage”

34. Line 2 - line 36 please revise this paragraph. I could not follow the logic nor find the main point.

Response: Thank you for this excellent suggestion. We have greatly revised the paragraph, as follows:
“According to our results, the highly conserved gene datasets had more AS genes and events, which either produce functional alternative protein-coding transcripts with distinct functions in biological processes or modulate the functional spliced transcript level by producing certain non-coding transcripts [12]. We hypothesize that the highly conserved genes with more AS events might be critical for evolution and function in generating gene functional diversity and the generation process of the highly conserved genes might undergo rigorous regulation during long-term evolution since the poorly conserved genes had less AS events than the highly conserved genes. Additionally, compared with the poorly conserved gene datasets, the highly conserved AS gene datasets had a low tissue-specific expression profile, indicating these genes might be core genes in fundamental functions, such as serving

as hubs in gene-gene networks. Therefore, we proposed that functionally important genes are generated by more frequent AS events. As an essential biological process, AS plays a crucial role in acquiring more functions, which might explain why the highly conserved AS possesses more AS events. We also hypothesize that this phenomenon likely applies not only to bamboo but also to other plants or even animals."

35. Line 10 - why having more AS events would have "functional priority"?
Response: We appreciate this observation. We have removed the confused description and revised the related description, as follows:
"In bamboo, the HCT family has more members and AS events than the CHS family, which indicate that the HCT family might be in a dominant position in the competition to bind p-coumaroyl CoA."

36. Line 41 - "uniform" you meant "unique"?

Response: Thank you for this excellent suggestion. We have revised the sentence, as follows:
"Based on the chromosome-level genome sequence and the abundant transcriptomic data from multiple tissues from six main bamboo producing areas in China, we provide a comprehensive AS perspective of moso bamboo by identifying 266,711 unique AS events in 25,225 AS genes using both the Illumina and PacBio sequencing technology platforms."

References:
1. Simão FA, Waterhouse RM, Ioannidis P, Kriventseva EV, Zdobnov EM. BUSCO: assessing genome assembly and annotation completeness with single-copy orthologs. Bioinformatics. 2015;31:3210–2.
2. Kim D, Langmead B, Salzberg SL. HISAT: a fast spliced aligner with low memory requirements. Nature Methods. 2015;12:357–60.
3. Zhang YE, Vibranovski MD, Landback P, Marais GAB, Long M. Chromosomal redistribution of male-biased genes in mammalian evolution with two bursts of gene gain on the X chromosome. Barton NH, editor. PLoS Biology. 2010;8:e1000494.
4. Chen F, Mackey AJ, Stoeckert CJ, Roos DS. OrthoMCL-DB: querying a comprehensive multi-species collection of ortholog groups. Nucleic Acids Research. 2006;34:D363–8.
5. Guindon S, Dufayard J-F, Lefort V, Anisimova M, Hordijk W, Gascuel O. New algorithms and methods to estimate maximum-likelihood phylogenies: assessing the performance of PhyML 3.0. Systematic Biology. 2010;59:307–21.
6. Yang Z. PAML 4: phylogenetic analysis by maximum likelihood. Molecular Biology and Evolution. 2007;24:1586–91.
7. International Brachypodium Initiative. Genome sequencing and analysis of the model grass Brachypodium distachyon. Nature. 2010;463:763–8.
8. Peng Z, Lu Y, Li L, Zhao Q, Feng Q, Gao Z, et al. The draft genome of the fast-growing non-timber forest species moso bamboo (Phyllostachys heterocycla). Nature Genetics. 2013;45:456–61.
9. Taylor JS, Raes J. Duplication and divergence: the evolution of new genes and old ideas. Annual Review of Genetics. 2004;38:615–43.
10. Barbosa-Morais NL, Irimia M, Pan Q, Xiong HY, Gueroussov S, Lee LJ, et al. The

Evolutionary Landscape of Alternative Splicing in Vertebrate Species. Science. 2012;338:1587–93.
11. Slotkin RK, Martienssen R. Transposable elements and the epigenetic regulation of the genome. Nature Reviews Genetics. 2007;8:272–85.
12. Roy SW, Irimia M. Splicing in the eukaryotic ancestor: form, function and dysfunction. Trends in Ecology and Evolution. 2009;24:447–55.
Close
