# Supplementary material for: Chromosome-level reference genome and alternative splicing atlas of moso bamboo (Phyllostachys edulis)
Source: Gigascience. 2018 Sep 8;7(10):giy115. doi: 10.1093/gigascience/giy115 (PMC6204424; doi:10.1093/gigascience/giy115)
Supplement: Response_to_Reviewer_Comments_Report_Revision_1.pdf [file giy115_response_to_reviewer_comments_report_revision_1.pdf]

Author's Response To Reviewer Comments

Close

Scott Edmunds
Executive Editor
GigaScience

4 Jul 2018

Dear Dr. Scott,

Re: Manuscript reference No. GIGA-D-18-00076R1
Please find attached a latest revised version (Revision 2) of our manuscript "Chromosome-level reference genome and alternative splicing atlas of moso bamboo (Phyllostachys edulis)", which we would like to resubmit for publication as a research article in GigaScience.

The comments of the reviewers were highly insightful and enabled us to greatly improve the quality of our manuscript. In the following pages are our point-by-point responses to each of the comments and suggestions of the reviewer.

Revision 2 in the text is shown using red highlight based on Revision 1. We hope that these revisions in the manuscript and our accompanying responses will be sufficient to make our manuscript suitable for publication in GigaScience.

We shall look forward to hearing from you at your earliest convenience.

Yours sincerely,

Prof. Hansheng Zhao
Address: No. 8, Fu Tong Dong Da Jie, Chaoyang District, Beijing 100102, P.R. China
Tel: +86-010-8478 9804
Fax: +86-010-8478 9802
E-mail: zhaohansheng@icbr.ac.cn

Responses to comments of Reviewer #2

The authors have clarified some of the issues I raised, but I still have a few comments/suggestions/edits. Please also see the marked word document attached.
Note the page and line numbers below are based on the attached word file.

1.Page 2, line 12. What does "uniform" mean here. You meant "unique"?

Response: Thank you for this excellent suggestion. According to your suggestion, we have revised the sentence, as follows:

“we provide a comprehensive AS profile based on the identification of 266,711 unique AS events in 25,225 AS genes by large-scale transcriptomic sequencing of 26 representative bamboo tissues using both the Illumina and PacBio sequencing platforms.”

2. Page 2, line 16. Please be specific about "specificity" here. You meant "tissue specificity"?

Response: Thank you for this excellent suggestion. Indeed, the description of “tissue specificity” was more proper and we have revised the sentence, as follows:
“Via comparison with orthologous genes in related plant species, we observed that the AS genes are concentrated in more conserved genes that tend to accumulate higher expressed transcripts and share less tissue specificity.”

3. Page 2, line 17. This sentence does not make sense. AS and positive selection on lignin biosynthesis do not indicate moso bamboo is a woody plant.

Response: Thank you for this excellent suggestion. We have removed the confused description, as follows:
“Furthermore, gene family expansion, abundant AS and positive selection were identified in crucial genes involved in the lignin biosynthesis pathway of moso bamboo.”

4. Page 4, line 6. What is "evolutionary landscape"?

Response: Thank you for this excellent suggestion. In the latest submission, we have used the description of “evolutionary aspect”, instead of “evolutionary landscape”, as follows:
“In conclusion, our analysis not only provides a global profile of AS in bamboo for further experimental studies investigating the functions of genes and regulatory networks but also reveals the roles of AS from the evolutionary aspect.”

5. Page 7, line 2. "the four main AS types" appears too suddenly here. You'd need to explain what these four types are first.

Response: Thank you for this excellent suggestion. We have transferred the related explanation to the first mentioned place of the four main AS types, as follows:
“In subsequent analyses, we defined the four main AS types as: intron retention (IR), alternative 3’ splice site donor (A3SS), alternative 5’ splice site acceptor (A5SS), and exon skipping (ES), and we also defined the other AS types represented some AS types except for the above four main AS types. Then, we found that on average, 80.37% of the AS events and 95.59% of the AS genes overlapped among the four main AS types (Additional Fig. S17).”

6. Page 7, line 5-6. This correlation was found among the four AS types? If so, you'd need to describe the four AS types first, and then say you found correlation of event number and gene number among the four AS types.

Response: Thank you for this excellent suggestion. According to your suggestion, we have transferred the related explanation to the first mentioned place of the four main AS types and provided the correlation among the four main AS types, as follows:
“In subsequent analyses, we defined the four main AS types as: intron retention (IR), alternative 3’ splice site donor (A3SS), alternative 5’ splice site acceptor (A5SS), and exon skipping (ES), and we also defined the other AS types represented some AS types except for the above four main AS types.”
“The AS event number was strongly and positively correlated with the AS gene number and those among the four main AS types (R2>0.91, Mann-Whitney U test with p value <0.05) (Fig. 2c).”

7. Page 7, line 18. You have two "two-thirds" here, which does not make sense mathematically.

Response: Thank you very much for pointing out this error. We have revised the sentence, as follows:
“Since AS possess strong specificity to different tissues or developmental stages, we identified 181,105 tissue-specific AS events (67.57%), which account for two-thirds of the AS events (termed as among-tissue). Then, the remaining one-third of the AS events were detected based on comparisons of the transcript isoforms within individual tissues (termed as within-tissue) (Additional Fig. S18).”

8. Page 8, line 4. Why do species divergence estimates have anything to do with ortholog classification you described in the following?

Response: Thank you for this excellent suggestion. We had performed a genome-wide classification of orthologous genes in the 8 groups. These groups were identified based on the species tree (Fig. 3a). The species divergence time was used to exhibit the origination time of genes in different datasets. The Fig. 3a facilitated to vividly exhibit the relationship. Additionally, we have revised the related description and added the description of origination time, as follows:
“Based on the genome-wide identification of orthologous genes in the selected 8 plant species (Amborella trichopoda, A. thaliana, Elaeis guineensis, B. distachyon, O. sativa, Spirodela polyrhiza, S. bicolor and Ph. edulis) and the constructed phylogeny (Fig. 3a), we identified eight unique orthologous gene datasets (D8-D1) based on the origination times of genes in each dataset. For instance, unique orthologous gene dataset 7 (D7) only contained orthologous genes which originated between 164.9 million year ago (Mya) and 213.6 Mya. In addition, we also extracted single-copy genes respectively from above datasets and termed as D8s-D1s.”

9. Page 8, line 6. "which were located in an early divergence time in our constructed phylogeny" this statement does not make sense. You cannot "locate" in a "time" in a "phylogeny".

Response: Thank you for this excellent suggestion. We have revised the description, as follows:
“For instance, unique orthologous gene dataset 7 (D7) only contained orthologous genes

which originated between 164.9 million year ago (Mya) and 213.6 Mya.”

10. Page 8, line 8. "According to a previous study [27], we obtained the divergence times of genes based on the presence and absence of orthologs in the phylogeny" please clarify this sentence. Presence/absence of orthologs cannot tell you the divergence of genes.

Response: Thank you for this excellent suggestion. According to your suggestion, we have removed the description.

11. Page 8, line 12. Please clarify "removing common genes in more than two gene datasets in eight original datasets and using single-copy genes in eight original datasets". I could not follow. What does "genes in more than two gene datasets" mean?

Response: Thank you for this excellent suggestion. We have revised the description, as follows:
“This trend was also observed in the single-copy datasets (D8s-D1s).”

12. Page 8, line 16. Did you do statistical test on all these four datasets?

Response: Thank you for this excellent suggestion. We have conducted a Chi square test on each corresponding orthologous group between D8-D1 and D8s-D1s (for example: D7 vs D7s), with p-value ranging from 0.86 to 0.98. Therefore, we concluded that the identical trends were detected in the two datasets. Additionally, we have revised the description, as follows:
“We investigated the distribution pattern of the four focal AS types in each dataset and found the identical trends (Fig. 3b), but the proportion of the AS types differed (IR>A3SS>A5SS>ES, Chi square test with p-value >0.86).”

13. Page 8, line 21-25. Again, what is the statistical result?

Response: Thank you for this excellent suggestion. We have conducted Pearson correlation between the median of maxTs and origination time in each group on D8-D1 (R2=0.863 and p value <0.01). Additionally, we have revised the description, as follows:
“Additionally, we compared with the AS events among the genes expressed in samples with different tissue specificities (maxTs) (for details, see Methods). The maxTs=1 and maxTs=0 represented constitutive expression and tissue specific expression, respectively. We found that the maxTs was negatively correlated with the origination time of the genes in D8-D1 (R2 > 0.86 and p value <0.01), representing an enhancement in the tissue specificity from a highly conserved gene dataset to a poorly conserved dataset (Fig. 3d).”

14. Page 8, line 25. "specificity" you meant "tissue specificity"?

Response: Thank you for this excellent suggestion. We have revised the sentence, as follows:
“Additionally, compared with the AS events among the genes expressed in samples with

different specificities (maxTs) (for details, see Methods), the maxTs obviously increased from D8 to D1, representing an enhancement in the tissue specificity from a highly conserved gene dataset to a poorly conserved dataset (Fig. 3d).

15. Page9, line 11-16. A lot of this can go into the method.

Response: Thank you for this excellent suggestion. We have revised the part and transferred the related description to the Method, as follows:

Analysis:
"Additionally, the divergence time of the gene involved in the lignin biosynthesis pathway (Additional Fig. S22) occurred at the 5~16 Mya, which correspond to the whole genome duplication (WGD) time 7~12 Mya in the moso bamboo genome."

Method:
"We calculated the synonymous substitution rate analysis for 13 gene families evolved in the lignin biosynthesis using the yn00, which was a package in PAML to estimate synonymous and nonsynonymous substitution rates. Then, the Ks rate was translated to the divergence time by the formula T=Ks/2r (r=6.5×10-9)."

16. Page 9 Discussion. Discussion in the current form is poorly organized. There are redundant points appearing in multiple paragraphs. Adding subheadings would help.

Response: Thank you for this excellent suggestion. According to your suggestion and the author instruction of GigaScience, we have removed redundant description and added the subheading. Please see the new revision for details due to many modifications.

17. Page 9, line 28. "High-throughput" is not appropriate to describe "assembly strategy". Also this sentence is written as like you developed new technologies, but I don't think so?

Response: Thank you for this excellent suggestion. We have revised the sentence, as follows:
"High-throughput genome sequencing and improved assembly strategy were broadly applied in current plant genomic studies with the development of new technologies and more useful data."

18. Page 10, line 1-2. I still do not understand why TE could be "a driving force during the formation process of AS in bamboo". Where is the evidence?

Response: Thank you for this excellent suggestion. We have removed the description.

19. Page 11, "More AS events were identified in the sample with vigorous growth, which is consistent with the previous studies" - this was not mentioned in the Results.

Response: Thank you for this excellent suggestion. We have removed the description in

discussion.

20. Page 11, "Obvious differences were observed in the AS event numbers in the final three shoot developmental stages, likely contributing to the fast growth during shoot development" - this was not mentioned in the Results. Also please provide statistical results to support "obvious".

Response: Thank you for this excellent suggestion. We have removed the description in discussion.

21. Page 11, line 23-24. I couldn't follow this sentence: "This finding was robust because we analyzed using the orthologous genes only in one dataset and using single-copy genes in selected species, respectively."

Response: Thank you for this excellent suggestion. We have revised the description, as follows:
“This finding was robust because we found the identical trends in the two types of eight gene datasets (D8-D1 and D8s-D1s).”

22. Page 11 line 27. I found the discussion on "new genes" is not well thought out. For example, the "hub genes" or "conserved genes" should have less functional diversity, not higher as the authors asserted here. I suggest drop this section.

Response: Thank you for this excellent suggestion. According to the references focusing on new genes, we have tried to revise the description, as follows:
“Previous reports have demonstrated that duplication is a major source of functional diversity and the generation of new genes [35], and conserved genes tend to have higher connectivity in gene-gene interaction networks, indicating their functional importance, while new genes were firstly added into gene-gene interaction networks with low connectivity and then gradually increased their connectivity and acquire pleiotropic roles [22,36]. In our study, highly conserved genes tended to have more AS events than poorly ones, which was consistent with the trend that conserved genes were apt to have higher connectivity in gene-gene interaction networks. Thus, we proposed that the AS may be associated with the increase of gene connectivity during evolution.”

23. Page 12, line 3-5. "Additionally, the four main AS types were abundant in the highly conserved gene datasets, and many other AS types appeared in the poorly conserved datasets. Thus, the four main AS types were conserved, and other types might represent an intermediate stage." I do not follow the logic here. Why AS of other types in the poorly conserved datasets would suggest they are "intermediate". I suggest remove this section.

Response: Thank you for this excellent suggestion. According to your suggestion, we have removed the section in discussion.

24. Page 12, line 14-18. "We hypothesize that the highly conserved genes with more AS

events might be critical for evolution and function in generating gene functional diversity and the generation process of the highly conserved genes might undergo rigorous regulation during long-term evolution since the poorly conserved genes had less AS events than the highly conserved genes." This sentence is too long and complicated. Please rephrase.

Response: Thank you for this excellent suggestion. We have rewritten the section and removed the redundant point in discussion, as follows:
“Previous reports have demonstrated that duplication is a major source of functional diversity and the generation of new genes [35], and conserved genes tend to have higher connectivity in gene-gene interaction networks, indicating their functional importance, while new genes were firstly added into gene-gene interaction networks with low connectivity and then gradually increased their connectivity and acquire pleiotropic roles [22,36]. In our study, highly conserved genes tended to have more AS events than poorly ones, which was consistent with the trend that conserved genes were apt to have higher connectivity in gene-gene interaction networks. Thus, we proposed that the AS may be associated with the increases of gene connectivity during evolution.”

25. Page 13, line 2-3. "During the evolutionary process, a new gene might be generated by duplication, which then forms less AS under strict constraints." Again this argument is flawed. A newly duplicated gene should have a "relaxed" functional constraint because a redundant copy is created.

Response: Thank you for this excellent suggestion. Indeed, the generation of a new genes was likely caused by either relaxation of functional constraint or positive Darwinian selection [1,2] and we have reorganized the section and removed the redundant point, as follows:
“Previous reports have demonstrated that duplication is a major source of functional diversity and the generation of new genes [35], and conserved genes tend to have higher connectivity in gene-gene interaction networks, indicating their functional importance, while new genes were firstly added into gene-gene interaction networks with low connectivity and then gradually increased their connectivity and acquire pleiotropic roles [22,36]. In our study, highly conserved genes tended to have more AS events than poorly ones, which was consistent with the trend that conserved genes were apt to have higher connectivity in gene-gene interaction networks. Thus, we proposed that the AS may be associated with the increases of gene connectivity during evolution.”

26. Page 13, line 19-20. What is the rationale that more AS would indicate "a dominant position in the competition to bind p-coumaroyl CoA"?

Response: Thank you for this excellent suggestion. HCT generates lignin by catalyzing p-coumaroyl CoA, which is also catalyzed by CHS to generate flavonoids. Thus, HCT and CHS compete with each other to bind p-coumaroyl CoA. In bamboo, the HCT family has more members and AS events than the CHS family as well as positive selection was detected in HCT family, which likely indicate that HCT family, compared to the CHS family, might be in a dominant position in the competition to bind p-coumaroyl CoA. Additionally, we have revised the description, as follows:
“In bamboo, the HCT family has more members and AS events than the CHS family, which likely indicate that the HCT family, compared to the CHS family, might be in a dominant

position in the competition to bind p-coumaroyl CoA.”

27. Page 14, line 12. What does "bamboo evolutionary landscape" mean?

Response: Thank you for this excellent suggestion. We have used the description of “evolutionary aspect”, instead of “evolutionary landscape”. Additionally, we have revised the description, as follows:
“In summary, these results will likely provide important resources for studies investigating bamboo’s unique woodiness in the Grass family (Poaceae) and exploring AS from the bamboo evolutionary aspect.”

28. Page 14, line 19. You meant "HuNan", not "HuHan" right?

Response: Thank you very much for pointing out this error. We have revised the sentence, as follows:
“(4) TaoJiang, HuNan Province (N:28°28′39.74″, E:112°11′18.62″, 320 M),”

29. Page 17, line 8. "identity > 95%"? Could you double check this threshold? Nucleotide identity > 95% is extremely stringent, and I cannot imagine you could get anything out.

Response: Thank you for this excellent suggestion. Indeed, the threshold was mistakes by double-checking our script and we have revised the description, as follows:
“Briefly, we performed standard protein BLAST searches (version 2.2.26) against the six genome sequences including moso bamboo using the coding sequence of the known genes with the following cut-off values: E-value <1e-10; identity > 40%; and coverage rate > 95% of query sequence.”

30. Figure 2. Change "PacBio" to "Iso-Seq".

Response: Thank you for this excellent suggestion. According to your suggestion, we have revised the Figure 2.

31. Figure 3C. It is unclear to me what this panel is showing. The figure legend also did not help much.

Response: Thank you for this excellent suggestion. According to your suggestion, we have revised the Figure 3C.

32. Figure 3D. Explain the y-axis: what does "number" and "rate" refer to? Also the x-axis "Species" should be replaced by something like "Datasets" right?

Response: Thank you for this excellent suggestion. Number and Rate refer to AS number and maxTs. According to your suggestion, we have revised the Figure 3D.

Responses to the comments of Reviewer #3

Reviewer #3: The manuscript presents a comprehensive assembly of the bamboo genome which provides assembled chromosomes; an improvement from the current more fragmented assembly for the species. It also identifies alternative splicing events from transcriptome data corresponding to 26 different tissues.
I believe that the data presented will be of great use to the scientific community, in particular those working on genomics and those interested in transcriptomics and alternative splicing.

Main comments:
1. FOCUS AND JUSTIFICATION. However, I think that study could be better justified and given a focus. While the relevance of having a more complete genome for an important plant is well justified, it is not clear how this relates to alternative splicing. There is also no justification as to why examining alternative splicing is important. For example, in the abstract it is stated that the paper assembles the genome and identifies alternative splicing events but does not explain WHY has alternative splicing was an important aspect to explore in a paper presenting a more complete assembly of the bamboo genome. Thus, one has the impression that this paper contains two separate stories running side by side.
Perhaps one solution is to explain that gene duplication and alternative splicing are important drivers of functional evolution in genomes. That incomplete and fragmented scaffolds of genomes makes it difficult to assess patterns of gene duplication and that low coverage transcriptomes of only a handful of tissues does not allow to fully understand the extent of alternative splicing. Thus, having a fully assembled genome as well as an extensive RNA sequencing for recovering RNA isoforms is required. It is important to explain in detail WHY is alternative splicing important.

Response: Thank you for this excellent suggestion. According to your suggestion, we have added the information in the Background, as follows:

“The incomplete and scattered scaffolds of moso bamboo genome and the low coverage transcriptomes of a handful of tissues make it difficult to fully dissert the AS profiles. Therefore, a high-quality assembled genome and an extensive RNA sequencing are critical for the comprehensive AS identification.”

2. JUSTIFICATION AND ORDER OF SPECIFIC ANALYSES. It is unclear to me why the description of the transposable element content should be in the section discussing alternative splicing events rather than on the section describing the genome sequence obtained.

Response: Thank you for this excellent suggestion. Due to the major modification, the description of the transposable element was removed in the Discussion.

3. DISCUSSION. Justification and relevance of many of the tests done is more evident in the discussion some of this information would be better placed in the introduction or as brief

sentences in the results so that the analyses make sense as they are presented.

Response: Thank you for this excellent suggestion. According to your suggestion, we have majorly revised the Discussion. Please see the details in the latest revision.

More specific points

4. On the evolution of AS section specify how many orthologs were found when comparing between species and what percentage of the total number of genes in the bamboo this represents. Even if these numbers are shown in the figure/tables it is always helpful to get an idea of the patterns from the text alone.

Response: Thank you for this excellent suggestion. According to your suggestion, we have added the orthologs number in the main text, as follows:

"We considered the bamboo-specific genes (4,023 orthologous genes; termed as D1) are poorly conserved, whereas the genes present in all selected plant species (18,997 orthologous genes; termed as D8) are highly conserved."

5. On the evolution of AS section it is not clear what analysis was done to compare the ortholog genes in other species. If I understand correctly, the analyses tries to assess whether having an older ortholog is associated with higher or lower rates of alternative splicing in the bamboo genome? This needs to be better worded. It is also important to explain WHY would this pattern be interesting important to understand evolution of alternative splicing. There is also a reference to a "robust pattern", does this refer to past literature in other species? If so then this needs to be better explained.

If actual conservation or overall patterns of AS in other plants, rather than presence absence of ortholog genes, were compared to the bamboo then this needs to be better explained as at the moment it is very unclear.

Response: Thank you for this excellent suggestion. Your understanding of this part is correct. Indeed, we tried to assess whether having an older ortholog is associated with higher or lower rates of alternative splicing in the bamboo genome? We then found that more conserved genes had more AS genes in bamboo. We are sorry for confusions. According to your suggestion, we have greatly revised this part, as follows:

"Based on the genome-wide identification of orthologous genes in the selected 8 plant species (Amborella trichopoda, A. thaliana, Elaeis guineensis, B. distachyon, O. sativa, Spirodela polyrhiza, S. bicolor and Ph. edulis) and the constructed phylogeny (Fig. 3a), we identified eight unique orthologous gene datasets (D8-D1) based on the origination times of genes in each dataset. For instance, unique orthologous gene dataset 7 (D7) only contained orthologous genes which originated between 164.9 million year ago (Mya) and 213.6 Mya. In addition, we also extracted single-copy genes respectively from above datasets and termed as D8s-D1s. We considered the bamboo-specific genes (4,023 orthologous genes; termed as D1) are poorly conserved, whereas the genes present in all selected plant species (18,997 orthologous genes; termed as D8) are highly conserved. The degree of conservation decreased monotonically from D8 to D1. AS was detected in all the datasets, but the proportion of AS genes in each dataset gradually decreased from D8 to D1 (Mann-Whitney U test with p value <0.05). This trend was also observed in the single-copy datasets (D8s-D1s). Therefore, the result was robust that more conserved genes had more AS genes in bamboo."

6. WHY the authors examine proportions of AS events by type is not entirely clear. There is an extensive literature on the patterns of prevalence of AS types as well as the differences in their potential contribution to functional adaptation.

Response: Thank you for this excellent suggestion. The difference in the frequencies or proportions of the AS types may reflect differences in their pre-mRNA splicing and this analysis is common in most genome-wide identification of AS. Thus, we examined the proportion of AS types and provided the details of bamboo for comparative analysis. Additionally, we have revised the description, as follows:
"The difference in the frequencies or proportions of the AS types may reflect differences in their pre-mRNA splicing and this analysis is common in most genome-wide identification of AS. The distribution of the AS types depicted that IR occupied the dominant position, indicating that the importance of IR could be inferred from inspecting its prevalence throughout evolution in plants. Nevertheless, a higher percentage of IR (38.22%) and other AS types (total 28.18%) were observed in bamboo."

7. It is unclear what the correlations for CDS length and intron number, etc. involved. Is this to correlate these parameters among the bamboo with its ortholog in the other plants? It is also not explained WHY was this done.

Response: Thank you for this excellent suggestion. To obtain an overview of the landscape of AS and its relationships with gene features, and to evaluate the factors that influence AS, we perform correlations between AS distribution and some gene features (e.g. the gene length, CDS length, intron length, exon number, exon cassette length, and intron cassette length) in the datasets. Additionally, we have revised the description, as follows:
"To obtain an overview of the landscape of AS and its relationships with gene features and to evaluate the factors that influence AS, we also examined the correlations between AS distribution and gene features in the datasets (Additional Fig. S21)."

8. In the last section of the results, the title implies that the EVOLUTION of gene families was assessed. However the text below does not give any details of how was this done or whether there has actually been an expansion and if so, with respect to WHAT species… It is also not explained WHY only 13 gene families were assessed.

Response: Thank you for this excellent suggestion. To better understand the identification of gene involved in the lignin biosynthetic pathway, we have revised the related method in the following. Additionally, according to the identification described previously [3], 13 gene families belong to the lignin biosynthesis pathway.

In the method
"Genome-wide identification of genes involved in the lignin biosynthetic pathway

The five genome sequences of A. thaliana (TAIR10), B. distachyon (v3.1), O. sativa (v7.0), Populus trichocarpa (JGI2.0.31), and S. bicolor (v3.1) were downloaded from the ENSEMBL database [4]. According to our literature-based investigations, 140 genes from the lignin biosynthetic pathway was experimentally validated from previous studies

(Additional Table S28), and then, these known genes were collected and used as the query sequences for further identification. We identified lignin biosynthetic genes using a BLAST search and domain analysis as described in a previous article[5]. Briefly, we performed standard protein BLAST searches (version 2.2.26) against the six genome sequences including moso bamboo using the coding sequence of the known genes with the following cut-off values: E-value <1e-10; identity >40%; and coverage rate >95% query sequence. The filtered sequences were subsequently analyzed by hmmsearch (version 3.1b2) using the Pfam-A.hmm database (released 2017/03/31). Consequently, unclear sequences with incomplete domains were discarded after manual correction. Phylogenetic analyses were carried out following. We also calculated the synonymous substitution rate analysis for 13 gene families evolved in the lignin biosynthesis using the yn00, which was a package in PAML to estimate synonymous and nonsynonymous substitution rates. Then, the Ks rate was translated to the divergence time by the formula T=Ks/2r (r=6.5×10-9)."

9. The discussion states that the paper presents evidence consistent with the role of TE in driving AS. The results only present a description of the rates of AS and the presence of TEs. If one drives the other then some analysis to link the two should be presented.

Response: Thank you for this excellent suggestion. According to your and other reviewer's suggestion, we have removed the description in Discussion.

10. I could not find in the results section a reference to the sample with more vigorous growth to have higher AS as it is stated in the introduction. Could this be made more prominent so that when reading the discussion this result can be easily found.

Response: Thank you for this excellent suggestion. Due to greatly modification of Discussion, we have removed the description.

11. I think a better explanation of what is a poorly conserved dataset and a highly conserved dataset means.

Response: Thank you for this excellent suggestion. According to your suggestion, we have greatly revised the part of "Evolutionary analysis of AS in moso bamboo" and provided an explanation about the poorly/highly conserved dataset, as follows:
"We considered the bamboo-specific genes (4,023 orthologous genes; termed as D1) are poorly conserved, whereas the genes present in all selected plant species (18,997 orthologous genes; termed as D8) are highly conserved. The degree of conservation decreased monotonically from D8 to D1."

References:
1. Chen S, Zhang YE, Long M. New genes in Drosophila quickly become essential. Science. 2010;330:1682–5.
2. Long M, Betrán E, Thornton K, Wang W. The origin of new genes: glimpses from the young and old. Nature Reviews Genetics. Nature Publishing Group; 2003;4:865–75.
3. Vanholme R, Demedts B, Morreel K, Ralph J, Boerjan W. Lignin biosynthesis and

structure. PLANT PHYSIOLOGY. American Society of Plant Biologists; 2010;153:895–905.
4. Kersey PJ, Allen JE, Allot A, Barba M, Boddu S, Bolt BJ, et al. Ensembl Genomes 2018: an integrated omics infrastructure for non-vertebrate species. Nucleic Acids Res. 2018;46:D802–8.
5. Fischer S, Brunk BP, Chen F, Gao X, Harb OS, Iodice JB, et al. Using OrthoMCL to assign proteins to OrthoMCL-DB groups or to cluster proteomes into new ortholog groups. Curr Protoc Bioinformatics. Hoboken, NJ, USA: John Wiley & Sons, Inc; 2011;Chapter 6:Unit6.12.1–19.
6. Zhang YE, Vibranovski MD, Landback P, Marais GAB, Long M. Chromosomal redistribution of male-biased genes in mammalian evolution with two bursts of gene gain on the X chromosome. Barton NH, editor. PLoS Biol. Public Library of Science; 2010;8:e1000494.

Close
