# Supplementary material for: Chromosome-level reference genome and alternative splicing atlas of moso bamboo (Phyllostachys edulis)
Source: Gigascience. 2018 Sep 8;7(10):giy115. doi: 10.1093/gigascience/giy115 (PMC6204424; doi:10.1093/gigascience/giy115)
Supplement: Reviewer_1_Report_(Original_Submission) -- Cláudio Benício Cardoso-Silva, Ph.D — 3/27/2018 Reviewed [file giy115_reviewer_1_report_(original_submission).pdf]

Reviewer Report

Title: **Chromosome-level reference genome and alternative splicing atlas of moso bamboo (Phyllostachys edulis)**

Version: **Original Submission** **Date:** 3/27/2018

Reviewer name: **Cláudio Benício Cardoso-Silva, Ph.D**

Reviewer Comments to Author:

The authors provide a high-quality genome assembly and gene annotation of moso bamboo in order to improve the first version published in 2013. Transcriptomic analysis was performed using several tissues to identify alternative splicing events and polymorphism within gene transcription by providing a repertoire of alternative transcription that could support tissue specialization. Additionally, an evolutionary insight, especially regarding the genes involved in lignin biosynthesis.
The genomic resource described in this manuscript will facilitate future studies on the evolution and functional genomic of moso bamboo and other grasses by providing a valuable information to the researchers interested in this area.
I recommend the manuscript for publication, following some minor revision, which I have listed below by manuscript page (p.) and line (L) numbers.

p. 3 - L20: Instead of "…have investigated in bamboo…" should be '…have been investigated in bamboo...'.
p. 4 - L27: Please add suppl. table reference for transcriptomic data.
p. 4 - L30: It was found a conflicting value "…We identified 266,711 uniform AS…". In the abstract section, the number of transcripts mentioned is 266,771. Please insert the correct value.
p. 4 L49: In the sentence "…we performed the genome assembly using different strategies to obtain a better genome assembly.", I suggest indicating the additional reference for detailed steps of the genome assembly.
p. 5 L8: Instead of "…rice genome to find a mean coverage…" should be '…rice genome and we obtained a mean coverage…'.
p. 5 L13: About bamboo BAC sequences, I suggest mentioning that these sequences are derived from other bamboo specie (Ph heterocycla) in this section or in the additional table S6.
p. 5 L24: Provide correct reference - "…we predicted 51,074 high-quality protein-coding loci… (Additional Table S10)". Instead of Table S10, it should be Table S11.
p. 5 L30: Provide correct reference - "… ~17% of the gene models were precisely refined (Additional Table S11)". Instead of Table S11, it should be Table S12.
p. 5 L36: Provide correct reference - Regarding the annotation using BUSCO, the reference should be Additional Table S13 instead of S12 in "(Fig 1d and Additional Table S12)".
p. 7 L27: The additional table reference for the enrichment analysis should be S25 instead of S26.
p. 7 L32: "…which account for one-third of the AS events (termed as among-tissue)." According to additional Fig S18, the AS events classified as among-tissue correspond to two-third of the AS events.
p. 8 L43-45: The sentence "the distribution of the TE genes in the 8 datasets was examined. A substantially negative correlation" could be '…was examined and a substantially negative…'
p. 10 L28: "…a higher percentage of IR (38.22%) and other AS types (total 28.18%) were observed in bamboo." In order to avoid misunderstanding, I suggest clarifying that 'other AS types' represent a set of AS events except the main AS types already mentioned in the manuscript.
p. 16 L50: Please, provide release number of the pfam-A.hmm database.

Figures and Tables
Figure 2: The figure legend should explain the meaning of the acronyms IR, A3SS, A5SS, and ES.
Figure S3: In the figure legend "…The while boxes…" should be '…The white boxes…'
Fig. S17: Is the x-axis data label named 'AS' correct?
Table S1: Please provide the correct number of libraries in the 'Total' description.

Table S3: Asterisk with description in the legend is not shown in the table.
Table S28: I recommend excluding the words 'totally' and 'were' in the table legend.
Some figures and tables citation are missing in the manuscript, such as Fig. 1a, 1b, and 3d; and additional table S23.

Dataset
The PacBio reads (IsoSeq) must also be submitted to GiGADB or SRA and their accession number provided in the manuscript.

Methods

Are the methods appropriate to the aims of the study, are they well described, and are necessary controls included? Yes

Conclusions

Are the conclusions adequately supported by the data shown? Yes

Reporting Standards

Does the manuscript adhere to the journal's guidelines on minimum standards of reporting? Yes

Choose an item.

Statistics

Are you able to assess all statistics in the manuscript, including the appropriateness of statistical tests used? Yes, and I have assessed the statistics in my report.

Quality of Written English

Please indicate the quality of language in the manuscript: Acceptable

Declaration of Competing Interests

Please complete a declaration of competing interests, considering the following questions:

- Have you in the past five years received reimbursements, fees, funding, or salary from an organisation that may in any way gain or lose financially from the publication of this manuscript, either now or in the future?
- Do you hold any stocks or shares in an organisation that may in any way gain or lose financially from the publication of this manuscript, either now or in the future?
- Do you hold or are you currently applying for any patents relating to the content of the manuscript?
- Have you received reimbursements, fees, funding, or salary from an organization that holds or has applied for patents relating to the content of the manuscript?
- Do you have any other financial competing interests?

- Do you have any non-financial competing interests in relation to this paper?

If you can answer no to all of the above, write 'I declare that I have no competing interests' below. If your reply is yes to any, please give details below.

I declare that I have no competing interests.

I agree to the open peer review policy of the journal. I understand that my name will be included on my report to the authors and, if the manuscript is accepted for publication, my named report including any attachments I upload will be posted on the website along with the authors' responses. I agree for my report to be made available under an Open Access Creative Commons CC-BY license (http://creativecommons.org/licenses/by/4.0/). I understand that any comments which I do not wish to be included in my named report can be included as confidential comments to the editors, which will not be published.

I agree to the open peer review policy of the journal

To further support our reviewers, we have joined with Publons, where you can gain additional credit to further highlight your hard work (see: https://publons.com/journal/530/gigascience). On publication of this paper, your review will be automatically added to Publons, you can then choose whether or not to claim your Publons credit. I understand this statement.

Yes Choose an item.
