# Supplementary material for: Chromosome-level reference genome and alternative splicing atlas of moso bamboo (Phyllostachys edulis)
Source: Gigascience. 2018 Sep 8;7(10):giy115. doi: 10.1093/gigascience/giy115 (PMC6204424; doi:10.1093/gigascience/giy115)
Supplement: Reviewer_2_Report_(Original_Submission) -- Fay-Wei Li — 4/4/2018 Reviewed [file giy115_reviewer_2_report_(original_submission).pdf]

Reviewer Report

Title: **Chromosome-level reference genome and alternative splicing atlas of moso bamboo (Phyllostachys edulis)**

Version: **Original Submission** **Date:** 4/4/2018

Reviewer name: **Fay-Wei Li**

Reviewer Comments to Author:

Zhao et al reported a much improved genome assembly of moso bamboo, and characterized its alternative splicing (AS) atlas. While I find the assembly result very impressive, I have several questions on the methodology.

1. According to the method text in the "Additional File", the RNA reads were mapped onto the genome by "BLAT" and refined by HISAT. This is a rather unconventional way to map RNA-seq data to a reference genome. Why not just use HISAT2? BLAT was designed to align transcripts, not individual RNA-seq reads. Also I'm not aware of the adjustment function in HISAT. Please make sure the read mapping was done correctly because this is fundamental to the AS analysis.

2. One important conclusion the authors made is that the conserved genes tend to have more AS events. It is however unclear to me how the authors measured the degrees of conservation. The authors did a gene family classification, and "obtained 8 datasets of orthologous genes representing different levels of conservation (Fig. 3a) designated dataset8 (more conserved genes) to dataset1 (bamboo-specific genes) based on a phylogenetic relationship of 8 selected species." But there was no further explanation. What does the "most conserved genes" in dataset8 entail? Based on presence or absence? And what is the difference between, say dataset8 and dataset7? How gene families were clustered was also unexplained (at least I couldn't find it). These are critical details that are missing.

3. A species phylogeny was reconstructed from 8 genomes, but no information is provided about how this was done. What are the "single-copy orthologous genes"? What methods and programs you used for phylogenetic reconstruction?

4. Species divergence time was estimated, but again, the authors provided no methodological detail. How was the molecular clock estimated? Did you test the validity of assuming a molecular clock (e.g. relative rate test)? Further, the time calibrations listed in the Additional File need citations; they also to me look like secondary calibrations rather than "fossil time".

5.Though I appreciate the artistic value of Fig. 3A, it is scientifically incorrect (or at least very confusing). The x-axis is apparently in unit of substitution/site, which is a branch length measurement. It does not make sense to have a terminal tip linked (by vertical dashed line) to a branch length value. There was also no "divergence times" information in this figure and the legend should be revised.

6. The expansion of lignin biosynthesis genes could be due to whole genome duplication (WGD), but WGD was not discussed. Are the two decoupled?

Some other comments are listed below. The manuscript has no page number, and the line numbers does not match the actual lines and restart in each page, which make the review difficult. Anyway, I tried my best to point out where in the text I was referring to.

Abstract
Line 18 - what is "additional abundance data"? You meant sequencing data?

Line 31 - "dramatic evolutionary characteristics" is too dramatic and unclear. Please be specific or take out this sentence.
Line 39 - what does "bamboo's specificity in being a woody plant" mean? Please clarify "specificity".

Background
Line 20 - change "investigated" to "been carried out".
Line 46 - change "is responsible" to "is partly responsible".
Line 46 - take out "our colorful dynamic world full of".
Line 6 "between conservation and AS" - What conservation? Sequence conservation? Gene functional conservation? Amino acid conservation? Protein structural conservation?
Line 6 - change "between evolution and the AS status of genes …" to "examine the evolution of AS status of genes …"

Data description
Line 21 - change "different strategies" to "different sequencing strategies".

Analyses
Line 53 - "assembly" statistics.
Line 34 - change "was higher than" to "more complete than"
Line 58 - what is "post-regulation level"? you meant post-translational level?
Line 25 - "RNA from a mixture of … " this sentence is unclear.
Line 48 - "…uniform AS events…" You meant "unique AS events"?
Line 4 - what are the four AS types? You need to introduce them first.
Line 6 - "A higher accuracy is a strong indicator of …" A higher accuracy of what?
Line 38 - change "were detected to TE-insertion" to "have TE insertion"
Line 1 - Define D1-D8
Line 6 - which statistic test you used to derive this p value?
Line 8 - what do the "original dataset", "overlapping genes", and "duplicated genes" mean here?
Line 15 - change "abundance" to "percentage"

Discussion
Line 33 - please rephrase this sentence.
Line 13 - "in addition to the protein-coding genes AS generates diverse transcripts of non-coding genes" Citation is needed here
Line 35 - I do not follow the logic here. You found "no noticeable relationship between TE genes and AS genes", but you suggested that "TE might be a driving force during the formation process of AS in bamboo"?
Line 28 - what do you mean by "redundancy" here?
Line 34 - take out "As a necessary substrate for the evolution of AS".
Line 47 - what are the "many other AS types"?
Line 49 - there is no way you could infer the "intermediate evolutionary stage". Plus I couldn't figure out what are the other AS types.
Line 2 - line 36 please revise this paragraph. I could not follow the logic nor find the main point.
Line 10 - why having more AS events would have "functional priority"?
Line 41 - "uniform" you meant "unique"?

Methods

Are the methods appropriate to the aims of the study, are they well described, and are necessary controls included? No

Conclusions

Are the conclusions adequately supported by the data shown? No

Reporting Standards

Does the manuscript adhere to the journal's guidelines on minimum standards of reporting? No

Choose an item.

Statistics

Are you able to assess all statistics in the manuscript, including the appropriateness of statistical tests used? Yes, and I have assessed the statistics in my report.

Quality of Written English

Please indicate the quality of language in the manuscript: Not suitable for publication unless extensively edited

Declaration of Competing Interests

Please complete a declaration of competing interests, considering the following questions:

- Have you in the past five years received reimbursements, fees, funding, or salary from an organisation that may in any way gain or lose financially from the publication of this manuscript, either now or in the future?
- Do you hold any stocks or shares in an organisation that may in any way gain or lose financially from the publication of this manuscript, either now or in the future?
- Do you hold or are you currently applying for any patents relating to the content of the manuscript?
- Have you received reimbursements, fees, funding, or salary from an organization that holds or has applied for patents relating to the content of the manuscript?
- Do you have any other financial competing interests?
- Do you have any non-financial competing interests in relation to this paper?

If you can answer no to all of the above, write 'I declare that I have no competing interests' below. If your reply is yes to any, please give details below.

I declare that I have no competing interests.

I agree to the open peer review policy of the journal. I understand that my name will be included on my report to the authors and, if the manuscript is accepted for publication, my named report including any attachments I upload will be posted on the website along with the authors' responses. I agree for my report to be made available under an Open Access Creative Commons CC-BY license (http://creativecommons.org/licenses/by/4.0/). I understand that any comments which I do not wish to be included in my named report can be included as confidential comments to the editors, which will not be published.

I agree to the open peer review policy of the journal

To further support our reviewers, we have joined with Publons, where you can gain additional credit to further highlight your hard work (see: https://publons.com/journal/530/gigascience). On publication of this paper, your review will be automatically added to Publons, you can then choose whether or not to claim your Publons credit. I understand this statement.

Yes Choose an item.
