# Supplementary material for: Chromosome-level reference genome and alternative splicing atlas of moso bamboo (Phyllostachys edulis)
Source: Gigascience. 2018 Sep 8;7(10):giy115. doi: 10.1093/gigascience/giy115 (PMC6204424; doi:10.1093/gigascience/giy115)
Supplement: Reviewer_2_Report_Revision_1 -- Fay-Wei Li — 5/1/2018 Reviewed [file giy115_reviewer_2_report_revision_1.pdf]

Reviewer Report

Title: Chromosome-level reference genome and alternative splicing atlas of moso bamboo (Phyllostachys edulis)

Version: Revision 1 Date: 5/1/2018

Reviewer name: Fay-Wei Li

Reviewer Comments to Author:

The authors have clarified some of the issues I raised, but I still have a few comments/suggestions/edits. Please also see the marked word document attached.

Note the the page and line numbers below are based on the attached word file.

Page 2, line 12. What does "uniform" mean here. You meant "unique"?

Page 2, line 16. Please be specific about "specificity" here. You meant "tissue specificity"?

Page 2, line 17. This sentence does not make sense. AS and positive selection on lignin biosynthesis do not indicate moso bamboo is a woody plant.

Page 4, line 6. What is "evolutionary landscape"?

Page 7, line 2. "the four main AS types" appears too suddenly here. You'd need to explain what these four types are first.

Page 7, line 5-6. This correlation was found among the four AS types? If so, you'd need to describe the four AS types first, and then say you found correlation of event number and gene number among the four AS types.

Page 7, line 18. You have two "two-thirds" here, which does not make sense mathematically.

Page 8, line 4. Why do species divergence estimates have anything to do with ortholog classification you described in the following?

Page 8, line 6. "which were located in an early divergence time in our constructed phylogeny" this statement does not make sense. You cannot "locate" in a "time" in a "phylogeny".

Page 8, line 8. "According to a previous study [27], we obtained the divergence times of genes based on the presence and absence of orthologs in the phylogeny" please clarify this sentence. Presence/absence of orthologs cannot tell you the divergence of genes.

Page 8, line 12. Please clarify "removing common genes in more than two gene datasets in eight original datasets and using single-copy genes in eight original datasets". I could not follow. What does "genes in more than two gene datasets" mean?

Page 8, line 16. Did you do statistical test on all these four datasets?

Page 8, line 21-25. Again, what is the statistical result?

Page 8, line 25. "specificity" you meant "tissue specificity"?

Page9, line 11-16. A lot of this can go into the method.

Page 9 Discussion. Discussion in the current form is poorly organized. There are redundant points appearing in multiple paragraphs. Adding subheadings would help.

Page 9, line 28. "High-throughput" is not appropriate to describe "assembly strategy". Also this sentence is written as like you developed new technologies, but I don't think so?

Page 10, line 1-2. I still do not understand why TE could be "a driving force during the formation process of AS in bamboo". Where is the evidence?

Page 11, "More AS events were identified in the sample with vigorous growth, which is consistent with the previous studies" - this was not mentioned in the Results.

Page 11, "Obvious differences were observed in the AS event numbers in the final three shoot developmental stages, likely contributing to the fast growth during shoot development" - this was not mentioned in the Results. Also please provide statistical results to support "obvious".

Page 11, line 23-24. I couldn't follow this sentence: "This finding was robust because we analyzed using the orthologous genes only in one dataset and using single-copy genes in selected species, respectively."

Page 11 line 27. I found the discussion on "new genes" is not well thought out. For example, the "hub genes" or "conserved genes" should have less functional diversity, not higher as the authors asserted here. I suggest drop this section.

Page 12, line 3-5. "Additionally, the four main AS types were abundant in the highly conserved gene datasets, and many other AS types appeared in the poorly conserved datasets. Thus, the four main AS types were conserved, and other types might represent an intermediate stage." I do not follow the logic here. Why AS of other types in the poorly conserved datasets would suggest they are "intermediate". I suggest remove this section.

Page 12, line 14-18. "We hypothesize that the highly conserved genes with more AS events might be critical for evolution and function in generating gene functional diversity and the generation process of the highly conserved genes might undergo rigorous regulation during long-term evolution since the poorly conserved genes had less AS events than the highly conserved genes." This sentence is too long and complicated. Please rephrase.

Page 13, line 2-3. "During the evolutionary process, a new gene might be generated by duplication, which then forms less AS under strict constraints." Again this argument is flawed. A newly duplicated gene should have a "relaxed" functional constraint because a redundant copy is created.

Page 13, line 19-20. What is the rationale that more AS would indicate "a dominant position in the competition to bind p-coumaroyl CoA"?

Page 14, line 12. What does "bamboo evolutionary landscape" mean?

Page 14, line 19. You meant "HuNan", not "HuHan" right?

Page 17, line 8. "identity > 95%"? Could you double check this threshold? Nucleotide identity > 95% is extremely stringent, and I cannot imagine you could get anything out.

Figure 2. Change "PacBio" to "Iso-Seq".

Figure 3C. It is unclear to me what this panel is showing. The figure legend also did not help much.

Figure 3D. Explain the y-axis: what does "number" and "rate" refer to? Also the x-axis "Species" should be replaced by something like "Datasets" right? **Methods**

Are the methods appropriate to the aims of the study, are they well described, and are necessary controls included? Choose an item.

Conclusions

Are the conclusions adequately supported by the data shown? Choose an item.

Reporting Standards

Does the manuscript adhere to the journal's guidelines on minimum standards of reporting? Choose an item.

Choose an item.

Statistics

Are you able to assess all statistics in the manuscript, including the appropriateness of statistical tests used? Choose an item.

Quality of Written English

Please indicate the quality of language in the manuscript: Choose an item.

Declaration of Competing Interests

Please complete a declaration of competing interests, considering the following questions:

- Have you in the past five years received reimbursements, fees, funding, or salary from an organisation that may in any way gain or lose financially from the publication of this manuscript, either now or in the future?
- Do you hold any stocks or shares in an organisation that may in any way gain or lose financially from the publication of this manuscript, either now or in the future?
- Do you hold or are you currently applying for any patents relating to the content of the manuscript?
- Have you received reimbursements, fees, funding, or salary from an organization that holds or has applied for patents relating to the content of the manuscript?
- Do you have any other financial competing interests?
- Do you have any non-financial competing interests in relation to this paper?

If you can answer no to all of the above, write 'I declare that I have no competing interests' below. If your reply is yes to any, please give details below.

No

I agree to the open peer review policy of the journal. I understand that my name will be included on my report to the authors and, if the manuscript is accepted for publication, my named report including any attachments I upload will be posted on the website along with the authors' responses. I agree for my report to be made available under an Open Access Creative Commons CC-BY license (http://creativecommons.org/licenses/by/4.0/). I understand that any comments which I do not wish to be included in my named report can be included as confidential comments to the editors, which will not be published.

I agree to the open peer review policy of the journal

To further support our reviewers, we have joined with Publons, where you can gain additional credit to further highlight your hard work (see: https://publons.com/journal/530/gigascience). On publication of this paper, your review will be automatically added to Publons, you can then choose whether or not to claim your Publons credit. I understand this statement.

Yes Choose an item.
