# Supplementary material for: Chromosome-level reference genome and alternative splicing atlas of moso bamboo (Phyllostachys edulis)
Source: Gigascience. 2018 Sep 8;7(10):giy115. doi: 10.1093/gigascience/giy115 (PMC6204424; doi:10.1093/gigascience/giy115)
Supplement: Reviewer_3_Report_Revision_1 -- Araxi Urrutia — 6/22/2018 Reviewed [file giy115_reviewer_3_report_revision_1.pdf]

Reviewer Report

Title: **Chromosome-level reference genome and alternative splicing atlas of moso bamboo (Phyllostachys edulis)**

Version: **Revision 1** **Date:** 6/22/2018

Reviewer name: **Araxi Urrutia**

Reviewer Comments to Author:

The manuscript presents a comprehensive assembly of the bamboo genome which provides assembled chromosomes; an improvement from the current more fragmented assembly for the species. It also identifies alternative splicing events from transcriptome data corresponding to 26 different tissues.
I believe that the data presented will be of great use to the scientific community, in particular those working on genomics and those interested in transcriptomics and alternative splicing.

Main comments:
1. FOCUS AND JUSTIFICATION. However, I think that study could be better justified and given a focus. While the relevance of having a more complete genome for an important plant is well justified, it is not clear how this relates to alternative splicing. There is also no justification as to why examining alternative splicing is important. For example, in the abstract it is stated that the paper assembles the genome and identifies alternative splicing events but does not explain WHY has alternative splicing was an important aspect to explore in a paper presenting a more complete assembly of the bambo genome. Thus, one has the impression that this paper contains two separate stories running side by side.
Perhaps one solution is to explain that gene duplication and alternative splicing are important drivers of functional evolution in genomes. That incomplete and fragmented scaffolds of genomes makes it difficult to assess patterns of gene duplication and that low coverage transcriptomes of only a handful of tissues does not allow to fully understand the extent of alternative splicing. Thus, having a fully assembled genome as well as an extensive RNA sequencing for recovering RNA isoforms is required. It is important to explain in detail WHY is alternative splicing important.

2. JUSTIFICATION AND ORDER OF SPECIFIC ANALYSES. It is unclear to me why the description of the transposable element content should be in the section discussing alternative splicing events rather than on the section describing the genome sequence obtained.

3. DISCUSSION. Justification and relevance of many of the tests done is more evident in the discussion some of this information would be better placed in the introduction or as brief sentences in the results so that the analyses make sense as they are presented.

More specific points
1. On the evolution of AS section specify how many orthologs were found when comparing between species and what percentage of the total number of genes in the bamboo this represents. Even if these numbers are shown in the figure/tables it is always helpful to get an idea of the patterns from the text alone.
2. On the evolution of AS section it is not clear what analysis was done to compare the ortholog genes in other species. If I understand correctly, the analyses tries to assess whether having an older ortholog is associated with higher or lower rates of alternative splicing in the bamboo genome? This needs to be better worded. It is also important to explain WHY would this pattern be interesting important to understand evolution of alternative splicing. There is also a reference to a "robust pattern", does this refer to past literature in other species? If so then this needs to be better explained.
If actual conservation or overall patterns of AS in other plants, rather than presence absence of ortholog genes, were compared to the bamboo then this needs to be better explained as at the moment it is very unclear.

3. WHY the authors examine proportions of AS events by type is not entirely clear. There is an extensive literature on the patterns of prevalence of AS types as well as the differences in their potential contribution to functional adaptation.
4. It is unclear what the correlations for CDS length and intron number, etc. involved. Is this to correlate these parameters among the bamboo with its ortholog in the other plants? It is also not explained WHY was this done.
5. In the last section of the results, the title implies that the EVOLUTION of gene families was assessed. However the text below does not give any details of how was this done or whether there has actually been an expansion and if so, with respect to WHAT species… It is also not explained WHY only 13 gene families were assessed.
6. The discussion states that the paper presents evidence consistent with the role of TE in driving AS. The results only present a description of the rates of AS and the presence of TEs. If one drives the other then some analysis to link the two should be presented.
7. I could not find in the results section a reference to the sample with more vigorous growth to have higher AS as it is stated in the introduction. Could this be made more prominent so that when reading the discussion this result can be easily found.
8. I think a better explanation of what is a poorly conserved dataset and a highly conserved dataset means.

Methods

Are the methods appropriate to the aims of the study, are they well described, and are necessary controls included? Choose an item.

Conclusions

Are the conclusions adequately supported by the data shown? Choose an item.

Reporting Standards

Does the manuscript adhere to the journal's guidelines on [minimum standards of reporting?]() Choose an item.

Choose an item.

Statistics

Are you able to assess all statistics in the manuscript, including the appropriateness of statistical tests used? Choose an item.

Quality of Written English

Please indicate the quality of language in the manuscript: Choose an item.

Declaration of Competing Interests

Please complete a declaration of competing interests, considering the following questions:

- Have you in the past five years received reimbursements, fees, funding, or salary from an organisation that may in any way gain or lose financially from the publication of this manuscript, either now or in the future?

- Do you hold any stocks or shares in an organisation that may in any way gain or lose financially from the publication of this manuscript, either now or in the future?
- Do you hold or are you currently applying for any patents relating to the content of the manuscript?
- Have you received reimbursements, fees, funding, or salary from an organization that holds or has applied for patents relating to the content of the manuscript?
- Do you have any other financial competing interests?
- Do you have any non-financial competing interests in relation to this paper?

If you can answer no to all of the above, write 'I declare that I have no competing interests' below. If your reply is yes to any, please give details below.

I declare that I have no competing interests

I agree to the open peer review policy of the journal. I understand that my name will be included on my report to the authors and, if the manuscript is accepted for publication, my named report including any attachments I upload will be posted on the website along with the authors' responses. I agree for my report to be made available under an Open Access Creative Commons CC-BY license (http://creativecommons.org/licenses/by/4.0/). I understand that any comments which I do not wish to be included in my named report can be included as confidential comments to the editors, which will not be published.

I agree to the open peer review policy of the journal

To further support our reviewers, we have joined with Publons, where you can gain additional credit to further highlight your hard work (see: https://publons.com/journal/530/gigascience). On publication of this paper, your review will be automatically added to Publons, you can then choose whether or not to claim your Publons credit. I understand this statement.

Yes Choose an item.
